# Hidden Markov model analysis of fluorescence blinking in fluorescently labeled DNA

**DOI:** 10.1038/s41598-026-40876-x

**Published:** 2026-02-27

**Authors:** Tatsuhiro Furuta, Shuya Fan, Tadao Takada, Yohei Kondo, Mamoru Fujitsuka, Atsushi Maruyama, Kiyohiko Kawai, Kazuma Nakamura

**Affiliations:** 1 Graduate School of Engineering, Kyushu Institute of Technology, 1-1 Sensui-cho, 804-8550 Tobata-ku, Kitakyushu, Fukuoka Japan; 2 SANKEN (The Institute of Scientific and Industrial Research), The University of Osaka, Mihogaoka 8-1, 567-0047 Ibaraki, Osaka Japan; 3 Department of Applied Chemistry, Graduate School of Engineering, University of Hyogo, 2167 Shosha, 671-2280 Himeji, Hyogo Japan; 4 Department of Life Science and Technology, Institute of Science Tokyo, 4259, Nagatsuta-cho, 226-8501 Midori-ku, Yokohama, Kanagawa Japan; 5 Department of Pharmacy, Tokyo University of Pharmacy and Life Sciences, Horinouchi, 192-0392 Hachioji, Japan; 6 Integrated Research Center for Energy and Environmental Technologies, Kyushu Institute of Technology, 1-1 Sensui-cho, 804-8550 Tobata-ku, Kitakyushu, Fukuoka Japan

**Keywords:** Hidden Markov model, Single-molecule fluorescence, Blinking dynamics, Photon statistics, Relaxation time, Biophysics, Physics

## Abstract

We investigate the transition processes between the emitting (ON) and non-emitting (OFF) states of fluorescent molecules using a machine-learning approach. In fluorescently labeled DNA, continuous fluorescence is observed under irradiation; however, the system occasionally transitions to a non-emitting state, often associated with a charge-separated configuration. The resulting fluorescence trajectories exhibit characteristic blinking behavior —alternating ON and OFF states— which is heavily obscured by various sources of noise, making reliable state classification challenging. Because such trajectories represent typical stochastic time-series data, advanced analytical techniques are required. In this study, we apply a hidden Markov model to extract hidden ON/OFF states from noisy fluorescence trajectories using the forward-filtered backward-blocking Gibbs sampling algorithm, and construct probability density functions of the ON- and OFF-state durations to characterize the blinking dynamics. From these distributions, the characteristic relaxation times are evaluated as 17.6 ms for the ON state and 7.8 ms for the OFF state. The relatively long OFF period indicates that the charge-separated state in the DNA-ATTO655 system is fairly stable, suggesting suppressed charge recombination. In addition, we discuss the characteristic timescale of the light absorption–emission process in the ON state in terms of the average photon count per time bin. These results provide new insights into the fluorescence dynamics of single DNA-fluorophore systems. Finally, we discuss the detailed conditions required for reliable time-series analysis in terms of the photon-count histogram shape and the time-bin width used in the trajectories.

## Introduction

Materials science relating to fluorescence phenomena has long been one of the most active research fields. The luminescent properties of quantum dots are expected to have many applications^1–5^due to the variable fluorescence as a function of the system size and a high fluorescence quantum yield. In addition, fluorescently labeled DNA, in which a fluorescent dye is embedded to a specific DNA sequence, is a most essential tool in molecular biochemistry^6^, biomedical research^7^, and biophysics^8^ to understand DNA dynamics and interactions.

**Fig. 1:**
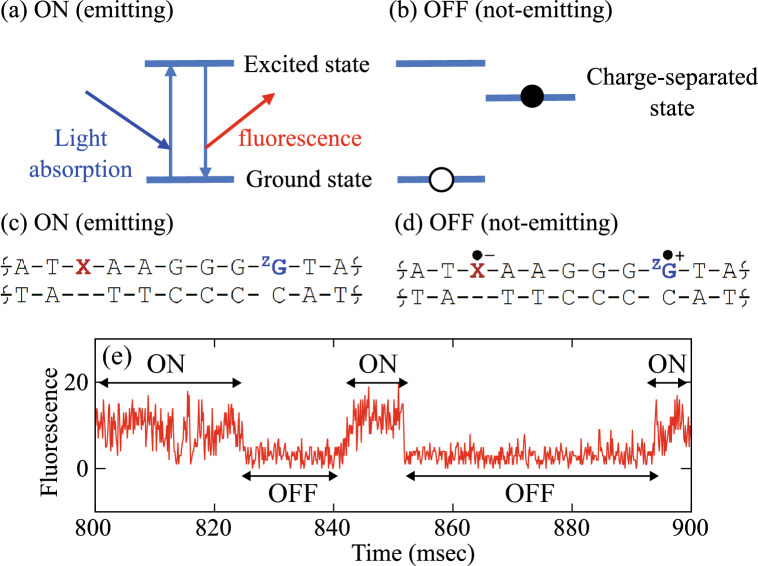
A schematic illustration of fluorescence blinking. Under irradiation, a fluorophore enters the ON (emitting) state [panel (**a**)], where it repeatedly undergoes light absorption and fluorescence emission. The system can also switch to the OFF (non-emitting) state [panel (**b**)], in which fluorescence emission is interrupted due to a transition to a charge-separated configuration. Panels (**c**) and (**d**) show schematic chemical structures of the fluorescently labeled DNA (double helix) analyzed in this study^13,14^. The symbols A, G, T, and C denote the nucleotide bases that constitute DNA, X represents the fluorescent dye (ATTO655), and ${}^{\textrm{Z}}\textrm{G}$ is a hole-trapping molecule. Panel (**c**) corresponds to the ON state, while panel (**d**) corresponds to the OFF state, where the molecule is trapped in a charge-separated state and does not emit light. See Section “Single-molecule measurement for fluorescence trajectory” for more details. Panel (**e**) shows a portion of the observed fluorescence photon-count trajectory, in which repeated ON and OFF segments can be seen within the noisy signal.

One of the most characteristic and important aspects of fluorescence is blinking^9–12^. The state in which a fluorescent molecule repeatedly absorbs light and emits fluorescence under irradiation is referred to as the ON state displayed in Fig. 1(a), and the state in which it does not emit light is called the OFF state in Fig. 1(b). When the fluorescence intensity, i.e., detected photon count, is monitored as a function of time, the ON and OFF states alternate repeatedly, a phenomenon known as fluorescence blinking, and the time series data shown in Fig. 1(e) are generated^13,14^. This time series records the durations of the ON and OFF states. The blinking plot is obtained by calculating a probability density function of the duration of each states, providing a basis to understand the fluorescent properties of materials. It should be noted that the fluorescence trajectory contains substantial noise, including background emission from the substrate, detector noise such as dark current, and intrinsic shot noise. When the noise becomes larger than the fluorescent signal, it becomes difficult to distinguish between the ON and OFF states, and the quantitative nature of the evaluated duration is ambiguous. As a result, the reliability of the blinking plot is also lost. Therefore, a data analysis method for extracting meaning signals and reliably identifying the ON/OFF states is highly desirable.

Machine learning has been increasingly applied in recent materials science research. However, most efforts have focused on the correlation analysis of static properties, while the analysis of dynamic (time-dependent) data remains relatively limited in material science. Yet, dynamic data analysis is a fundamentally important challenge and has been actively utilized in other fields such as stock price prediction^15^and equipment anomaly detection^16^. The Hidden Markov Model (HMM) is a classical statistical learning method that has been widely used for time-series analysis. Although its use in materials science is still relatively rare, several applications have been reported for quantum dots^17^, random telegraph noise^18–22^, nitrogen-vacancy centers in diamond^23,24^, electron holograms^25^, electron holography^26^, atomic quantum jumps^27^, and crack propagation^28^. HMM introduces hidden variables that represent the underlying states behind observed data, making it particularly powerful for analyzing noisy fluorescence trajectories. Such trajectories are inherently stochastic and affected by noise, so it is essential to uncover the physical mechanisms that give rise to the observed behavior. In this framework, ON and OFF states can be identified via the hidden-state time series instead of the raw fluorescence data. Importantly, the inferred hidden-state time series reduces the ambiguity caused by measurement noise, allowing for more robust evaluation of ON/OFF durations and blinking plots.

Fluctuations and correlations in the fluorescence intensity of fluorescently labeled DNA provide valuable information about molecular composition, conformation, diffusion, dynamics, intramolecular interactions, and environmental influences^29^. To extract such information from time-series data, analytical methods based on the autocorrelation function^13,14,30,31^, photon-counting histograms^32^, and change-point analysis^33,34^have been widely utilized. These approaches have contributed significantly to the understanding of conformational dynamics in single proteins^35^, enzymatic turnover in single flavoenzyme molecules^36^, and electron transfer reactions in azurin molecules^37^. More recently, HMM analysis has also been employed to study dynamic molecular behavior, particularly in fluorescence resonance energy transfer (FRET) (Refs^38–43^.), nanoparticle diffusion in cytoplasmic environments^44^, biomolecular motors^45^, and multichromophore photobleaching processes in polymer systems^46^. These studies highlight the versatility of HMM in capturing state transitions embedded in noisy fluorescence time series. However, despite its advantages, systematic HMM studies for fluorescence photon-count trajectories, especially under high temporal resolution and low signal-to-noise conditions, remain limited. In particular, the factors determining the stability and reliability of HMM-based state inference have not been systematically addressed.

In this study, we demonstrate that a suitably constructed HMM framework enables robust extraction of hidden ON/OFF states and their duration statistics from single-molecule fluorescence trajectories, thereby providing deeper insights into the underlying molecular dynamics. We apply this analysis to fluorescence blinking observed in a DNA system labeled with the fluorophore ATTO655 acting as a photosensitizer. First, we describe the HMM computational framework in detail, including the method for constructing the blinking plot, along with the experimental setup for single-molecule fluorescence measurements. Next, we report computational results for the experimental trajectories and perform exponential fitting of the ON- and OFF-state duration probability densities to evaluate characteristic relaxation times. The extracted photon dynamics are discussed from a quantitative point of view. In addition, we clarify that the shape of the photon-count histogram, which reflects the emission distinguishability between ON and OFF states, plays a decisive role in the stability of HMM analyses. By systematically varying the time-bin width and examining the resulting histogram structure, we show that the emergence of a bimodal distribution is a key prerequisite for reliable state identification, while unimodal distributions lead to noisy hidden-state sequences due to inherent identifiability limits. This perspective provides a simple and practical criterion for selecting appropriate time-bin widths and assessing analysis reliability in fluorescence trajectory studies. Finally, we summarize the conclusions of this study.

## Methods

### Hidden Markov model

The present HMM framework is based on our previous formulation developed for quantum-dot blinking dynamics^17^. However, there are two important differences. First, the inference algorithm for the hidden state sequence has been modified. While Ref^17^. employed a marginal-based Gibbs sampling scheme, the present work adopts forward-filtered backward sampling^47,48^, which preserves the Markov structure of the hidden states. This modification significantly improves robustness against noise and accelerates convergence, which is essential for the analysis of noisy fluorescence time series of dye-labeled DNA. Second, in contrast to the concise presentation in Ref^17^., the present manuscript provides a detailed derivation of the conditional probability expressions underlying the algorithm. This is intended to facilitate reproducibility and practical implementation by readers.

The aim of the HMM modeling is to estimate the underlying ON/OFF state sequence and the emission parameters without explicitly incorporating the time-bin width $\Delta$ into the model formulation. The HMM is a mixture model involving multiple stochastic variables, and the joint distribution of the model employed in the present study is expressed as follows:

1 $$\begin{aligned} p({\boldsymbol{I}}, \boldsymbol{S}, \boldsymbol{\Theta }, \boldsymbol{\pi }, \boldsymbol{A}) = p({\boldsymbol{I}}|\boldsymbol{S}, \boldsymbol{\Theta }) p(\boldsymbol{S}|\boldsymbol{\pi },\boldsymbol{A}) p(\boldsymbol{\Theta }) p(\boldsymbol{\pi }) p(\boldsymbol{A}). \end{aligned}$$

**Fig. 2:**
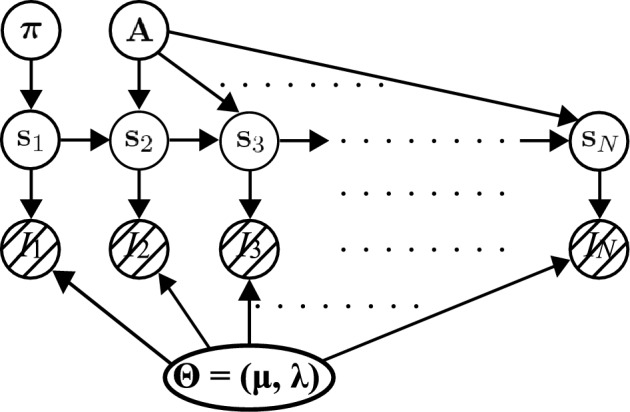
A graphical model for HMM defined as Eq. (1): {$I_n$} are observed fluorescence trajectory in Eq. (2), and {$\boldsymbol{s}_n$} are a time series of hidden variables in Eq. (3). ${\boldsymbol{\Theta }}$ = ($\boldsymbol{\mu }$, $\boldsymbol{\lambda })$ in Eq. (1) are parameters characterizing the intensity distributions that the fluorescence trajectory {$I_n$} follows. An initial-step hidden variable $\boldsymbol{s}_1$ is generated based on the parameter $\boldsymbol{\pi }$ in Eq. (1), while the *n*-th step $\boldsymbol{s}_n$ is generated by the previous-step $\boldsymbol{s}_{n-1}$, based on the Markov process, and the transition matrix $\boldsymbol{A}$ in Eq. (1). Stochastic variables are represented with nodes and the dependency between the variables is represented by arrows. The observed data is drawn with a shadow.

Here,

2 $$\begin{aligned} {\boldsymbol{I}}=(I_1, I_2, \cdots , I_N) \end{aligned}$$

is observed data corresponding to fluorescence trajectory to be analyzed, and $I_n$ describes detected photon numbers in the *n*-th time bin. *N* is the total numbers of the time grids, describing the trajectory length. $\boldsymbol{S}$ is a time series of hidden variables as

3 $$\begin{aligned} \boldsymbol{S} = (\boldsymbol{s}_1, \boldsymbol{s}_2, \cdots , \boldsymbol{s}_N), \end{aligned}$$

in which $\boldsymbol{s}_n$ describes the internal state at the *n*-th time step in the fluorescence trajectory; $\boldsymbol{s}_n$ is a *K*-dimensional vector in the one-hot representation (see below). To identify an ON or OFF state, we set *K* = 2. Essentially, the number of photons detected within a time bin determines whether the state is ON or OFF. $\boldsymbol{\Theta }$ describes parameters characterizing an emission distribution of observed data ${\boldsymbol{I}}$. In the present study, we assume that the distribution of each state follows the Gaussian distribution with a mean $\mu$ and a precision $\lambda$, where $\lambda ^{-1}$ represents a variance. Then, we write $\boldsymbol{\Theta }$ as $\{{\boldsymbol{\mu }}, {\boldsymbol{\lambda }}\}$ with ${\boldsymbol{\mu }}=(\mu _{\textrm{ON}}, \mu _{\textrm{OFF}})$ and ${\boldsymbol{\lambda }}=(\lambda _{\textrm{ON}}, \lambda _{\textrm{OFF}})$. In general, photon counting follows Poisson statistics. However, when the number of detected photons within a time bin becomes sufficiently large, the Poisson distribution can be well approximated by a Gaussian distribution, as a consequence of the central limit theorem. Based on this approximation, the emission probability is modeled by a Gaussian distribution. $\boldsymbol{\pi }$ represents the probability of the initial-step hidden variable, and $\boldsymbol{\pi } = (\pi _{\textrm{ON}}, \pi _{\textrm{OFF}})$. The $\boldsymbol{A}$ matrix describes a 2$\times$2 transition matrix for the time evolution of the hidden variables. $p(\boldsymbol{S}|\boldsymbol{\pi }, \boldsymbol{A})$ in Eq. (1) describes the conditional probability distribution of $\boldsymbol{S}$ with $\boldsymbol{A}$ and $\boldsymbol{\pi }$ fixed, and similarly, $p(\boldsymbol{I}|\boldsymbol{S},\boldsymbol{\Theta })$ is the conditional probability distribution of $\boldsymbol{I}$ after $\boldsymbol{S}$ and $\boldsymbol{\Theta }$ were determined. A graphical model of the present joint distribution in Eq. (1) is shown in Fig. 2, where the relations among the stochastic variables are depicted by arrows.

The explicit form of $p({\boldsymbol{I}}|\boldsymbol{S}, \boldsymbol{\Theta })=p({\boldsymbol{I}}|\boldsymbol{S}, \boldsymbol{\mu }, \boldsymbol{\lambda })$ in Eq. (1) is given as

4 $$\begin{aligned} p(\boldsymbol{I}|\boldsymbol{S}, \boldsymbol{\mu },\boldsymbol{\lambda }) = \prod _{n=1}^N p(I_n| \boldsymbol{s}_n, \boldsymbol{\mu },\boldsymbol{\lambda }) = \prod _{n=1}^N \prod _{k=1}^K p(I_n|\mu _k,\lambda _k)^{s_{kn}}, \end{aligned}$$

where we used a one-hot representation for the hidden state $\boldsymbol{s}_n$; it at time step *n* is represented by a one-hot vector $\boldsymbol{s}_n=(s_{1n}, s_{2n}, \cdots , s_{Kn})^T$. In this representation $s_{kn}$ = 0 or 1, and exactly one component is 1:

5 $$\begin{aligned} \sum _{k}s_{kn}=1. \end{aligned}$$

This one-hot encoding allows us to express discrete state variables using vector notation, which is particularly convenient for matrix-based formulations of the HMM. The emission probability of $p(I_{n}|\mu _k, \lambda _k)$ in Eq. (4) is assumed to the Gaussian distribution as

6 $$\begin{aligned} p(I_{n}|\mu _k, \lambda _k) = \mathscr {N}(I_n|\mu _k, \lambda _k^{-1}) = \sqrt{\frac{\lambda _k}{2\pi }} \exp \left( -\frac{\lambda _k}{2}(I_n-\mu _k)^2\right) . \end{aligned}$$

$p({\boldsymbol{S}}|\boldsymbol{\pi }, \boldsymbol{A})$ is described as

7 $$\begin{aligned} p(\boldsymbol{S}|\boldsymbol{\pi },\boldsymbol{A}) = p(\boldsymbol{s}_1|\boldsymbol{\pi })\prod _{n=2}^N p(\boldsymbol{s}_{n}|\boldsymbol{s}_{n-1}, \boldsymbol{A}), \end{aligned}$$

where $p(\boldsymbol{s}_1|\boldsymbol{\pi })$ and $p(\boldsymbol{s}_{n}|\boldsymbol{s}_{n-1}, \boldsymbol{A})$ are described with a categorical distribution as

8 $$\begin{aligned} p(\boldsymbol{s}_1|\boldsymbol{\pi }) = \prod _{k=1}^K \pi _k^{s_{k1}} \end{aligned}$$

and

9 $$\begin{aligned} p(\boldsymbol{s}_{n}|\boldsymbol{s}_{n-1}, \boldsymbol{A}) = \prod _{k=1}^K \prod _{\ell =1}^K A_{k\ell }^{s_{kn}s_{\ell n-1}}, \end{aligned}$$

respectively.

The prior distribution of the emission parameter $p(\boldsymbol{\mu },\boldsymbol{\lambda })$ is given as

10 $$\begin{aligned} p(\boldsymbol{\mu },\boldsymbol{\lambda }) = \prod _{k=1}^K p(\mu _{k},\lambda _{k}), \end{aligned}$$

and $p(\mu _k, \lambda _k)$ is described by the Gauss-Gamma distribution as

11 $$\begin{aligned} p(\mu _k,\lambda _k)\equiv & \mathscr {N}\textrm{G}(\mu _{k},\lambda _{k}|m,\nu , a,b) \nonumber \\= & \mathscr {N}(\mu _{k}|m,(\nu \lambda _{k})^{-1}) \textrm{Gam}(\lambda _{k}|a,b) \nonumber \\= & \left( \sqrt{\frac{\nu \lambda _{k}}{2\pi }} \exp { \left( -\frac{\nu \lambda _{k}}{2}(\mu _{k}-m)^2\right) }\right) \left( C_G(a,b)\lambda _{k}^{a-1}e^{-b\lambda _{k}} \right) , \end{aligned}$$

where $\nu$, *m*, *a*, and *b* are hyperparameters, and $C_G(a,b)$ is a normalization constant of the Gamma distribution.

$p(\boldsymbol{A})$ is the prior distribution of transition matrix $\boldsymbol{A}$, which is given as the product of the Dirichlet distribution as

12 $$\begin{aligned} p(\boldsymbol{A}) = \prod _{k=1}^K p(\boldsymbol{A}_{k}), \end{aligned}$$

where $p(\boldsymbol{A}_{k})$ is expressed by a Dirichlet distribution as

13 $$\begin{aligned} p(\boldsymbol{A}_k)= C_D(\boldsymbol{\beta }_{k})\prod _{\ell =1}^K A_{k \ell }^{\beta _{k \ell }-1} \end{aligned}$$

with $\boldsymbol{\beta }_k$ and $C_D(\boldsymbol{\beta }_k)$ being hyperparameters and normalization constant, respectively.

Lastly, $p(\boldsymbol{\pi })$ denotes the prior distribution of the initial-state probability vector $\boldsymbol{\pi }$, which is also given by the Dirichlet distribution as

14 $$\begin{aligned} p(\boldsymbol{\pi }) = C_D(\boldsymbol{\alpha }) \prod _{k=1}^K \pi _k^{\alpha _k - 1}, \end{aligned}$$

where $\boldsymbol{\alpha }$ and $C_D(\boldsymbol{\alpha })$ are the hyperparameters and the normalization constant, respectively.

By inserting Eqs. (4), (7), (10), (12) into Eq. (1), we obtain

15 $$\begin{aligned} & p(\boldsymbol{I}, \boldsymbol{S}, \boldsymbol{\mu }, \boldsymbol{\lambda }, \boldsymbol{\pi }, \boldsymbol{A}) \nonumber \\= & \left( \prod _{n=1}^N \prod _{k=1}^K p(I_n|\mu _k,\lambda _k)^{s_{kn}}\right) \left( p(\boldsymbol{s}_1|\boldsymbol{\pi })\prod _{n=2}^N p(\boldsymbol{s}_{n}|\boldsymbol{s}_{n-1}, \boldsymbol{A})\right) \left( \prod _{k=1}^K p(\mu _{k},\lambda _{k})\right) p(\boldsymbol{\pi }) \left( \prod _{k=1}^K p(\boldsymbol{A}_{k})\right) . \end{aligned}$$

### Gibbs sampling

The purpose of the HMM simulation is to infer the hidden-variable time series $\boldsymbol{S}$ in Eq. (3) with the observed fluorescence trajectory ${\boldsymbol{I}}$ in Eq. (2). For this purpose, we need to calculate a posterior distribution which is a conditional distribution function under the observed $\boldsymbol{I}$ as

16 $$\begin{aligned} p(\boldsymbol{S},{\boldsymbol{\mu }}, {\boldsymbol{\lambda }}, \boldsymbol{\pi }, \boldsymbol{A}|\boldsymbol{I}) =\frac{p(\boldsymbol{I}, \boldsymbol{S}, \boldsymbol{\mu }, \boldsymbol{\lambda }, \boldsymbol{\pi }, \boldsymbol{A})}{p(\boldsymbol{I})}, \end{aligned}$$

where $p(\boldsymbol{I})$ is the marginal distribution written as

17 $$\begin{aligned} p(\boldsymbol{I}) =\sum _{\boldsymbol{S}} \int p(\boldsymbol{I}, \boldsymbol{S}, \boldsymbol{\mu }, \boldsymbol{\lambda }, \boldsymbol{\pi }, \boldsymbol{A}) d\boldsymbol{\mu } d\boldsymbol{\lambda } d\boldsymbol{\pi } d\boldsymbol{A}. \end{aligned}$$

To approximately calculate the posterior distribution in Eq. (16), we use a Gibbs sampling^49,50^ based on Bayesian inference. The Gibbs sampling considers the conditional probability distribution of one variable given that other random variables are fixed. It is ensured that the Gibbs sampling leads to a well converged result to the true posterior distribution in Eq. (16), when the sampling is large enough.

Gibbs sampling proceeds as follows. At iteration *i* we draw each block from its full conditional:

1. Sample the hidden state sequence from the full conditional given the current parameters and data,
  18 $$\begin{aligned} \boldsymbol{S}^{(i)} \sim p\!\left( \boldsymbol{S}\mid \boldsymbol{\mu }^{(i-1)},\boldsymbol{\lambda }^{(i-1)},\boldsymbol{\pi }^{(i-1)},\boldsymbol{A}^{(i-1)},\boldsymbol{I}\right) . \end{aligned}$$
  Initial values $\boldsymbol{\mu }^{(0)}$, $\boldsymbol{\lambda }^{(0)}$, $\boldsymbol{\pi }^{(0)}$, and $\boldsymbol{A}^{(0)}$ are set from the hyperparameters in Table 1.
2. Sample emission parameters,
  19 $$\begin{aligned} \boldsymbol{\mu }^{(i)},\boldsymbol{\lambda }^{(i)} \sim p\!\left( \boldsymbol{\mu },\boldsymbol{\lambda }\mid \boldsymbol{S}^{(i)},\boldsymbol{\pi }^{(i-1)},\boldsymbol{A}^{(i-1)},\boldsymbol{I}\right) . \end{aligned}$$
3. Sample the initial-state distribution,
  20 $$\begin{aligned} \boldsymbol{\pi }^{(i)} \sim p\!\left( \boldsymbol{\pi }\mid \boldsymbol{S}^{(i)},\boldsymbol{\mu }^{(i)},\boldsymbol{\lambda }^{(i)},\boldsymbol{A}^{(i-1)},\boldsymbol{I}\right) . \end{aligned}$$
4. Sample the transition matrix,
  21 $$\begin{aligned} \boldsymbol{A}^{(i)} \sim p\!\left( \boldsymbol{A}\mid \boldsymbol{S}^{(i)},\boldsymbol{\mu }^{(i)},\boldsymbol{\lambda }^{(i)},\boldsymbol{\pi }^{(i)},\boldsymbol{I}\right) . \end{aligned}$$

One iteration is Eq. (18) $\rightarrow$ (19) $\rightarrow$ (20) $\rightarrow$ (21); repeating these draws generates a Markov chain targeting the joint posterior over $(\boldsymbol{S},\boldsymbol{\mu },\boldsymbol{\lambda },\boldsymbol{\pi },\boldsymbol{A})$. In the sampling step 1 for $\boldsymbol{S}$, we use a blocking Gibbs sampling based on the forward-filtered backward sampling algorithm^47,48^.

For monitoring we track a per-sample surrogate objective of log likelihood $\mathscr {L}$ (natural logarithms; units are nats/sample),

22 $$\begin{aligned} \mathscr {L}= & \frac{1}{N}\,\ln \!\Big \{p\!\left( \boldsymbol{I}\mid \boldsymbol{S}^{(i)},\boldsymbol{\mu }^{(i)},\boldsymbol{\lambda }^{(i)}\right) \,p\!\left( \boldsymbol{S}^{(i)}\mid \boldsymbol{\pi }^{(i)},\boldsymbol{A}^{(i)}\right) \Big \} \nonumber \\= & \frac{1}{N}\sum _{k=1}^{\!K}\sum _{n=1}^{\!N} s_{k n}^{(i)} \left( \frac{1}{2}\ln \frac{\lambda _k^{(i)}}{2\pi } -\frac{\lambda _k^{(i)}}{2}\big (I_n-\mu _k^{(i)}\big )^2\right) +\frac{1}{N}\sum _{k=1}^{\!K} s_{k1}^{(i)}\ln \pi _k^{(i)} +\frac{1}{N}\sum _{k=1}^{\!K}\sum _{\ell =1}^{\!K}\sum _{n=2}^{\!N} s_{k n}^{(i)}\,s_{\ell n-1}^{(i)}\,\ln A_{k\ell }^{(i)}. \end{aligned}$$

The three terms correspond to the emission, initial-state, and transition contributions, respectively. Basically, it turns out that the present optimization of random variables is quite stable.

### Posterior distribution of hidden variables $\boldsymbol{S}$

We first consider the derivation of the posterior distribution of hidden variables $\boldsymbol{S}$. The conditional distribution of hidden sequence $\boldsymbol{S}$ given all the parameters and observations is transformed as follows:

23 $$\begin{aligned} p(\boldsymbol{S}|\boldsymbol{\mu },\boldsymbol{\lambda },\boldsymbol{\pi },\boldsymbol{A},\boldsymbol{I}) = \frac{p(\boldsymbol{S}, \boldsymbol{\mu },\boldsymbol{\lambda },\boldsymbol{\pi },\boldsymbol{A},\boldsymbol{I})}{p(\boldsymbol{\mu },\boldsymbol{\lambda },\boldsymbol{\pi },\boldsymbol{A},\boldsymbol{I})} \propto p(\boldsymbol{S}, \boldsymbol{I}, \boldsymbol{\mu }, \boldsymbol{\lambda }, \boldsymbol{\pi }, \boldsymbol{A}) \propto p(\boldsymbol{I}|\boldsymbol{S},\boldsymbol{\mu },\boldsymbol{\lambda })p(\boldsymbol{S}|\boldsymbol{\pi },\boldsymbol{A}). \end{aligned}$$

This relation shows that the posterior distribution of $\boldsymbol{S}$ is independent of the parameters $\boldsymbol{\mu },\boldsymbol{\lambda },\boldsymbol{\pi },\boldsymbol{A}$ once the likelihood $p(\boldsymbol{I}|\boldsymbol{S},\boldsymbol{\mu },\boldsymbol{\lambda })$ and the state prior $p(\boldsymbol{S}|\boldsymbol{\pi },\boldsymbol{A})$ are specified. The problem is inferring the hidden sequence $\boldsymbol{S}$, and the number of hidden variables is enormous, requiring an effective inference method. For this purpose, we use the forward-filtered backward sampling algorithm^47,48^ for the inference of $\boldsymbol{S}$. With Eqs. (4) and (7),

24 $$\begin{aligned} & p(\boldsymbol{S}|\boldsymbol{\mu },\boldsymbol{\lambda },\boldsymbol{\pi },\boldsymbol{A},\boldsymbol{I}) \sim p(\boldsymbol{I}|\boldsymbol{S},\boldsymbol{\mu },\boldsymbol{\lambda })p(\boldsymbol{S}|\boldsymbol{\pi },\boldsymbol{A})\nonumber \\= & \left( \prod _{n=1}^N p(I_{n}|\boldsymbol{s}_{n},\boldsymbol{\mu },\boldsymbol{\lambda })\right) \left( p(\boldsymbol{s}_1|\boldsymbol{\pi })\prod _{n=2}^N p(\boldsymbol{s}_{n}|\boldsymbol{s}_{n-1}, \boldsymbol{A})\right) \nonumber \\= & p(I_{1}|\boldsymbol{s}_{1},\boldsymbol{\mu },\boldsymbol{\lambda })p(\boldsymbol{s}_1|\boldsymbol{\pi }) \Biggl (\prod _{n'=2}^{n} p(I_{n'}|\boldsymbol{s}_{n'},\boldsymbol{\mu },\boldsymbol{\lambda })p(\boldsymbol{s}_{n'}|\boldsymbol{s}_{n'-1}, \boldsymbol{A}) \Biggr ) \Biggl (\prod _{n'=n+1}^{N} p(I_{n'}|\boldsymbol{s}_{n'},\boldsymbol{\mu },\boldsymbol{\lambda })p(\boldsymbol{s}_{n'}|\boldsymbol{s}_{n'-1}, \boldsymbol{A}) \Biggr ) \nonumber \\= & p(\boldsymbol{I}_{1:n}, \boldsymbol{S}_{1:n}) p(\boldsymbol{I}_{n+1:N}, \boldsymbol{S}_{n+1:N}|\boldsymbol{s}_{n}) \end{aligned}$$

with

25 $$\begin{aligned} p(\boldsymbol{I}_{1:n}, \boldsymbol{S}_{1:n}) \equiv p(I_{1}|\boldsymbol{s}_{1})p(\boldsymbol{s}_1|\boldsymbol{\pi }) \Biggl (\prod _{n'=2}^{n} p(I_{n'}|\boldsymbol{s}_{n'})p(\boldsymbol{s}_{n'}|\boldsymbol{s}_{n'-1}) \Biggr ) \end{aligned}$$

and

26 $$\begin{aligned} p(\boldsymbol{I}_{n+1:N}, \boldsymbol{S}_{n+1:N}|\boldsymbol{s}_{n}) \equiv \prod _{n'=n+1}^{N} p(I_{n'}|\boldsymbol{s}_{n'})p(\boldsymbol{s}_{n'}|\boldsymbol{s}_{n'-1}), \end{aligned}$$

where we introduce abbreviations as

27 $$\begin{aligned} p(I_{n}|\boldsymbol{s}_{n}) \equiv p(I_{n}|\boldsymbol{s}_{n},\boldsymbol{\mu },\boldsymbol{\lambda }) \end{aligned}$$

and

28 $$\begin{aligned} p(\boldsymbol{s}_{n}|\boldsymbol{s}_{n-1}) \equiv p(\boldsymbol{s}_{n}|\boldsymbol{s}_{n-1}, \boldsymbol{A}). \end{aligned}$$

Now, $p(\boldsymbol{s}_n|\boldsymbol{\mu },\boldsymbol{\lambda },\boldsymbol{\pi },\boldsymbol{A},\boldsymbol{I})$ is calculated by sum over other hidden variables than $\boldsymbol{s}_n$ in $\boldsymbol{S}$; from Eq. (24)

29 $$\begin{aligned} p(\boldsymbol{s}_n|\boldsymbol{\mu },\boldsymbol{\lambda },\boldsymbol{\pi },\boldsymbol{A},\boldsymbol{I})= & \sum _{\boldsymbol{s}_1} \sum _{\boldsymbol{s}_2} \cdots \sum _{\boldsymbol{s}_{n-1}} \sum _{\boldsymbol{s}_{n+1}} \cdots \sum _{\boldsymbol{s}_N} p(\boldsymbol{S}|\boldsymbol{\mu },\boldsymbol{\lambda },\boldsymbol{\pi },\boldsymbol{A},\boldsymbol{I}) \nonumber \\\sim & \sum _{\boldsymbol{S}_{1:n-1}} p(\boldsymbol{I}_{1:n}, \boldsymbol{S}_{1:n}) \sum _{\boldsymbol{S}_{n+1:N}} p(\boldsymbol{I}_{n+1:N}, \boldsymbol{S}_{n+1:N}|\boldsymbol{s}_{n}) \nonumber \\= & p(\boldsymbol{I}_{1:n}, \boldsymbol{s}_{n}) p(\boldsymbol{I}_{n+1:N}|\boldsymbol{s}_{n}) \nonumber \\= & f(\boldsymbol{s}_n)b(\boldsymbol{s}_n) \end{aligned}$$

with

30 $$\begin{aligned} \sum _{\boldsymbol{S}_{1:n-1}} =\sum _{\boldsymbol{s}_{1}} \sum _{\boldsymbol{s}_{2}} \cdots \sum _{\boldsymbol{s}_{n-1}} \end{aligned}$$

and

31 $$\begin{aligned} \sum _{\boldsymbol{S}_{n+1:N}} =\sum _{\boldsymbol{s}_{n+1}} \sum _{\boldsymbol{s}_{n+2}} \cdots \sum _{\boldsymbol{s}_{N}}. \end{aligned}$$

Also, $\sum _{\boldsymbol{s}_n}$ stands for the pattern sum of the one-hot vector $\boldsymbol{s}_n$ over $(1, 0, \cdots , 0)$, $(0, 1, \cdots , 0)$, $\cdots$, and $(0, 0, \cdots , 1)$. In Eq. (29), the forward probability $f(\boldsymbol{s}_n)$ and the backward probability $b(\boldsymbol{s}_n)$ are defined by

32 $$\begin{aligned} f(\boldsymbol{s}_n) \equiv p(\boldsymbol{I}_{1:n}, \boldsymbol{s}_{n}) = \sum _{\boldsymbol{S}_{1:n-1}} p(\boldsymbol{I}_{1:n}, \boldsymbol{S}_{1:n}) = p(I_{n}|\boldsymbol{s}_{n}) \sum _{\boldsymbol{S}_{1:n-1}} p(\boldsymbol{s}_{n}|\boldsymbol{s}_{n-1})p(\boldsymbol{I}_{1:n-1}, \boldsymbol{S}_{1:n-1}) \end{aligned}$$

and

33 $$\begin{aligned} b(\boldsymbol{s}_n) \equiv p(\boldsymbol{I}_{n+1:N}|\boldsymbol{s}_{n}) = \sum _{\boldsymbol{S}_{n+1:N}} p(\boldsymbol{I}_{n+1:N}, \boldsymbol{S}_{n+1:N}|\boldsymbol{s}_{n}), \end{aligned}$$

respectively. Now, about $f(\boldsymbol{s}_n)$ in Eq. (32), since $p(\boldsymbol{I}_{1:n-1}, \boldsymbol{S}_{1:n-1})$ has a recurrence structure by definition in Eq. (25) as

34 $$\begin{aligned} p(\boldsymbol{I}_{1:n-1}, \boldsymbol{S}_{1:n-1}) = p(I_{n-1}|\boldsymbol{s}_{n-1}) p(\boldsymbol{s}_{n-1}|\boldsymbol{s}_{n-2}) p(\boldsymbol{I}_{1:n-2}, \boldsymbol{S}_{1:n-2}), \end{aligned}$$

by inserting Eq. (34) into Eq. (32), we obtain a recurrence formula for $f(\boldsymbol{s}_n)$ as

35 $$\begin{aligned} f(\boldsymbol{s}_n)= & p(I_{n}|\boldsymbol{s}_{n}) \sum _{\boldsymbol{S}_{1:n-1}} p(\boldsymbol{s}_{n}|\boldsymbol{s}_{n-1}) p(I_{n-1}|\boldsymbol{s}_{n-1}) p(\boldsymbol{s}_{n-1}|\boldsymbol{s}_{n-2}) p(\boldsymbol{I}_{1:n-2}, \boldsymbol{S}_{1:n-2})\nonumber \\= & p(I_{n}|\boldsymbol{s}_{n}) \sum _{\boldsymbol{s}_{n-1}} p(\boldsymbol{s}_{n}|\boldsymbol{s}_{n-1}) \left( p(I_{n-1}|\boldsymbol{s}_{n-1})\sum _{\boldsymbol{S}_{1:n-2}}p(\boldsymbol{s}_{n-1}|\boldsymbol{s}_{n-2})p(\boldsymbol{I}_{1:n-2}, \boldsymbol{S}_{1:n-2})\right) \nonumber \\= & p(I_{n}|\boldsymbol{s}_{n}) \sum _{\boldsymbol{s}_{n-1}} p(\boldsymbol{s}_{n}|\boldsymbol{s}_{n-1})f(\boldsymbol{s}_{n-1}). \end{aligned}$$

We next consider a concrete calculation for the forward probability $f(\boldsymbol{s}_n)$, assuming that it takes a form of the categorical distribution as

36 $$\begin{aligned} f(\boldsymbol{s}_n) \equiv p(\boldsymbol{s}_n|\boldsymbol{f}_n) = \prod _{k=1} ^{K} f_{kn} ^{s_{kn}}. \end{aligned}$$

Based on the above, Eq. (35) is expressed as

37 $$\begin{aligned} p(\boldsymbol{s}_n|\boldsymbol{f}_n)= & p(I_{n}|\boldsymbol{s}_{n}) \sum _{\boldsymbol{s}_{n-1}} p(\boldsymbol{s}_{n}|\boldsymbol{s}_{n-1}) p(\boldsymbol{s}_{n-1}|\boldsymbol{f}_{n-1}) \nonumber \\= & p(I_{n}|\boldsymbol{s}_{n}) \sum _{\boldsymbol{s}_{n-1}} \left( \prod _{k=1}^K \prod _{\ell =1}^K A_{k\ell }^{s_{kn}s_{\ell n-1}} \right) \left( \prod _{\ell '=1}^K f_{\ell ' n-1}^{s_{\ell ' n-1}} \right) \nonumber \\= & p(I_{n}|\boldsymbol{s}_{n}) \sum _{\boldsymbol{s}_{n-1}} \prod _{k=1}^K \prod _{\ell =1}^K \left( A_{k \ell }^{s_{kn}} f_{\ell n-1} \right) ^{s_{\ell n-1}} \nonumber \\= & p(I_{n}|\boldsymbol{s}_{n}) \sum _{\ell =1}^K \prod _{k=1}^K A_{k \ell }^{s_{kn}}f_{\ell n-1}, \end{aligned}$$

where we used Eq. (9) for $p(\boldsymbol{s}_{n}|\boldsymbol{s}_{n-1})$. Further, with using Eqs. (4) and (6), Eq. (37) reads as

38 $$\begin{aligned} p(\boldsymbol{s}_n|\boldsymbol{f}_n)= & \left( \prod _{k'=1}^K \mathscr {N}(I_n|\mu _{k'}, \lambda _{k'}^{-1})^{s_{k' n}}\right) \sum _{\ell =1}^K \prod _{k=1}^K A_{k \ell }^{s_{kn}}f_{\ell n-1}, \nonumber \\= & \sum _{\ell =1}^K \left( \left( \mathscr {N}(I_n|\mu _1, \lambda _1^{-1}) A_{1 \ell } \right) ^{s_{1n}} \left( \mathscr {N}(I_n|\mu _2, \lambda _2^{-1}) A_{2 \ell } \right) ^{s_{2n}} \cdots \left( \mathscr {N}(I_n|\mu _K, \lambda _K^{-1}) A_{K \ell } \right) ^{s_{Kn}} \right) f_{\ell n-1} \nonumber \\= & \prod _{k=1}^K f_{kn}^{s_{kn}} \end{aligned}$$

with

39 $$\begin{aligned} f_{kn} = \sum _{\ell =1}^K \mathscr {N}(I_n|\mu _k, \lambda _k^{-1}) A_{k \ell } f_{\ell n-1}. \end{aligned}$$

Let us now consider the backward-sampling part of the forward-filtered backward Gibbs sampling procedure. For this purpose, we start from the posterior distribution at two neighboring time steps,

40 $$\begin{aligned} p(\boldsymbol{s}_n, \boldsymbol{s}_{n+1}, \boldsymbol{\mu }, \boldsymbol{\lambda }, \boldsymbol{\pi }, \boldsymbol{A}, \boldsymbol{I})\equiv & p(\boldsymbol{s}_n, \boldsymbol{s}_{n+1}, I_{1:N}) \nonumber \\= & \sum _{\boldsymbol{S}_{1:n-1}} \sum _{\boldsymbol{S}_{n+2:N}} p(\boldsymbol{I}_{1:N}, \boldsymbol{S}_{1:N}) \nonumber \\= & f(\boldsymbol{s}_n)\, p(I_{n+1}|\boldsymbol{s}_{n+1})\, p(\boldsymbol{s}_{n+1}|\boldsymbol{s}_{n})\, b(\boldsymbol{s}_{n+1}), \end{aligned}$$

where $f(\boldsymbol{s}_n)$ and $b(\boldsymbol{s}_{n+1})$ denote the forward and backward messages, respectively. The conditional distribution of the hidden state $\boldsymbol{s}_n$ given the next state $\boldsymbol{s}_{n+1}$ and the entire observation sequence is then written as

41 $$\begin{aligned} p(\boldsymbol{s}_n | \boldsymbol{s}_{n+1}, I_{1:N})= & \frac{p(\boldsymbol{s}_n, \boldsymbol{s}_{n+1}, I_{1:N})}{\sum _{\boldsymbol{s}_{n}} p(\boldsymbol{s}_{n}, \boldsymbol{s}_{n+1}, I_{1:N})} \nonumber \\\propto & f(\boldsymbol{s}_n)\, p(I_{n+1}|\boldsymbol{s}_{n+1})\, p(\boldsymbol{s}_{n+1}|\boldsymbol{s}_{n})\, b(\boldsymbol{s}_{n+1}) \nonumber \\\propto & f(\boldsymbol{s}_n)\, p(\boldsymbol{s}_{n+1}|\boldsymbol{s}_{n}), \end{aligned}$$

indicating that $p(\boldsymbol{s}_n | \boldsymbol{s}_{n+1}, I_{1:N})$ is proportional only to the forward probability and the transition probability. This conditional distribution is used for the backward sampling of the state sequence. The practical formula for backward sampling is derived as

42 $$\begin{aligned} p(\boldsymbol{s}_n | \boldsymbol{s}_{n+1}, I_{1:N})\propto & f(\boldsymbol{s}_n)\, p(\boldsymbol{s}_{n+1}|\boldsymbol{s}_{n}) \nonumber \\= & \left( \prod _{k=1}^K f_{kn}^{s_{kn}} \right) \left( \prod _{\ell =1}^{K} \prod _{k=1}^{K} A_{\ell k}^{s_{\ell n+1} s_{kn}} \right) \nonumber \\= & \prod _{k=1}^K \left( f_{kn} A_{\ell k} \right) ^{s_{kn}}, \end{aligned}$$

where $\ell = \arg \max (\boldsymbol{s}_{n+1})$ specifies the active state at time $n{+}1$. In the practical implementation, the normalization is introduced as

43 $$\begin{aligned} p(\boldsymbol{s}_n | \boldsymbol{s}_{n+1}, I_{1:N}) \equiv p(\boldsymbol{s}_n | \boldsymbol{\eta }_n) = \prod _{k=1}^K \eta _{kn}^{s_{kn}}, \end{aligned}$$

with

44 $$\begin{aligned} \eta _{kn} = \frac{f_{kn} A_{\ell k}}{\displaystyle \sum _{k'=1}^K f_{k'n} A_{\ell k'}}. \end{aligned}$$

Here, $\eta _{kn}$ represents the normalized backward-sampling weight of state *k* at time step *n*. Note that the evaluation of $\{\eta _{kn}\}$ requires the information of $\ell$, which corresponds to the active state at the next time step ($n+1$).

### Posterior distribution of the emission parameters $\boldsymbol{\mu }, \boldsymbol{\lambda }$

We next consider the posterior distribution of the emission parameters $\boldsymbol{\mu }$ and $\boldsymbol{\lambda }$. For this purpose, we consider the following chain rule of conditional distribution as

45 $$\begin{aligned} p(\boldsymbol{\mu }, \boldsymbol{\lambda }|\boldsymbol{S}, \boldsymbol{\pi }, \boldsymbol{A}, \boldsymbol{I}) =p(\boldsymbol{\mu }|\boldsymbol{\lambda }, \boldsymbol{S}, \boldsymbol{\pi }, \boldsymbol{A}, \boldsymbol{I}) p(\boldsymbol{\lambda }|\boldsymbol{S}, \boldsymbol{\pi }, \boldsymbol{A}, \boldsymbol{I}). \end{aligned}$$

**Posterior distribution for **$\boldsymbol{\mu }$**:**

We first derive the posterior distribution of the mean parameter $\boldsymbol{\mu }=(\mu _1, \mu _2, \cdots , \mu _K)$, assuming that the emission probability for state *k* is Gaussian with known precision $\lambda _k$. According to Bayes’ theorem, the posterior distribution of $\mu _k$ given the hidden sequence $\boldsymbol{S}$ and the observations $\boldsymbol{I}$ is proportional to the product of the likelihood and the prior; from Eq. (45),

46 $$\begin{aligned} p(\boldsymbol{\mu }|\boldsymbol{\lambda }, \boldsymbol{S}, \boldsymbol{\pi }, \boldsymbol{A}, \boldsymbol{I})= & \frac{p(\boldsymbol{\mu }, \boldsymbol{\lambda }|\boldsymbol{S}, \boldsymbol{\pi }, \boldsymbol{A}, \boldsymbol{I})}{p(\boldsymbol{\lambda }|\boldsymbol{S}, \boldsymbol{\pi }, \boldsymbol{A}, \boldsymbol{I})} \propto p(\boldsymbol{\mu }, \boldsymbol{\lambda }|\boldsymbol{S}, \boldsymbol{\pi }, \boldsymbol{A}, \boldsymbol{I}) = \frac{p(\boldsymbol{\mu }, \boldsymbol{\lambda }, \boldsymbol{S}, \boldsymbol{\pi }, \boldsymbol{A}, \boldsymbol{I})}{p(\boldsymbol{S}, \boldsymbol{\pi }, \boldsymbol{A}, \boldsymbol{I})} \propto p(\boldsymbol{\mu }, \boldsymbol{\lambda }, \boldsymbol{S}, \boldsymbol{\pi }, \boldsymbol{A}, \boldsymbol{I}) \nonumber \\\propto & p(\boldsymbol{I}|\boldsymbol{S}, \boldsymbol{\mu }, \boldsymbol{\lambda }) p(\boldsymbol{\mu }, \boldsymbol{\lambda }). \end{aligned}$$

By taking of logarithm of the above equation, we obtain

47 $$\begin{aligned} \ln p(\boldsymbol{\mu }|\boldsymbol{\lambda }, \boldsymbol{S}, \boldsymbol{\pi }, \boldsymbol{A}, \boldsymbol{I})= & \ln p(\boldsymbol{I}|\boldsymbol{S}, \boldsymbol{\mu }, \boldsymbol{\lambda }) p(\boldsymbol{\mu }, \boldsymbol{\lambda }) + \text {const.} \nonumber \\= & \ln \left( \prod _{n=1}^N \prod _{k=1}^K p(I_n|\mu _k,\lambda _k)^{s_{kn}}\right) \left( \prod _{k=1}^K p(\mu _{k},\lambda _{k}) \right) + \text {const.}\, \end{aligned}$$

where we used Eq. (4) for $p(\boldsymbol{I}|\boldsymbol{S}, \boldsymbol{\mu }, \boldsymbol{\lambda })$ and Eq. (10) for $p(\boldsymbol{\mu }, \boldsymbol{\lambda })$. The emission probability $p(I_n|\mu _k,\lambda _k)$ is expressed by the Gaussian distribution in Eq. (6), and the prior $p(\mu _{k},\lambda _{k})$ is described by the Gauss-Gamma distribution in Eq. (11), and thus we obtain

48 $$\begin{aligned} & \ln \left( \prod _{n=1}^N \prod _{k=1}^K \mathscr {N}(I_n|\mu _k,\lambda _k^{-1})^{s_{kn}}\right) \left( \prod _{k=1}^K \mathscr {N}\textrm{G}(\mu _{k},\lambda _{k}|m,\nu , a,b) \right) + \text {const.} \nonumber \\= & \sum _{k=1}^K \left( \sum _{n=1}^N s_{kn} \ln \mathscr {N}(I_n|\mu _k,\lambda _k^{-1}) + \ln \biggl (\mathscr {N}(\mu _{k}|m,(\nu \lambda _{k})^{-1}) \textrm{Gam}(\lambda _{k}|a,b)\biggr ) \right) + \text {const.} \nonumber \\= & \sum _{k=1}^K \Biggl ( \sum _{n=1}^N s_{kn} \ln \mathscr {N}(I_n|\mu _k,\lambda _k^{-1}) + \ln \mathscr {N}(\mu _{k}|m,(\nu \lambda _{k})^{-1}) \Biggr ) + \text {const.} \nonumber \\= & \sum _{k=1}^K \left( \sum _{n=1}^N s_{kn} \left( -\frac{\lambda _k}{2}(I_n-\mu _k)^2+\frac{1}{2}\ln {\lambda _k}-\frac{1}{2}\ln {2\pi } \right) + \left( -\frac{\nu \lambda _{k}}{2}(\mu _{k}-m)^2+\frac{1}{2}\ln {\nu }+\frac{1}{2}\ln {\lambda _k}-\frac{1}{2}\ln {2\pi }\right) \right) + \text {const.} \nonumber \\= & \sum _{k=1}^K \left( - \frac{\hat{\nu }_k \lambda _k}{2} (\mu _k-\hat{m}_k)^2 \right) + \text {const.}\, \end{aligned}$$

where $\hat{\nu }_k$ and $\hat{m}_k$ are given as

49 $$\begin{aligned} \hat{\nu }_k= \sum _{n=1}^N s_{kn}+ \nu , \end{aligned}$$

and

50 $$\begin{aligned} \hat{m}_k= \frac{1}{\hat{\nu }_k}\left( \sum _{n=1}^N s_{kn} I_n+\nu m \right) , \end{aligned}$$

respectively. From Eq. (48), the resulting posterior distribution for $\boldsymbol{\mu }$ is expressed in terms of the Gaussian distribution with updated parameters ($\hat{\boldsymbol{m}}, \hat{\boldsymbol{\nu }}$) as

51 $$\begin{aligned} p(\boldsymbol{\mu }|\boldsymbol{\lambda }, \boldsymbol{S}, \boldsymbol{\pi }, \boldsymbol{A}, \boldsymbol{I}) = \prod _{k=1}^K \mathscr {N}(\mu _k|\hat{m}_k,(\hat{\nu }_k \lambda _k)^{-1}). \end{aligned}$$

**Posterior distribution for **$\boldsymbol{\lambda }$**:**

We next derive the posterior distribution of the precision parameter $\boldsymbol{\lambda }=(\lambda _1, \lambda _2, \cdots , \lambda _K)$ with the determined $p(\boldsymbol{\mu }|\boldsymbol{\lambda }, \boldsymbol{S}, \boldsymbol{\pi }, \boldsymbol{A}, \boldsymbol{I})$ in Eq. (51): From Eq. (45), it is written as

52 $$\begin{aligned} p(\boldsymbol{\lambda }|\boldsymbol{S}, \boldsymbol{\pi }, \boldsymbol{A}, \boldsymbol{I}) = \frac{p(\boldsymbol{\mu }, \boldsymbol{\lambda }|\boldsymbol{S}, \boldsymbol{\pi }, \boldsymbol{A}, \boldsymbol{I})}{p(\boldsymbol{\mu }|\boldsymbol{\lambda }, \boldsymbol{S}, \boldsymbol{\pi }, \boldsymbol{A}, \boldsymbol{I})} \propto \frac{p(\boldsymbol{\mu }, \boldsymbol{\lambda }, \boldsymbol{S}, \boldsymbol{\pi }, \boldsymbol{A}, \boldsymbol{I})}{p(\boldsymbol{\mu }|\boldsymbol{\lambda }, \boldsymbol{S}, \boldsymbol{\pi }, \boldsymbol{A}, \boldsymbol{I})} \propto \frac{p(\boldsymbol{I}|\boldsymbol{S}, \boldsymbol{\mu }, \boldsymbol{\lambda }) p(\boldsymbol{\mu }, \boldsymbol{\lambda })}{p(\boldsymbol{\mu }|\boldsymbol{\lambda }, \boldsymbol{S}, \boldsymbol{\pi }, \boldsymbol{A}, \boldsymbol{I})}. \end{aligned}$$

By taking the logarithm of the above equation,

53 $$\begin{aligned} & \ln p(\boldsymbol{\lambda }|\boldsymbol{S}, \boldsymbol{\pi }, \boldsymbol{A},\boldsymbol{I}) \nonumber \\= & \ln \frac{p(\boldsymbol{I}|\boldsymbol{S}, \boldsymbol{\mu }, \boldsymbol{\lambda })p(\boldsymbol{\mu }, \boldsymbol{\lambda })}{p(\boldsymbol{\mu }|\boldsymbol{\lambda }, \boldsymbol{S}, \boldsymbol{\pi }, \boldsymbol{A}, \boldsymbol{I})} + \text {const.} \nonumber \\= & \ln p(\boldsymbol{I}|\boldsymbol{S}, \boldsymbol{\mu }, \boldsymbol{\lambda })p(\boldsymbol{\mu }, \boldsymbol{\lambda }) - \ln p(\boldsymbol{\mu }|\boldsymbol{\lambda }, \boldsymbol{S}, \boldsymbol{\pi }, \boldsymbol{A}, \boldsymbol{I}) + \text {const.} \nonumber \\= & \ln \left( \prod _{n=1}^N \prod _{k=1}^K \mathscr {N}(I_n|\mu _k,\lambda _k^{-1})^{s_{kn}}\right) \left( \prod _{k=1}^K \mathscr {N}\textrm{G}(\mu _{k},\lambda _{k}|m,\nu , a,b) \right) - \ln \left( \prod _{k=1}^K \mathscr {N}(\mu _k|\hat{m}_k,(\hat{\nu }_k \lambda _k)^{-1}) \right) + \text {const.} \nonumber \\= & \sum _{k=1}^K \Biggl ( \sum _{n=1}^N s_{kn} \left( -\frac{\lambda _k}{2}(I_n-\mu _k)^2+\frac{1}{2}\ln {\lambda _k}-\frac{1}{2}\ln {2\pi } \right) + \left( -\frac{\nu \lambda _{k}}{2}(\mu _{k}-m)^2+\frac{1}{2}\ln {\nu }+\frac{1}{2}\ln {\lambda _k}-\frac{1}{2}\ln {2\pi } \right) \nonumber \\ & + \left( (a-1) \ln \lambda _{k}-b\lambda _{k} + \ln C_G(a,b) \right) - \left( \frac{\hat{\nu }_k \lambda _k}{2} (\mu _k-\hat{m}_k)^2+\frac{1}{2}\ln {\hat{\nu }_k}+\frac{1}{2}\ln {\lambda _k}-\frac{1}{2}\ln {2\pi } \right) \Biggr ) + \text {const.} \nonumber \\= & \sum _{k=1}^K \left( (\hat{a}_k-1) \ln \lambda _{k}-\hat{b}_k\lambda _{k} \right) + \text {const.}\, \end{aligned}$$

where $\hat{a}_k$ and $\hat{b}_k$ are given as

54 $$\begin{aligned} \hat{a}_k= \frac{1}{2} \sum _{n=1}^N s_{kn}+a \end{aligned}$$

and

55 $$\begin{aligned} \hat{b}_k=\frac{1}{2} \left( \sum _{n=1}^N s_{kn} I_n^2+\nu m^2 -\hat{\nu }_k \hat{m}_k^2 \right) +b, \end{aligned}$$

respectively. Thus, from Eq. (53), the resulting posterior distribution for $\boldsymbol{\lambda }$ is expressed in terms of the Gamma distribution with updated parameters $(\hat{\boldsymbol{a}}, \hat{\boldsymbol{b}})$ as

56 $$\begin{aligned} p(\boldsymbol{\lambda }|\boldsymbol{S}, \boldsymbol{\pi }, \boldsymbol{A},\boldsymbol{I}) = \prod _{k=1}^K \left( C_G(\hat{a}_k,\hat{b}_k)\lambda _k^{\hat{a}_k-1}e^{-\hat{b}_k\lambda _k}\right) . \end{aligned}$$

### Posterior distribution of the transition matrix $\boldsymbol{A}$

The posterior distribution of the transition matrix $\boldsymbol{A}$ is proportional to the product of the $p(\boldsymbol{S}|\boldsymbol{\pi },\boldsymbol{A})$ and $p(\boldsymbol{A})$ as

57 $$\begin{aligned} p(\boldsymbol{A}|\boldsymbol{S},\boldsymbol{\mu },\boldsymbol{\lambda },\boldsymbol{\pi },\boldsymbol{I}) = \frac{p(\boldsymbol{A},\boldsymbol{S},\boldsymbol{\mu },\boldsymbol{\lambda },\boldsymbol{\pi },\boldsymbol{I})}{p(\boldsymbol{S},\boldsymbol{\mu },\boldsymbol{\lambda },\boldsymbol{\pi },\boldsymbol{I})} \propto p(\boldsymbol{A},\boldsymbol{S},\boldsymbol{\mu },\boldsymbol{\lambda },\boldsymbol{\pi },\boldsymbol{I}) \propto p(\boldsymbol{S}|\boldsymbol{\pi },\boldsymbol{A})p(\boldsymbol{A}). \end{aligned}$$

By taking the logarithm of the above equation and inserting Eq. (7) for $p(\boldsymbol{S}|\boldsymbol{\pi },\boldsymbol{A})$ and Eq. (12) for $p(\boldsymbol{A})$,

58 $$\begin{aligned} & \ln p(\boldsymbol{A}|\boldsymbol{S},\boldsymbol{\mu },\boldsymbol{\lambda },\boldsymbol{\pi },\boldsymbol{I}) \nonumber \\= & \ln p(\boldsymbol{S}|\boldsymbol{\pi },\boldsymbol{A})p(\boldsymbol{A}) + \text {const.} \nonumber \\= & \ln \left( p(\boldsymbol{s}_1|\boldsymbol{\pi }) \prod _{n=2}^N p(\boldsymbol{s}_{n}|\boldsymbol{s}_{n-1}, \boldsymbol{A}) \right) \left( \prod _{k=1}^K p(\boldsymbol{A}_{k})\right) + \text {const.} \nonumber \\= & \ln \left( \prod _{n=2}^N \biggl (\ \prod _{k=1}^K \prod _{\ell =1}^K A_{k \ell }^{s_{kn} s_{\ell n-1}} \biggr )\right) \left( \prod _{k=1}^K \biggl ( C_D(\boldsymbol{\beta }_{k}) \prod _{\ell =1}^K A_{k \ell }^{\beta _{k \ell }-1} \biggr )\right) + \text {const.} \nonumber \\= & \sum _{n=2}^N \sum _{k=1}^K \sum _{\ell =1}^{K} s_{kn} s_{\ell n-1} \ln A_{k \ell } + \sum _{k=1}^K \sum _{\ell =1}^K (\beta _{k \ell }-1) \ln A_{k \ell } + \text {const.} \nonumber \\= & \sum _{\ell =1}^K \left( \sum _{k=1}^K (\hat{\beta }_{k \ell } -1 ) \ln A_{k \ell } \right) + \text {const.} \end{aligned}$$

with

59 $$\begin{aligned} \hat{\beta }_{k \ell } = \sum _{n=2}^N s_{kn} s_{\ell n-1} + \beta _{k \ell }, \end{aligned}$$

where we used for Eq. (9) for $p(\boldsymbol{s}_{n}|\boldsymbol{s}_{n-1}, \boldsymbol{A})$ and Eq. (13) for $p(\boldsymbol{A}_{k})$. Thus, from Eq. (58), the posterior distribution of $\boldsymbol{A}$ is expressed by a Dirichlet distribution with updated parameters {$\boldsymbol{\hat{\beta }}_{k}$} as

60 $$\begin{aligned} p(\boldsymbol{A}|\boldsymbol{S},\boldsymbol{\mu },\boldsymbol{\lambda },\boldsymbol{\pi },\boldsymbol{I}) = \prod _{k=1}^K C_D(\boldsymbol{\hat{\beta }}_{k}) \prod _{\ell =1}^K A_{k \ell }^{\hat{\beta }_{k \ell }-1}. \end{aligned}$$

### Posterior distribution of the initial state distribution $\boldsymbol{\pi }$

The posterior distribution of the initial state distribution $\boldsymbol{\pi }$ is proportional to the product of the $p(\boldsymbol{s}_1|\boldsymbol{\pi })$ and $p(\boldsymbol{\pi })$ as

61 $$\begin{aligned} p(\boldsymbol{\pi }|\boldsymbol{S},\boldsymbol{\mu },\boldsymbol{\lambda },\boldsymbol{A},\boldsymbol{I})= \frac{p(\boldsymbol{\pi },\boldsymbol{S},\boldsymbol{\mu },\boldsymbol{\lambda },\boldsymbol{A},\boldsymbol{I})}{p(\boldsymbol{S},\boldsymbol{\mu },\boldsymbol{\lambda },\boldsymbol{A},\boldsymbol{I})} \propto p(\boldsymbol{\pi },\boldsymbol{S},\boldsymbol{\mu },\boldsymbol{\lambda },\boldsymbol{A},\boldsymbol{I}) \propto p(\boldsymbol{S}|\boldsymbol{\pi },\boldsymbol{A})p(\boldsymbol{\pi }) \propto p(\boldsymbol{s}_1|\boldsymbol{\pi })p(\boldsymbol{\pi }). \end{aligned}$$

Thus,

62 $$\begin{aligned} \ln p(\boldsymbol{\pi }|\boldsymbol{S},\boldsymbol{\mu },\boldsymbol{\lambda },\boldsymbol{A},\boldsymbol{I})= & \ln p(\boldsymbol{s}_1|\boldsymbol{\pi }) + \ln p(\boldsymbol{\pi }) + \text {const.} \nonumber \\= & \ln \left( \prod _{k=1}^K \pi _k^{s_{k1}} \right) + \ln \left( C_D(\boldsymbol{\alpha }) \prod _{k=1}^K \pi _k^{\alpha _k-1} \right) + \text {const.} \nonumber \\= & \ln \left( \prod _{k=1}^K \pi _k^{s_{k1}+\alpha _k-1}\right) + \text {const.}, \end{aligned}$$

where we used Eq. (8) for $p(\boldsymbol{s}_1|\boldsymbol{\pi })$ and Eq. (14) for $p(\boldsymbol{\pi })$. Therefore,

63 $$\begin{aligned} p(\boldsymbol{\pi }|\boldsymbol{S},\boldsymbol{\mu },\boldsymbol{\lambda },\boldsymbol{A},\boldsymbol{I}) = C_D(\boldsymbol{\hat{\alpha }}) \prod _{k=1}^K \pi _k^{\hat{\alpha }_k-1} \end{aligned}$$

with

64 $$\begin{aligned} \hat{\alpha }_k = \alpha _k + s_{k1} \end{aligned}$$

This result indicates that the posterior distribution of $\boldsymbol{\pi }$ is also a Dirichlet distribution with updated parameters $\boldsymbol{\hat{\alpha }} = \boldsymbol{\alpha } + \boldsymbol{s}_1$.

### Simulation conditions

Table 1 summarizes the simulation condition and hyperparameter setting. The number of hidden states *K* was set to 2. The total number of Gibbs sampling iterations was $N_{itr}$ = 1,000, which we confirmed to be sufficient for convergence. In the simulation, photon-count trajectory data is standardized to zero mean and unit variance. For the hyperparameters $(\nu , m, a, b)$ of the prior emission distributions $p(\mu _k, \lambda _k)$ in Eq. (11), we used the values (1, 0, 1, 1). Note that these parameters have no *k*-dependence. The hyperparameter $\boldsymbol{\alpha } = (\alpha _1, \alpha _2)$ for the initial-state probability vector, $p(\boldsymbol{\pi })$ in Eq. (14), was set to (1, 1), corresponding to an unbiased prior. The hyperparameter $\boldsymbol{\beta }$ for the transition matrix, $p(\boldsymbol{A})$ in Eq. (12), was set to an 2$\times$2 all-ones matrix, also indicating an unbiased prior. In fact, in the initial stage of the HMM simulation, the $\boldsymbol{\beta }$ is set to a rather biased form as

65 $$\begin{aligned} \boldsymbol{\beta } = \Biggl ( \begin{array}{cc} 20 N & 1 \\ 1 & 20 N \end{array} \Biggr ) \end{aligned}$$

to stabilize the initial stage simulation, where *N* is the length of time series. After the 10 cycle of the Gibbs sampling, we switched the above matrix to the all-ones matrix.

**Table 1:** Simulation condition and setting of hyperparameter:.

Number of states	*K*	2
Iteration cycle	$N_{itr}$	1,000
Emission parameter	$(\nu ,m, a, b)$ [Eq. (11)]	(1, 0, 1, 1)
Initial hidden state	$\boldsymbol{\alpha }$ [Eq. (14)]	(1, 1)
Transition matrix	$\boldsymbol{\beta }$ [Eq. (12)]	(1, 1; 1, 1)

### Probability density of duration

**Fig. 3:**
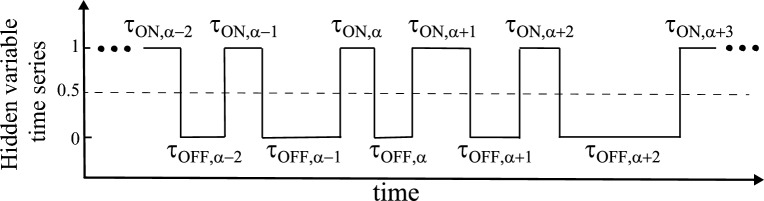
A schematic diagram for the definition of {$\tau _{\textrm{ON}, \alpha }$} and {$\tau _{\textrm{OFF}, \alpha }$}, where these data are collected from the hidden variable time series $\boldsymbol{S}$ in Eq. (3), denoted by solid line, obtained by the HMM simulation. It is noted that $\boldsymbol{S}$ is basically noise-free, so we can safely identify the ON and OFF duration with threshold of 0.5 (dashed line).

Next, we describe how to analyze the hidden-variable time series $\boldsymbol{S}$ in Eq. (3) obtained from the HMM simulation. We first collect ON-duration data {$\tau _{\textrm{ON}, \alpha }$} and OFF-duration data {$\tau _{\textrm{OFF}, \alpha }$} from $\boldsymbol{S}$. Figure 3 describes the definition of {$\tau _{\textrm{ON}, \alpha }$} and {$\tau _{\textrm{OFF}, \alpha }$}, and the collection process of these data. We note that $\boldsymbol{S}$ is basically noise-free, so we can safely identify the ON- and OFF-duration data with using proper threshold of 0.5 (dashed line). With these data, we calculate blinking plots describing probability densities of the ON or OFF duration in the histogram type as

66 $$\begin{aligned} p(\tau _k)=\frac{n_k}{N_e \Delta \tau }, \end{aligned}$$

where $n_k$ is the total number of the events with the duration from $\tau _{k-1}$ to $\tau _k$ as

67 $$\begin{aligned} n_k = \sum _{\alpha }^{N_e} \textbf{1}_{\tau _{k-1} \le \tau _{\alpha } < \tau _{k}}, \end{aligned}$$

and $\boldsymbol{1}_{A}$ is an indicator function for the condition *A*. Also, the duration grid $\tau _k$ is given as $k\Delta \tau$ with $\Delta \tau$ being a grid spacing of duration, and $\tau _{\alpha }$ in Eq. (67) is the $\alpha$-th ON or OFF event duration. $N_{e}$ is the total number of the {$\tau _{\alpha }$} data. We note that $p(\tau _k)$ is normalized as $\sum _{k=1}^{N_{\tau }} p(\tau _k) \Delta \tau =1$ with $N_\tau$ being the total number of the duration grids. These $p(\tau _{\textrm{ON}})$ and $p(\tau _{\textrm{OFF}})$ are called the blinking plot.

The calculation detail is as follows: We analyze the fluorescence trajectories of 39 molecules. The length of the fluorescence trajectories is ranging from 0.46 s to 9.27 s, depending on the molecules. For the time bin $\Delta$ of the trajectory, we consider 0.5 ms, where $\Delta$ represents the time interval to accumulate the photon counts. The total number of the time grids of each trajectory, *N* in Eqs. (2) and (3), depends on the trajectory length and $\Delta$. The total number of the collected {$\tau _{\alpha }$} data, $N_e$ in Eqs. (66) and (67), is 2,557 for the ON case and 2,553 for the OFF case. The grid spacing of duration $\Delta \tau$ in Eq. (66) is set to 1 ms.

### Single molecule measurement for fluorescence trajectory

In the present study, we analyze the fluorescence fluctuation data previously measured for charge-separation and charge-recombination processes in DNA^13,14^. Figures 1 (c) and (d) are schematic chemical structures of the fluorescently labeled DNA (double helix structure) to be analyzed, where A, G, T, and C are the bases that make up DNA, X is the fluorescent molecule ATTO655 as a photosensitizer, and $^{\textrm{Z}}$G is a deazaguanine as a hole trap to observe a charge-separation and charge recombination process in DNA at the single-molecule level. The panel (c) represents a schematic chemical structure for an emitting ON state, where ATTO655 repeats photon-absorption and emission. The panel (d) represents a schematic chemical structure of a not-emitting OFF state; during the charge-separated state, ATTO655 is in the radical anion form and cannot emit, therefore the duration of the OFF state corresponds to the lifetime of the charge-separated state.

Briefly, for the experimental setup, biotin group was introduced at the end of the duplex and was anchored on a glass surface through well-established biotinylated-BSA, streptavidin, biotinylated-DNA chemistry. The single molecule measurement was performed on a custom-made confocal fluorescence microscope consisting of an inverted optical microscope (IX73, Olympus). The output of the laser (OBIS 637 LX, Coherent, 637 nm, 4.6 $\mu$W) was focused in the sample by an objective (UPlanXApo, x60, oil, NA 1.42, Olympus), and the detection volume (confocal volume) was regulated by a pinhole (diameter 25 $\mu$m, Thorlabs MPH16). The scattered light was blocked by a band-pass filter (FF01-697/58-25, Semrock), and the emitted photons from ATTO655 were detected by an avalanche photodiode (SPCM-AQRH-14, Perkin-Elmer). A counting board (SPC-130EMN, Becker & Hickl GmbH) was used to count the output, and real-time monitoring of fluorescence intensity fluctuations has been achieved using the Spcm64 system. (Becker & Hickl GmbH). The time resolution of this device on the photon counting is 80 ns.

**Fig. 4:**
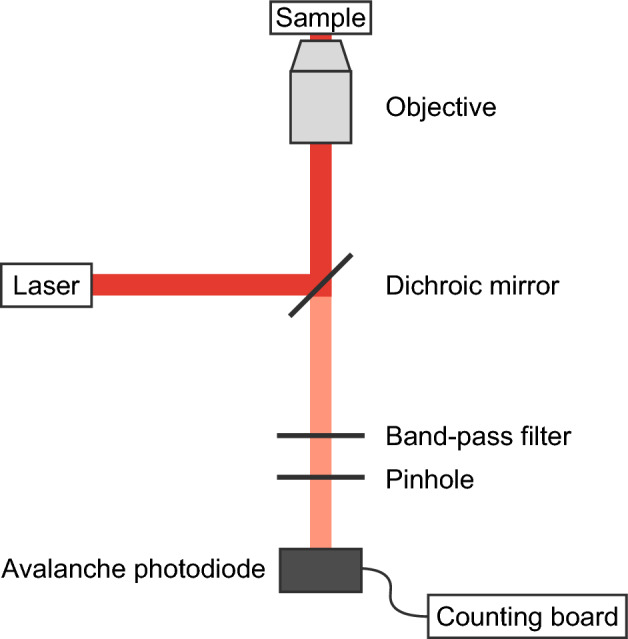
Schematic illustration of the optical setup used for single-molecule fluorescence measurements. The excitation laser is directed into the objective through a dichroic mirror and focused onto the sample. Fluorescence photons emitted from the molecule are preferentially radiated toward the glass side and are collected by the same objective. The collected fluorescence is separated from the excitation light by optical filters and detected by an avalanche photodiode connected to a photon-counting board.

Figure 4shows a schematic of the optical setup used in this study. The excitation laser is introduced into the objective via a dichroic mirror and focused onto fluorescently labeled DNA molecules placed near the glass-water interface (sample). The same objective is used both for delivering the excitation light and for collecting the emitted fluorescence. Due to the proximity to the interface, the emission pattern is biased toward the glass side, allowing efficient collection of fluorescence photons^51^, which are subsequently detected by an avalanche photodiode.

## Results and discussions

### Analysis for fluorescence trajectory

**Fig. 5:**
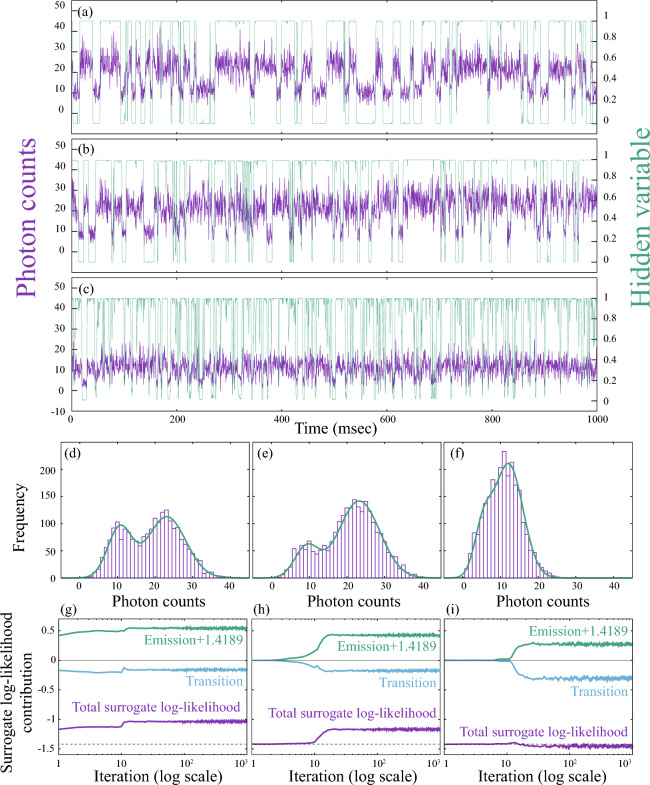
(**a**–**c**) Three representative experimental fluorescence photon-count trajectories, $\boldsymbol{I}$ (purple), defined in Eq. (2), and the corresponding hidden-state sequences, $\boldsymbol{S}$ (green), defined in Eq. (3). The photon counts were calculated using fixed-width time bins of $\Delta = 0.5$ ms. (**d**–**f**) Photon-count histograms *h*(*n*) corresponding to the trajectories in panels (**a**–**c**). The model distributions $h_{\textrm{model}}(n)$ calculated from Eq. (68) are shown as green curves. (**g**–**i**) Iteration-step dependence of the surrogate objective of the log-likelihood $\mathscr {L}$ defined in Eq. (22), together with its decomposition into emission and transition contributions. Panels (**g**), (**h**), and (**i**) correspond to the $\mathscr {L}$ values for the data shown in panels (**a**), (**b**), and (**c**), respectively.

Figures 5 (a), (b), and (c) show three representative experimental fluorescence trajectories $\boldsymbol{I}$ defined in Eq. (2), where the photon counts are calculated in fixed-width time bins of $\Delta = 0.5$ ms. The purple lines represent the raw photon-count data, while the overlaid thin green lines indicate the most probable hidden-state sequences $\boldsymbol{S}$ [Eq. (3)] obtained by the HMM simulations. As we move from the panel (a) to (c), the hidden-state trajectories become progressively noisier, suggesting that state identification becomes less reliable.

To investigate the underlying cause of this behavior, we examined the photon-count histogram *h*(*n*), where *n* denotes the photon count per time bin. We show in Figs. 5 (d), (e), and (f) the corresponding histograms for the data in the panels (a), (b), and (c), respectively. The histogram in Fig. 5 (d) shows a clear bimodal structure, which reflects a well-separated ON/OFF signal distribution. In contrast, Fig. 5 (e) displays a broad, shoulder-like shape, indicating partial overlap between ON and OFF states. Figure 5(f) exhibits a single, nearly symmetric peak, suggesting that the emission levels from the ON and OFF states become difficult to distinguish.

These results indicate that the emission distribution shape *h*(*n*) plays a critical role in determining the success of state classification. When *h*(*n*) is clearly bimodal, the HMM analysis yields well-defined state sequences. However, when the emission distribution lacks clear separation, the hidden-state trajectories become ambiguous, even when applying the same inference algorithm. This highlights the importance of emission distinguishability in model-based trajectory analysis.

We note that, in case where *h*(*n*) exhibits a single-peak structure, the emission parameters $(\mu _k, \lambda _k)$ and the statistically-averaged ON/OFF event numbers can still be stably estimated from the HMM simulation. To demonstrate this point, we calculated the model distribution for $h_{model}(n)$ as

68 $$\begin{aligned} h_{model}(n) = N_{eve} \left( w_{\textrm{ON}} \sqrt{\frac{\lambda _{\textrm{ON}}}{2\pi }} \exp \left( -\frac{\lambda _{\textrm{ON}}}{2} (n-\mu _{\textrm{ON}})^2 \right) + w_{\textrm{OFF}} \sqrt{\frac{\lambda _{\textrm{OFF}}}{2\pi }} \exp \left( -\frac{\lambda _{\textrm{OFF}}}{2} (n-\mu _{\textrm{OFF}})^2 \right) \right) , \end{aligned}$$

where $w_{\textrm{ON}}$ and $w_{\textrm{OFF}}$ are weights of the ON and OFF emission distributions, defined as

69 $$\begin{aligned} w_{\textrm{ON}} = \frac{\langle N_{\textrm{ON}} \rangle }{N} \quad \text {and} \quad w_{\textrm{OFF}} = \frac{\langle N_{\textrm{OFF}} \rangle }{N}. \end{aligned}$$

Here, $\langle N_{\textrm{ON}} \rangle$ and $\langle N_{\textrm{OFF}} \rangle$ denote the numbers of ON and OFF events in the hidden-state sequence $\boldsymbol{S}$, respectively, averaged statistically over the Gibbs sampling. The quantity $N_{\textrm{eve}}$ in Eq. (68) represents the total number of photon-counting events, which is estimated as $N_{\textrm{eve}} = \sum _{n} h(n)$ from the original histogram *h*(*n*). The resulting $h_{model}(n)$ is overlaid in Figs. 5 (d)-(f) with green curves. We observe good agreements between the original *h*(*n*) and the model $h_{model}(n)$, indicating that static properties such as photon count distributions can be well reproduced by the HMM simulation. On the other hand, in case where the ON and OFF emission distributions strongly overlap [Fig. 5 (f)], the posterior probability for the hidden-state sequence becomes ambiguous at each time point [Fig. 5 (c)], resulting in noisy or rapidly fluctuating state sequences. This behavior does not indicate a failure of the HMM, but rather reflects the fundamental identifiability limit under the given signal-to-noise conditions.

The goodness of fit between the HMM and the observed data is evaluated by monitoring the surrogate objective of the log-likelihood $\mathscr {L}$ in Eq. (22), which can be expressed, omitting the initial-state contribution, as

70 $$\begin{aligned} \mathscr {L} \sim \frac{1}{2} \sum _{k=1}^{K} w_{k}^{(i)} \ln \lambda _k^{(i)} -\frac{1}{2}\left( 1+\ln (2\pi ) \right) +\sum _{\ell =1}^{K} w_{\ell }^{(i)} \sum _{k=1}^{K} A_{k\ell }^{(i)} \ln A_{k\ell }^{(i)}. \end{aligned}$$

The quantity

71 $$\begin{aligned} w_{k}^{(i)} = \frac{1}{N} \sum _{n=1}^{N} s_{kn}^{(i)} \end{aligned}$$

represents the occurrence frequency of state *k* in the entire time series. In Eq. (70), we assume that $\boldsymbol{w}$ does not depend on the time step. Note also that the observation data $\{I_n\}$ are standardized to zero mean and unit variance in the simulation, and that the superscript (*i*) denotes the iteration step in the Gibbs sampling. The first and second terms in Eq. (70) constitute the emission contribution, with the second term serving as a constant baseline of $-1.4189$. The first term increases positively as the precision $\lambda _{k}^{(i)}$ becomes larger, thereby contributing to a higher likelihood. The third term represents the transition contribution, which is negative and approaches zero as the state transitions become fewer, again leading to a higher overall likelihood.

Figures 5 (g)–(i) show the iteration-step dependence of the surrogate objective of the log-likelihood $\mathscr {L}$ (purple lines) in Eq. (22) and its decompositions, where the contribution from the initial state is omitted. Panels (g), (h), and (i) correspond to the values of $\mathscr {L}$ for the data shown in Figs. 5 (a), (b), and (c), respectively. From the figure, it is evident that the goodness of fit becomes higher for the bimodal histogram, while it becomes lower for the unimodal case. In particular, for panel (i), $\mathscr {L}$ is smaller than the baseline value of $-1.4189$ (dotted line). We also observe a rapid convergence behavior in the HMM simulation: $\mathscr {L}$ fluctuates around a constant value after approximately 20–30 iteration steps.

From this perspective, we selected 39 reliable trajectories out of the 40 experimental datasets; in fact, the dataset shown in Fig. 5(c) was excluded from the analysis. The 39 trajectories were then subjected to further blinking-state analysis in the main text. For completeness, we provide the full results — including the inferred hidden-state sequences of the fluorescence trajectories, the emission histograms, and the iteration-step dependence of the surrogate objective $\mathscr {L}$ — for all 40 experimental datasets in the Supplemental Information, as well as benchmark tests for the Gaussian emission HMM employed in the present study.

### Probability density of duration

**Fig. 6:**
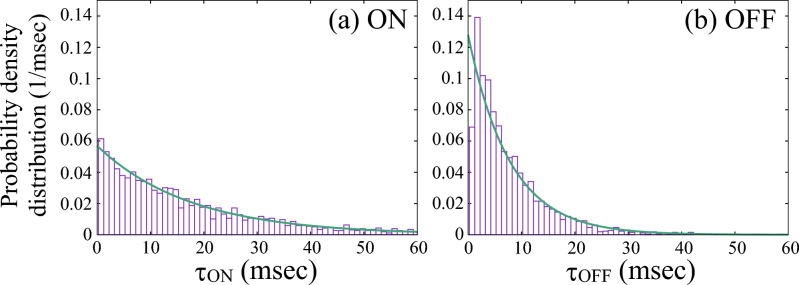
Calculated probability density distribution for (**a**) ON- and (**b**) OFF-state duration. The fitting curves (solid green curves) are single exponential functions of the form $(1/s)\exp (-\tau /s)$ with $s = 17.56$ ms for the ON state and $s = 7.82$ ms for the OFF state.

Figure 6 displays the probability density distributions $p(\tau _k)$ in Eq. (66) for the ON (a) and OFF (b) durations. The purple bars represent normalized histograms of dwell times with unit area, and should therefore be interpreted as empirical probability density functions. We fitted the following exponential probability density function

72 $$\begin{aligned} p(\tau )=\frac{1}{s}\exp \biggl ( - \frac{\tau }{s} \biggr ) \end{aligned}$$

to the dwell time distributions, where *s* is the characteristic decay constant. The fitted values were $s = 17.56$ ms for the ON durations and $s = 7.82$ ms for the OFF durations. The fitting curves are described by green.

The relaxation time associated with the ON$\rightarrow$OFF transition is estimated to be 17.6 ms. Given that the intrinsic fluorescence lifetime is typically in the range of $10^{-9}$ to $10^{-6}$ s, this ON-state duration is remarkably long. This indicates that, once in the ON state, the molecule undergoes many excitation–emission cycles before switching OFF. The detected photon count per 0.5-ms time bin is approximately 20, as estimated from the averaged mean ON-state intensity (Gaussian mean parameter) over 39 datasets. This corresponds to an average detected inter-photon interval of roughly 25 $\mu$s. However, a quantitative estimate of the absolute number of photons emitted by a molecule is highly nontrivial in practice. The detected photon counts depend sensitively on experimental factors such as the numerical aperture of the objective lens, the transmission efficiency of the band-pass filters, the pinhole size in front of the detector, and the precise position and orientation of the fluorescent molecule within the focal volume. Therefore, the above estimate should be regarded as an order-of-magnitude indication rather than a precise determination of the intrinsic emission cycle. Since this timescale is still much longer than the intrinsic optical excitation–emission timescale of individual molecules, which is on the order of nanoseconds, the ON state can be better described not as a continuously fluorescing state, but as a quasi-steady state in which photon emission events occur intermittently on a relatively slow timescale.

The relaxation time associated with the OFF$\rightarrow$ON transition was estimated to be 7.8 ms. This value is consistent with previous reports based on autocorrelation analyses^13,14^, which identified a characteristic timescale of approximately 5 ms. In our system, the OFF state is attributed to a charge-separated configuration^13,14^, during which the molecule cannot absorb or emit light. This interpretation is supported by the near-complete disappearance of photon counts during OFF periods. Therefore, the 7.8 ms OFF-state lifetime can be regarded as the timescale of charge recombination, although this value is expected to vary depending on the local DNA environment. The OFF-state duration of about 8 ms corresponds to a relatively long-lived charge-separated state compared with typical fluorescent dyes, suggesting that charge recombination in the present DNA-ATTO655 system is strongly suppressed.

To verify the accuracy of the relaxation times, we also estimated them from the transition matrix $\boldsymbol{A}$ obtained through the HMM simulations as

73 $$\begin{aligned} \tau _{\textrm{ON}} = \frac{\Delta }{1 - \bar{A}_{\textrm{ON,ON}}} \quad \text {and} \quad \tau _{\textrm{OFF}} = \frac{\Delta }{1 - \bar{A}_{\textrm{OFF,OFF}}}, \end{aligned}$$

where $\Delta$ is the time bin of 0.5 ms, and $\bar{A}_{\textrm{ON,ON}}$ and $\bar{A}_{\textrm{OFF,OFF}}$ are the transition-matrix elements averaged statistically over the Gibbs sampling, representing the average ON$\rightarrow$ON and OFF$\rightarrow$OFF transition probabilities, respectively. The estimated relaxation times were $\tau _{\textrm{ON}} = 19.30 \pm 8.06$ ms and $\tau _{\textrm{OFF}} = 6.98 \pm 2.15$ ms, in reasonable agreement with those obtained from exponential fits to the blinking plots ($\tau _{\textrm{ON}} \sim 17.6$ ms and $\tau _{\textrm{OFF}} \sim 7.8$ ms), confirming the consistency between the two analyses.

### Time-bin dependence of photon count histogram

**Fig. 7:**
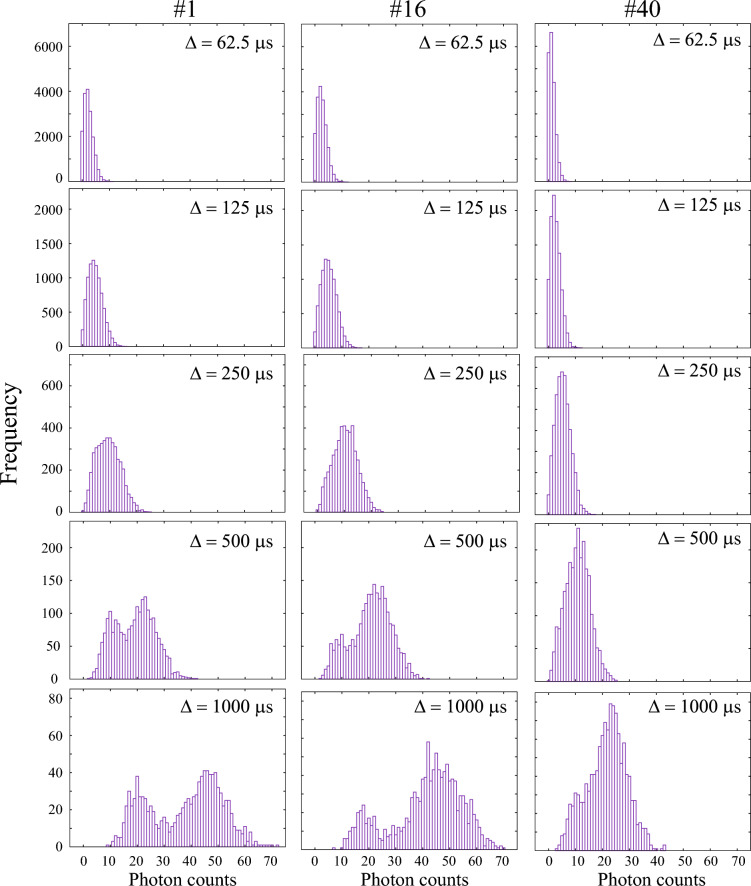
Time-bin-width ($\Delta$) dependence of the photon-count histogram *h*(*n*) for datasets #1, #16, and #40. Fluorescence photon-count trajectories were reconstructed using different $\Delta$ values, and the corresponding histograms *h*(*n*) were calculated. The results are displayed from top to bottom in order of increasing $\Delta$. Note that the total number of detected photons, $\sum _n n\,h(n)$, remains constant regardless of the choice of $\Delta$.

Here, we discuss the numerical stability of the HMM-based state determination in terms of the photon count histogram. In our analysis, the photon-count distribution is modeled by a Gaussian emission distribution [Eq. (6)]. Deviations of the empirical photon-count histogram from the Gaussian shape significantly reduce the accuracy of state determination. Figure 7 compares the photon-count histograms *h*(*n*) for three datasets (#1, #16, and #40). Photon-count time series were generated by varying the time bin $\Delta$, and histograms were computed from these time series. Panels from top to bottom show the changes in the histograms as $\Delta$ increases. Data #1, #16, and #40 correspond to the left, middle, and right columns, respectively, and these $\Delta = 500~\mu \textrm{s}$ results correspond to Figs. 5(d), (e), and (f). For all datasets, when $\Delta$ = 250 $\mu$s, the histograms exhibit a unimodal shape. Furthermore, when $\Delta$ is small, the histograms are heavily biased toward $n = 0$. Under such conditions, *h*(*n*) strongly deviates from the Gaussian assumption, and the quantitative performance of the HMM can be degraded. As $\Delta$ is increased to 500 $\mu$s, datasets #1 and #16 develop a clear bimodal structure, and when $\Delta$ reaches 1000 $\mu$s, a shoulder-like structure emerges in #40 as well. The bimodal nature of *h*(*n*) is crucial for stable ON/OFF state determination, and in practice, $\Delta$ is usually chosen large enough for this bimodal distribution to appear.

However, because $\Delta$ provides a lower limit on the state duration that can be resolved in the analysis, increasing $\Delta$ inevitably results in the loss of information about short-lived events. Therefore, to obtain an accurate lifetime distribution, $\Delta$ should be kept as small as possible. In practice, reliable evaluation of events with duration $\tau = \Delta$, where a single time step corresponds to the event duration, is difficult. Reliable estimation of state durations is typically possible only for events longer than approximately $\tau \ge 3\Delta$. To summarize,

1. In time series data with time-bin $\Delta$, $\Delta$ itself sets the lower bound for reliable state determination.
2. In practice, the minimum duration $\tau _{\textrm{min}}$ that ensures accurate state identification is approximately $3\Delta$.
3. In addition, for Gaussian HMMs, it is desirable that the emission distribution is bimodal. When the emission distribution is unimodal, state identification becomes unstable, resulting in noisy hidden-state sequences.
4. Although increasing $\Delta$ can induce bimodality in the emission distribution, this comes at the cost of neglecting short-lived components.

### Mean-variance relation of inferred photon counts

**Fig. 8:**
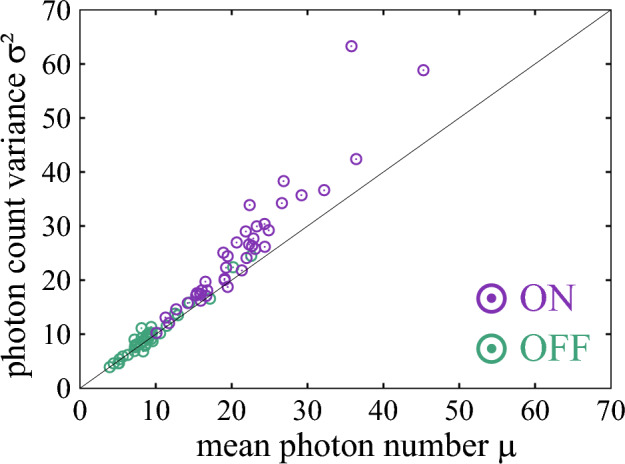
Inferred emission parameters in Eq. (6). Shown are the mean photon count $\mu _k$ and the photon count variance $\sigma _k^2 = \lambda _k^{-1}$ for each molecule. Purple and green circles correspond to the ON and OFF states, respectively. The solid line represents the Poisson relation $\sigma _k^2 = \mu _k$.

Finally, we discuss emission characteristics of individual molecules. For this purpose, we examined the inferred Gaussian emission parameters ($\mu$, $\sigma ^2$) in Eq. (6) with $\sigma ^2=\lambda ^{-1}$ for each molecule separately. Figure 8 shows a $\mu$–$\sigma ^2$ plot for all molecules. The resulting scatter plot shows a clear correlation following the Poisson relation $\sigma ^2 \sim \mu$, which indicates that the detected photons basically follow Poisson statistics even when analyzed through our Gaussian-emission HMM. Although the Gaussian model treats $\mu$ and $\sigma ^2$ as independent free parameters, the data naturally fall on the Poisson-like relation when the time bin is sufficiently large (500 $\mu$s), demonstrating that the Gaussian approximation captures the photon-count statistics well in this regime. At the same time, the plot also shows systematic deviations from the ideal Poisson line, particularly for the ON state. These deviations may indeed reflect molecular heterogeneity, for example variations in the donor–acceptor distance^13,14^. Regarding conformational dynamics of molecules, previous work has shown that the relevant motions (e.g., base flipping and minor tautomeric structures) occur on sub-microsecond to millisecond timescales^13,14^, which are much faster than the blinking dwell times ($\sim$10 ms). Therefore, these fast motions are effectively averaged out in our measurements. The key observation is that the mean ON photon counts vary between molecules (from $\sim$10 to $\sim$40), while the relation $\sigma ^2 \sim \mu$ remains approximately preserved across this range. This suggests that while molecules differ in brightness–likely reflecting variations in optical detection efficiency and/or molecular orientation–the underlying photon-count statistics are dominated by Poisson-type fluctuations rather than by heterogeneity in the blinking kinetics.

## Conclusion

In this study, we analyzed the blinking phenomenon of fluorescently labeled DNA with using HMM. The time series of the inferred hidden variables enabled the quantitative determination of the ON (light-emitting) and OFF (non-emitting) states. Although the fluorescence trajectories are short in the present system (approximately 1 s) and exhibit frequent ON-OFF switching, the HMM analysis worked stably. We performed analyses on fluorescence trajectories with a time bin of 0.5 ms, and found that the probability densities of both the ON and OFF durations are well represented by exponential distributions, with relaxation times of 17.6 ms for the ON state and 7.8 ms for the OFF state. The relatively long OFF period indicates that the charge-separated state in the DNA-ATTO655 system is fairly stable. The detected photon count per 0.5-ms time bin is approximately 20, as estimated from the averaged mean ON-state intensity over the datasets, corresponding to a naive detected inter-photon interval of roughly 25 $\mu$s. While this value is not regarded as an intrinsic emission timescale of molecules, it can still be much longer than the intrinsic optical excitation-emission timescale of individual molecules, which is on the order of the nanoseconds. Achieving a consistent understanding of these distinct timescales is essential for a more complete characterization of the ON-state dynamics. These results provide a physically meaningful picture of the underlying fluorescence dynamics, which remains robust against model bias and time-resolution effects as long as the emission distinguishability is maintained.

We also investigated the relationship between the time bin $\Delta$ introduced in the photon-count time series and the resulting photon-count histogram. Increasing $\Delta$ not only shifts the photon-count histogram toward larger values but can also lead to the emergence of a bimodal structure. For the fluorescent material studied here, a time-bin width of $\Delta = 0.5$ ms was required for this bimodal pattern to appear in many datasets. The bimodal nature of the histogram is crucial for HMMs that employ Gaussian emission distributions, since time series with bimodal emission histograms enable more robust state identification. On the other hand, using a large $\Delta$ artificially excludes the possibility of states with lifetimes shorter than $\Delta$, which can lead to a significant loss of information in the analysis. Since photon-count statistics are inherently Poissonian, one possible approach is to adopt a Poisson distribution as the emission model^46^, which may allow the use of smaller time bins. However, regardless of the emission model, the introduction of $\Delta$ remains a critical step.

Recent approaches have attempted to assign states on a photon-by-photon basis, rather than using time bins. A representative class of such methods is change-point analysis^33,34^, which detects changes in photon arrival rates directly from arrival-time records, thereby avoiding explicit time binning. While the change-point analysis has proven powerful for high signal-to-noise data, it is essentially based on local information around each change point. More recently, photon-by-photon hidden Markov models (H$^2$MM) have been proposed^40,41^, which combine the advantages of arrival-time-based analysis with the global inference framework of HMMs by assigning hidden states directly to individual photon arrival events. Although this method has mainly been applied to FRET analysis, applying it to more fundamental blinking analyses would be highly valuable. In this formulation, however, the transition probabilities generally depend on the inter-photon time intervals, which makes the application of efficient sampling algorithms, such as forward-filtered backward sampling, nontrivial. Designing stable and efficient inference algorithms under these conditions remains an important challenge. In the present work, we therefore focused on a Gaussian HMM formulation with time binning, which allows the use of forward-filtered backward sampling and provides stable global inference for noisy fluorescence blinking trajectories under experimentally relevant conditions. Extending this framework to photon-by-photon analyses is an important direction for future studies.

## Supplementary Information

Supplementary Information.

## Data Availability

The data that support the findings of this study are available from the corresponding author upon reasonable request.
