# Supplementary material for: Hidden Markov model analysis of fluorescence blinking in fluorescently labeled DNA
Source: Sci Rep. 2026 Feb 27;16:11306. doi: 10.1038/s41598-026-40876-x (PMC13048983; doi:10.1038/s41598-026-40876-x)
Supplement: Supplementary file 1 — Supplementary Information. [file 41598_2026_40876_MOESM1_ESM.pdf]

Supplemental Information for Hidden Markov model analysis of fluorescence blinking in fluorescently labeled DNA

Tatsuhiro Furuta[1], Shuya Fan[2], Tadao Takada[3], Yohei Kondo[4], Mamoru Fujitsuka[2], Atsushi Maruyama[5], Kiyohiko Kawai[4,+], and Kazuma Nakamura[1,6,*]

[1] *Graduate School of Engineering, Kyushu Institute of Technology, 1-1 Sensui-cho, Tobata-ku, Kitakyushu, Fukuoka 804-8550, Japan.*
[2] *SANKEN (The Institute of Scientific and Industrial Research), The University of Osaka, Mihogaoka 8-1, Ibaraki, Osaka 567-0047, Japan.*
[3] *Department of Applied Chemistry, Graduate School of Engineering, University of Hyogo, 2167 Shosha, Himeji, Hyogo 671-2280, Japan.*
[4] *Department of Life Science and Technology, Institute of Science Tokyo, Nagatsuta, Midori-ku, Yokohama, Kanagawa 226-8501, Japan.*
[5] *Department of Pharmacy, Tokyo University of Pharmacy and Life Sciences, Horinouchi, Hachioji, 192-0392, Japan.*
[6] *Integrated Research Center for Energy and Environmental Technologies, Kyushu Institute of Technology, 1-1 Sensui-cho, Tobata-ku, Kitakyushu, Fukuoka 804-8550, Japan.*
[*] kazuma@mns.kyutech.ac.jp
[+] kawai.k@life.isct.ac.jpk

Benchmark for Gaussian emission hidden Markov model simulation

Here we present a benchmark test for the Gaussian emission hidden Markov model (HMM) used in this study. The benchmark validation is based on the method presented in Ref.[1]. Computational procedure and analysis are summarized as follows:

(1) Construction of the ground-truth synthetic blinking traces:

The true ON and OFF dwell-time distributions were drawn as exponential distributions with time constants $\tau_{\mathrm{ON}} = 17.6$ ms, $\tau_{\mathrm{OFF}} = 7.8$ ms, and these timescales are based on the analysis results for the experimental time series addressed in the main text. One hundred ON and one hundred OFF dwell times were independently sampled from the exponential distributions and concatenated to generate a ground-truth hidden sequence ("synthetic trace 1"), where the mean intensities of the ON and OFF states were set to $1 + \Delta$ and 1, respectively. The synthetic trace 1 is binned in the time-bin 0.5 ms.

(2) Addition of noise:

Gaussian noise with standard deviation $\sigma = 0.5$ was added to generate realistic noisy observations ("synthetic trace 2"). The signal-to-noise ratio of each synthetic dataset is thus characterized by S/N $= \Delta/\sigma$. We generated 50 independent realizations of synthetic trace 2.

(3) HMM inference and validation:

We applied our HMM analysis to the standardized synthetic trace 2 and obtained the inferred hidden sequence. Using this inferred sequence, we reconstructed the ON and OFF dwell-time histograms from the 50 samples and compared them with the ground-truth exponential distributions set in the step (1). We performed this procedure for a range of S/N values.

(4) Results: S/N threshold for reliable recovery:

Figure S1 (a–c) compares three analysis results for benchmark time series: (a) S/N = 1.0, (b) S/N = 1.5, (c) S/N = 2.0. The upper part of each figure shows resulting "synthetic trace 1" (ground truth, red line) and "synthetic trace 2" (noisy trace, black line), and the lower part compares the ground-truth synthetic trace 1 and the inferred hidden sequence (cyan line). Figure S1(d–f) represent iteration-step dependence of the surrogate objective of the log-likelihood $\mathcal{L}$ defined in Eq. (22) of the main text, used as a convergence and reliability indicator. The $\mathcal{L}$ is shown together with its decomposition into emission and transition contributions. Panels (d), (e), and (f) correspond to the data shown in panels (a), (b), and (c), respectively. Figure S2 displays our calculated blinking plots (purple bars), compared with the ground-truth dwell-time distribution (green curves). Panels (a,b), (c,d), and (e,f) correspond to S/N = 1.0, 1.5, and 2.0, respectively.

From both qualitative inspection of the ON/OFF blinking plots and quantitative evaluation using the surrogate log-likelihood $\mathcal{L}$, we found that a reliable recovery is achieved around $\Delta/\sigma \sim 1.5$; for $\Delta/\sigma > 1.5$, the HMM accurately recovers both the hidden sequence and the underlying exponential dwell-time distributions. We also see that $\mathcal{L}$ is larger than the baseline value of -1.4189, which corresponds to a trivial reference case where both the emission and transition contributions to $\mathcal{L}$ vanish. For $\Delta/\sigma < 1.5$, the HMM tends to produce spurious transitions and the reconstructed dwell-time distributions deviate significantly from the ground truth, particularly in the short duration region. We also see that $\mathcal{L}$ is lower than the baseline of -1.4189.

(5) Implication for the experimental data:

Most of the experimental traces analyzed in this study satisfy S/N above this threshold, because $\mathcal{L}$ is basically larger than the baseline (see "Analysis results for all 40 experimental datasets" in Supporting information), except for the specific trace #40 shown in Figs. 5(c) of the main text, which indeed fell below the baseline and was therefore excluded from the final analysis. We believe that this synthetic-data benchmark provides a quantitative and objective criterion for assessing the reliability of our HMM-based reconstruction of the hidden state sequence $\boldsymbol{S}$.

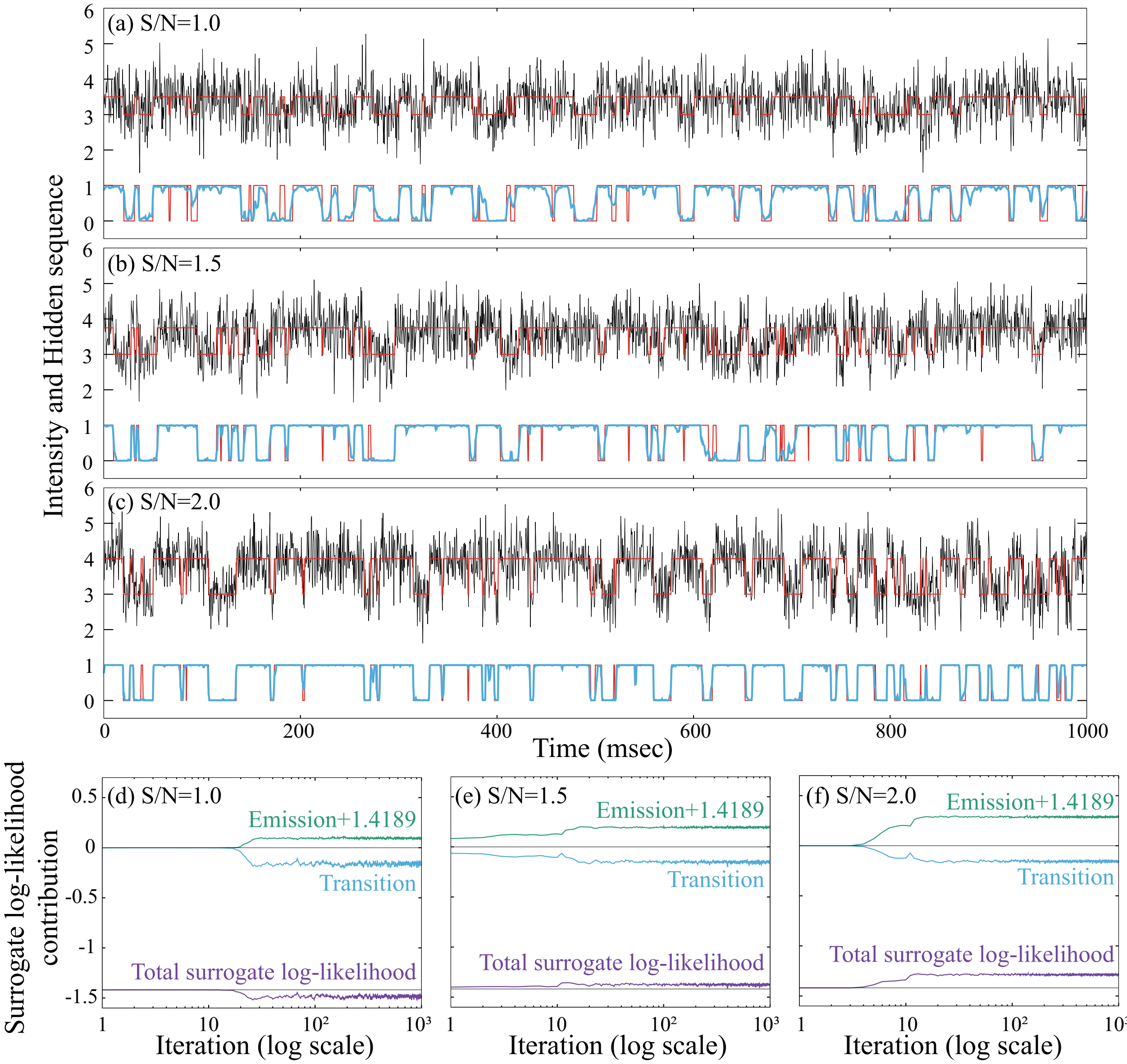

Figure S1: Analysis results for benchmark time series: (a) S/N = 1.0, (b) S/N = 1.5, (c) S/N = 2.0. The upper part of each figure shows resulting “synthetic trace 1” (ground truth, red line) and “synthetic trace 2” (noisy trace, black line), and the lower part shows the synthetic trace 1 and the inferred hidden sequence (cyan line), where the former is scaled to compare with the latter. (d–f) Iteration-step dependence of the surrogate objective of the log-likelihood $\mathcal{L}$ defined in Eq. (22) of the main text, used as a convergence and reliability indicator. The $\mathcal{L}$ is shown together with its decomposition into emission and transition contributions. Panels (d), (e), and (f) correspond to the data shown in panels (a), (b), and (c), respectively.

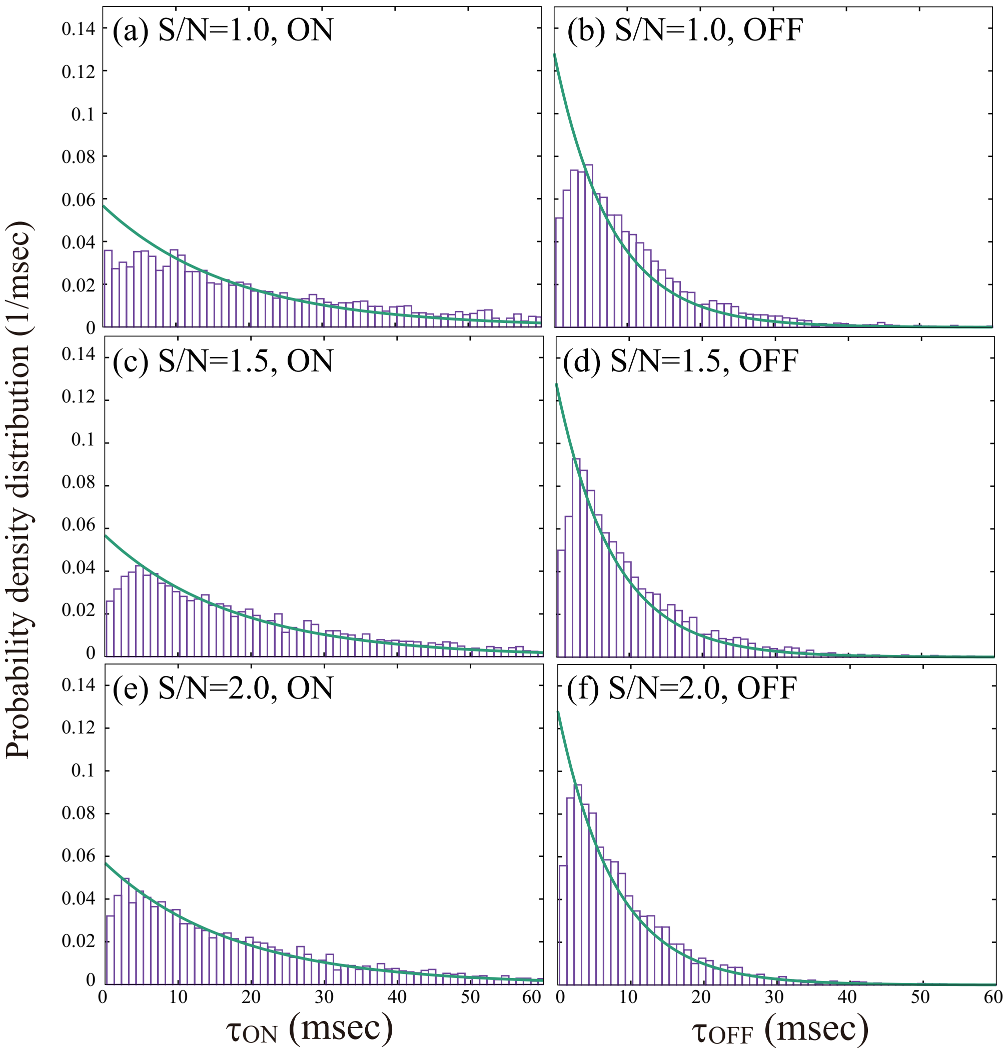

Figure S2: Blinking plots calculated for the benchmark time series. Panels (a,b), (c,d), and (e,f) correspond to signal-to-noise ratios S/N = 1.0, 1.5, and 2.0, respectively. Histograms show the distributions of the inferred ON- and OFF-state dwell times, averaged over 50 independent realizations. Green curves indicate the ground-truth exponential distributions of the form $(1/s)\exp(-\tau/s)$, with $s = 17.56$ ms for the ON state and $s = 7.82$ ms for the OFF state.

Analysis results for all 40 experimental datasets

In this Supplemental Information, we provide the analysis results for all 40 experimental datasets, including the photon-count time-series analysis, the corresponding histograms, and the iteration-step dependence of the surrogate objective $\mathcal{L}$. Data were collected for a total of 40 single-molecule trajectories (`data0001`–`data0040`). On each page, the upper panel shows the photon-count time series, where the solid purple line represents the number of photons detected within each 0.5 ms time bin, and the thin green line indicates the hidden-state sequence inferred from the hidden Markov model simulation. The middle panel displays the histogram of photon counts, while the lower panel presents the surrogate objective $\mathcal{L}$ as a function of the iteration step, decomposed into the emission and transition contributions. Among the 40 datasets, `data0040` was excluded from the calculation of the blinking plot because the inferred hidden-state sequence exhibited substantial noise. Notably, the photon-count histogram of `data0040` shows a unimodal pattern, and its total surrogate objective $\mathcal{L}$ is appreciably lower than those of the other datasets.

References

[1] Tatsuhiro Furuta, Keisuke Hamada, Masaru Oda, and Kazuma Nakamura. Hidden markov model analysis for fluorescent time series of quantum dots. *Phys. Rev. B*, 106:104305, Sep 2022.

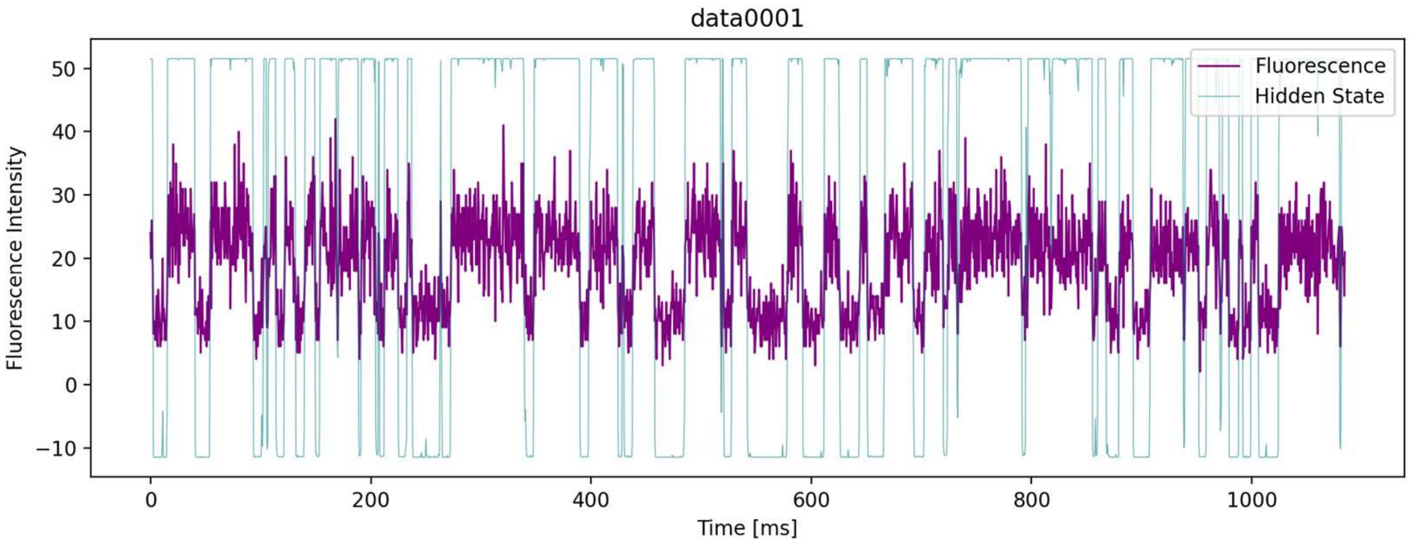
data0001
Fluorescence
Hidden State
Fluorescence Intensity
Time [ms]
50
40
30
20
10
0
−10
0
200
400
600
800
1000

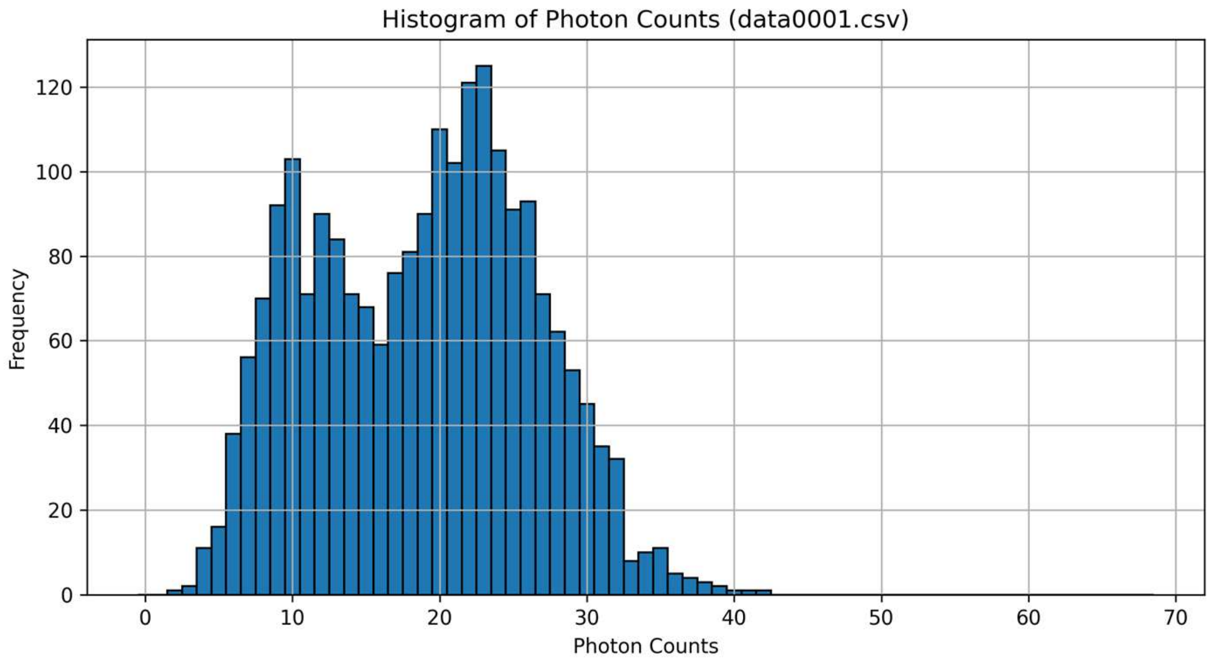
Histogram of Photon Counts (data0001.csv)
Frequency
Photon Counts
120
100
80
60
40
20
0
0
10
20
30
40
50
60
70

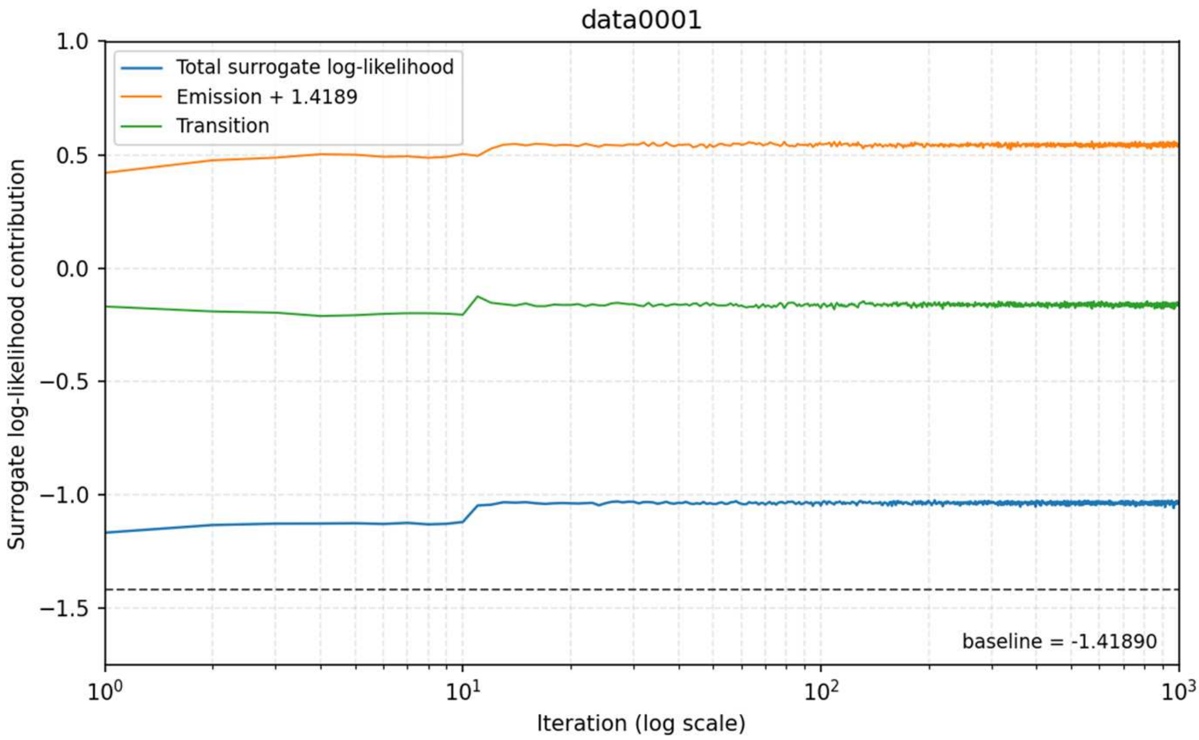
data0001
Total surrogate log-likelihood
Emission + 1.4189
Transition
Surrogate log-likelihood contribution
Iteration (log scale)
baseline = -1.41890
1.0
0.5
0.0
−0.5
−1.0
−1.5
10^0
10^1
10^2
10^3

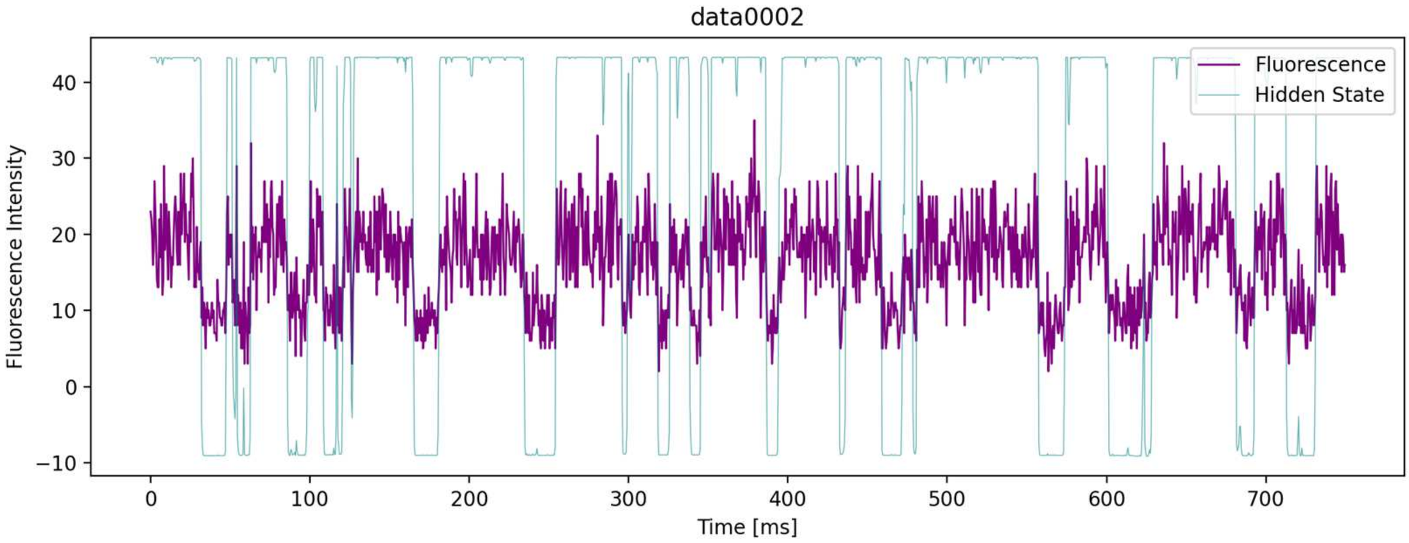
data0002
Fluorescence
Hidden State
Fluorescence Intensity
Time [ms]

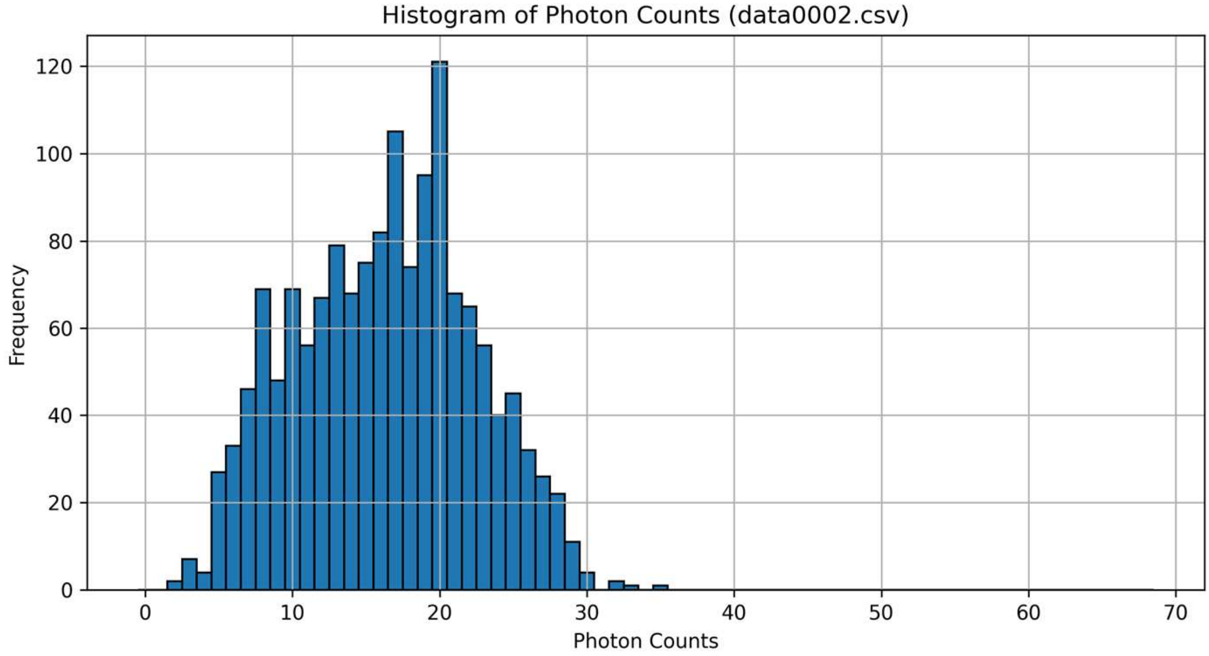
Histogram of Photon Counts (data0002.csv)
Frequency
Photon Counts

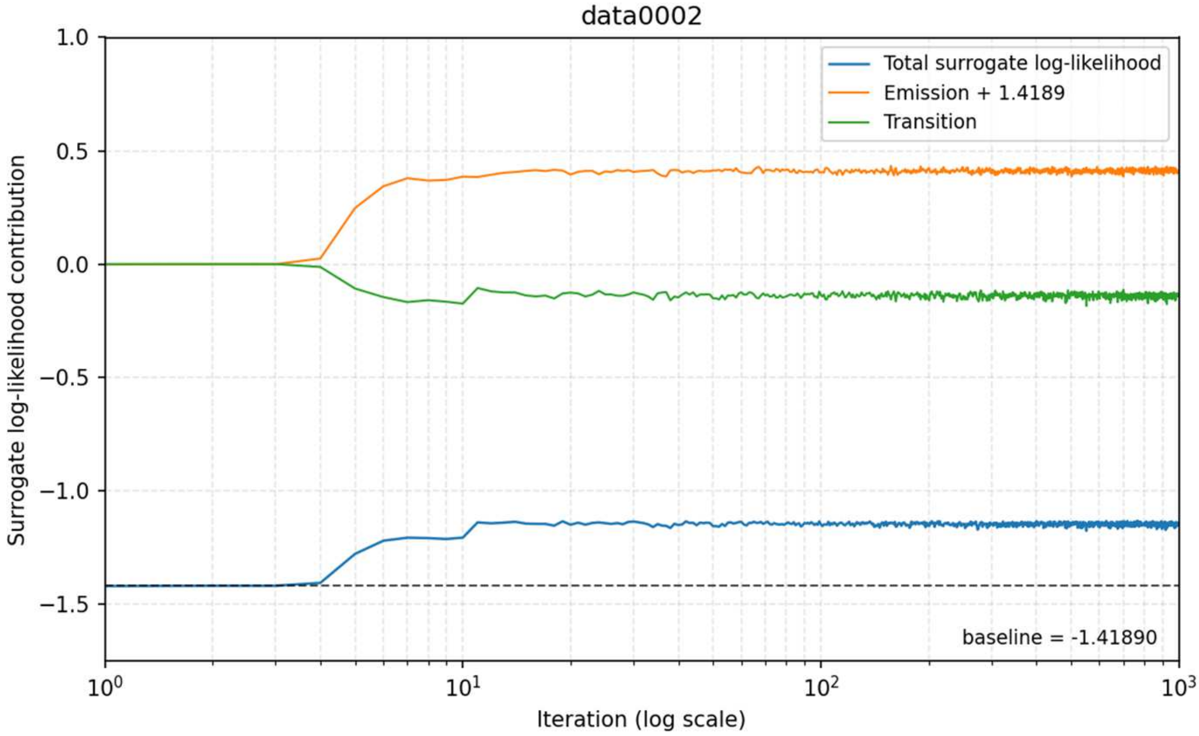
data0002
Total surrogate log-likelihood
Emission + 1.4189
Transition
Surrogate log-likelihood contribution
baseline = -1.41890
Iteration (log scale)

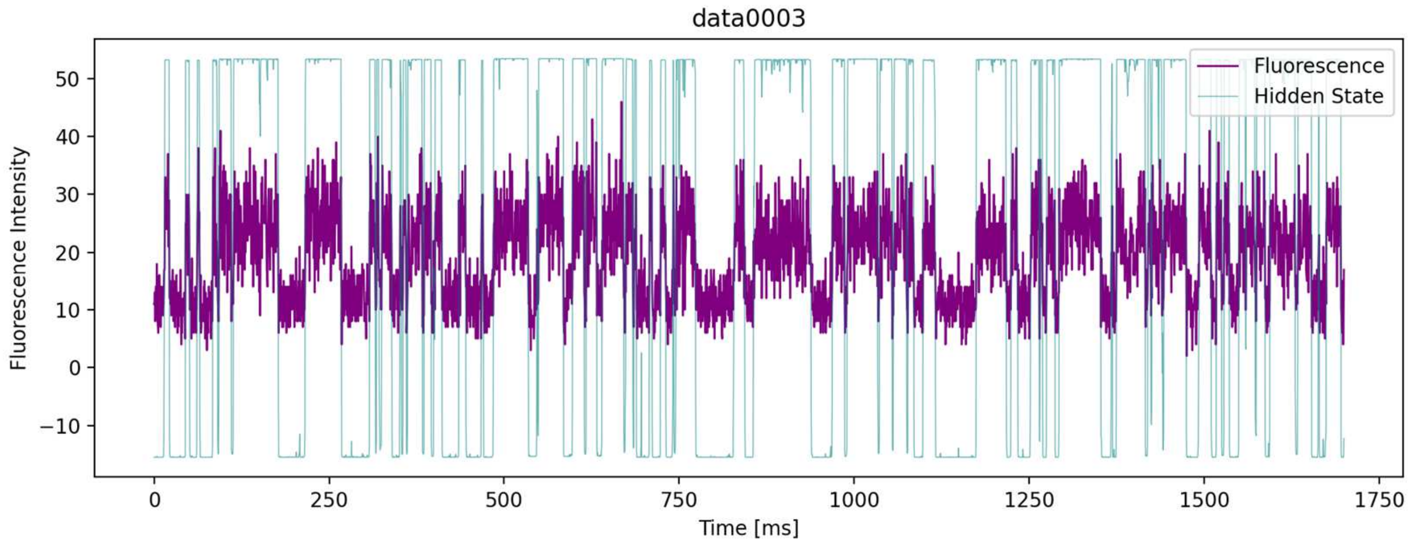
data0003
Fluorescence
Hidden State
Fluorescence Intensity
Time [ms]

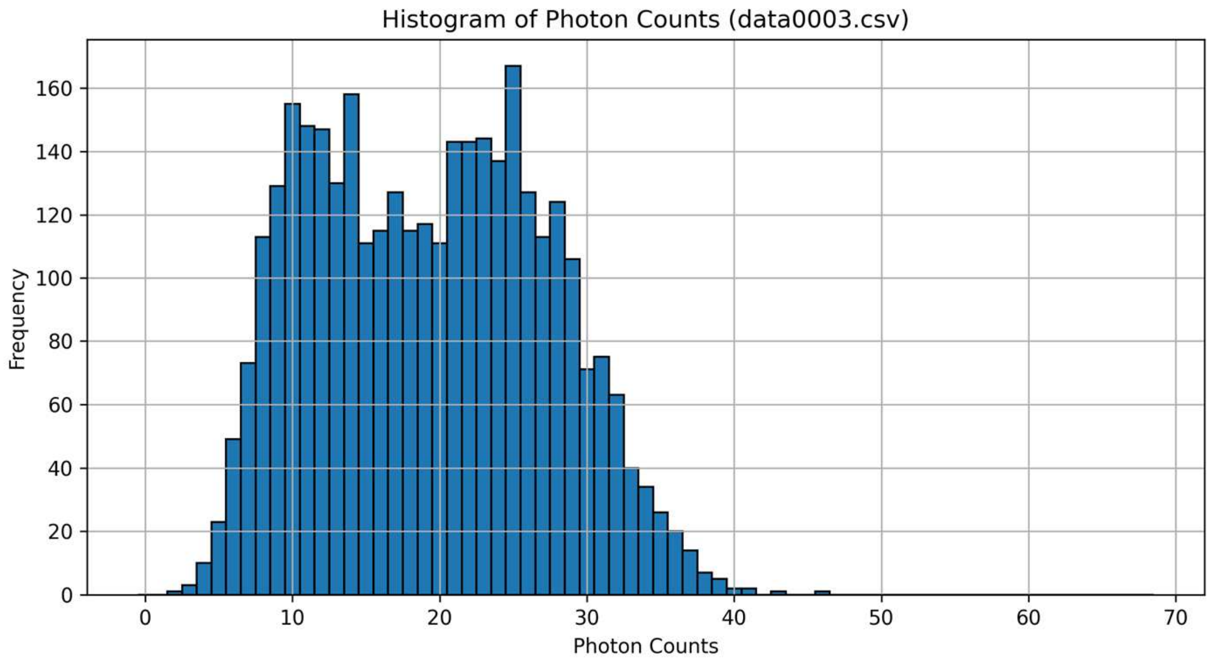
Histogram of Photon Counts (data0003.csv)
Frequency
Photon Counts

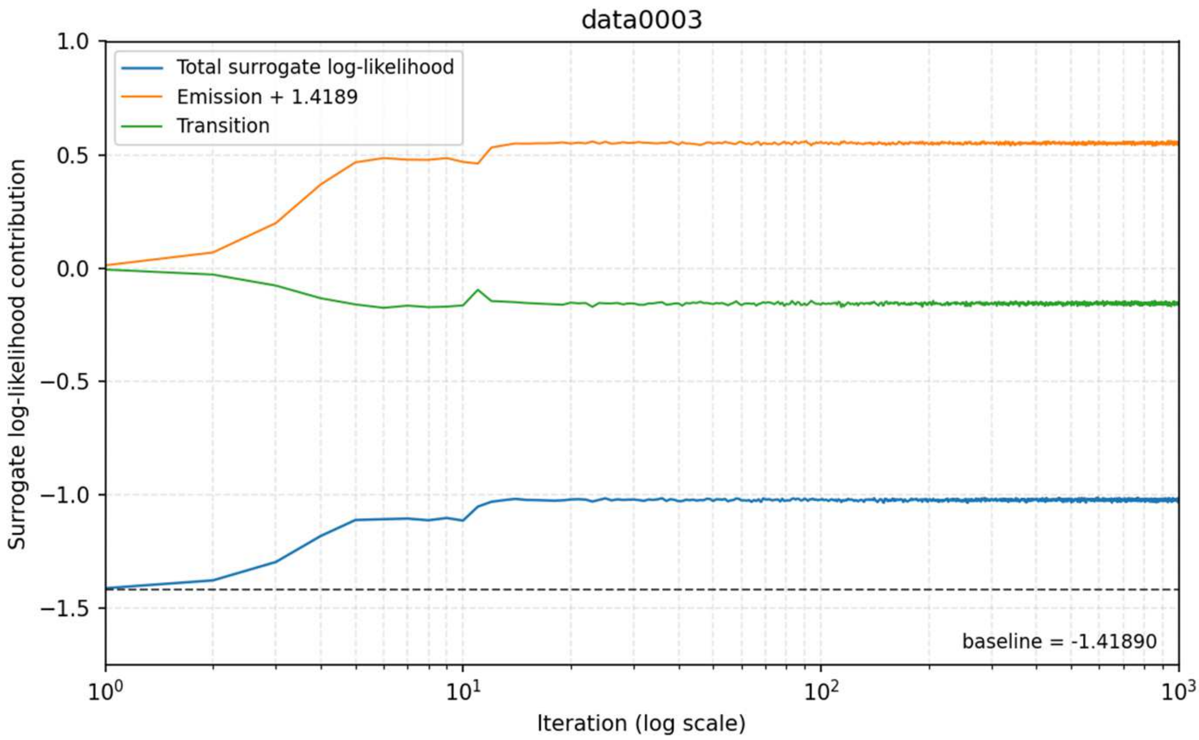
data0003
Total surrogate log-likelihood
Emission + 1.4189
Transition
Surrogate log-likelihood contribution
baseline = -1.41890
Iteration (log scale)

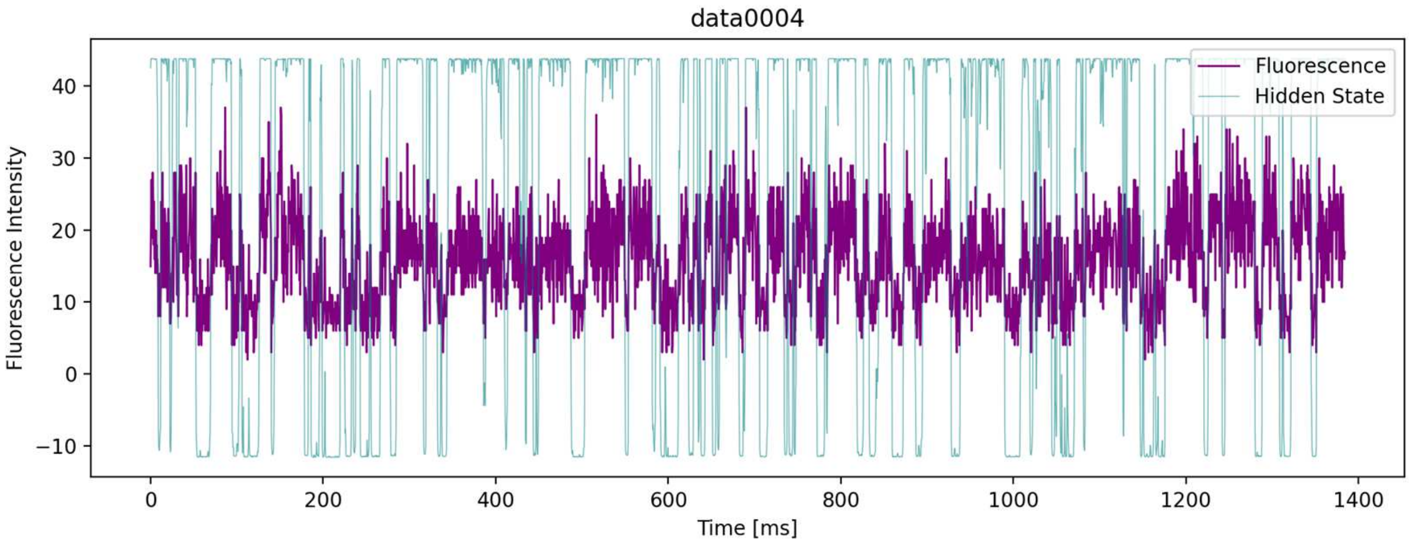
data0004
Fluorescence
Hidden State
Fluorescence Intensity
Time [ms]

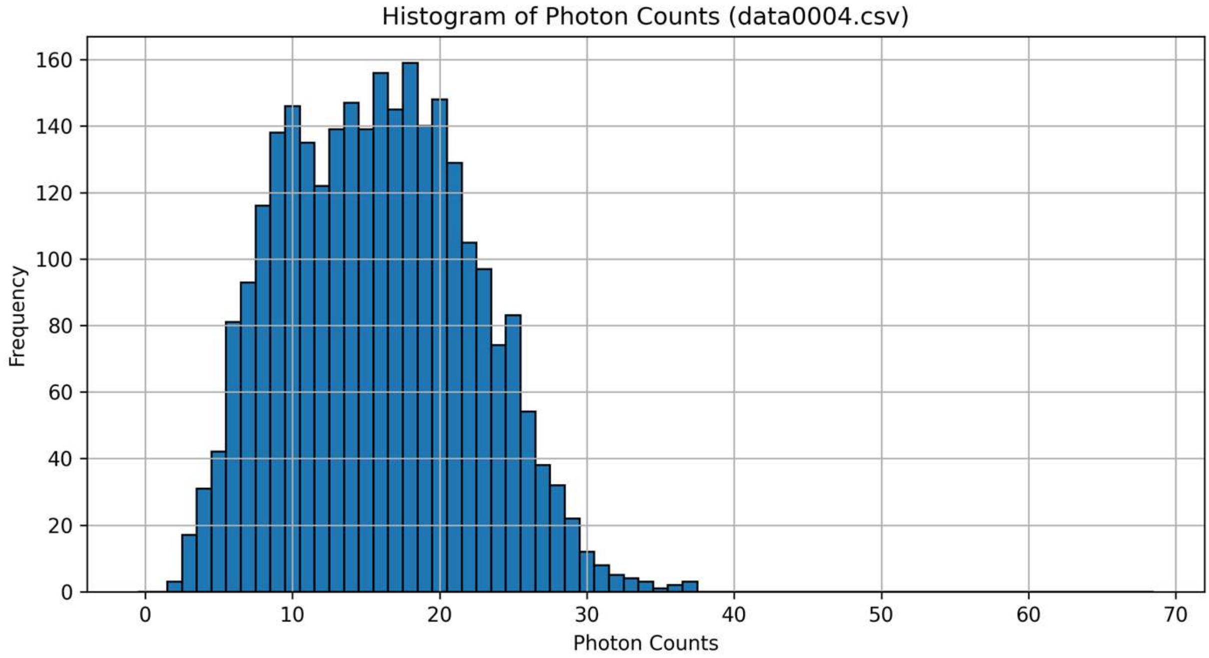
Histogram of Photon Counts (data0004.csv)
Frequency
Photon Counts

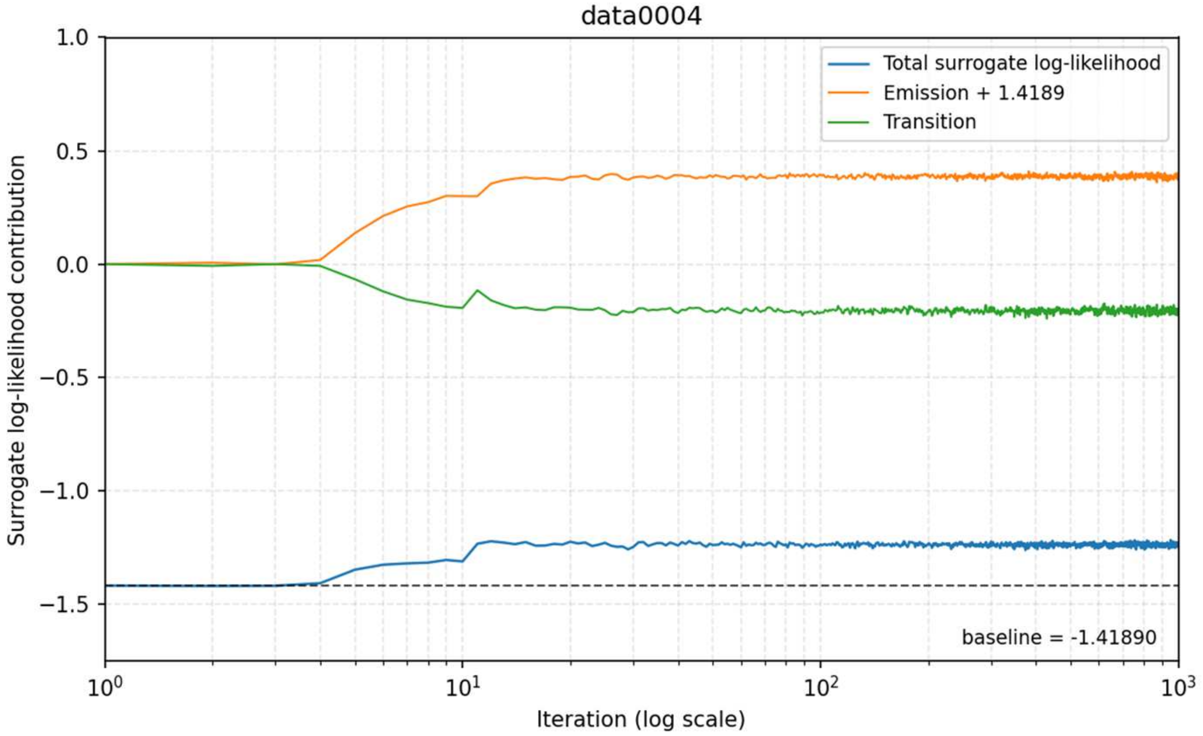
data0004
Total surrogate log-likelihood
Emission + 1.4189
Transition
Surrogate log-likelihood contribution
baseline = -1.41890
Iteration (log scale)

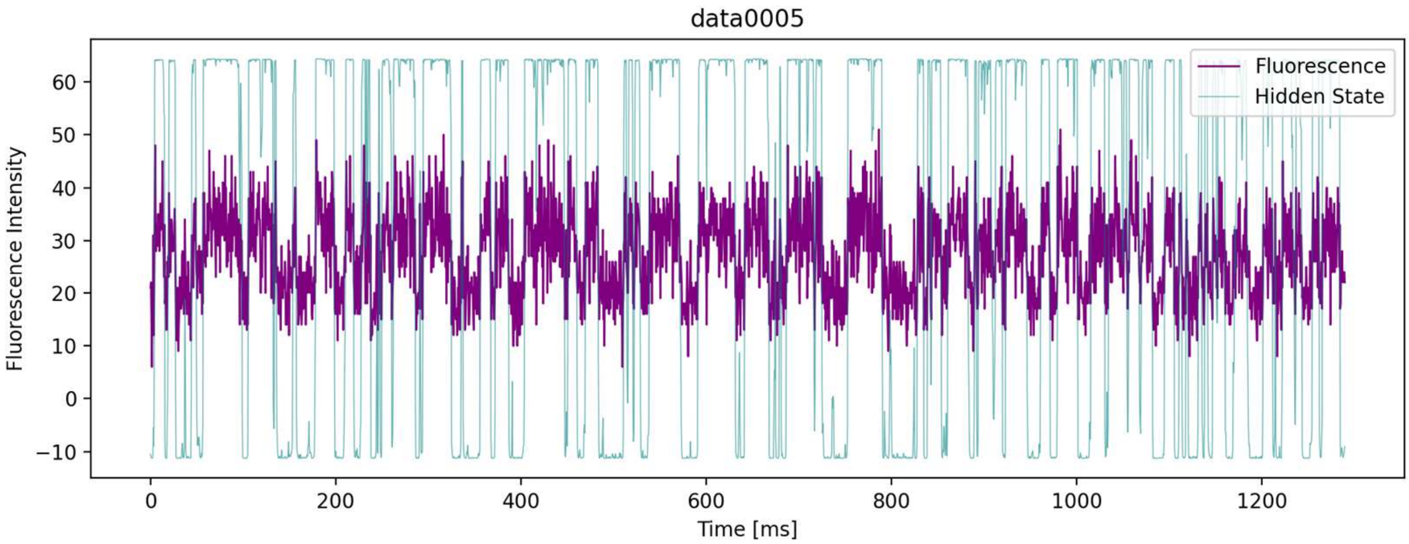
data0005
Fluorescence
Hidden State
Fluorescence Intensity
Time [ms]

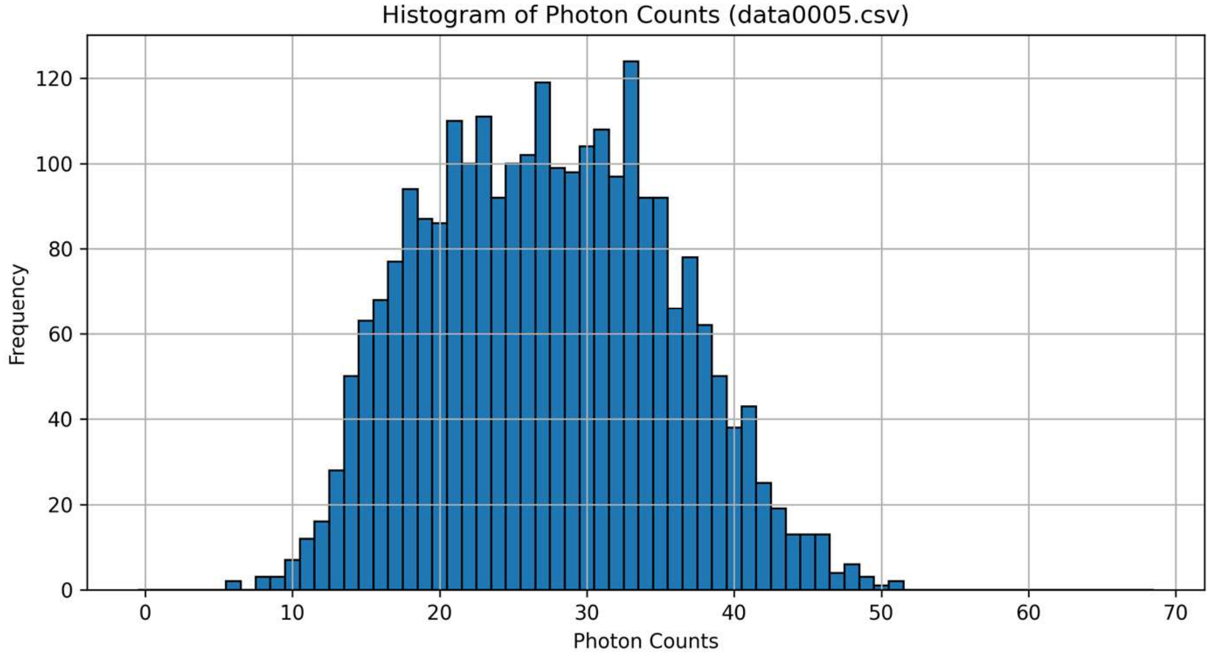
Histogram of Photon Counts (data0005.csv)
Frequency
Photon Counts

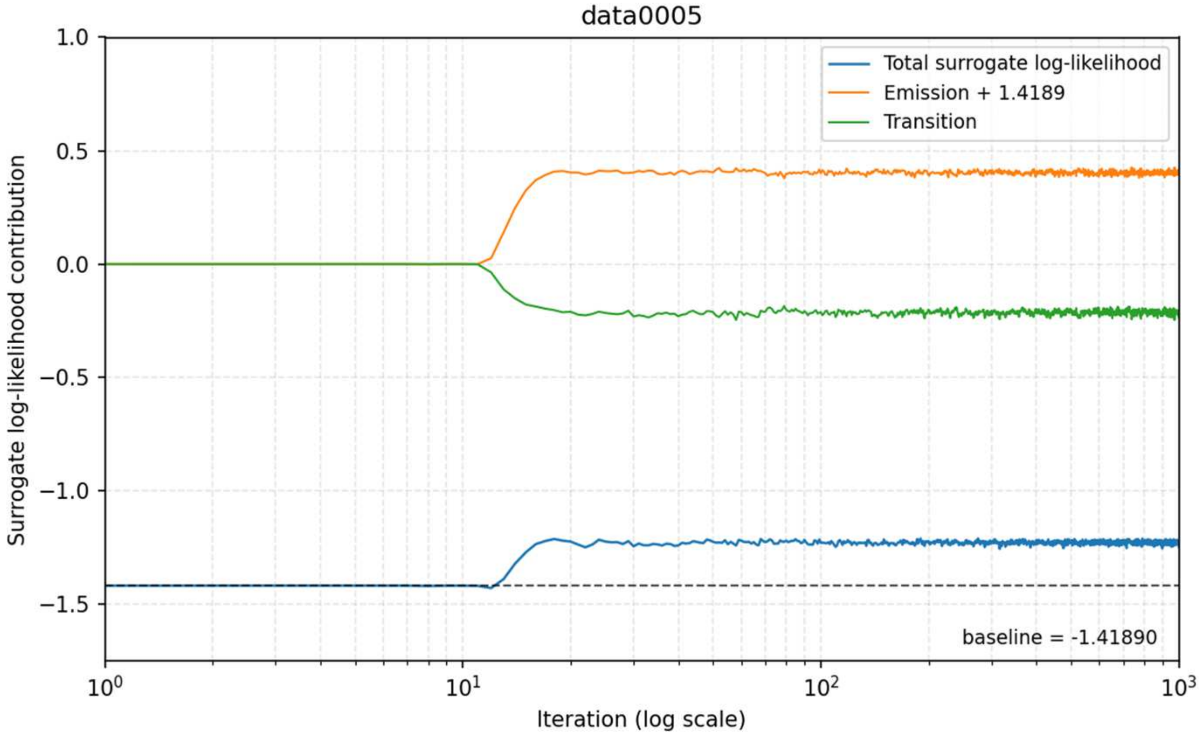
data0005
Total surrogate log-likelihood
Emission + 1.4189
Transition
Surrogate log-likelihood contribution
baseline = -1.41890
Iteration (log scale)

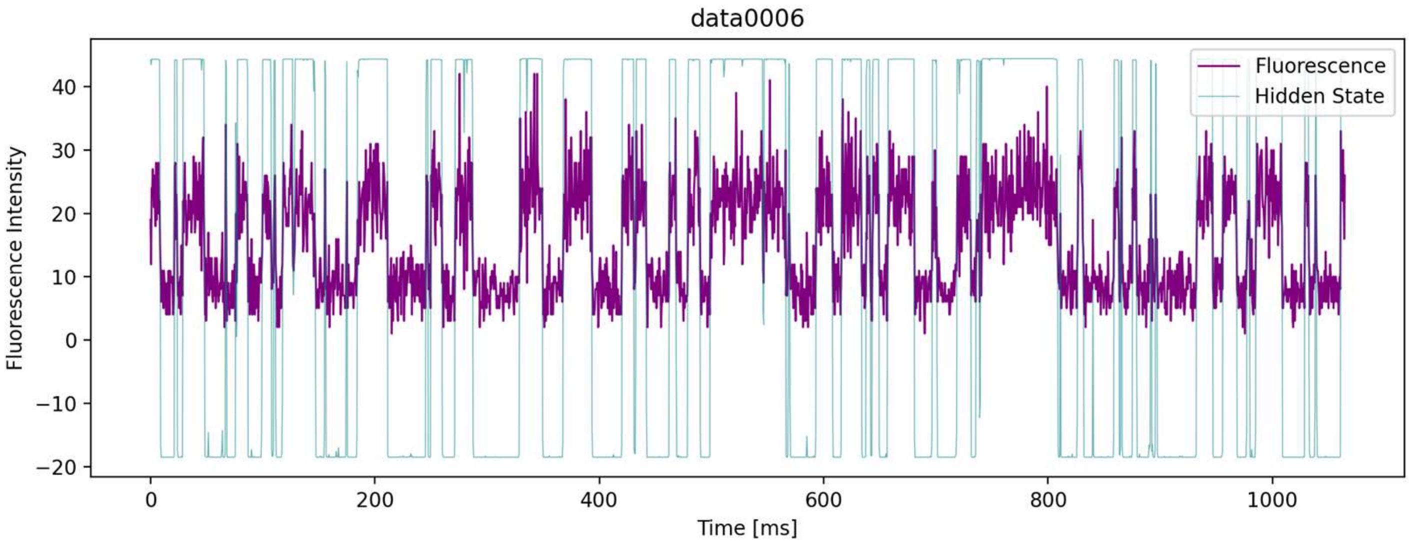
data0006
Fluorescence
Hidden State
Fluorescence Intensity
Time [ms]

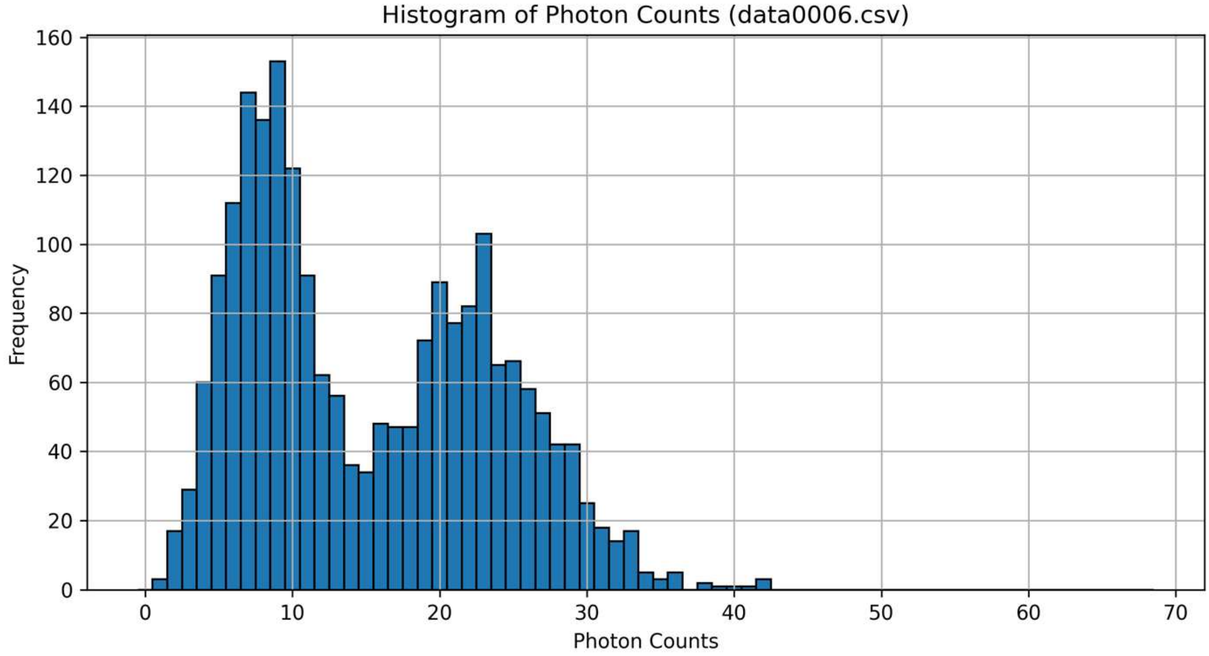
Histogram of Photon Counts (data0006.csv)
Frequency
Photon Counts

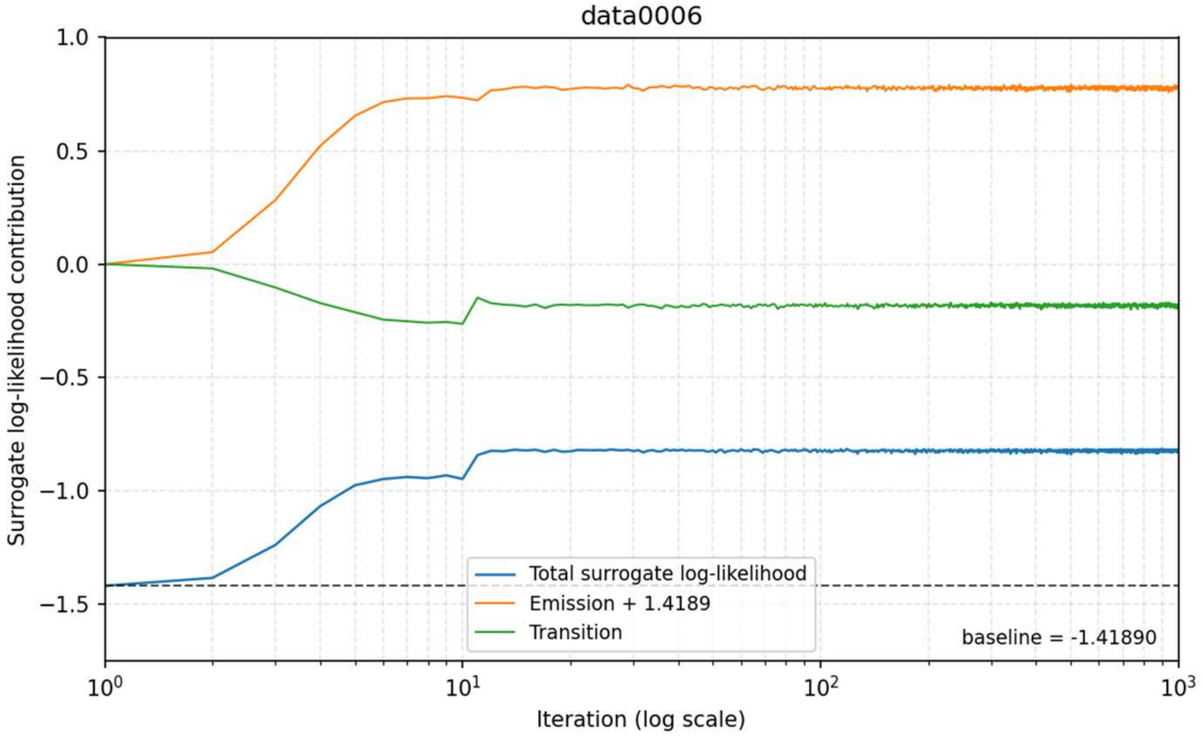
data0006
Surrogate log-likelihood contribution
Total surrogate log-likelihood
Emission + 1.4189
Transition
baseline = -1.41890
Iteration (log scale)

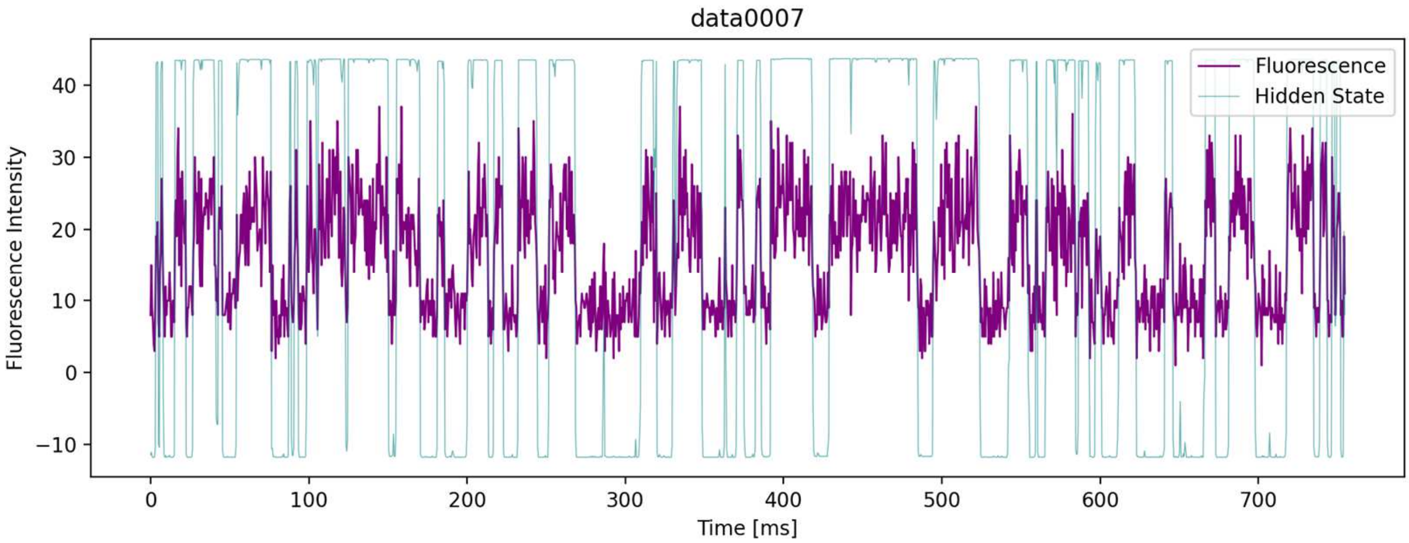

data0007
Fluorescence
Hidden State
Fluorescence Intensity
Time [ms]

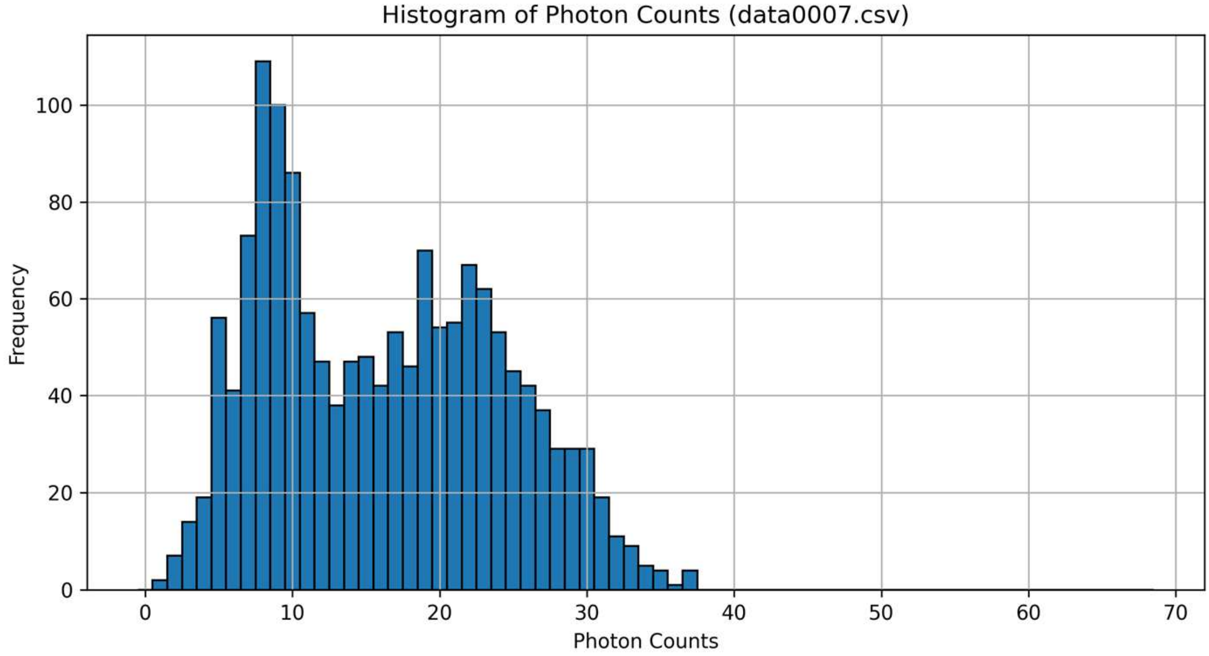

Histogram of Photon Counts (data0007.csv)
Frequency
Photon Counts

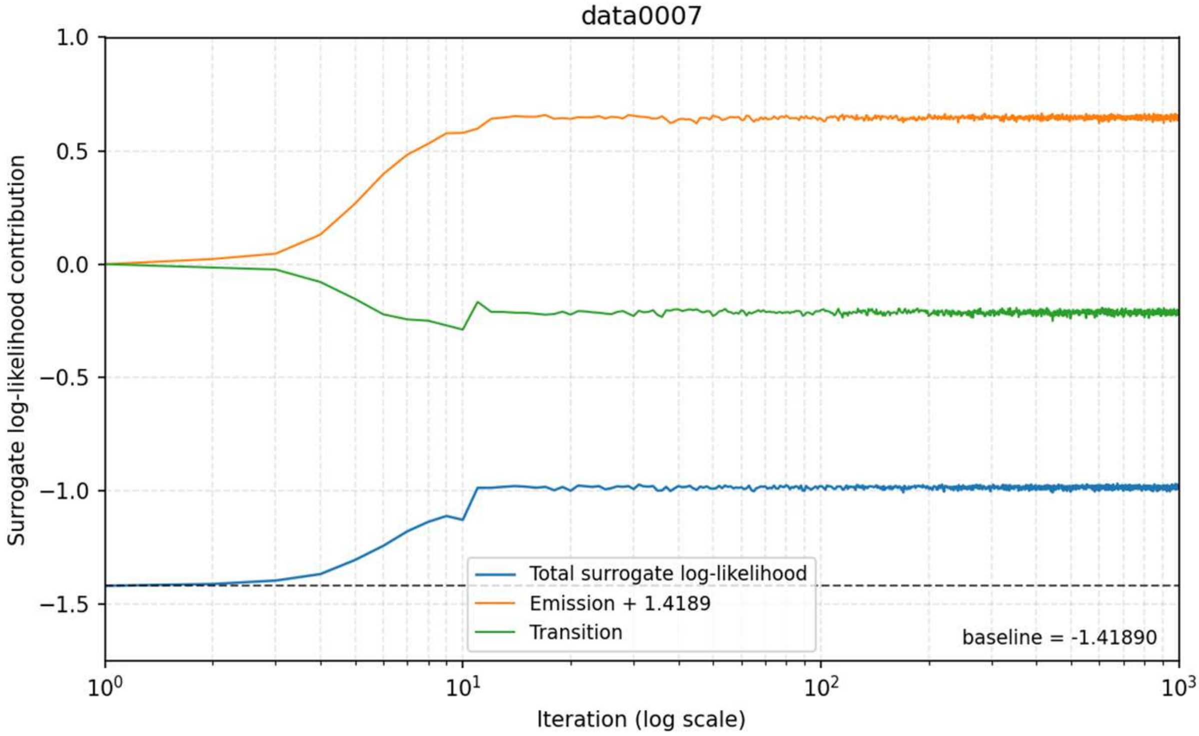

data0007
Surrogate log-likelihood contribution
Total surrogate log-likelihood
Emission + 1.4189
Transition
baseline = -1.41890
Iteration (log scale)

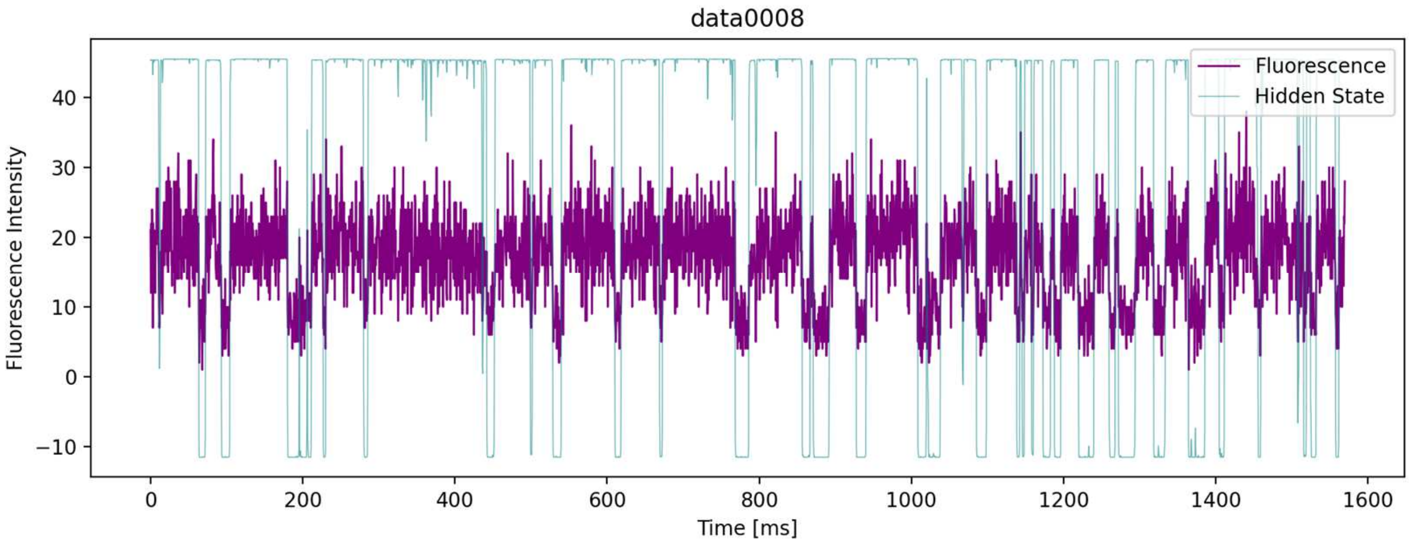

data0008
Fluorescence
Hidden State
Fluorescence Intensity
Time [ms]

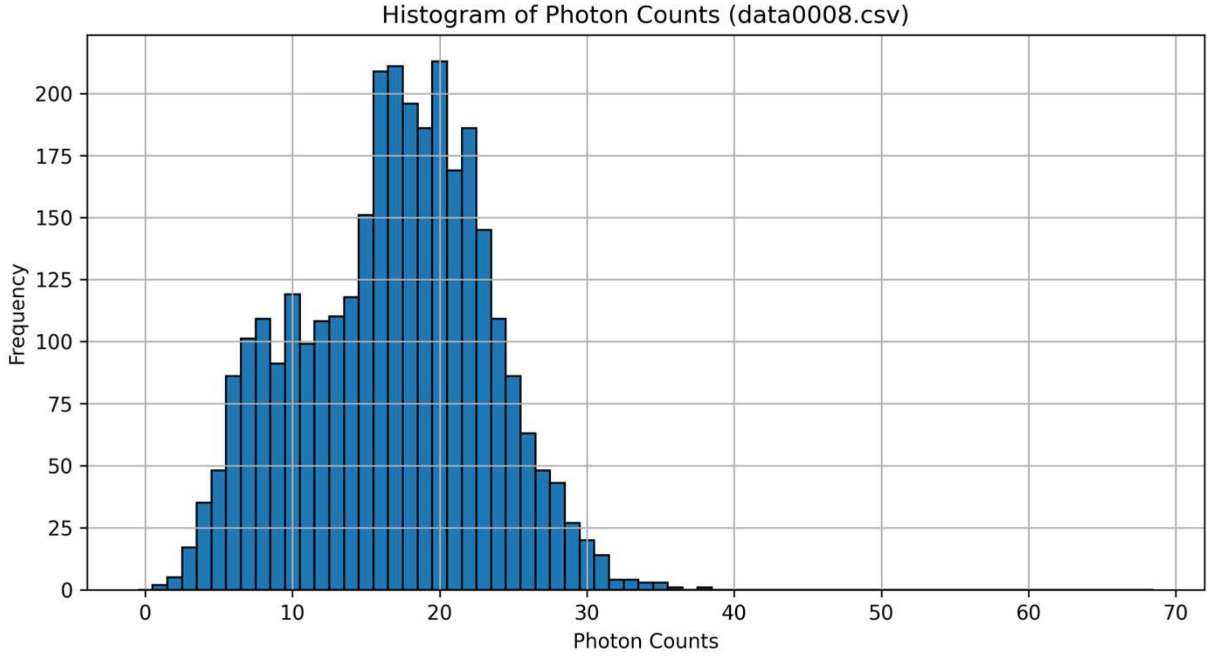

Histogram of Photon Counts (data0008.csv)
Frequency
Photon Counts

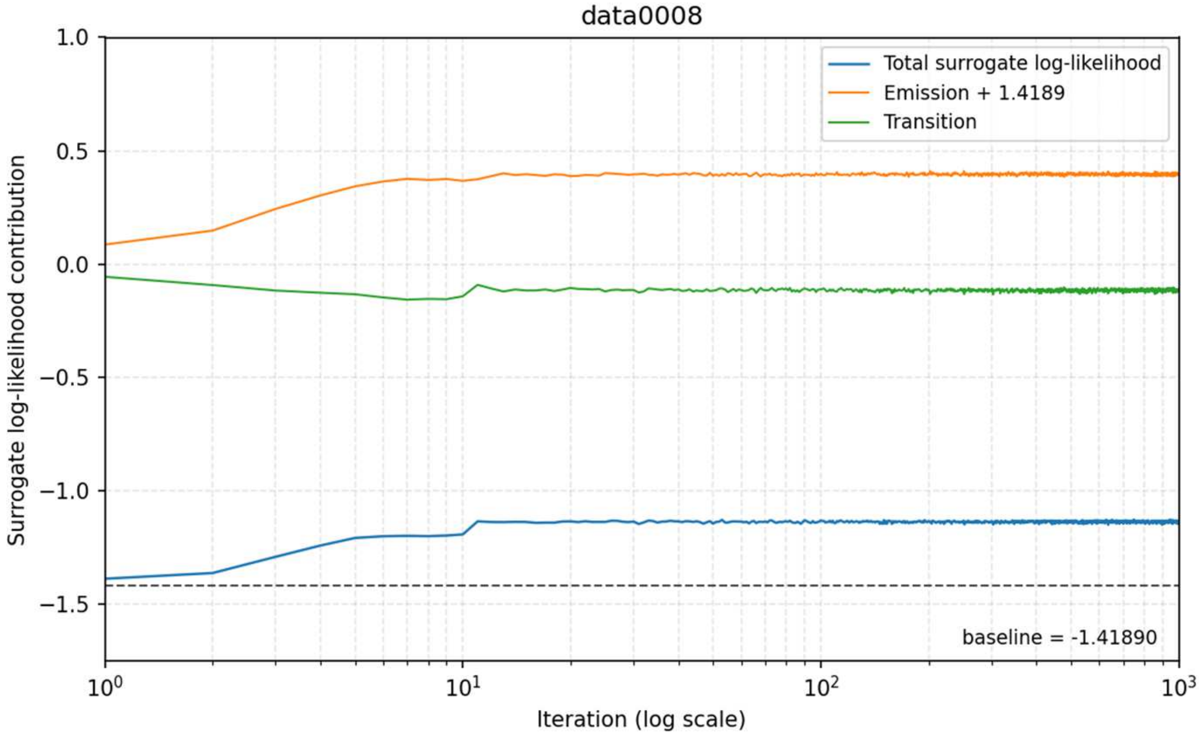

data0008
Total surrogate log-likelihood
Emission + 1.4189
Transition
Surrogate log-likelihood contribution
baseline = -1.41890
Iteration (log scale)

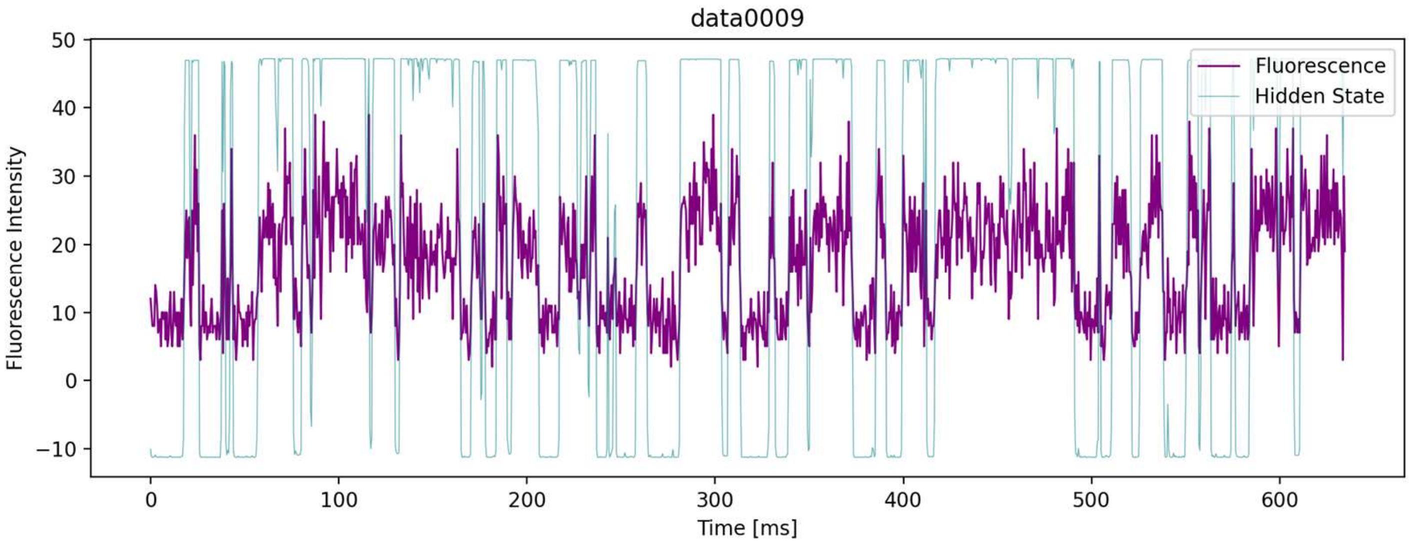
data0009
Fluorescence
Hidden State
Fluorescence Intensity
Time [ms]

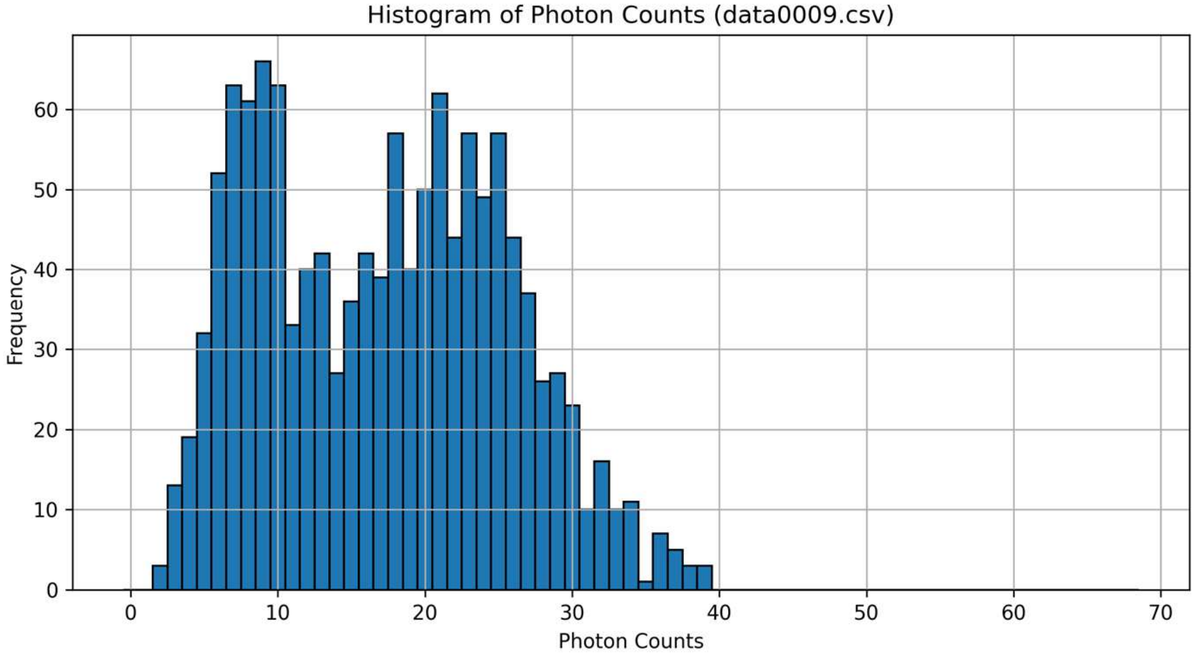
Histogram of Photon Counts (data0009.csv)
Frequency
Photon Counts

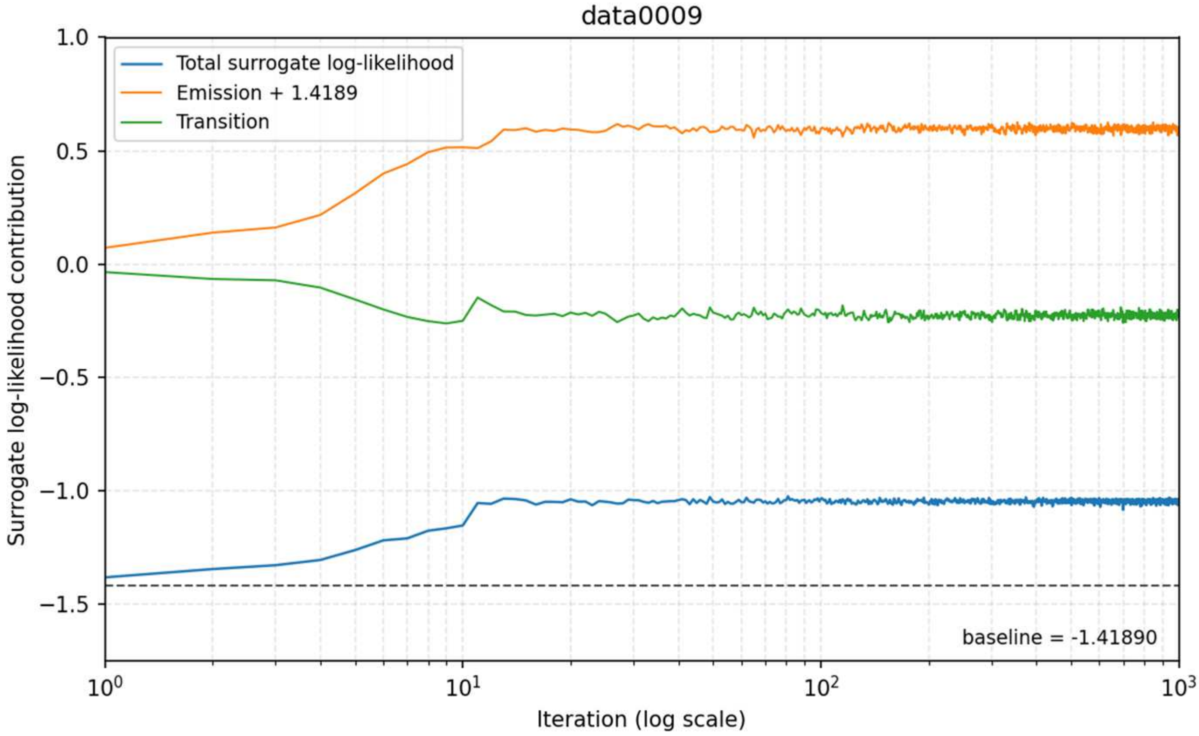
data0009
Total surrogate log-likelihood
Emission + 1.4189
Transition
Surrogate log-likelihood contribution
baseline = -1.41890
Iteration (log scale)

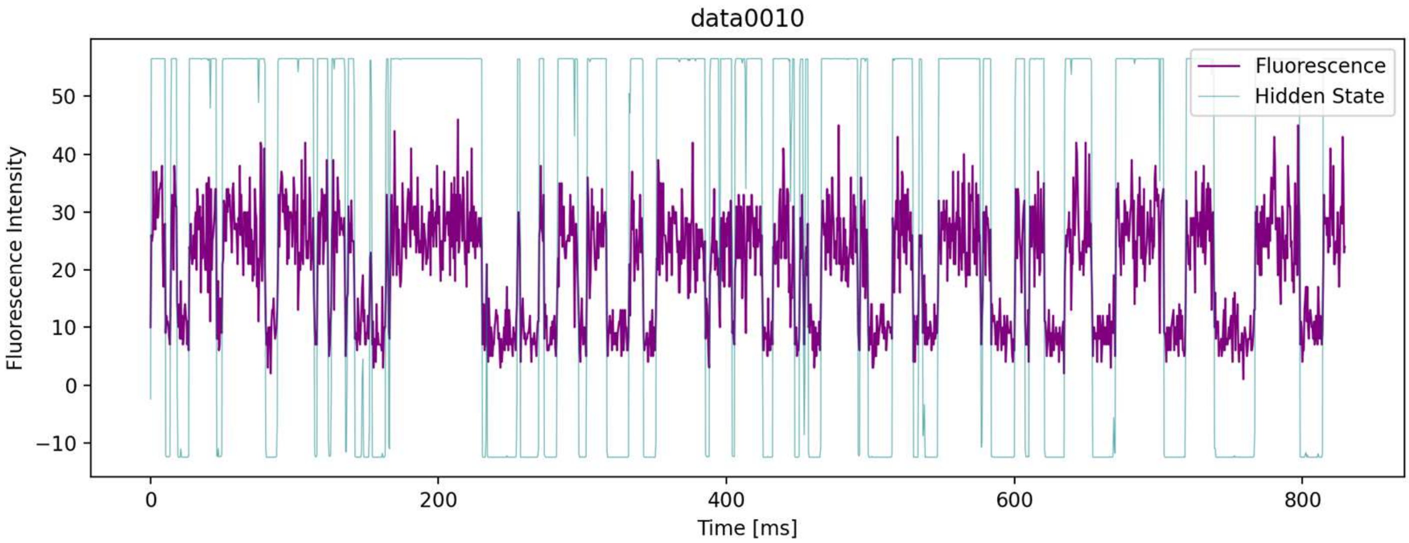
data0010
Fluorescence
Hidden State
Fluorescence Intensity
Time [ms]

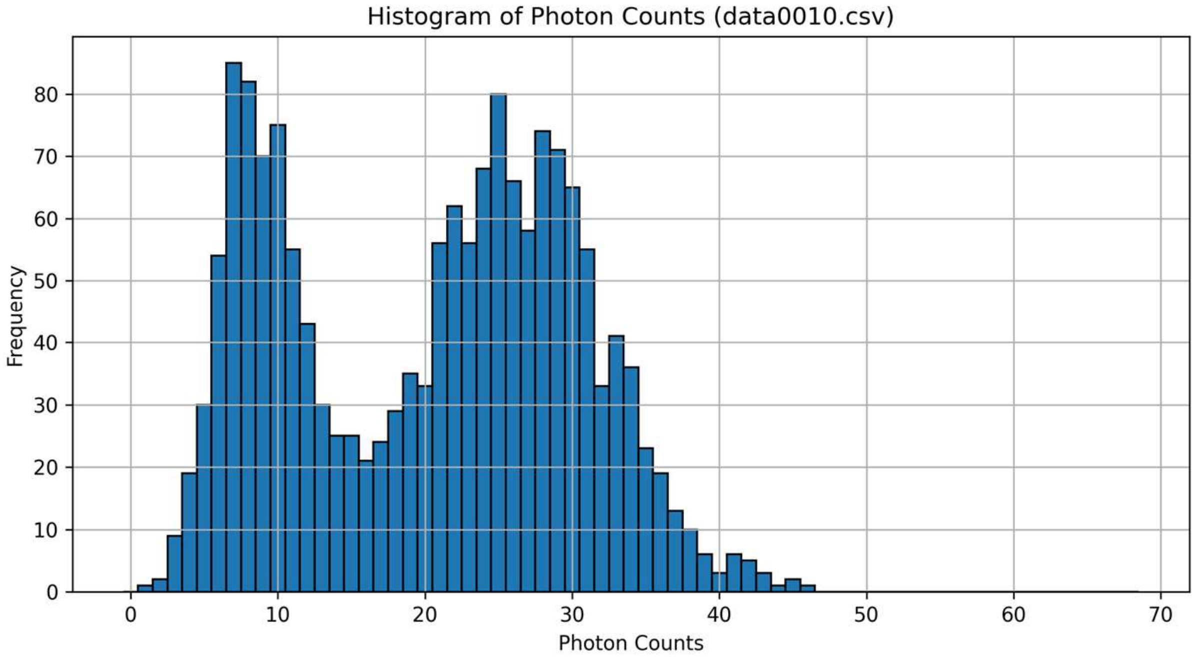
Histogram of Photon Counts (data0010.csv)
Frequency
Photon Counts

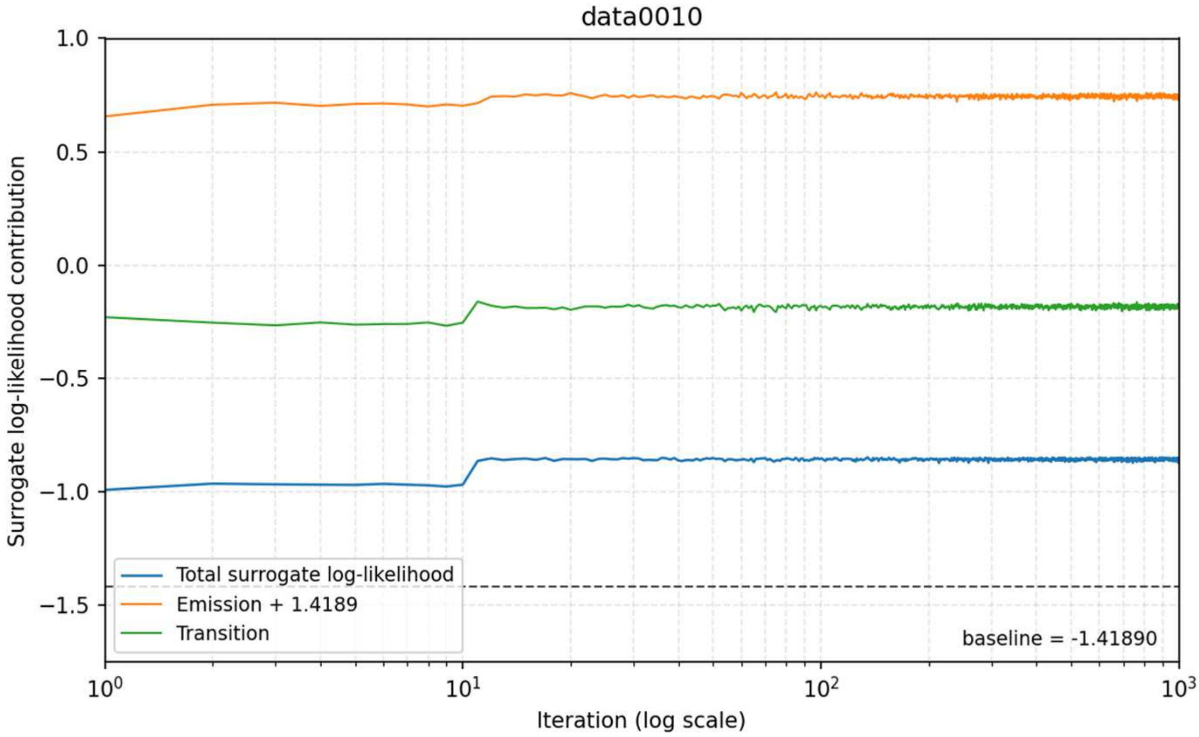
data0010
Surrogate log-likelihood contribution
Total surrogate log-likelihood
Emission + 1.4189
Transition
baseline = -1.41890
Iteration (log scale)

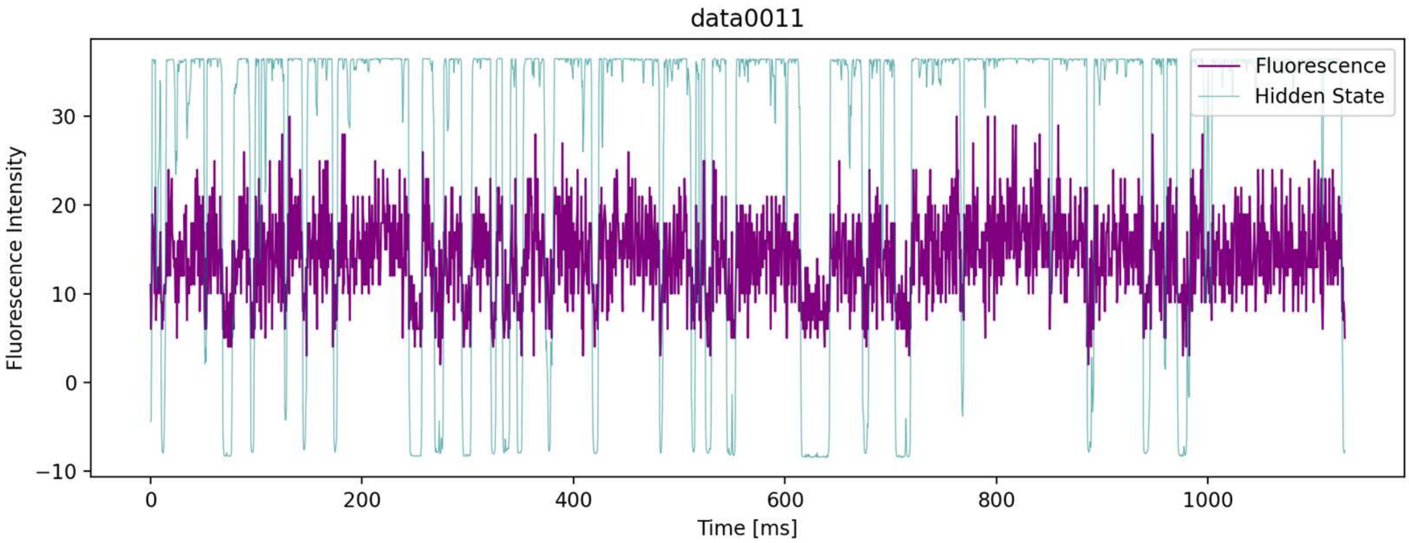
data0011
Fluorescence
Hidden State
Fluorescence Intensity
Time [ms]
30
20
10
0
−10
0
200
400
600
800
1000

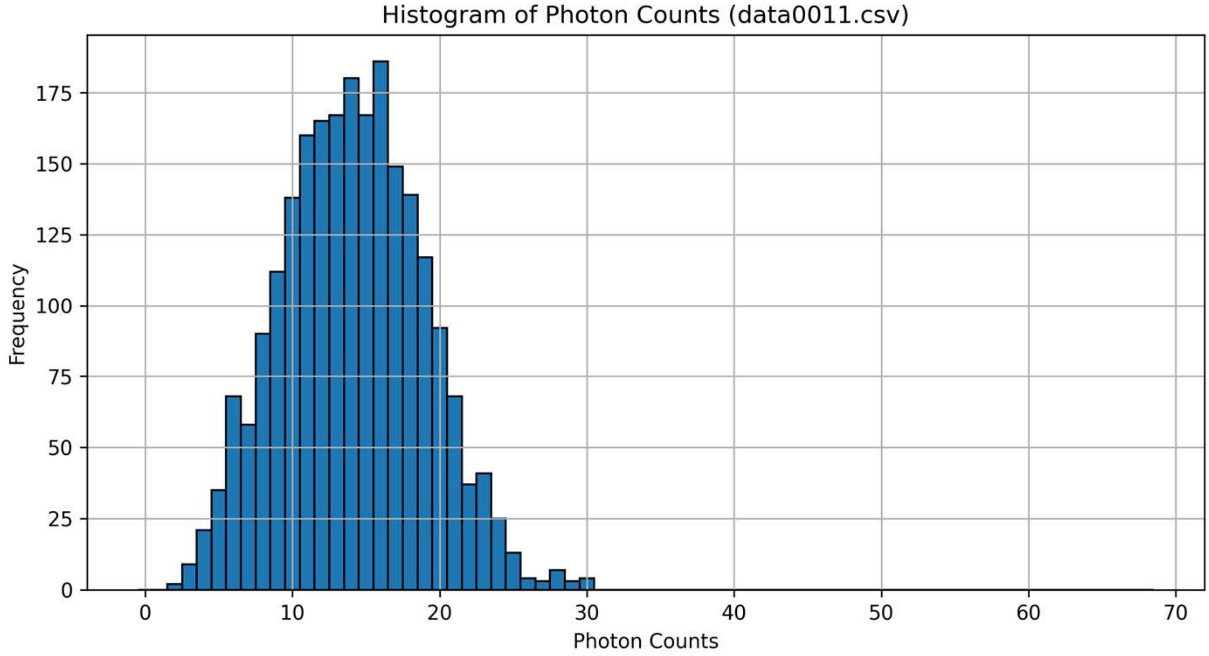
Histogram of Photon Counts (data0011.csv)
Frequency
Photon Counts
175
150
125
100
75
50
25
0
0
10
20
30
40
50
60
70

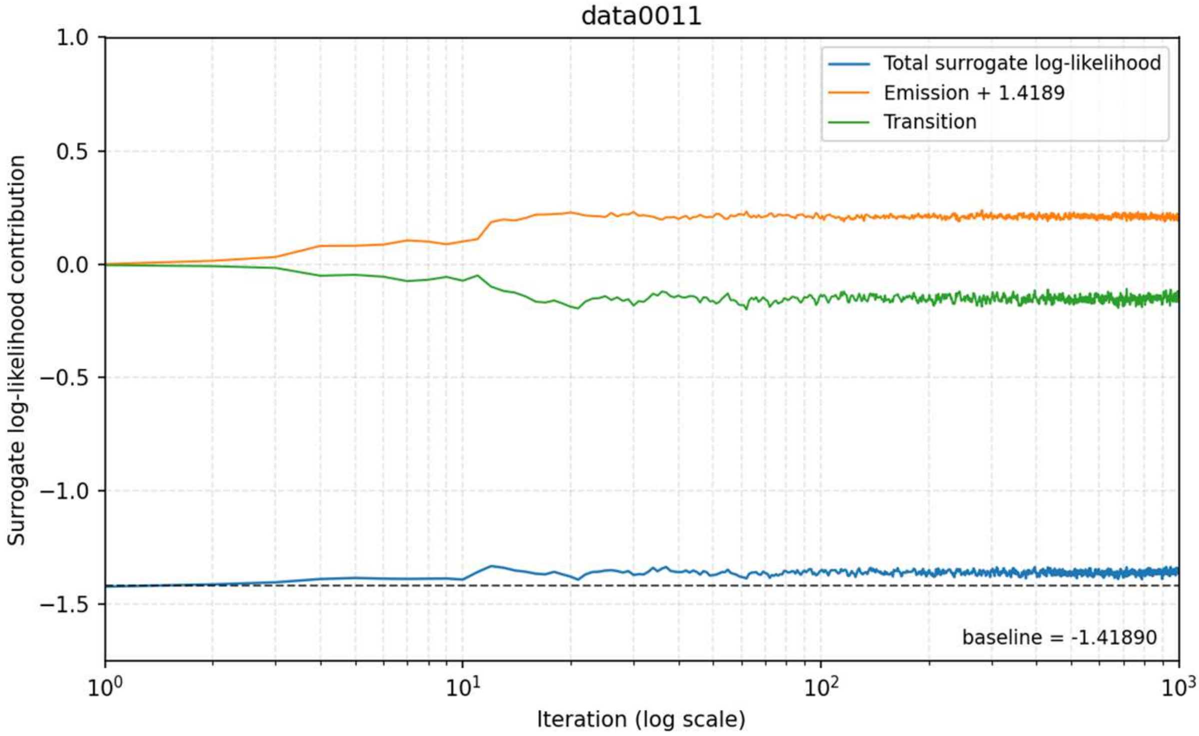
data0011
Total surrogate log-likelihood
Emission + 1.4189
Transition
Surrogate log-likelihood contribution
baseline = -1.41890
Iteration (log scale)
1.0
0.5
0.0
−0.5
−1.0
−1.5

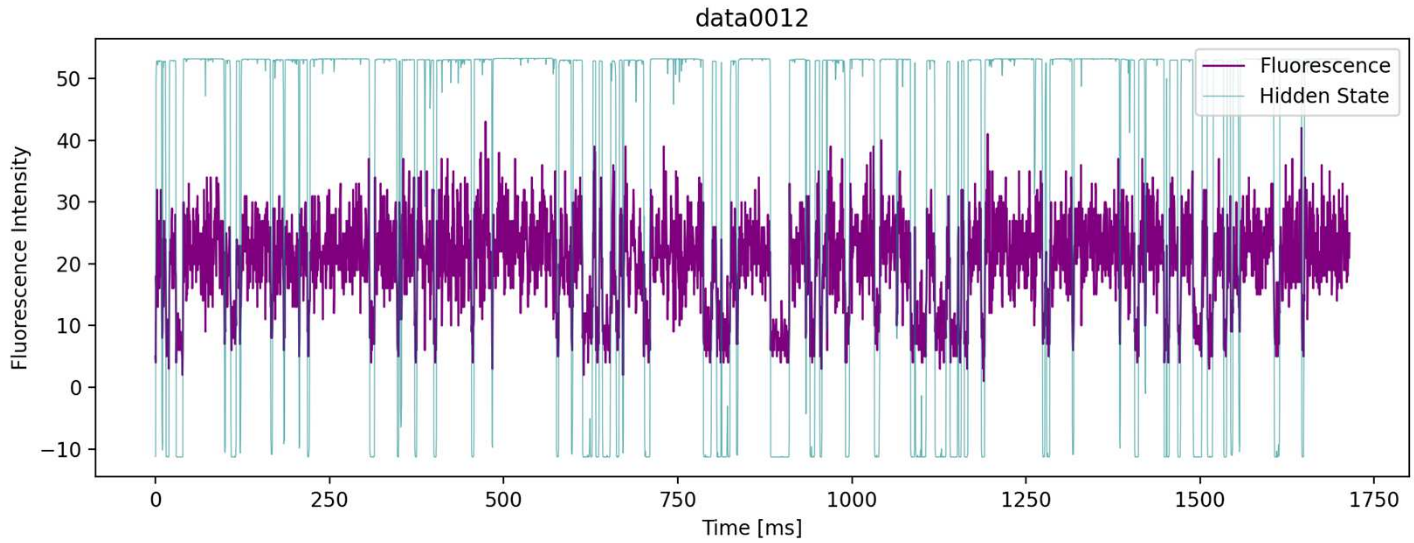
data0012
Fluorescence
Hidden State
Fluorescence Intensity
Time [ms]

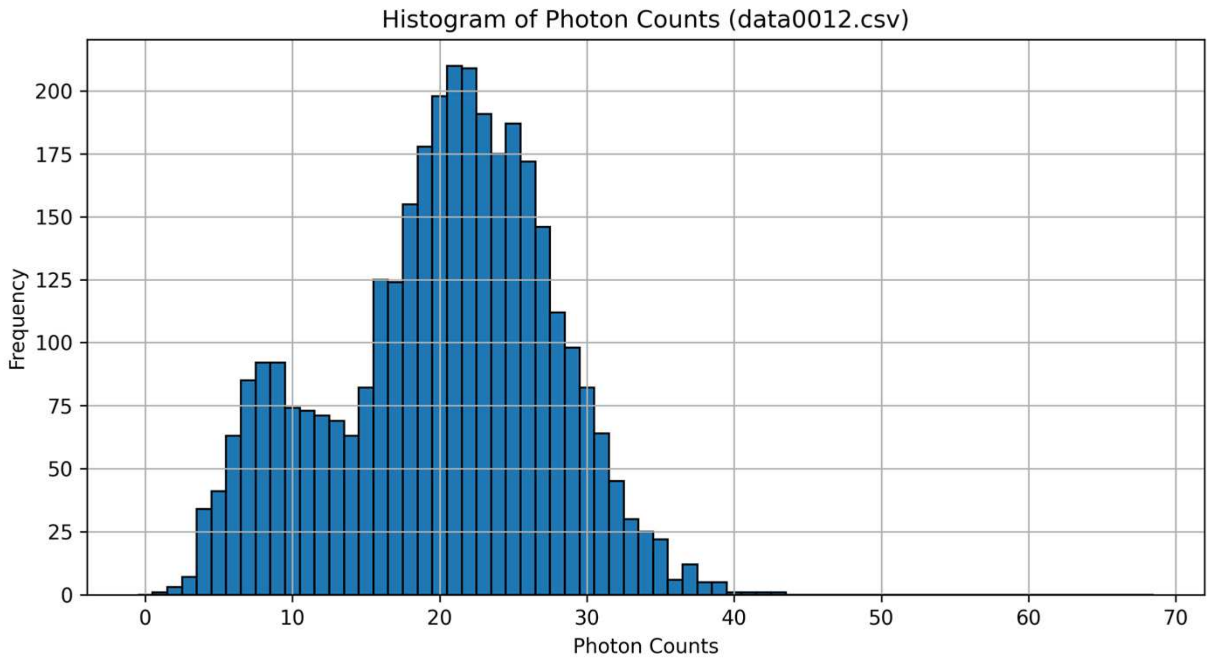
Histogram of Photon Counts (data0012.csv)
Frequency
Photon Counts

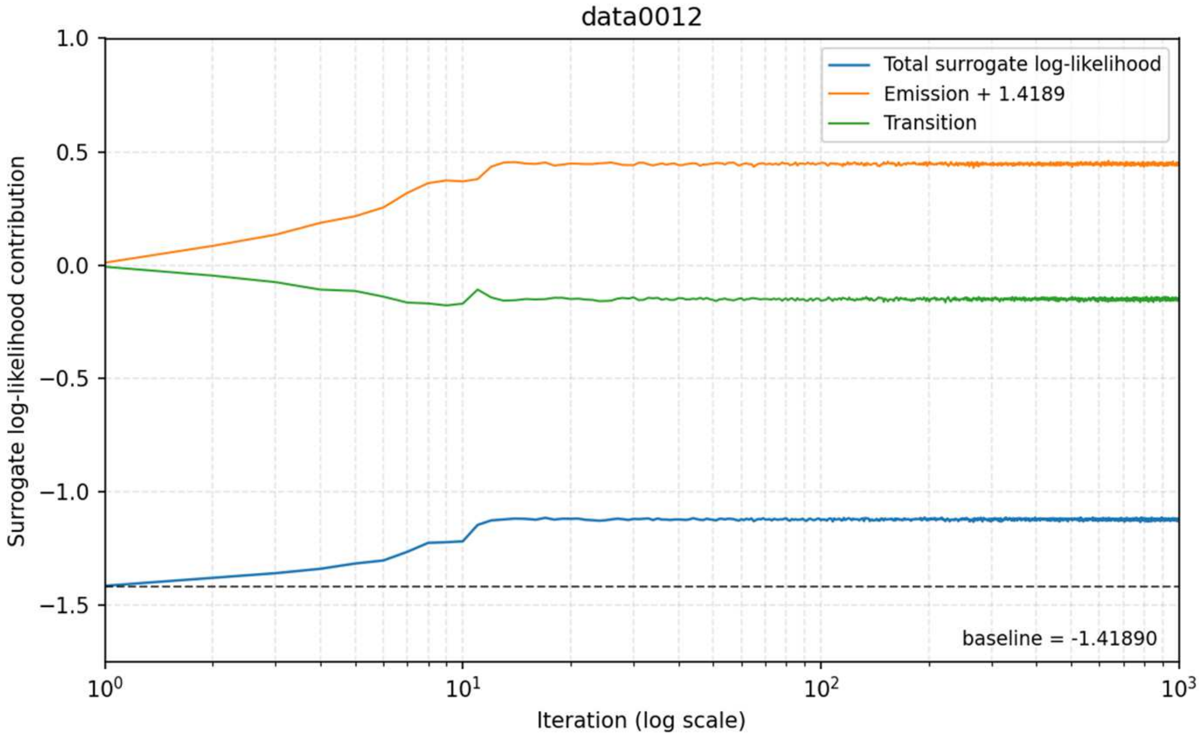
data0012
Total surrogate log-likelihood
Emission + 1.4189
Transition
Surrogate log-likelihood contribution
baseline = -1.41890
Iteration (log scale)

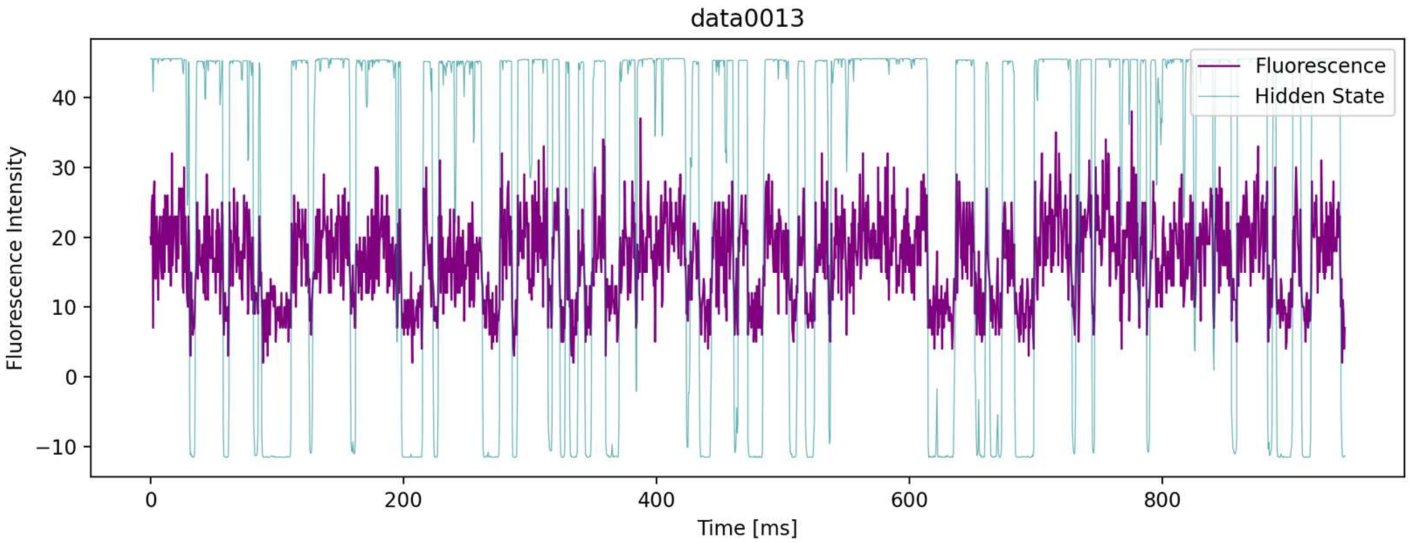

data0013
Fluorescence
Hidden State
Fluorescence Intensity
Time [ms]

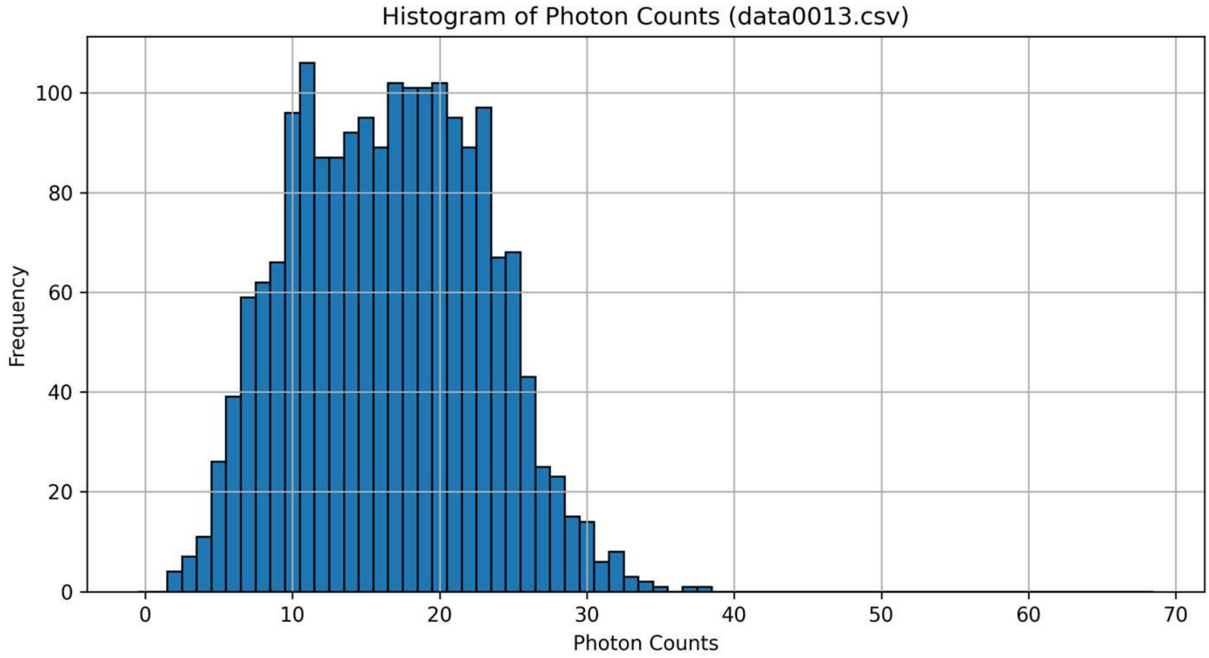

Histogram of Photon Counts (data0013.csv)
Frequency
Photon Counts

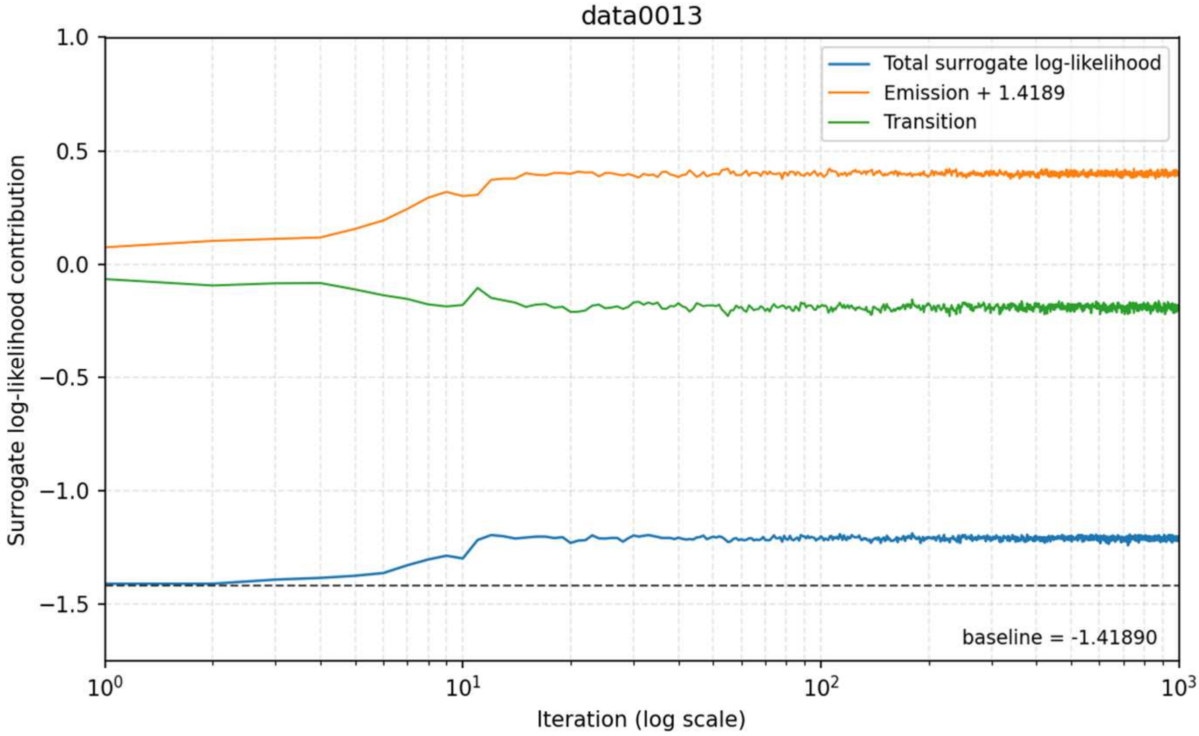

data0013
Total surrogate log-likelihood
Emission + 1.4189
Transition
Surrogate log-likelihood contribution
baseline = -1.41890
Iteration (log scale)

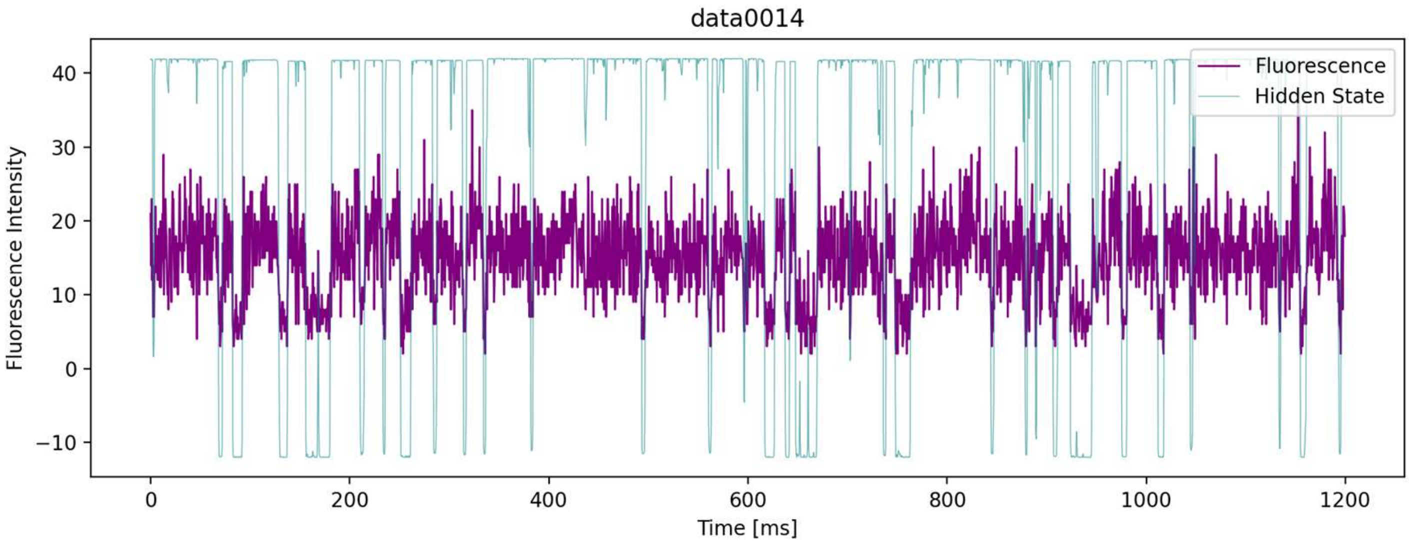
data0014
Fluorescence
Hidden State
Fluorescence Intensity
Time [ms]
40
30
20
10
0
−10
0
200
400
600
800
1000
1200

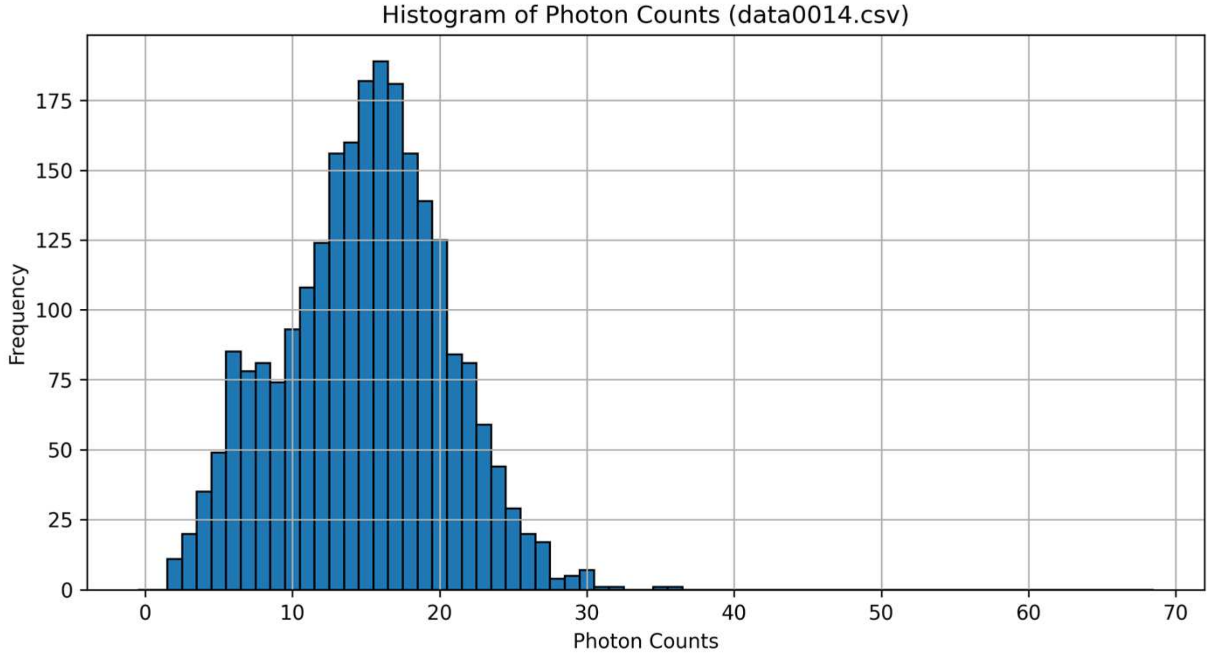
Histogram of Photon Counts (data0014.csv)
Frequency
Photon Counts
175
150
125
100
75
50
25
0
0
10
20
30
40
50
60
70

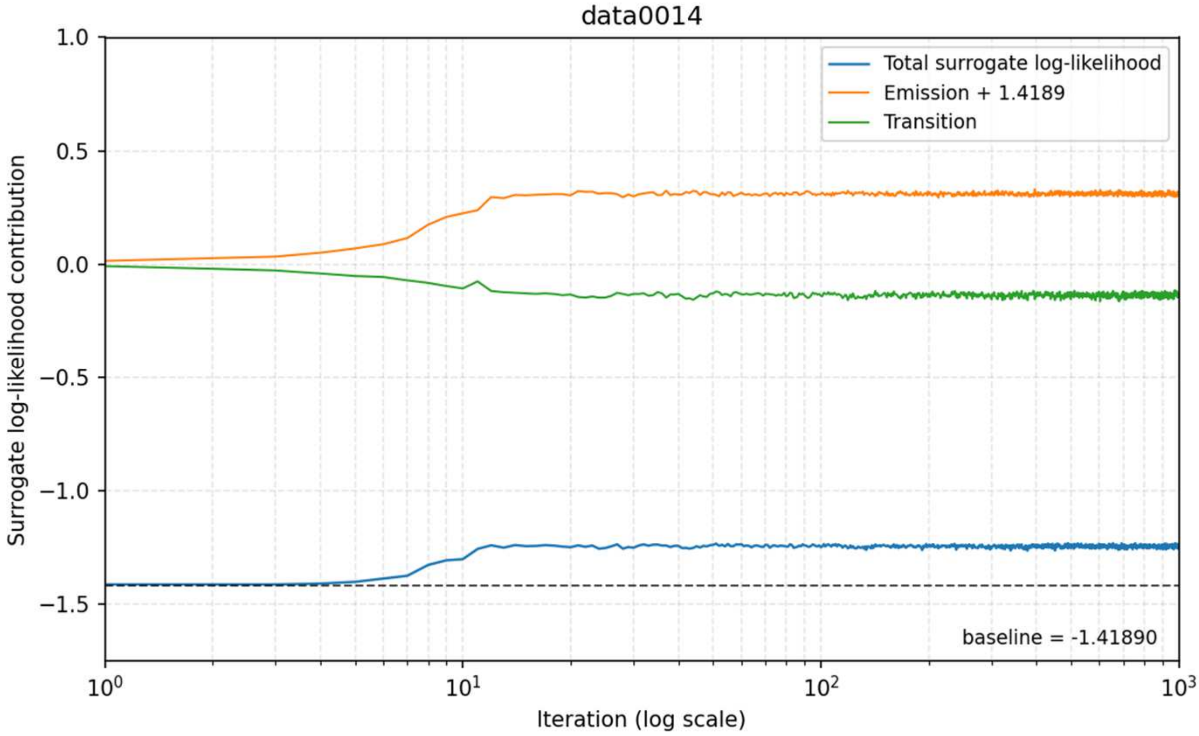
data0014
Total surrogate log-likelihood
Emission + 1.4189
Transition
Surrogate log-likelihood contribution
Iteration (log scale)
baseline = -1.41890
1.0
0.5
0.0
−0.5
−1.0
−1.5

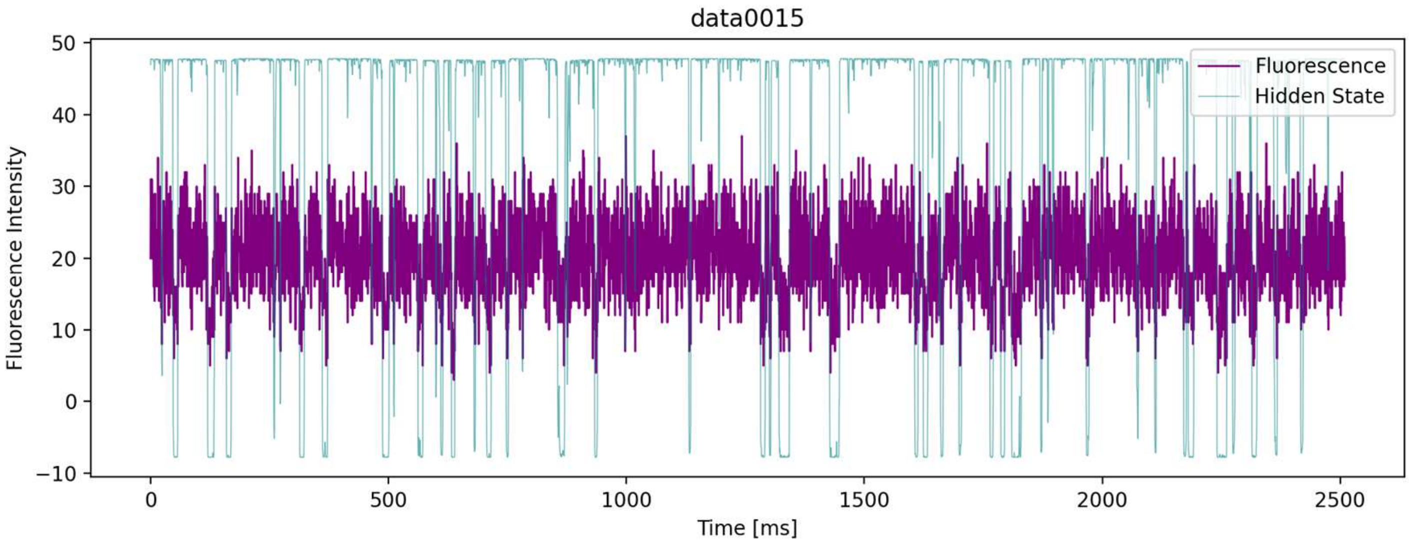

data0015
Fluorescence
Hidden State
Fluorescence Intensity
Time [ms]

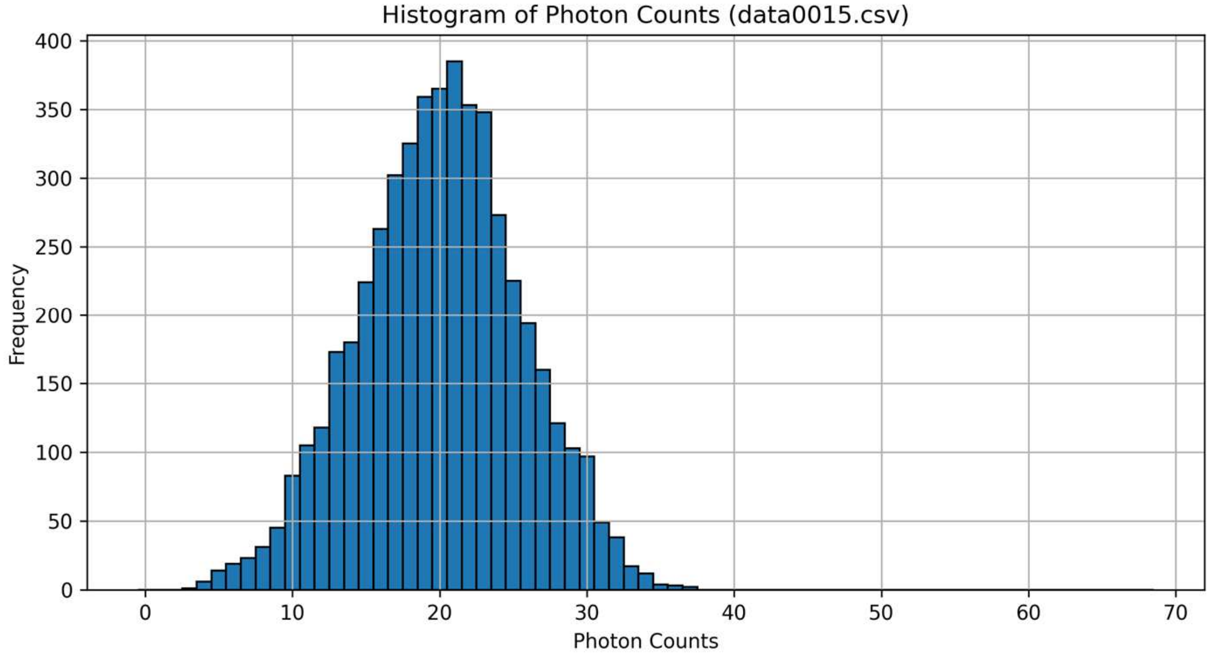

Histogram of Photon Counts (data0015.csv)
Frequency
Photon Counts

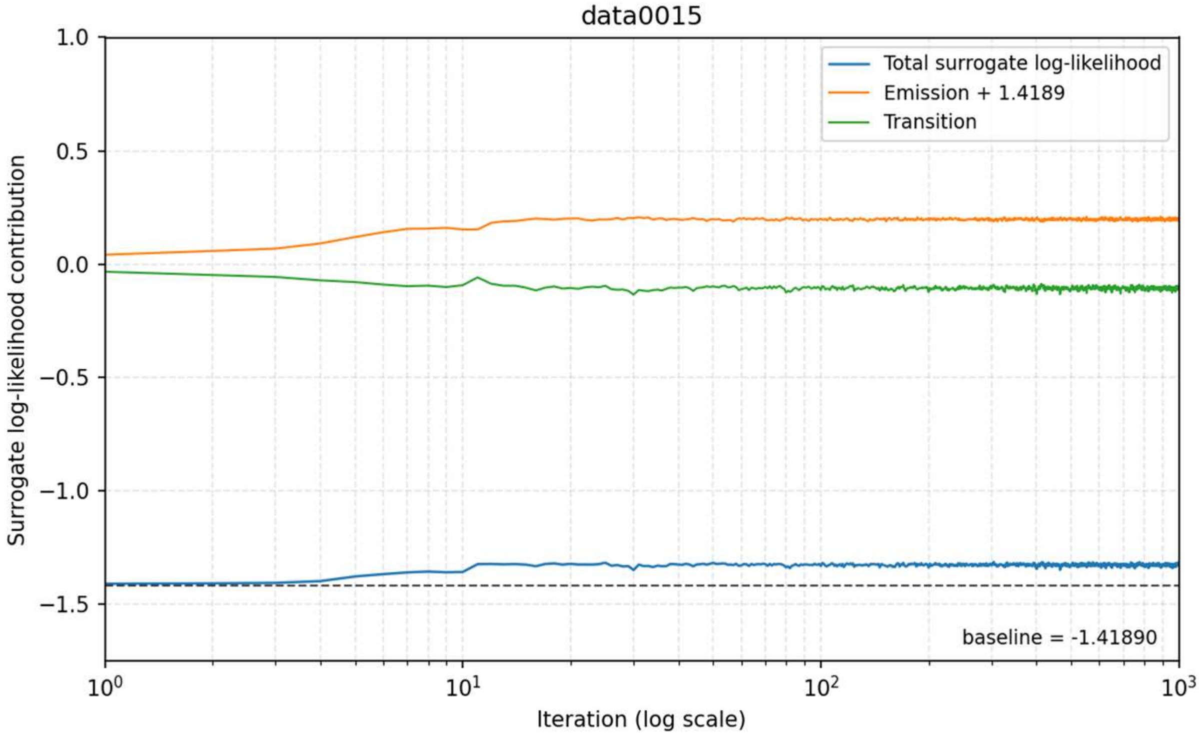

data0015
Total surrogate log-likelihood
Emission + 1.4189
Transition
Surrogate log-likelihood contribution
baseline = -1.41890
Iteration (log scale)

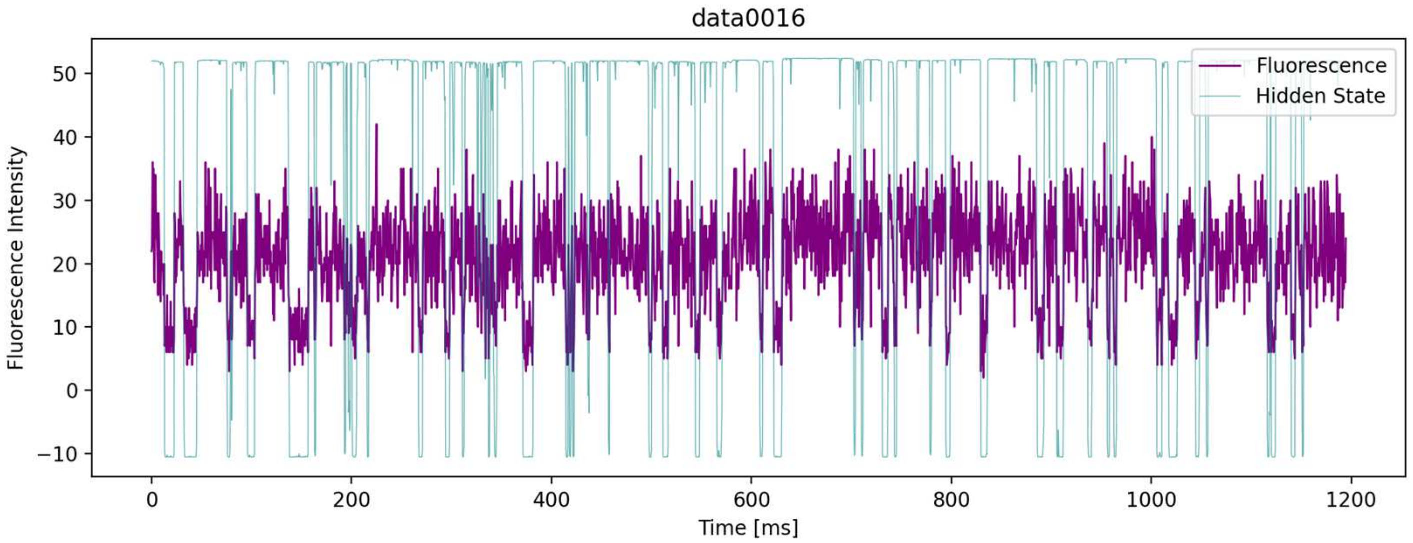

data0016
Fluorescence
Hidden State
Fluorescence Intensity
Time [ms]

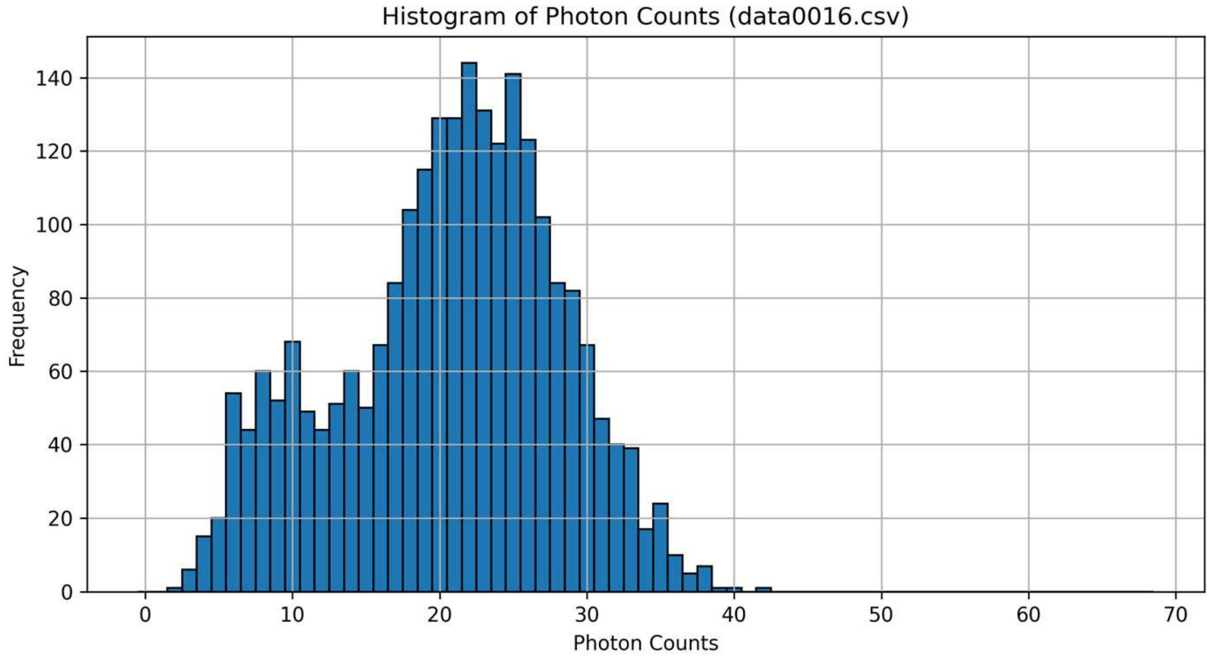

Histogram of Photon Counts (data0016.csv)
Frequency
Photon Counts

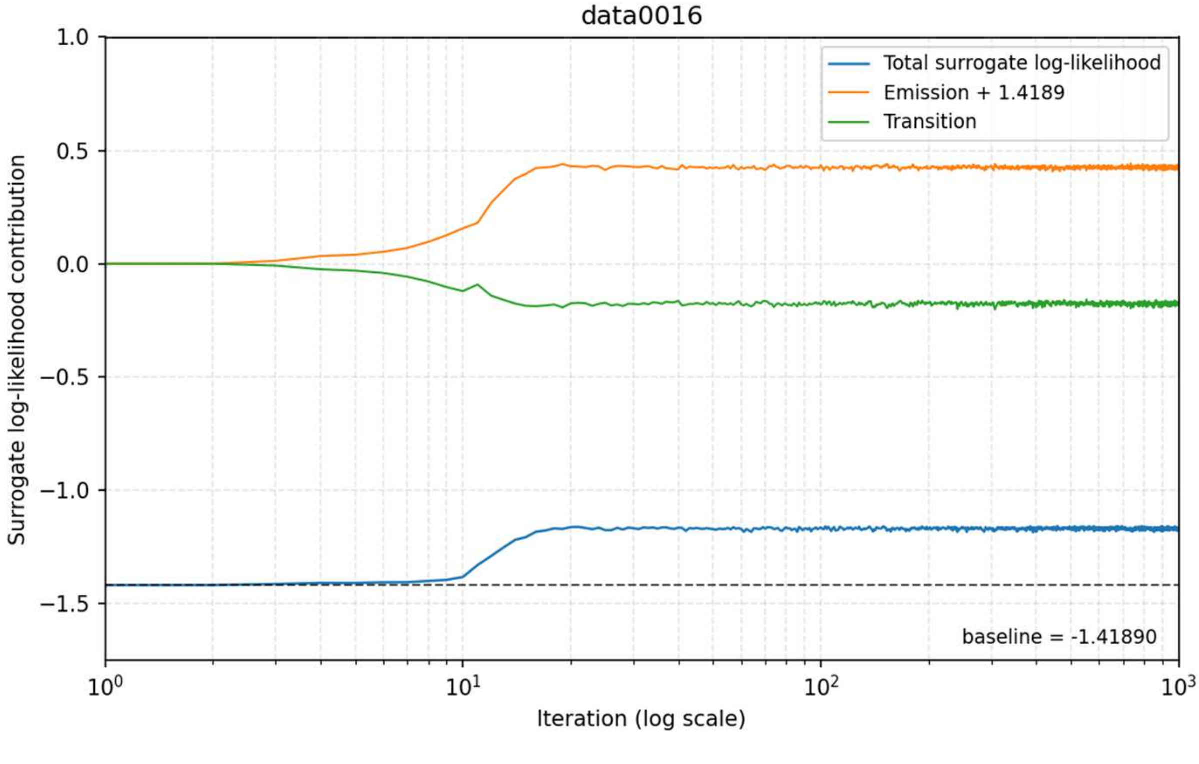

data0016
Total surrogate log-likelihood
Emission + 1.4189
Transition
Surrogate log-likelihood contribution
baseline = -1.41890
Iteration (log scale)

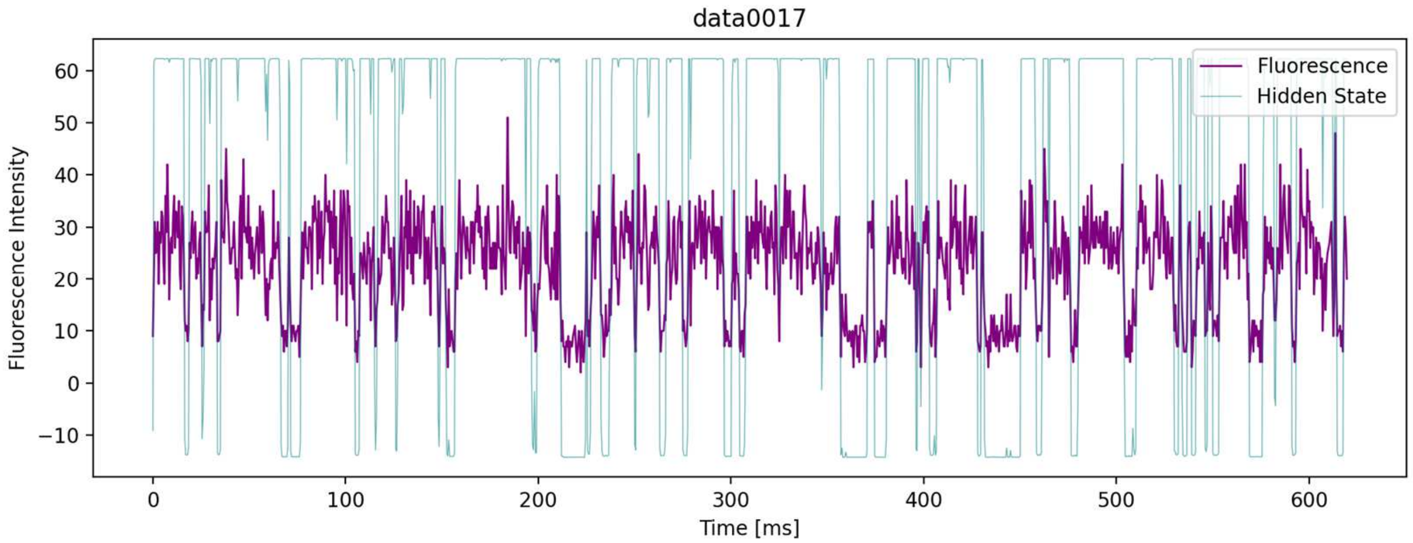
data0017
Fluorescence
Hidden State
Fluorescence Intensity
Time [ms]

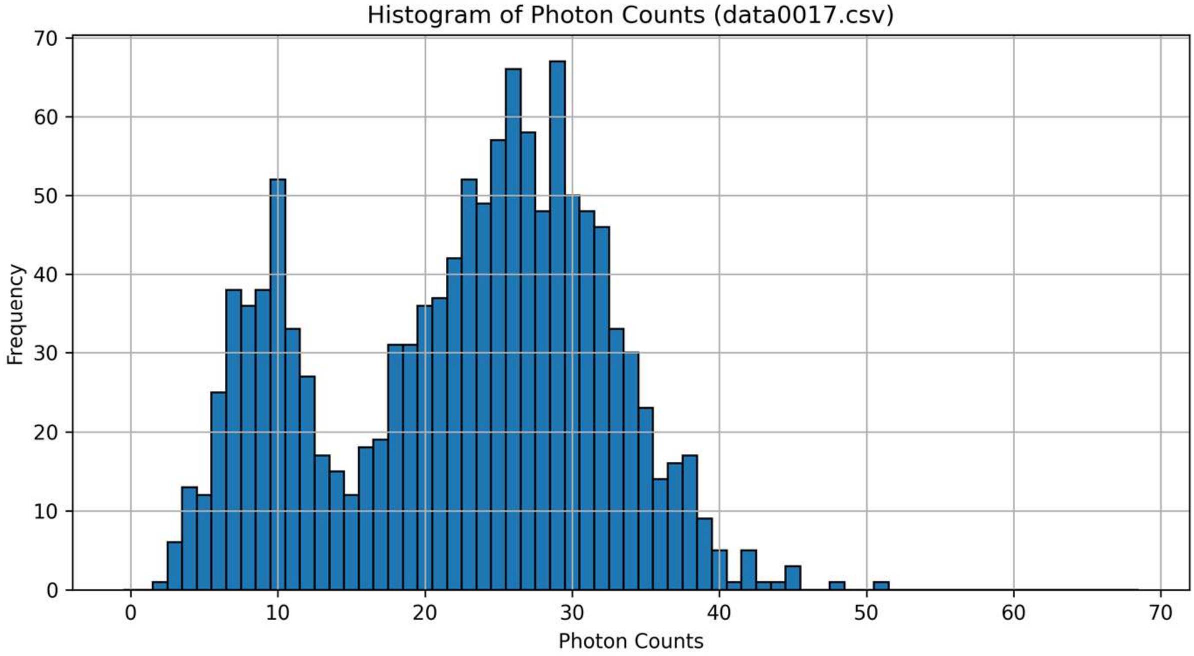
Histogram of Photon Counts (data0017.csv)
Frequency
Photon Counts

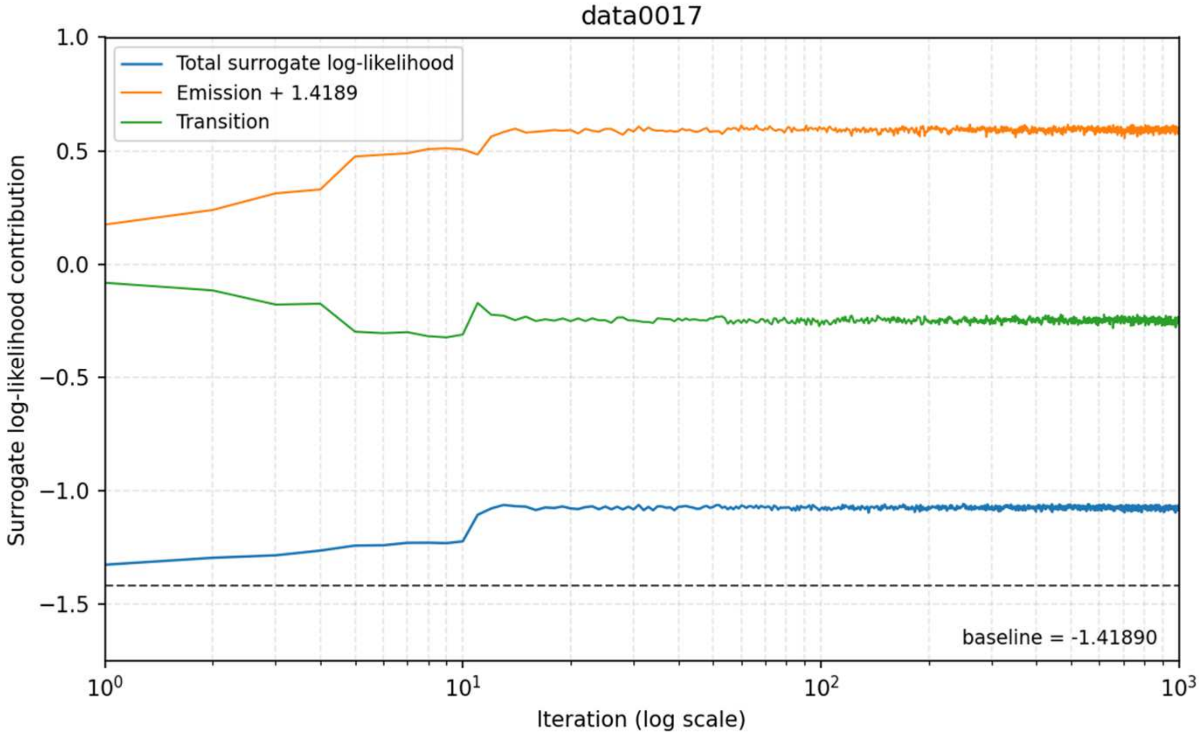
data0017
Total surrogate log-likelihood
Emission + 1.4189
Transition
Surrogate log-likelihood contribution
baseline = -1.41890
Iteration (log scale)

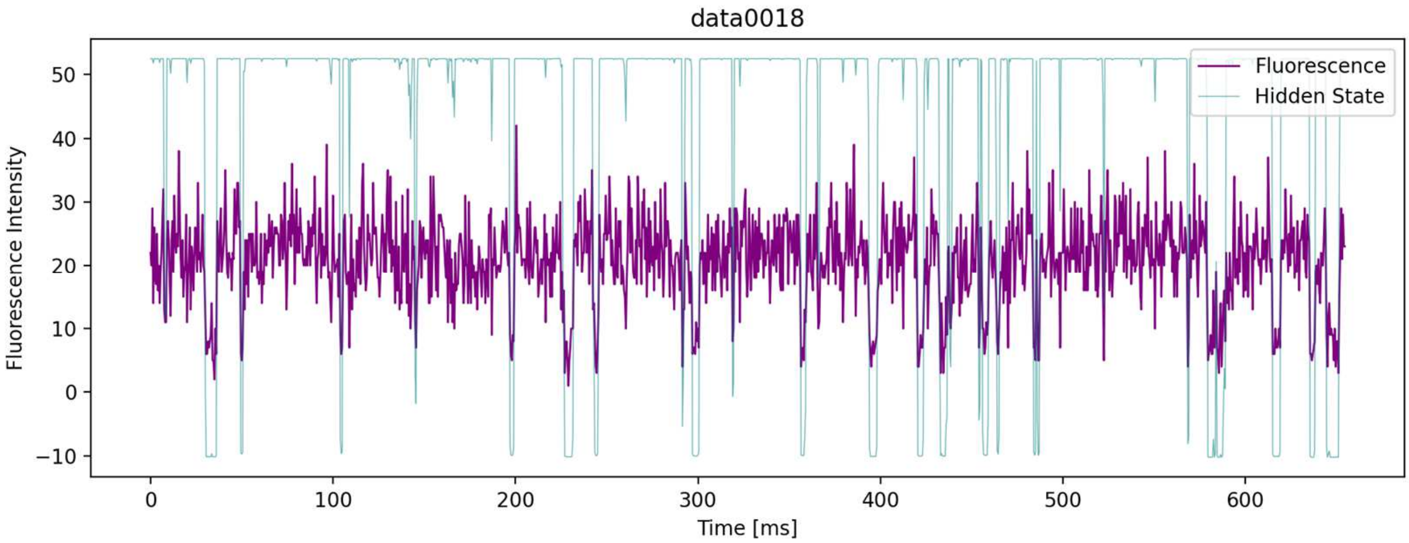
data0018
Fluorescence
Hidden State
Fluorescence Intensity
Time [ms]

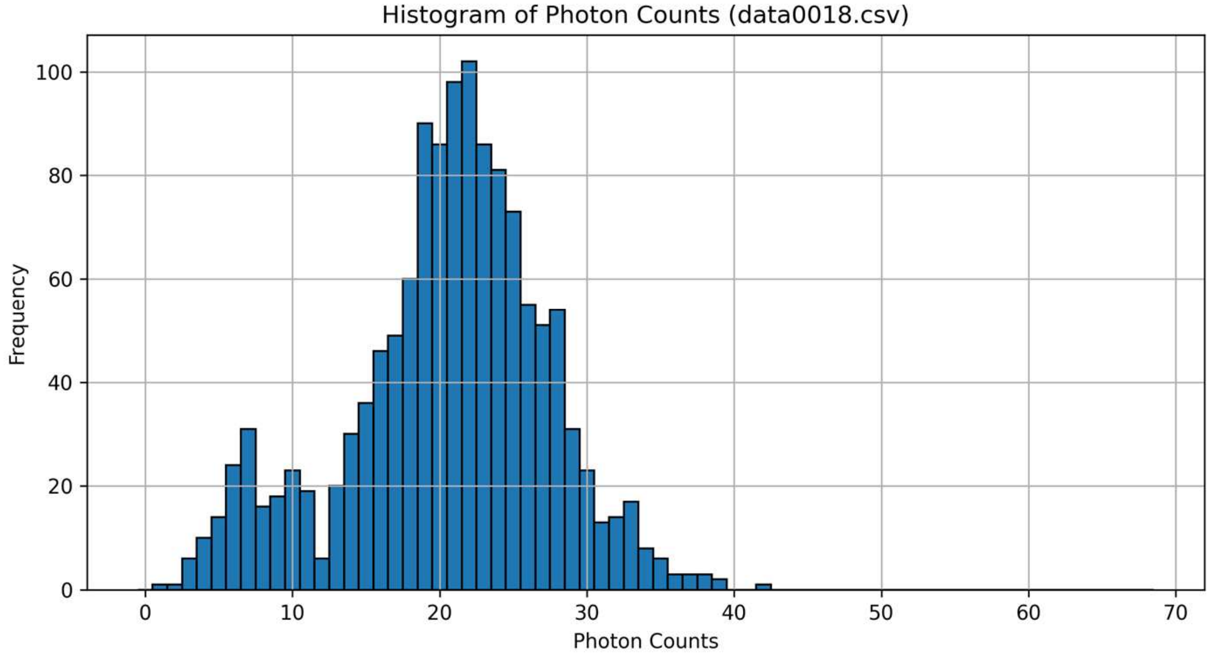
Histogram of Photon Counts (data0018.csv)
Frequency
Photon Counts

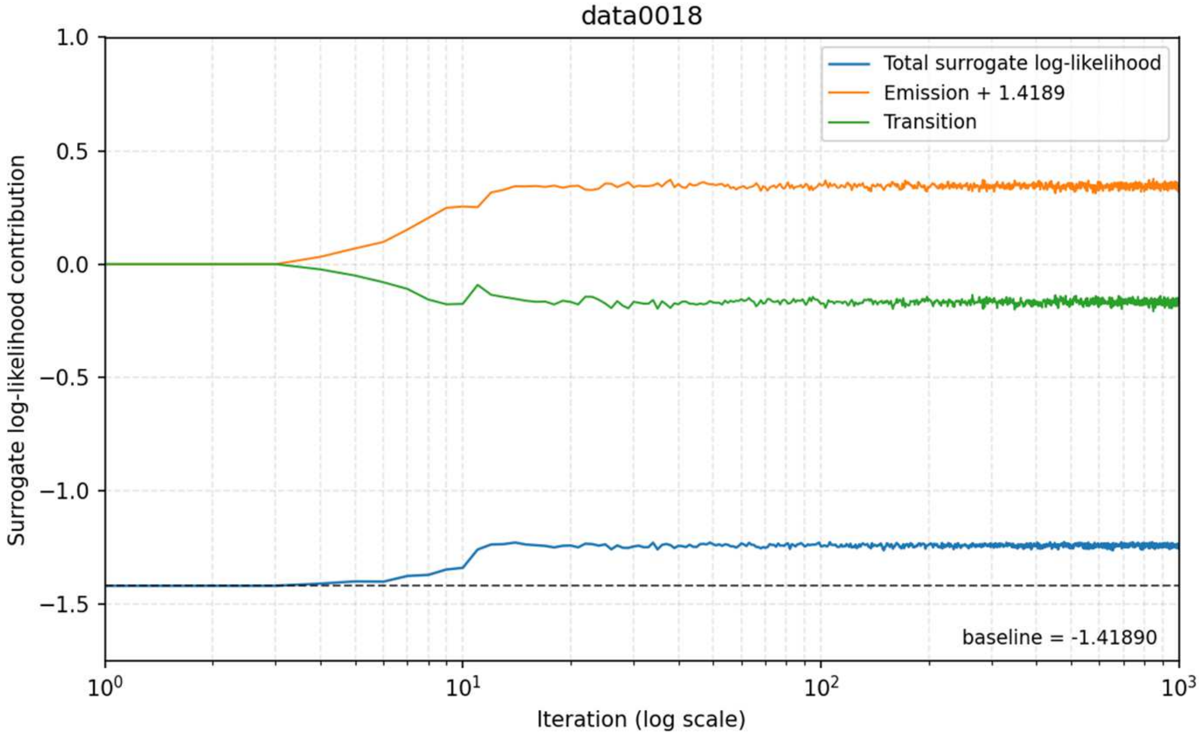
data0018
Total surrogate log-likelihood
Emission + 1.4189
Transition
Surrogate log-likelihood contribution
baseline = -1.41890
Iteration (log scale)

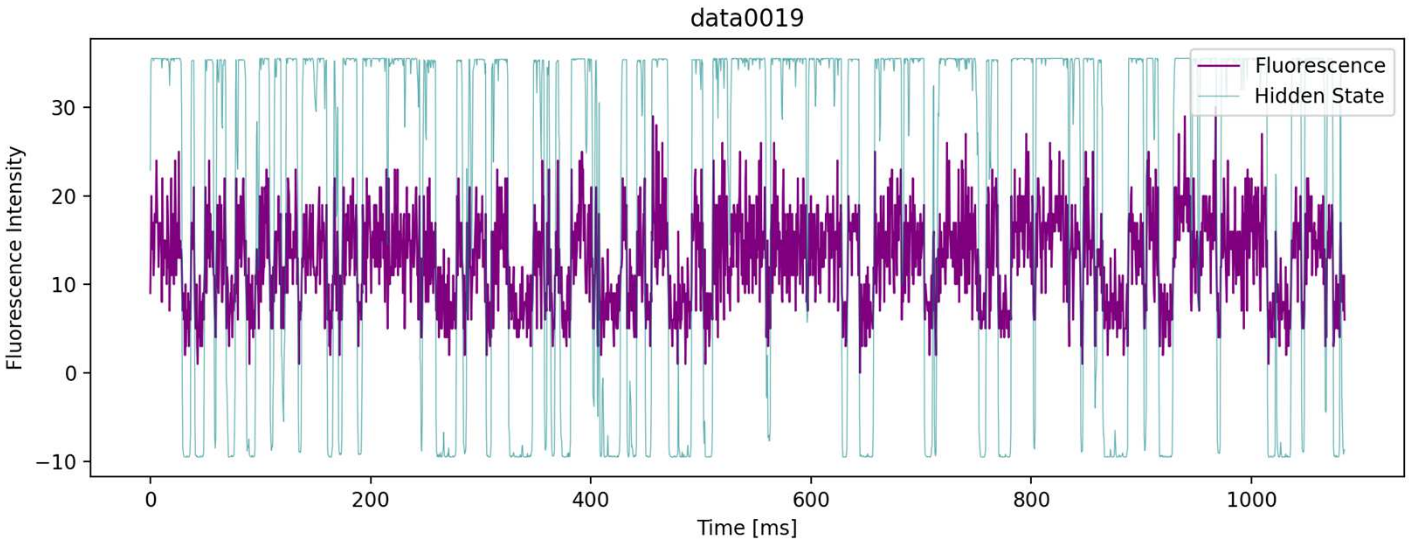
data0019
Fluorescence
Hidden State
Fluorescence Intensity
30
20
10
0
−10
0
200
400
600
800
1000
Time [ms]

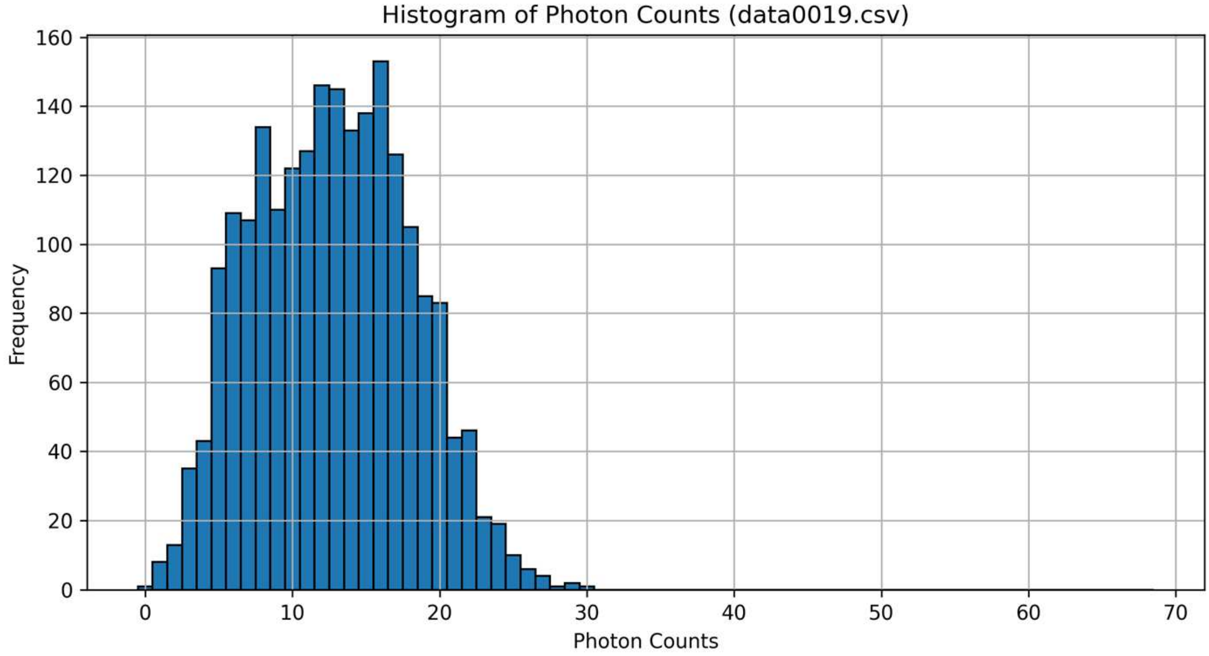
Histogram of Photon Counts (data0019.csv)
Frequency
160
140
120
100
80
60
40
20
0
0
10
20
30
40
50
60
70
Photon Counts

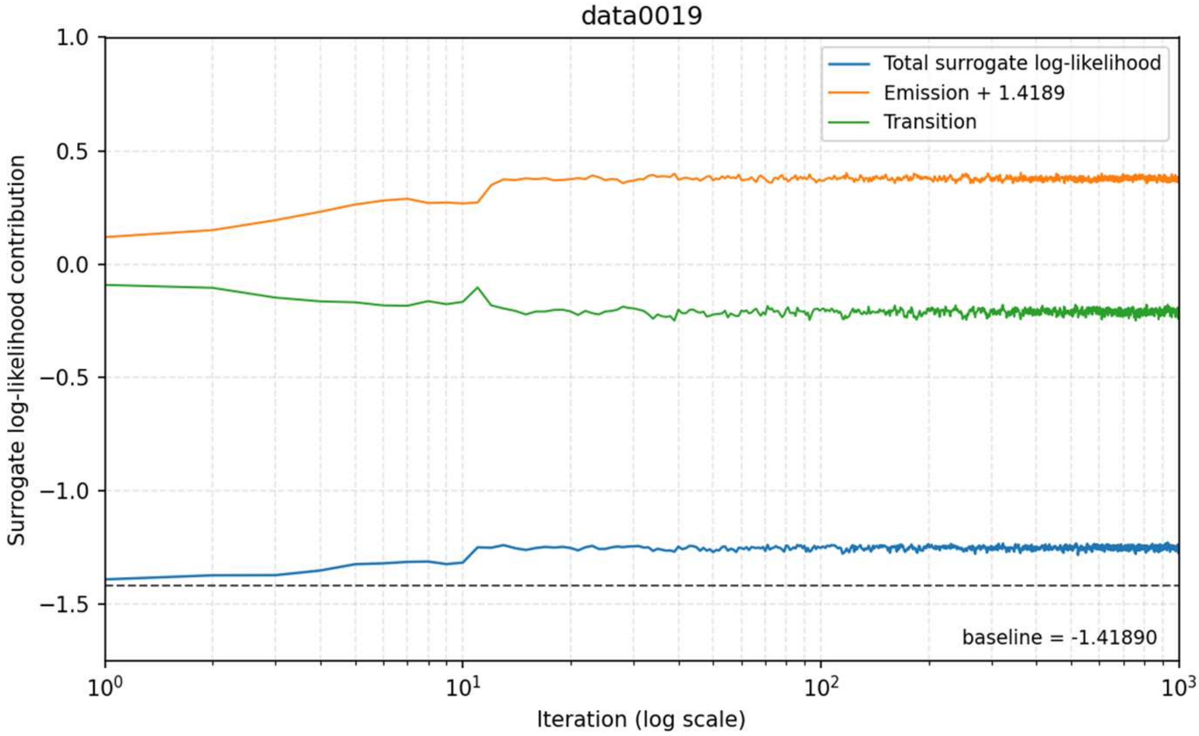
data0019
Total surrogate log-likelihood
Emission + 1.4189
Transition
Surrogate log-likelihood contribution
1.0
0.5
0.0
−0.5
−1.0
−1.5
baseline = -1.41890
10^0
10^1
10^2
10^3
Iteration (log scale)

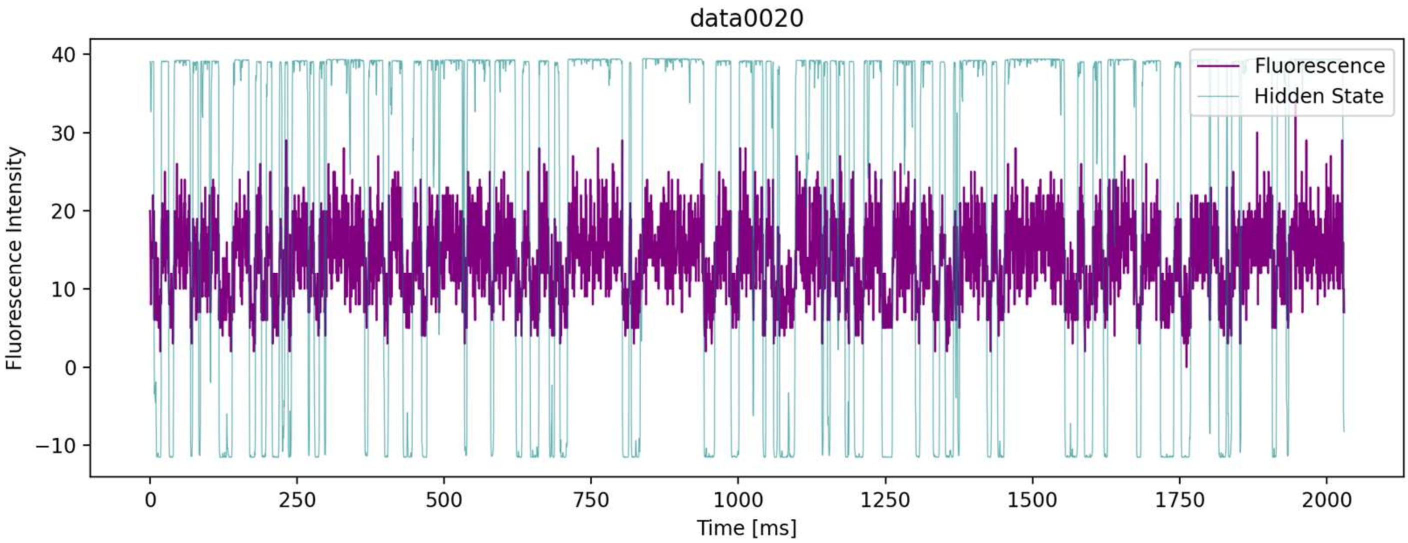
data0020
Fluorescence
Hidden State
Fluorescence Intensity
Time [ms]

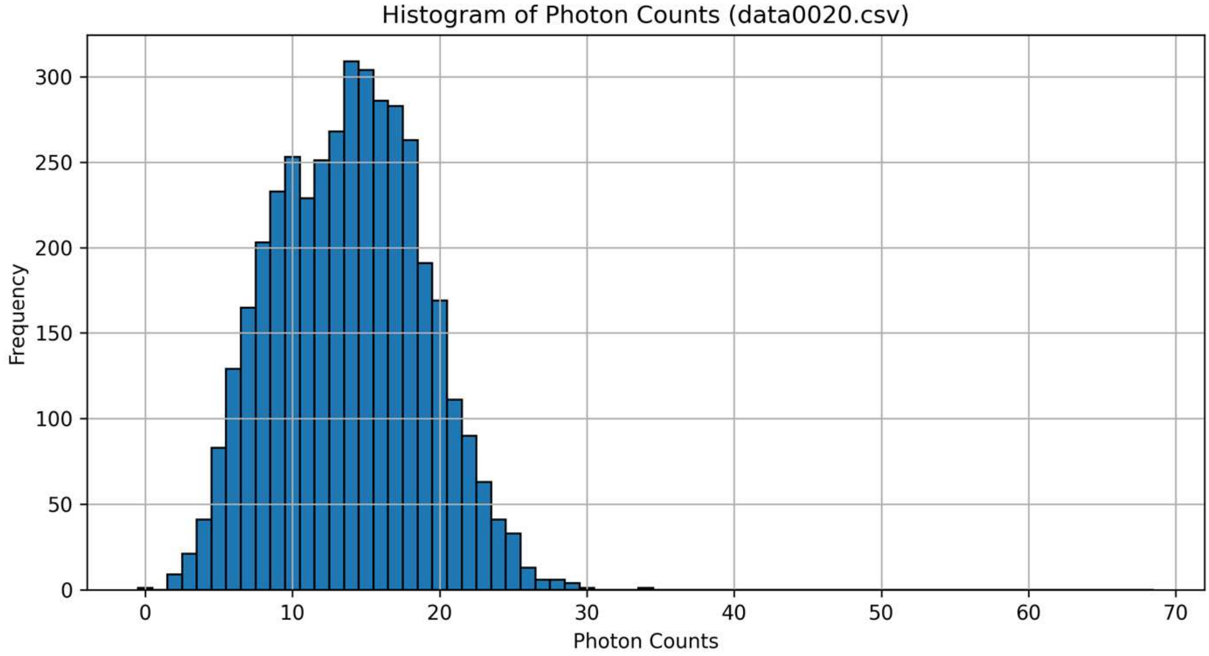
Histogram of Photon Counts (data0020.csv)
Frequency
Photon Counts

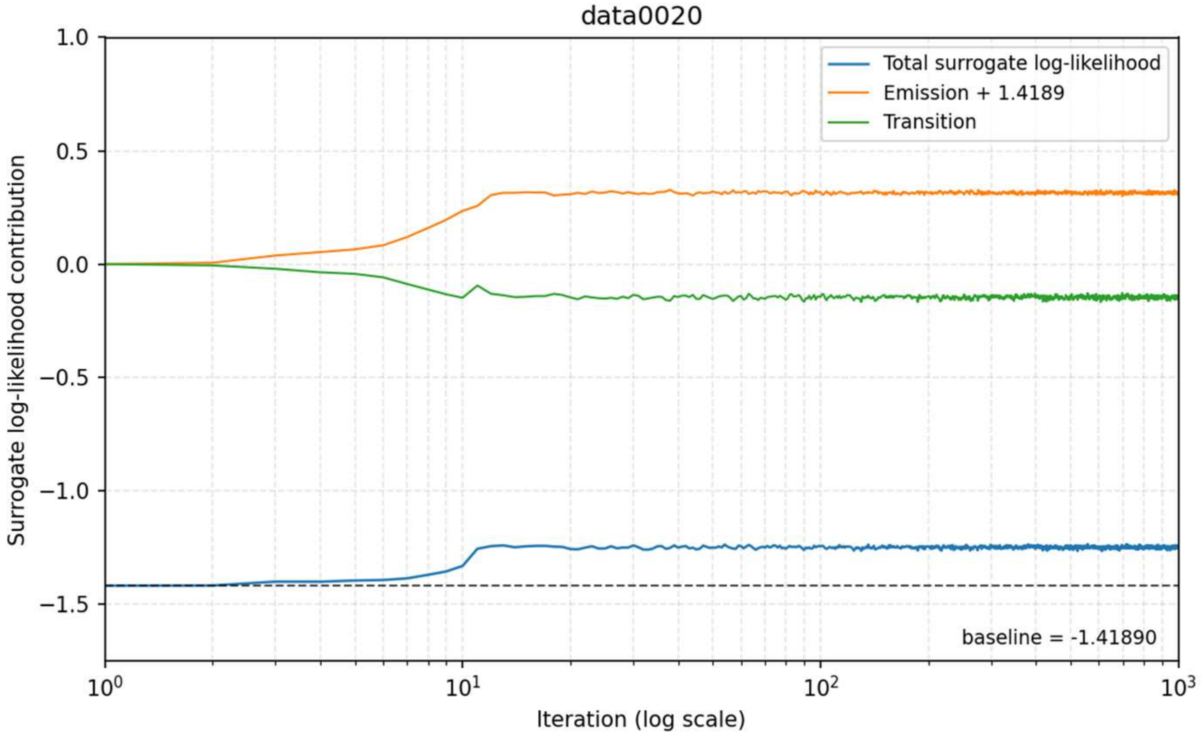
data0020
Total surrogate log-likelihood
Emission + 1.4189
Transition
Surrogate log-likelihood contribution
baseline = -1.41890
Iteration (log scale)

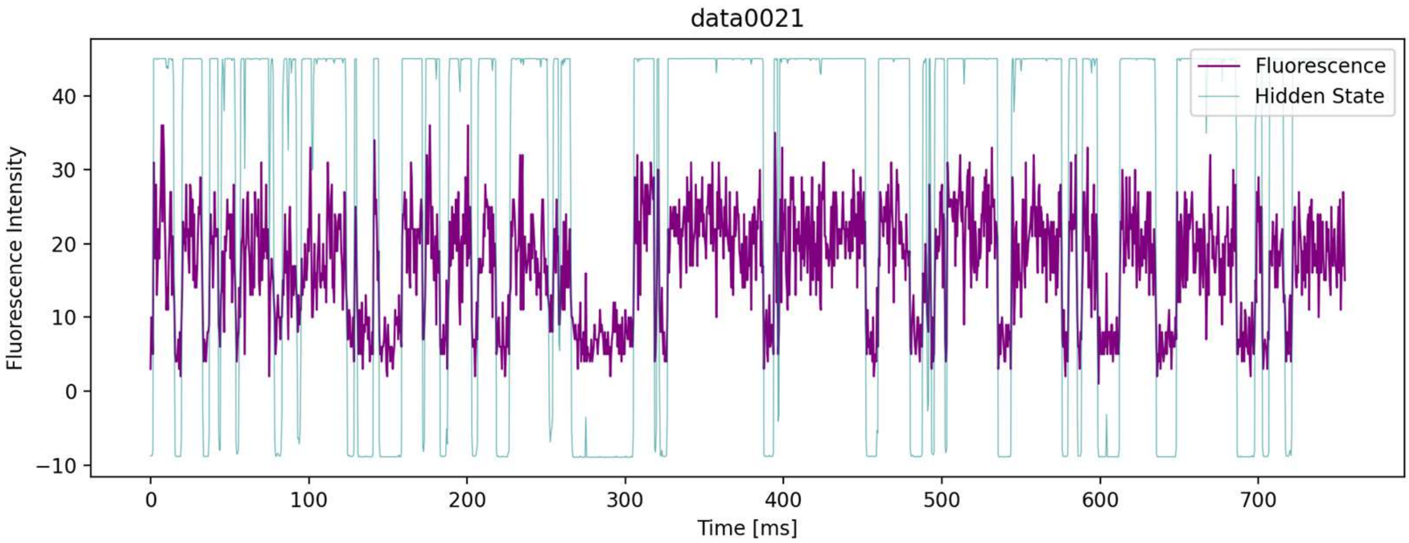
data0021
Fluorescence
Hidden State
Fluorescence Intensity
Time [ms]

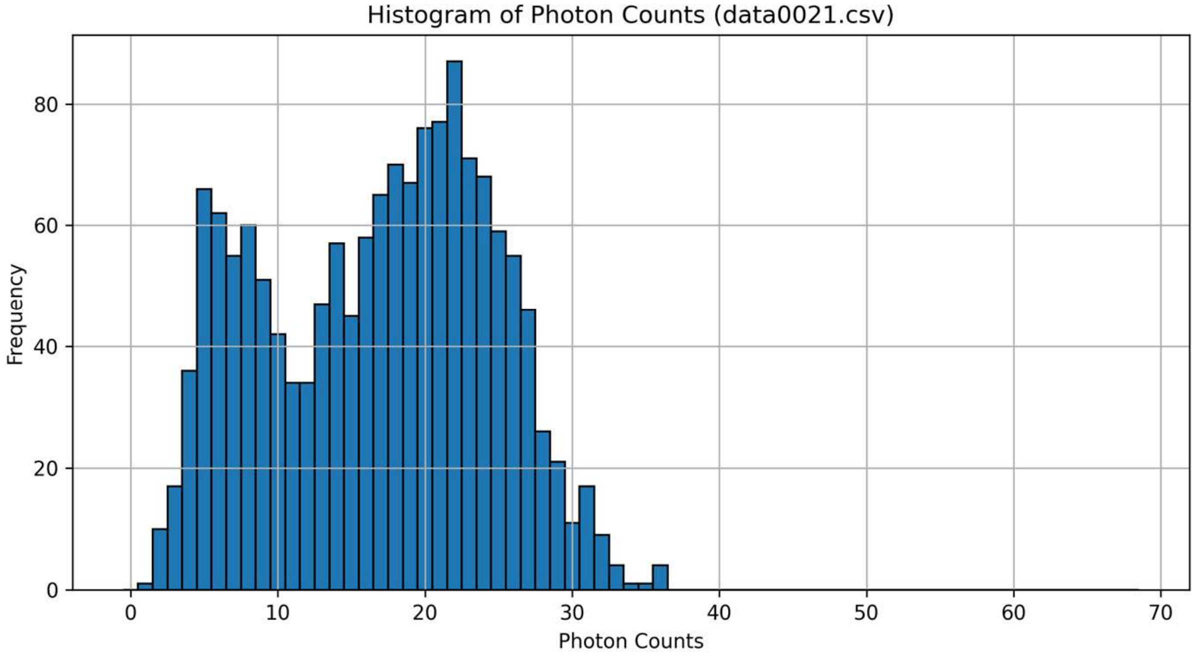
Histogram of Photon Counts (data0021.csv)
Frequency
Photon Counts

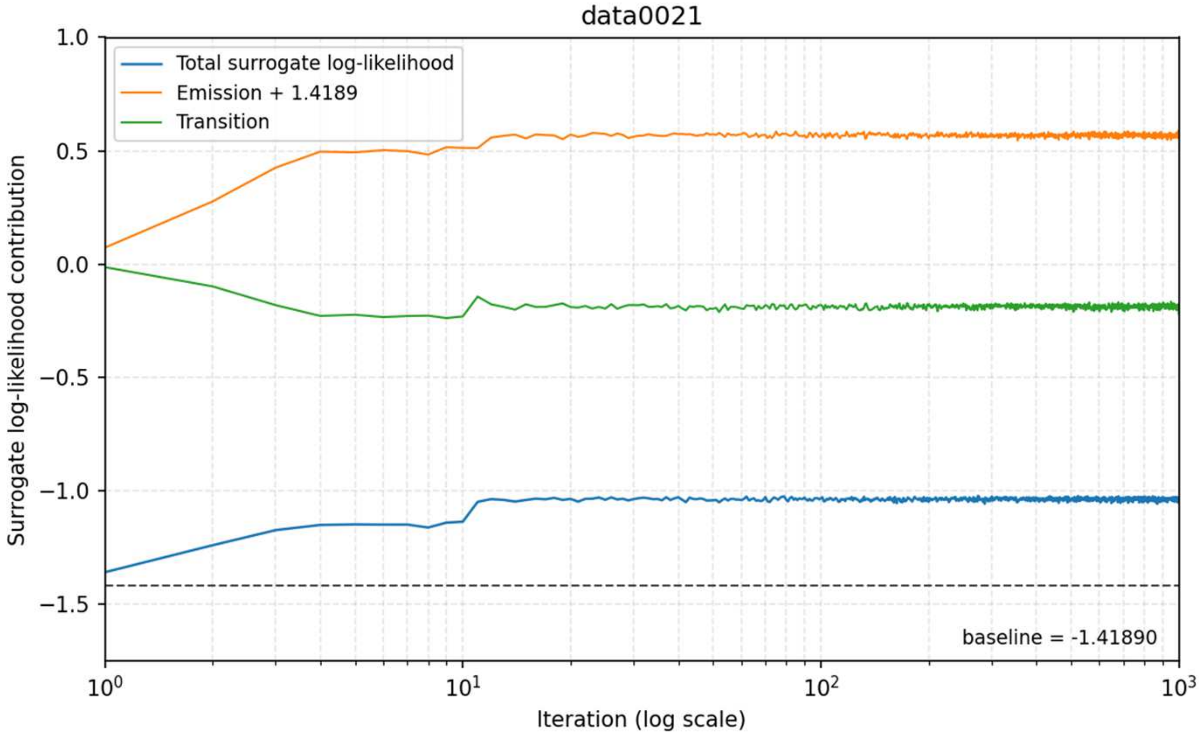
data0021
Total surrogate log-likelihood
Emission + 1.4189
Transition
Surrogate log-likelihood contribution
baseline = -1.41890
Iteration (log scale)

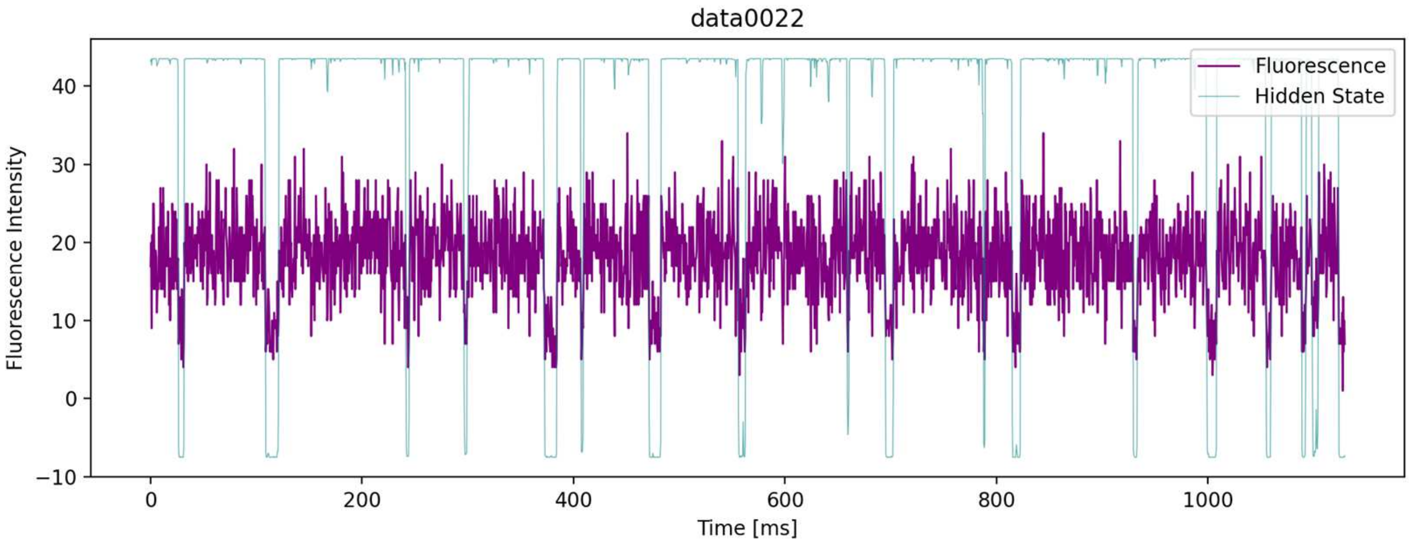
data0022
Fluorescence
Hidden State
Fluorescence Intensity
Time [ms]

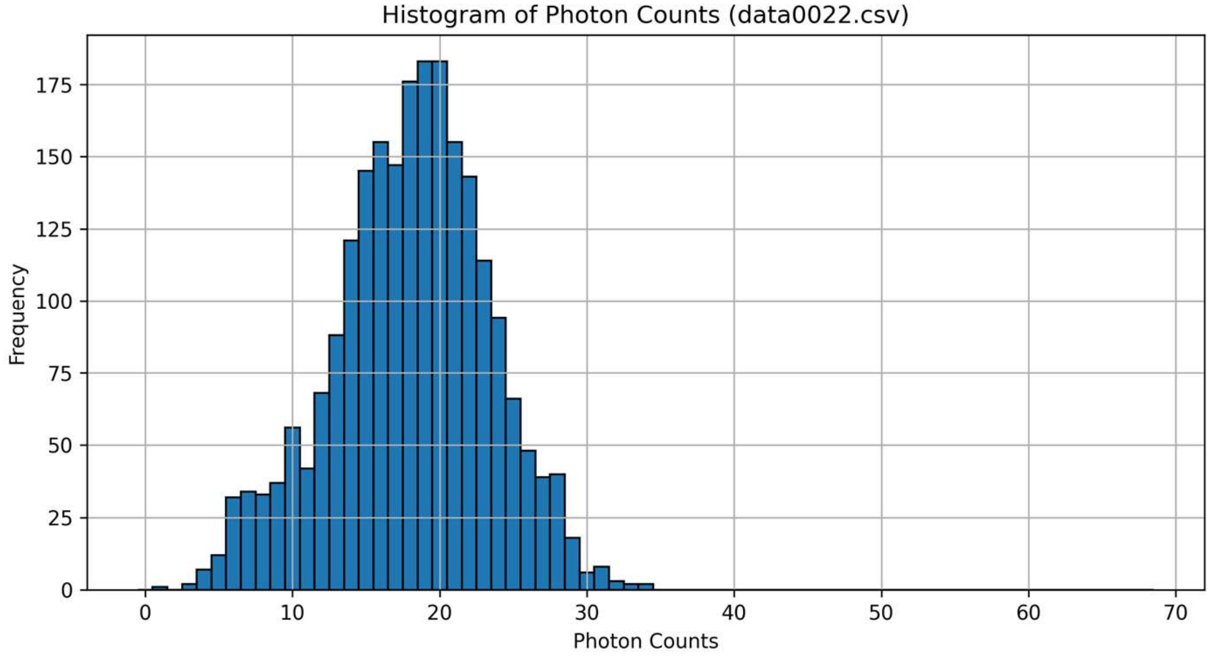
Histogram of Photon Counts (data0022.csv)
Frequency
Photon Counts

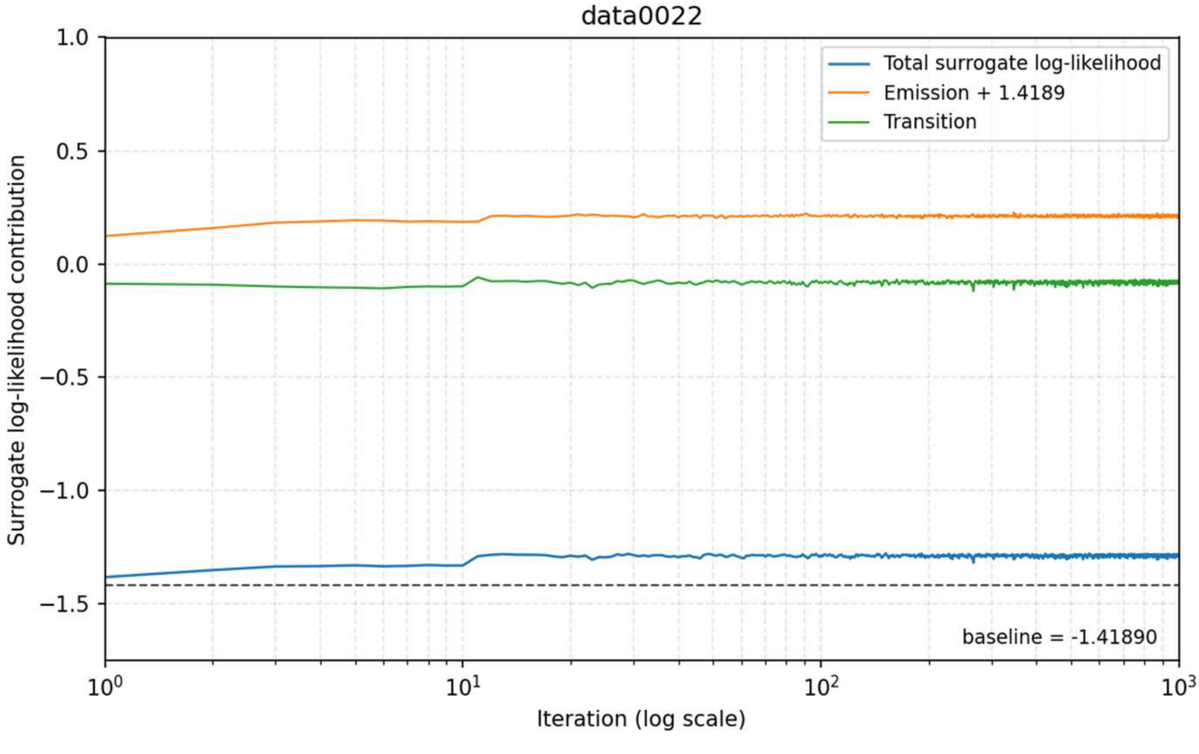
data0022
Total surrogate log-likelihood
Emission + 1.4189
Transition
Surrogate log-likelihood contribution
baseline = -1.41890
Iteration (log scale)

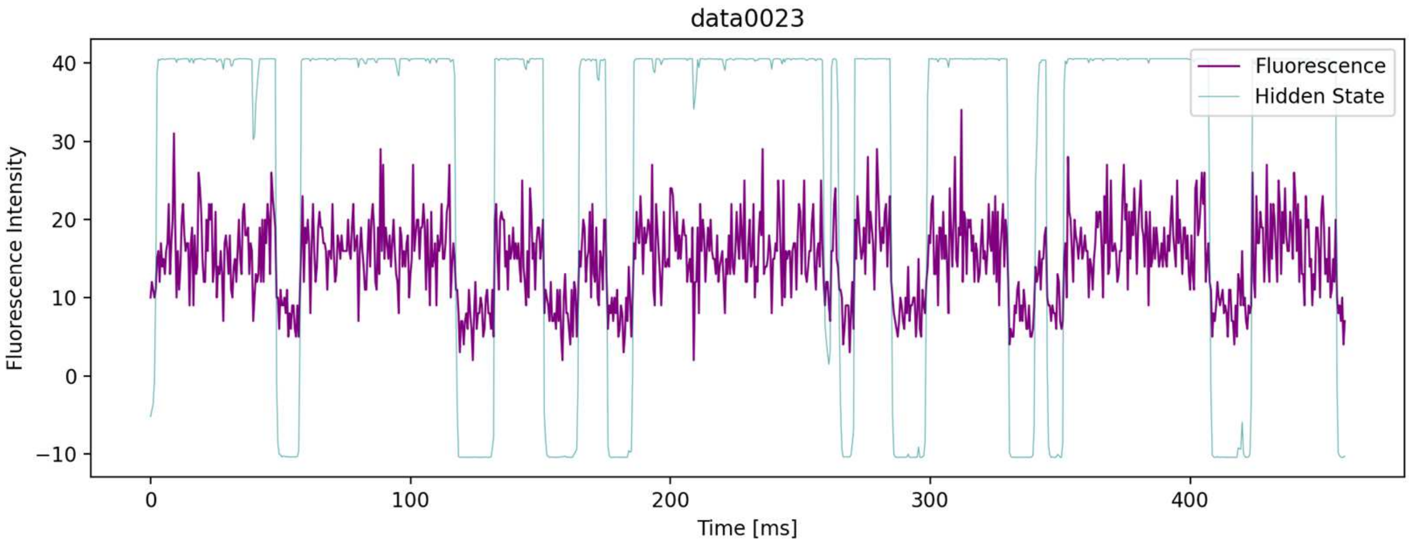
data0023
Fluorescence
Hidden State
Fluorescence Intensity
Time [ms]

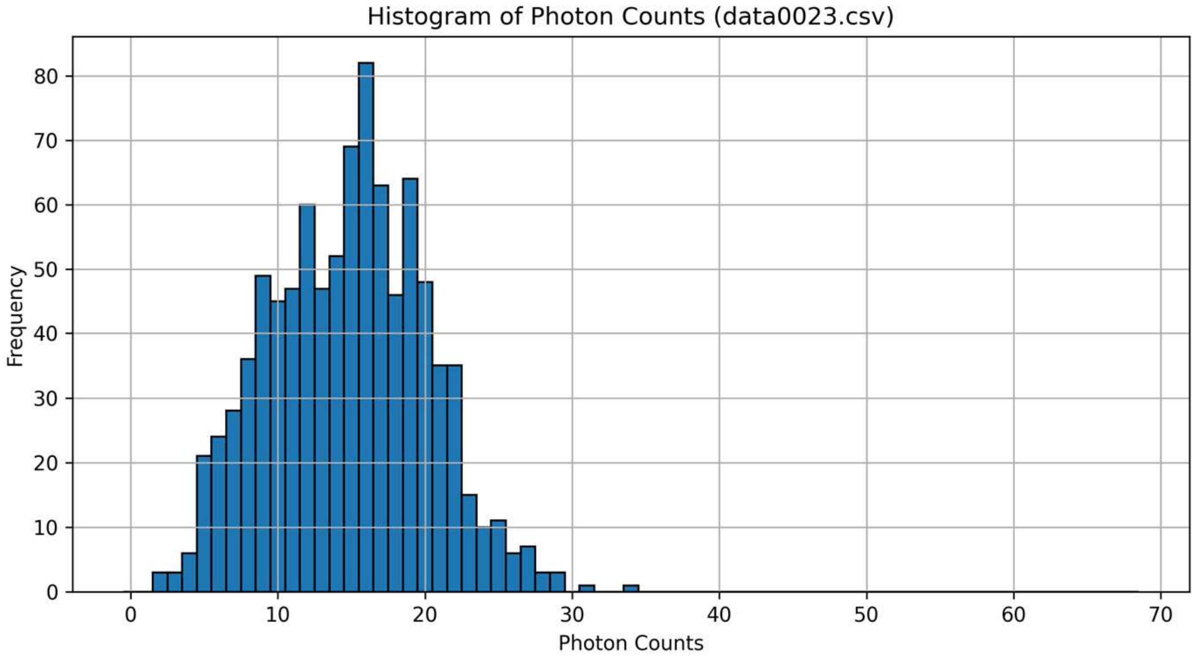
Histogram of Photon Counts (data0023.csv)
Frequency
Photon Counts

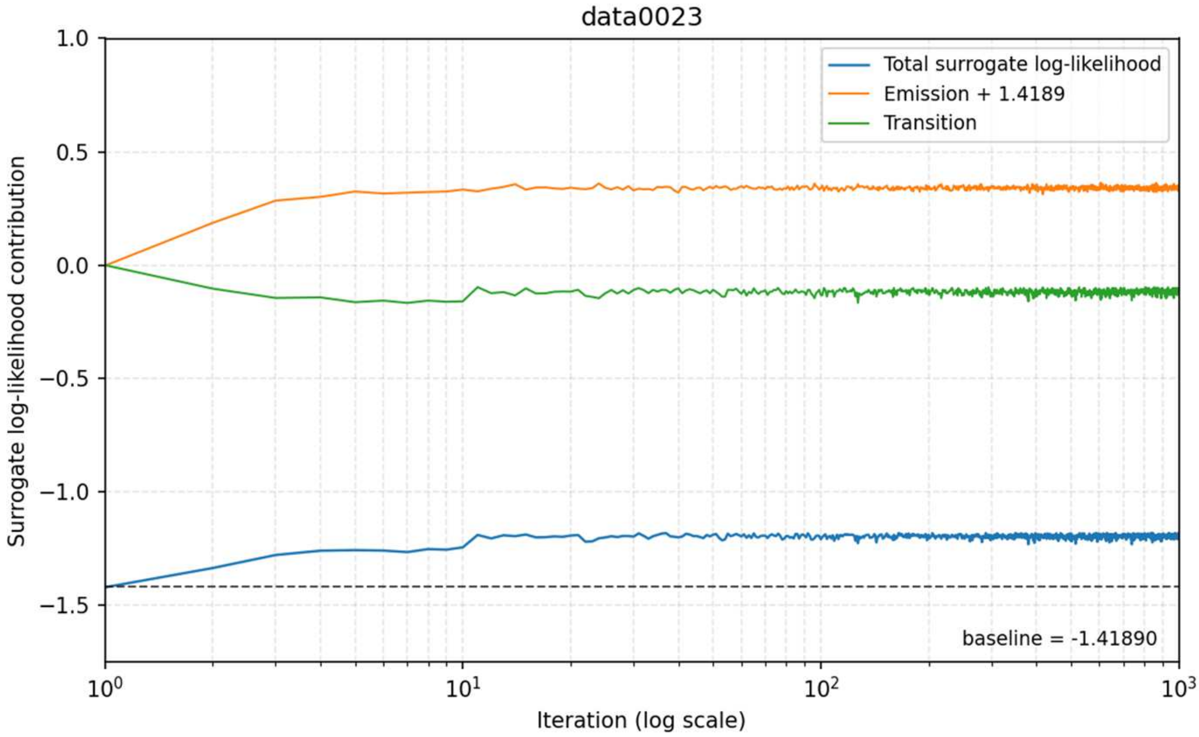
data0023
Total surrogate log-likelihood
Emission + 1.4189
Transition
Surrogate log-likelihood contribution
baseline = -1.41890
Iteration (log scale)

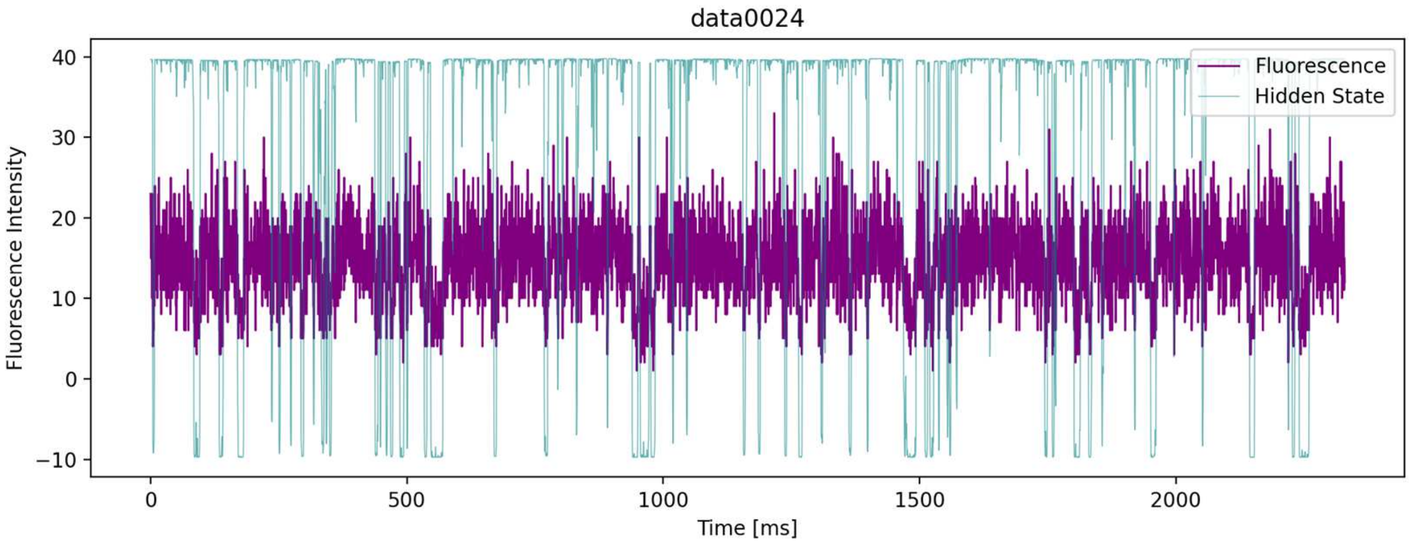
data0024
Fluorescence
Hidden State
Fluorescence Intensity
Time [ms]

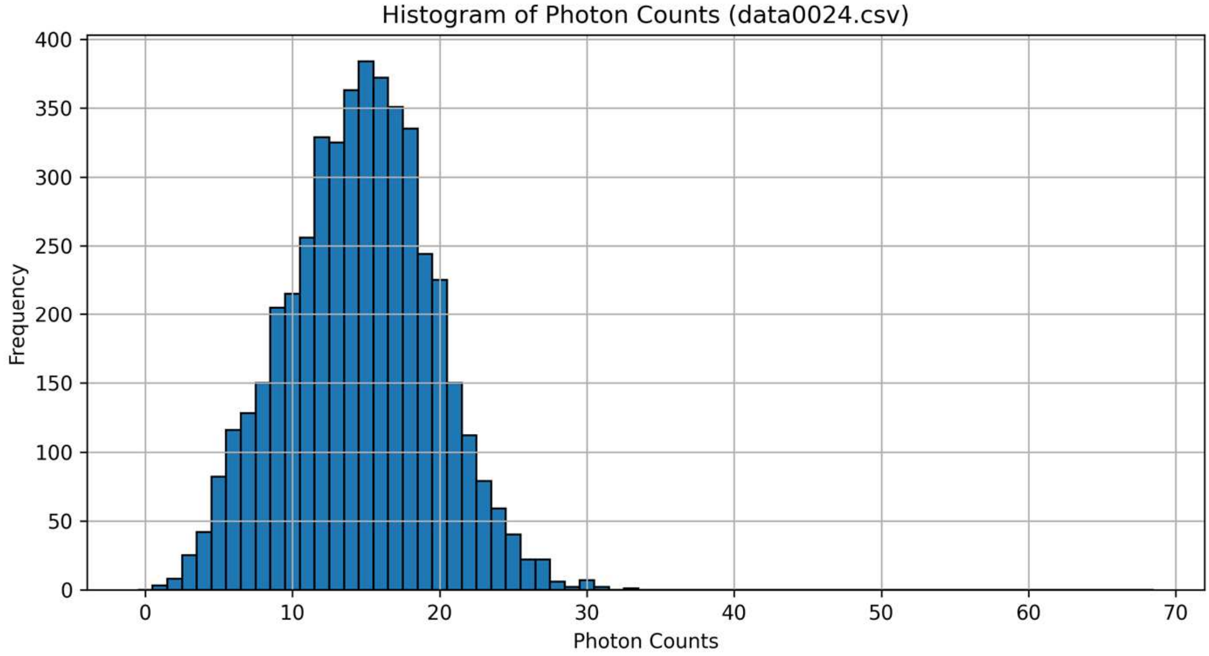
Histogram of Photon Counts (data0024.csv)
Frequency
Photon Counts

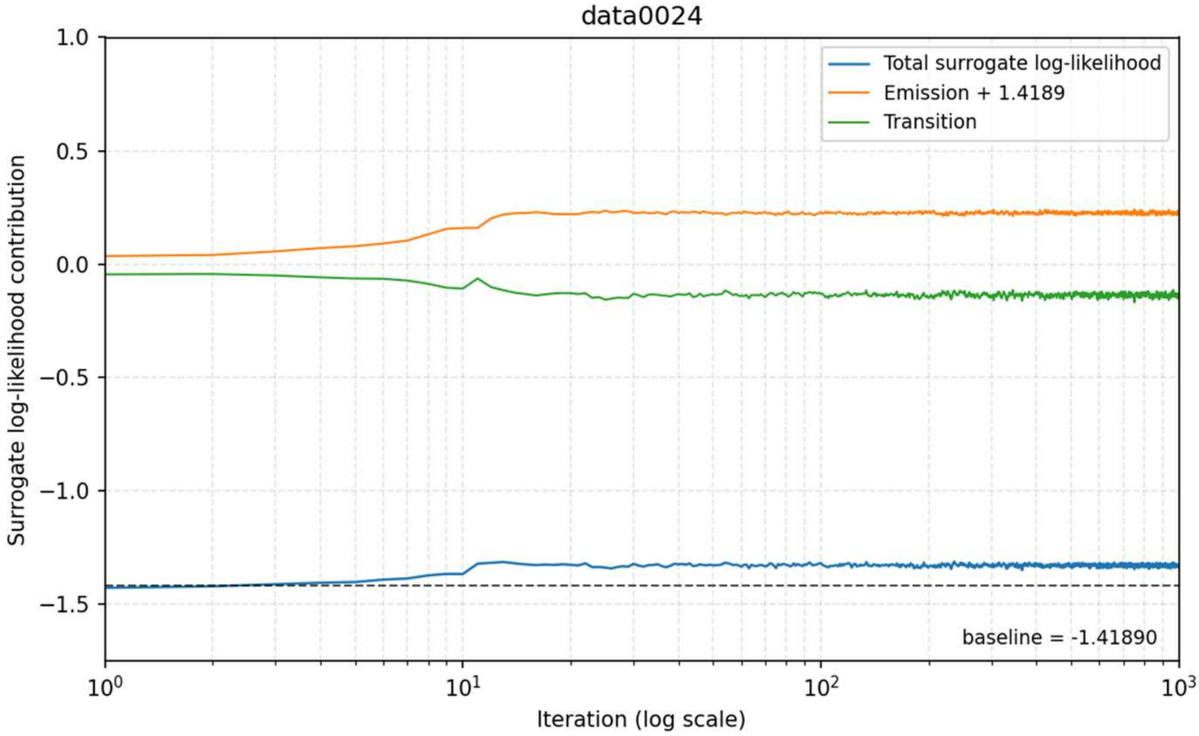
data0024
Total surrogate log-likelihood
Emission + 1.4189
Transition
Surrogate log-likelihood contribution
baseline = -1.41890
Iteration (log scale)

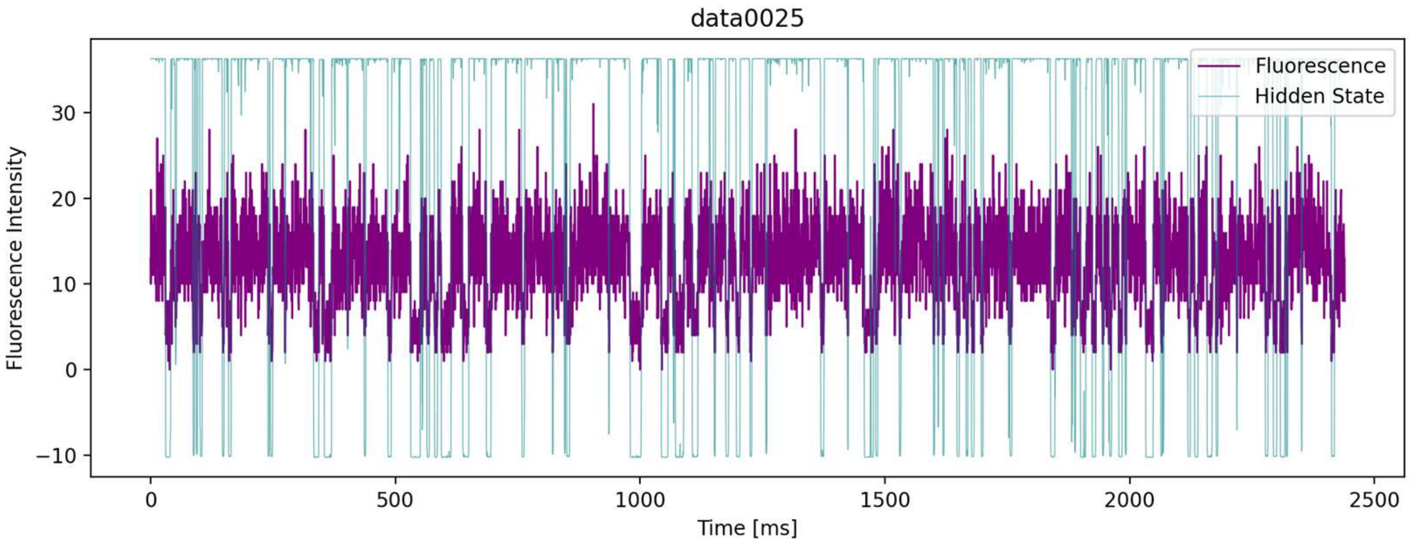
data0025
Fluorescence
Hidden State
Fluorescence Intensity
Time [ms]
30
20
10
0
−10
0
500
1000
1500
2000
2500

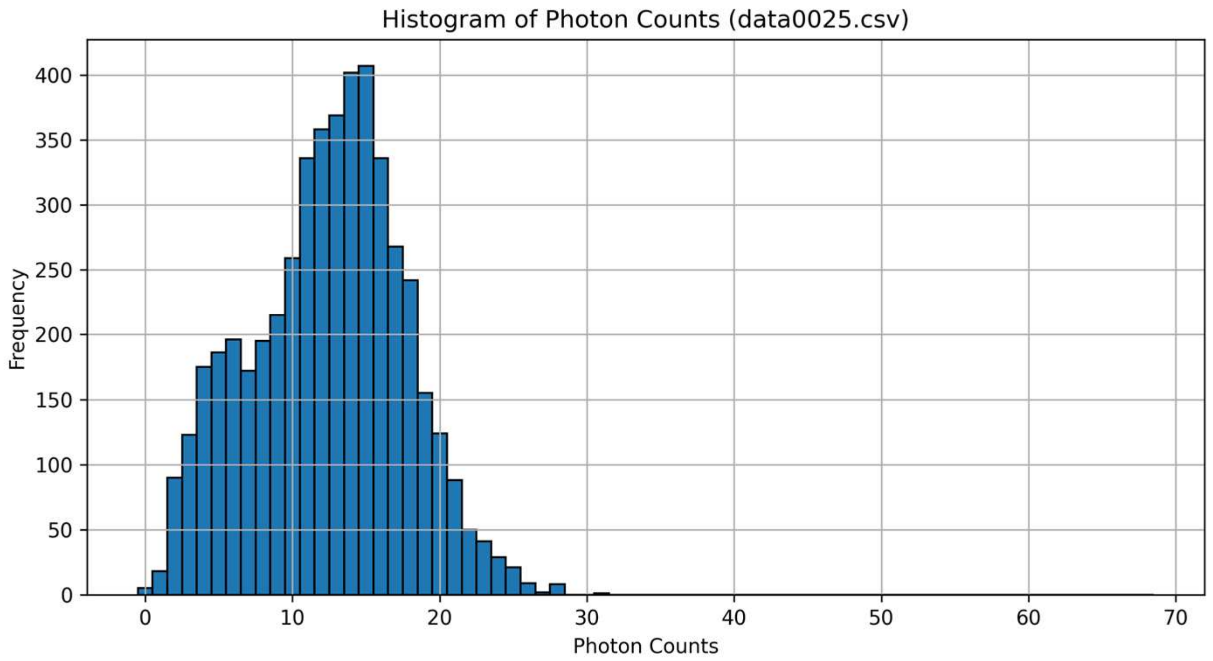
Histogram of Photon Counts (data0025.csv)
Frequency
Photon Counts
400
350
300
250
200
150
100
50
0
10
20
30
40
50
60
70

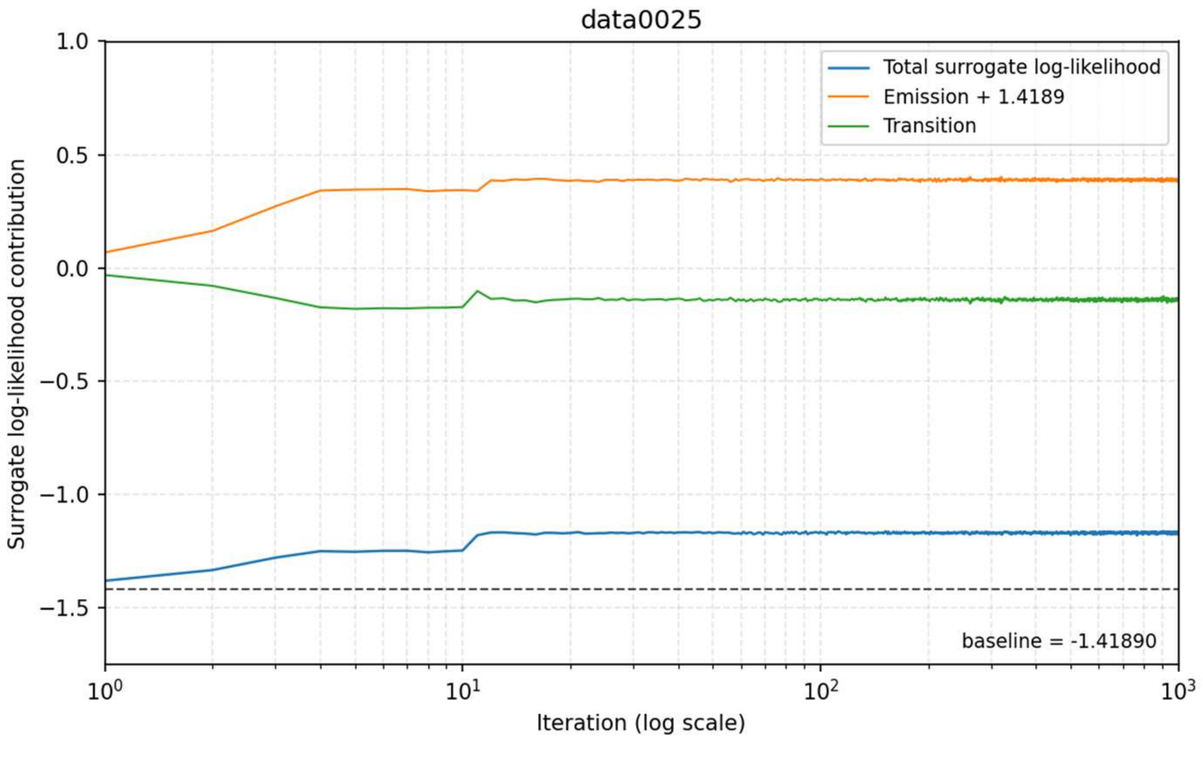
data0025
Total surrogate log-likelihood
Emission + 1.4189
Transition
Surrogate log-likelihood contribution
Iteration (log scale)
baseline = -1.41890
1.0
0.5
0.0
−0.5
−1.0
−1.5

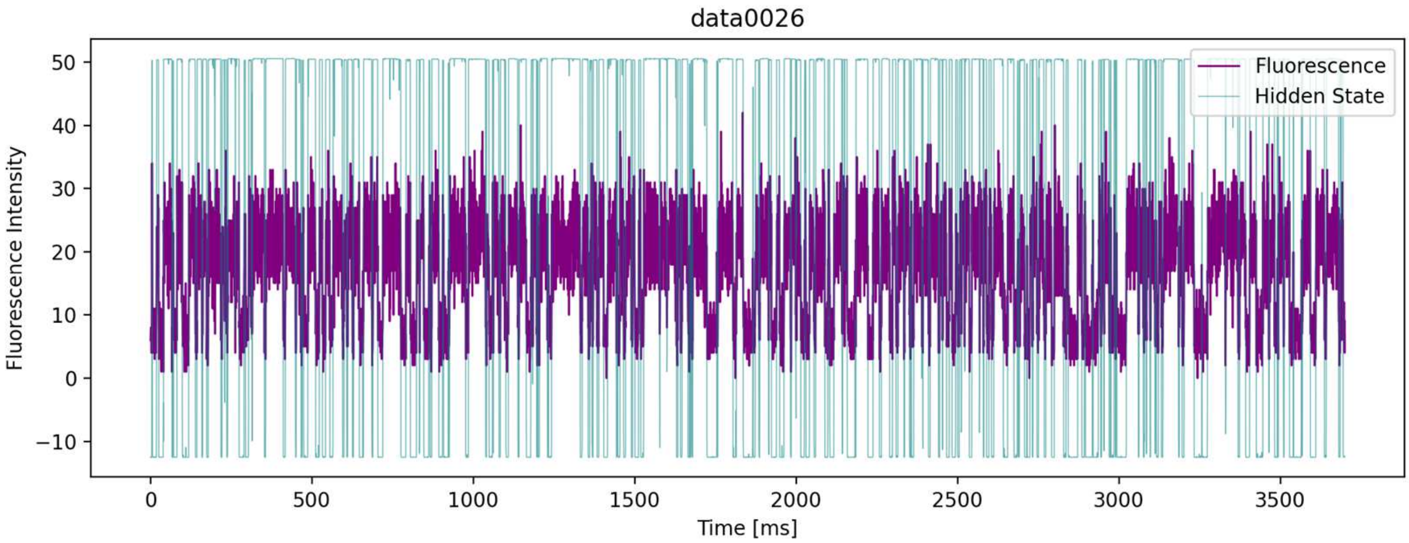
data0026
Fluorescence
Hidden State
Fluorescence Intensity
Time [ms]

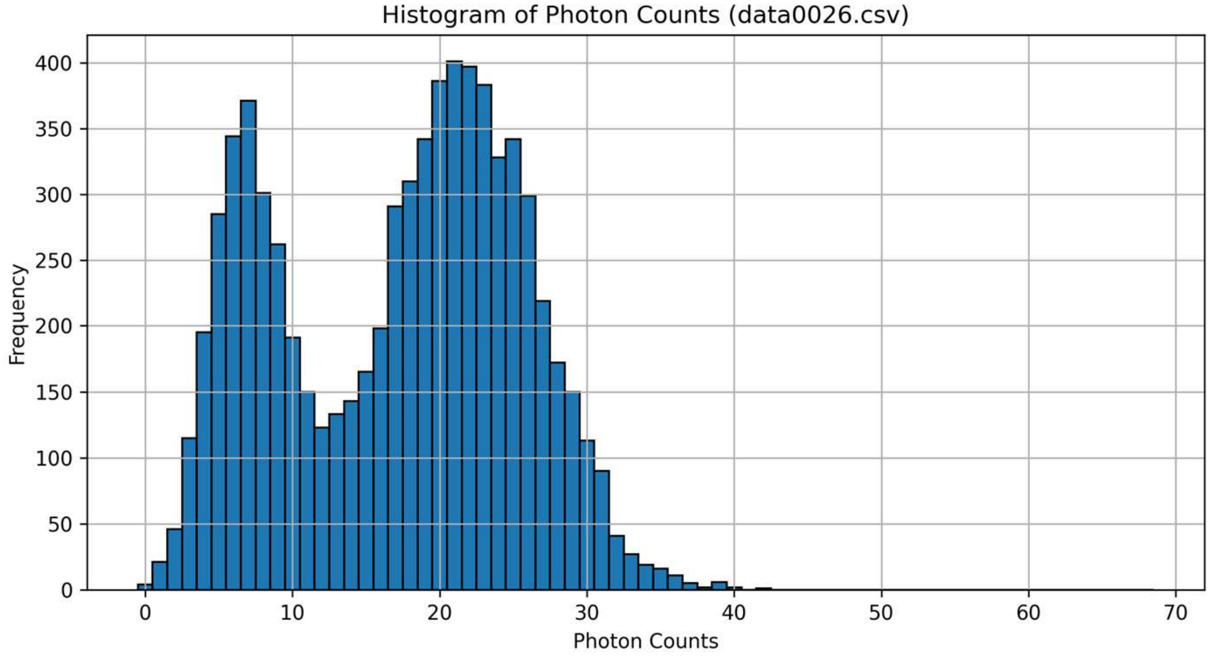
Histogram of Photon Counts (data0026.csv)
Frequency
Photon Counts

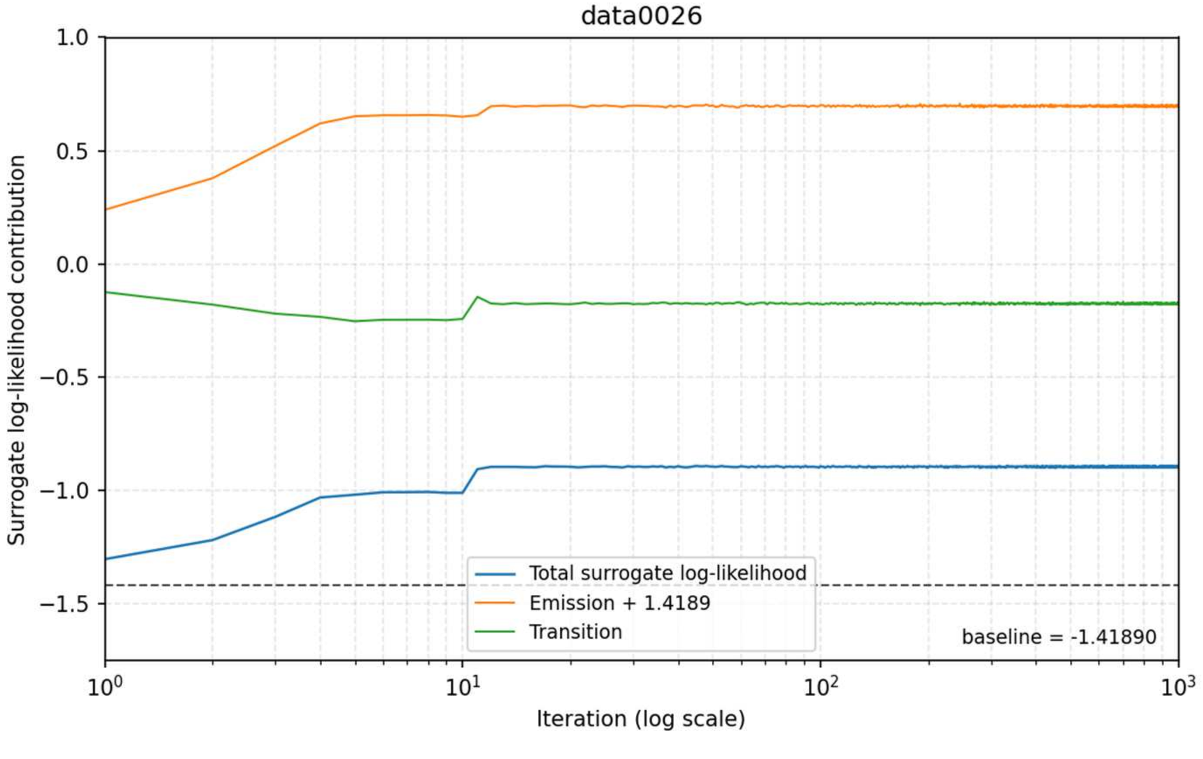
data0026
Surrogate log-likelihood contribution
Total surrogate log-likelihood
Emission + 1.4189
Transition
baseline = -1.41890
Iteration (log scale)

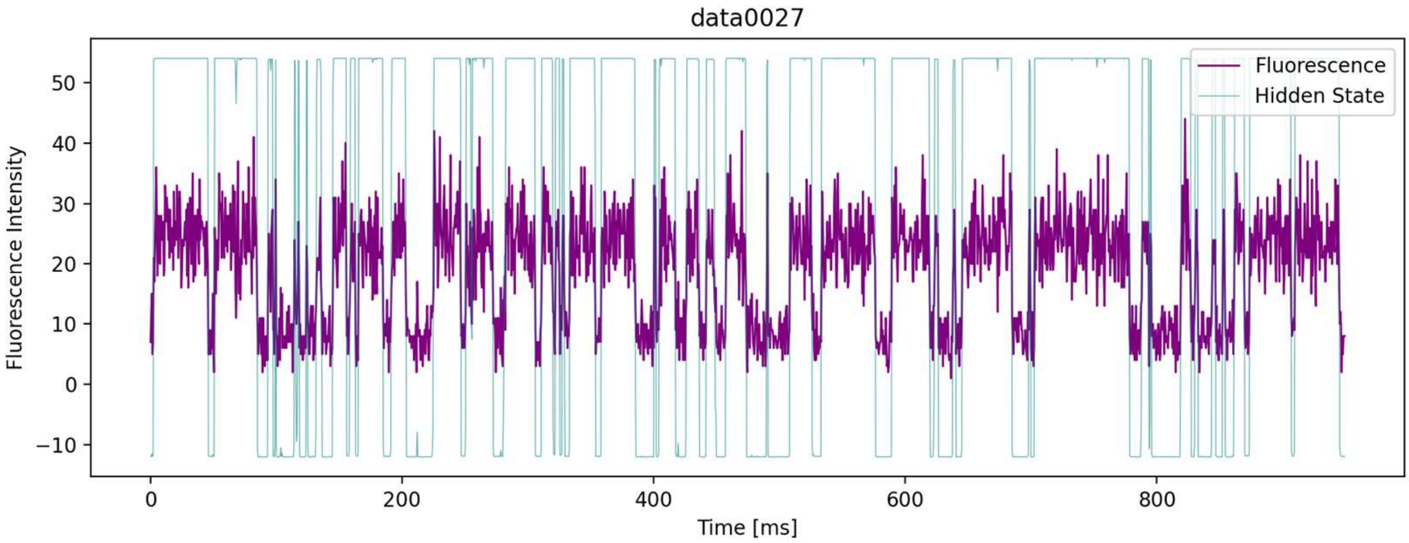
data0027
Fluorescence
Hidden State
Fluorescence Intensity
50
40
30
20
10
0
−10
0
200
400
600
800
Time [ms]

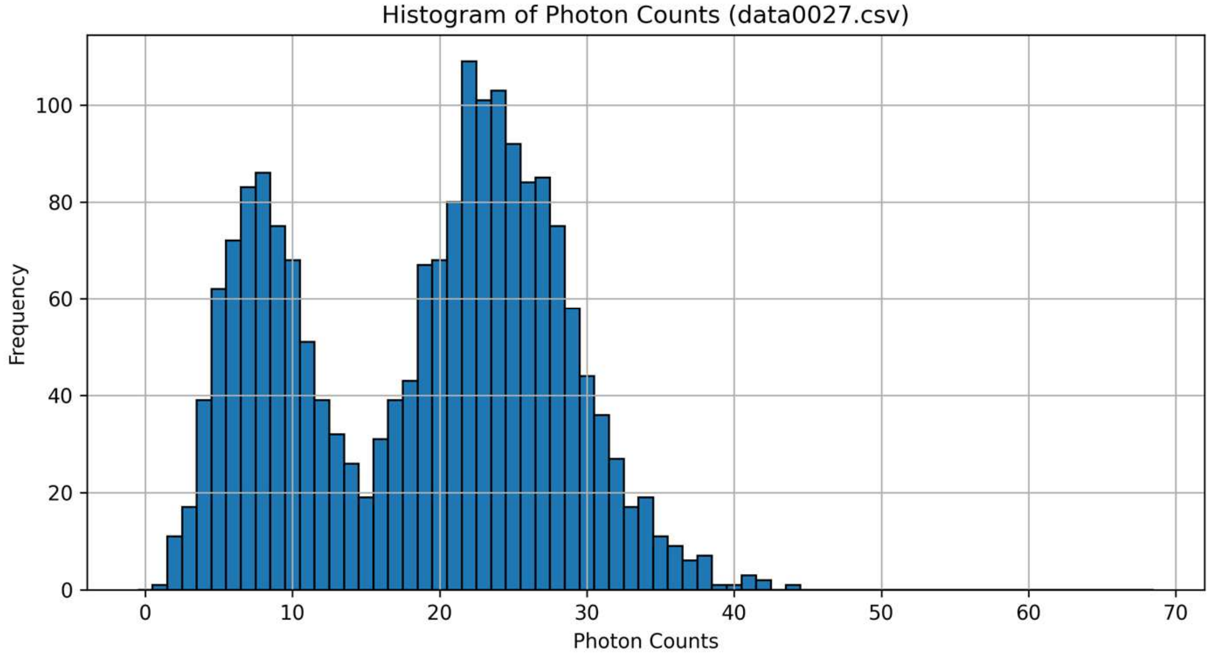
Histogram of Photon Counts (data0027.csv)
Frequency
100
80
60
40
20
0
0
10
20
30
40
50
60
70
Photon Counts

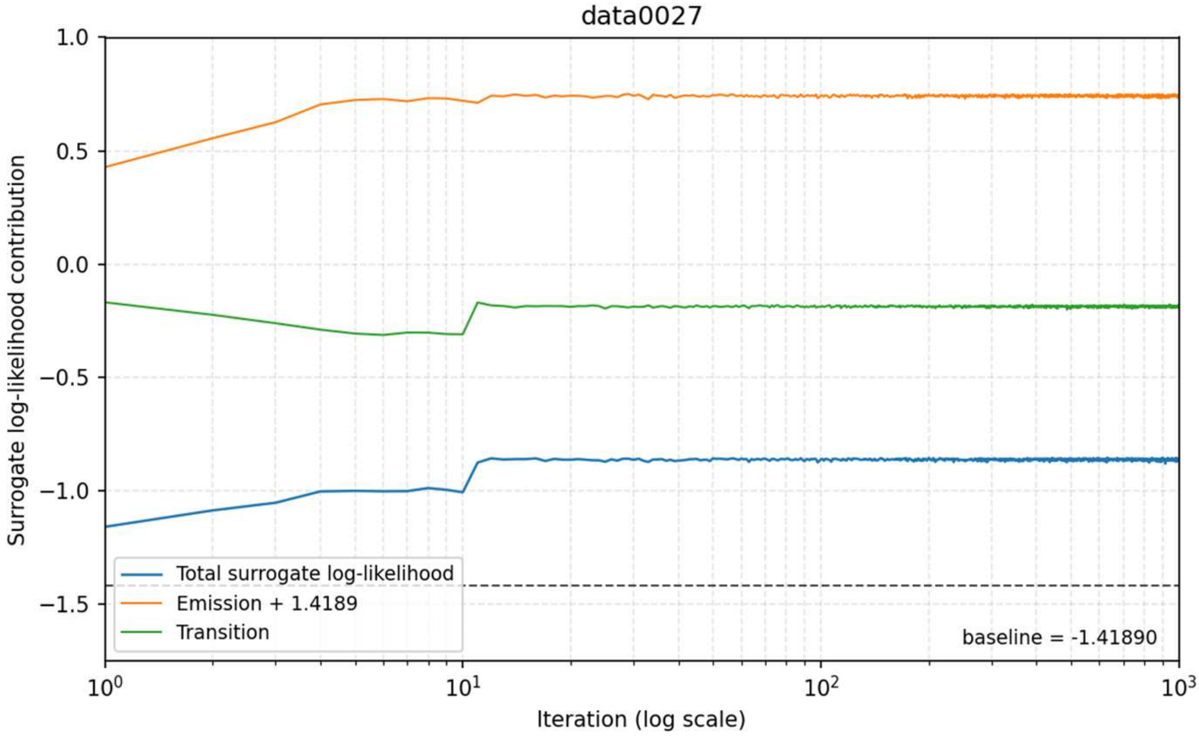
data0027
Surrogate log-likelihood contribution
1.0
0.5
0.0
−0.5
−1.0
−1.5
Total surrogate log-likelihood
Emission + 1.4189
Transition
baseline = -1.41890
10^0
10^1
10^2
10^3
Iteration (log scale)

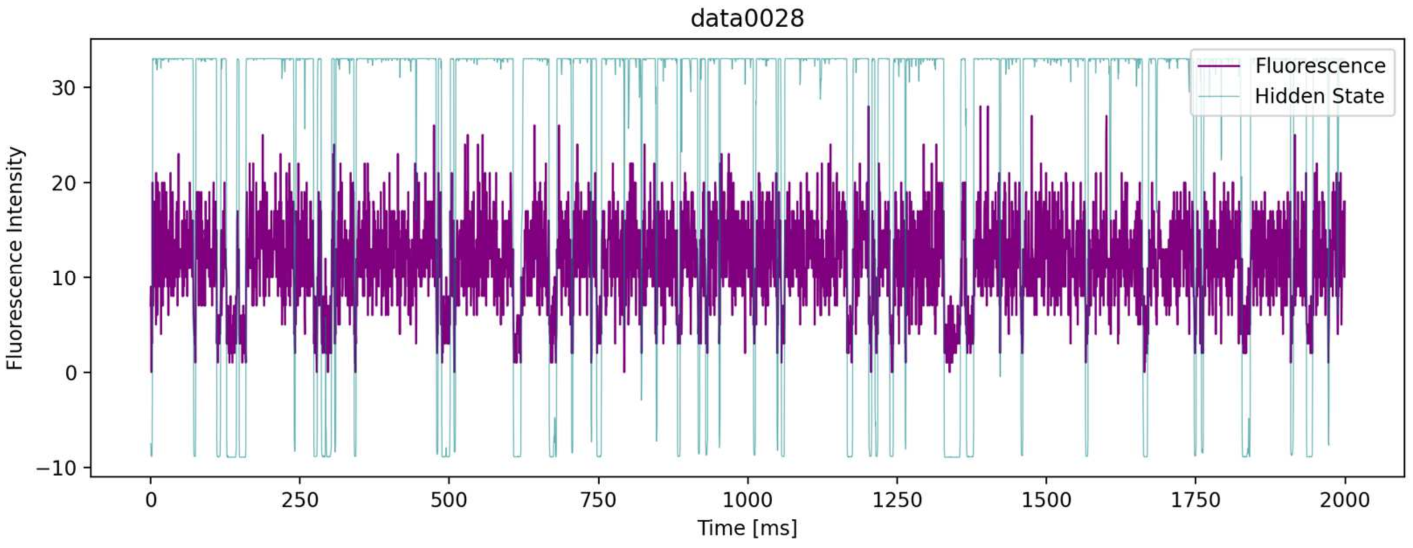
data0028
Fluorescence
Hidden State
Fluorescence Intensity
Time [ms]
30
20
10
0
−10
0
250
500
750
1000
1250
1500
1750
2000

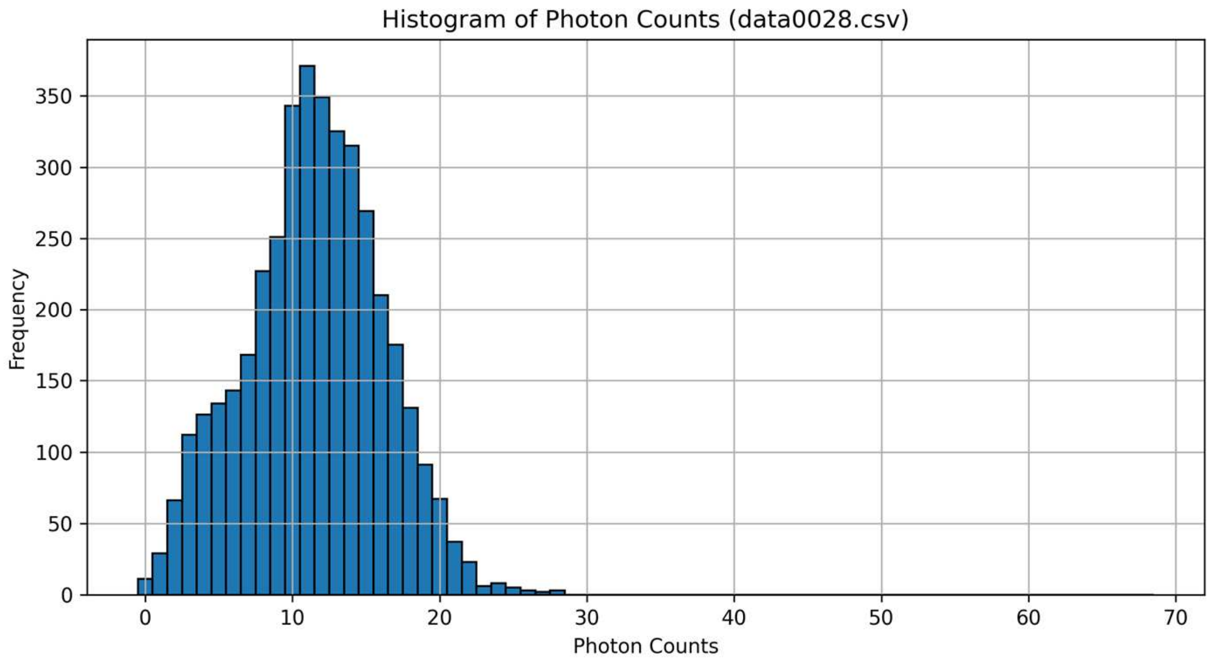
Histogram of Photon Counts (data0028.csv)
Frequency
Photon Counts
350
300
250
200
150
100
50
0
0
10
20
30
40
50
60
70

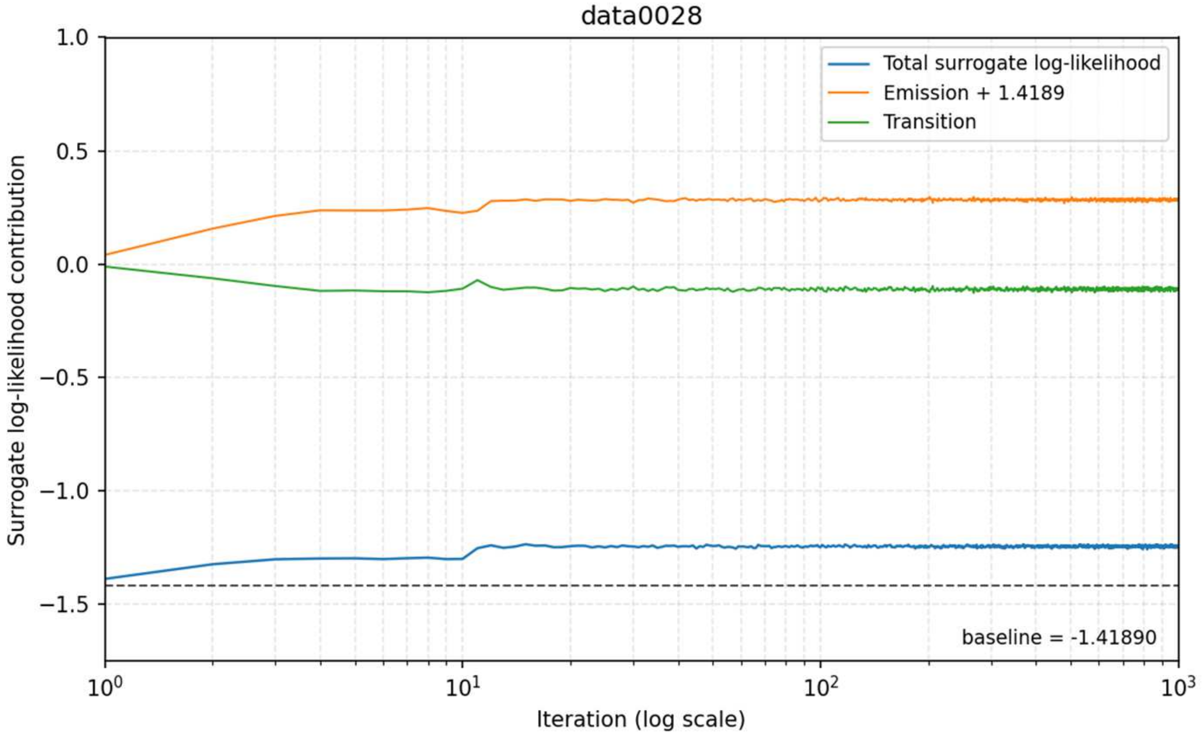
data0028
Total surrogate log-likelihood
Emission + 1.4189
Transition
Surrogate log-likelihood contribution
Iteration (log scale)
baseline = -1.41890
1.0
0.5
0.0
−0.5
−1.0
−1.5
10^0
10^1
10^2
10^3

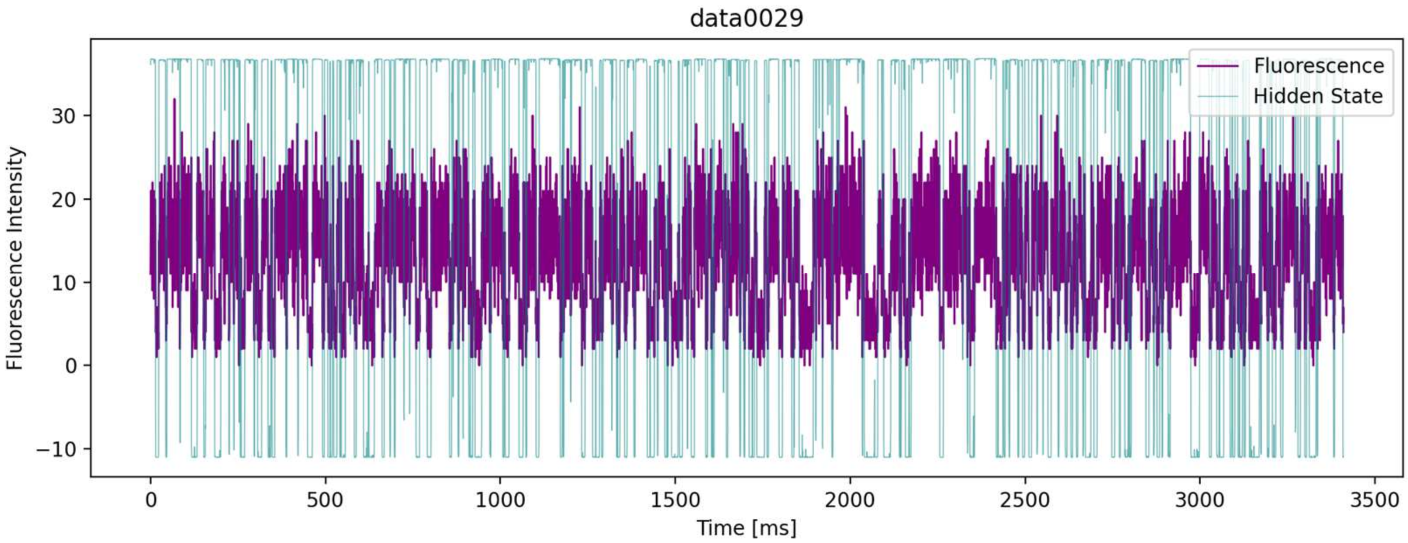
data0029
Fluorescence
Hidden State
Fluorescence Intensity
Time [ms]

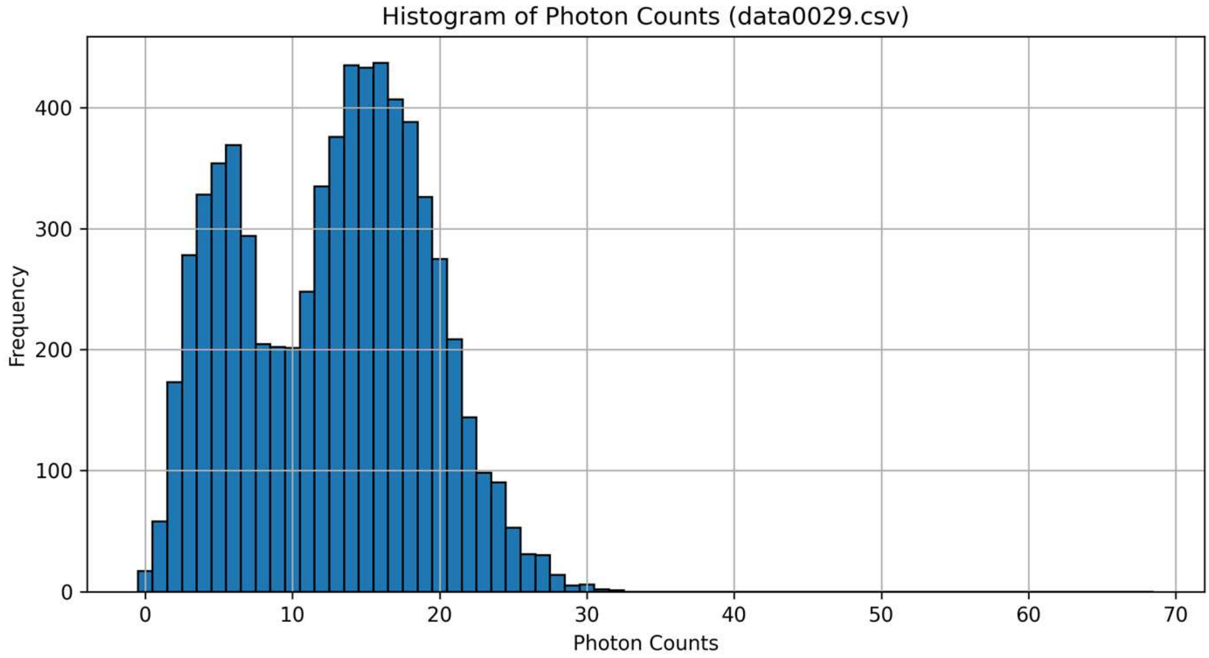
Histogram of Photon Counts (data0029.csv)
Frequency
Photon Counts

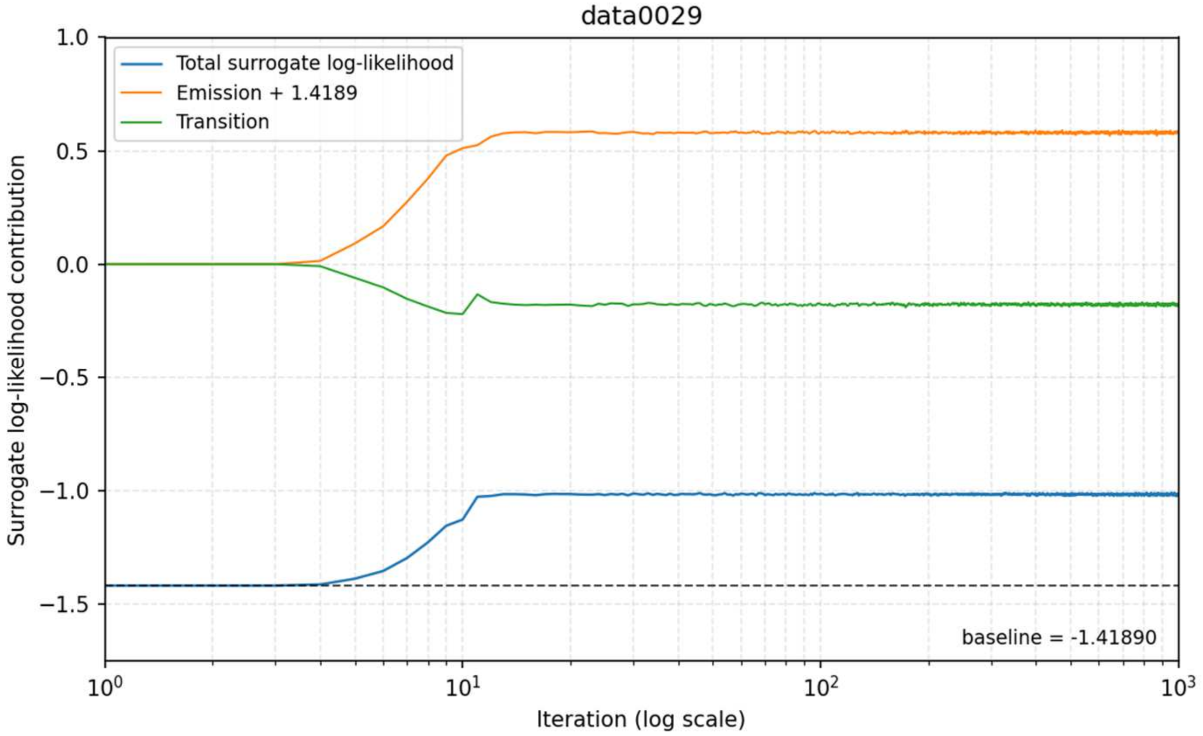
data0029
Total surrogate log-likelihood
Emission + 1.4189
Transition
Surrogate log-likelihood contribution
baseline = -1.41890
Iteration (log scale)

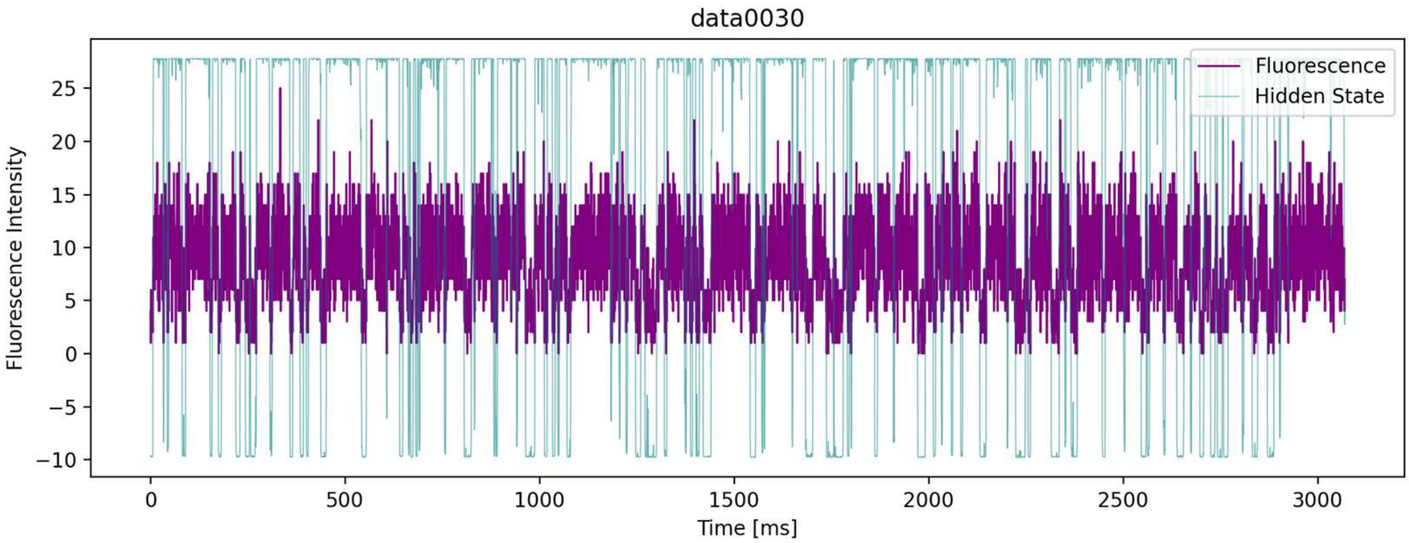
data0030
Fluorescence
Hidden State
Fluorescence Intensity
Time [ms]

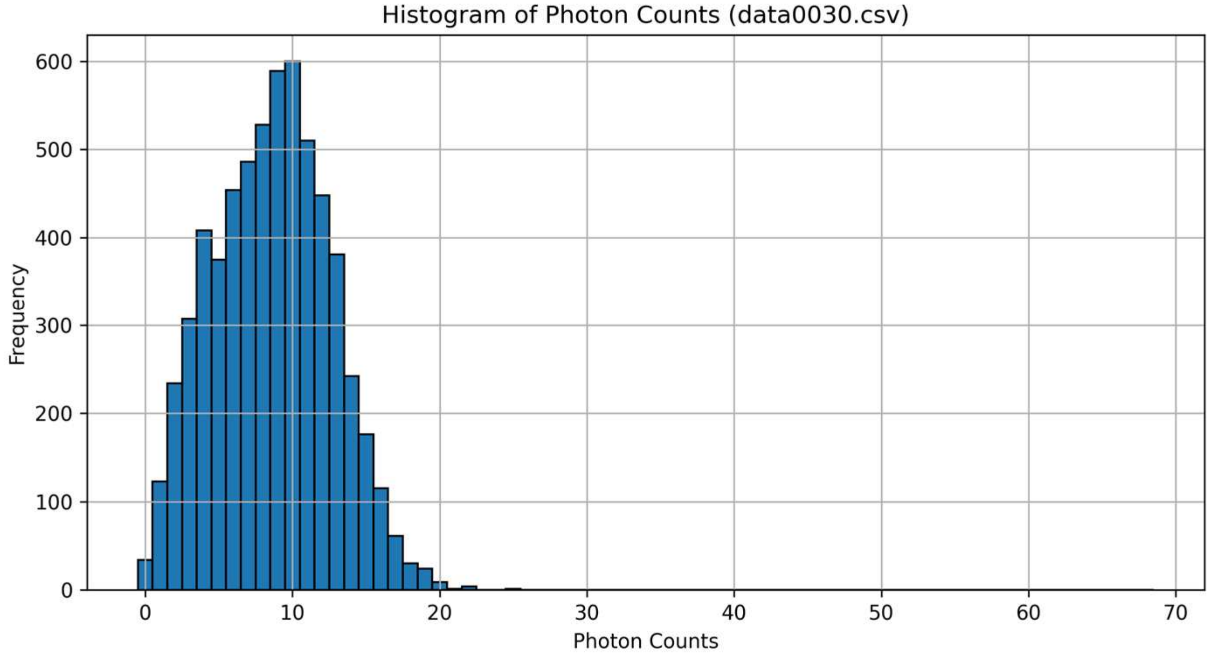
Histogram of Photon Counts (data0030.csv)
Frequency
Photon Counts

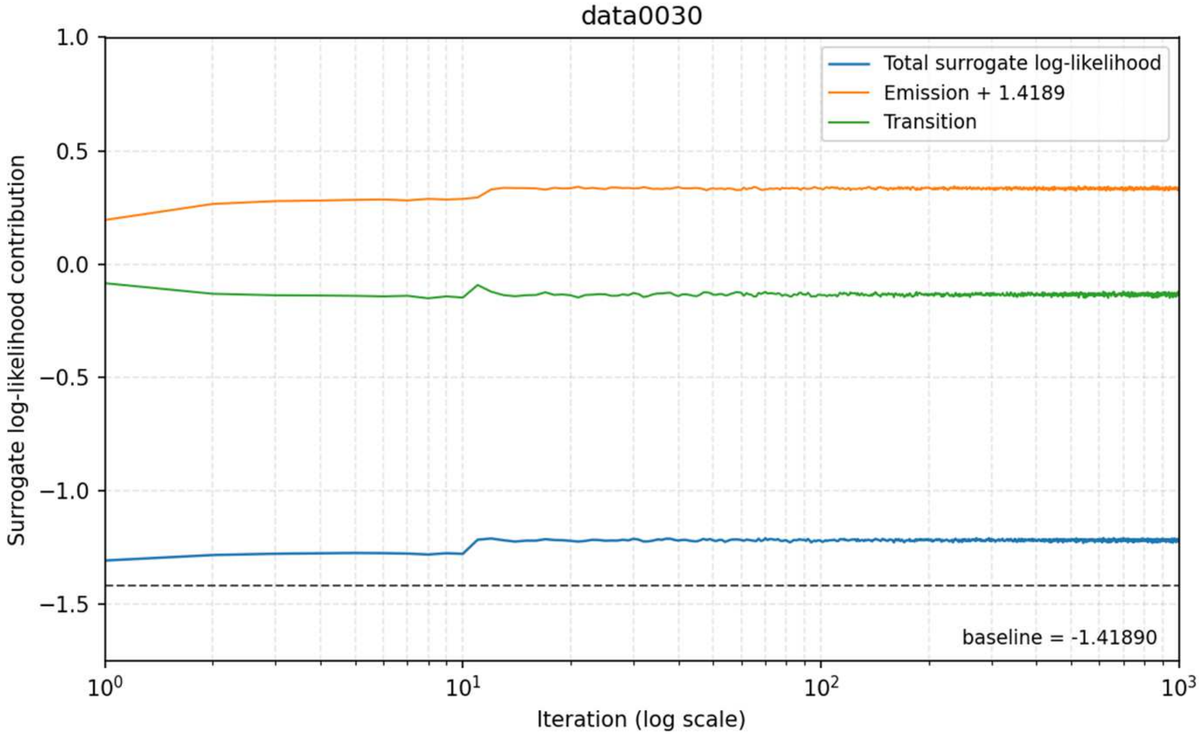
data0030
Total surrogate log-likelihood
Emission + 1.4189
Transition
Surrogate log-likelihood contribution
baseline = -1.41890
Iteration (log scale)

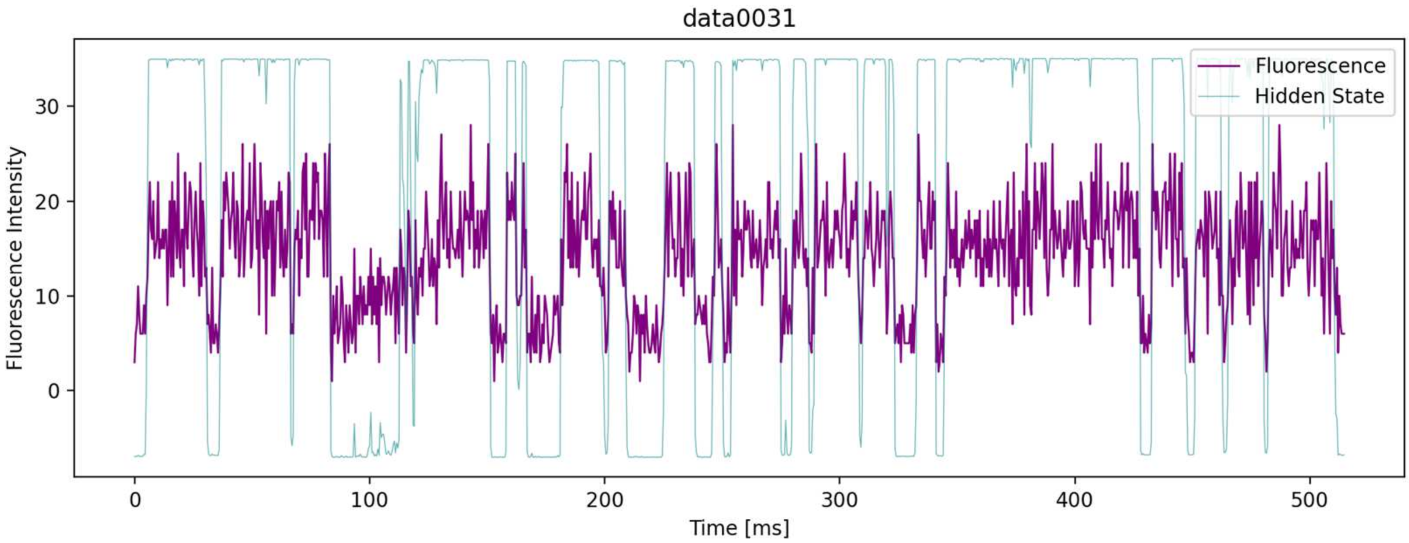
data0031
Fluorescence
Hidden State
Fluorescence Intensity
Time [ms]
0
100
200
300
400
500
0
10
20
30

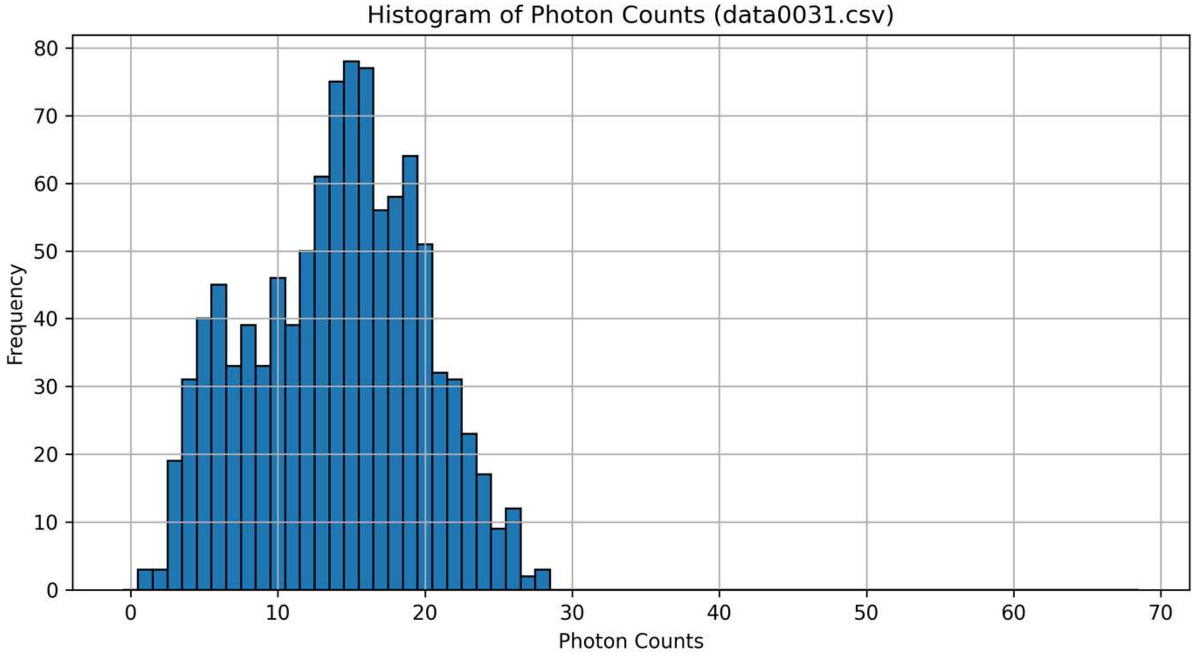
Histogram of Photon Counts (data0031.csv)
Frequency
Photon Counts
0
10
20
30
40
50
60
70
80

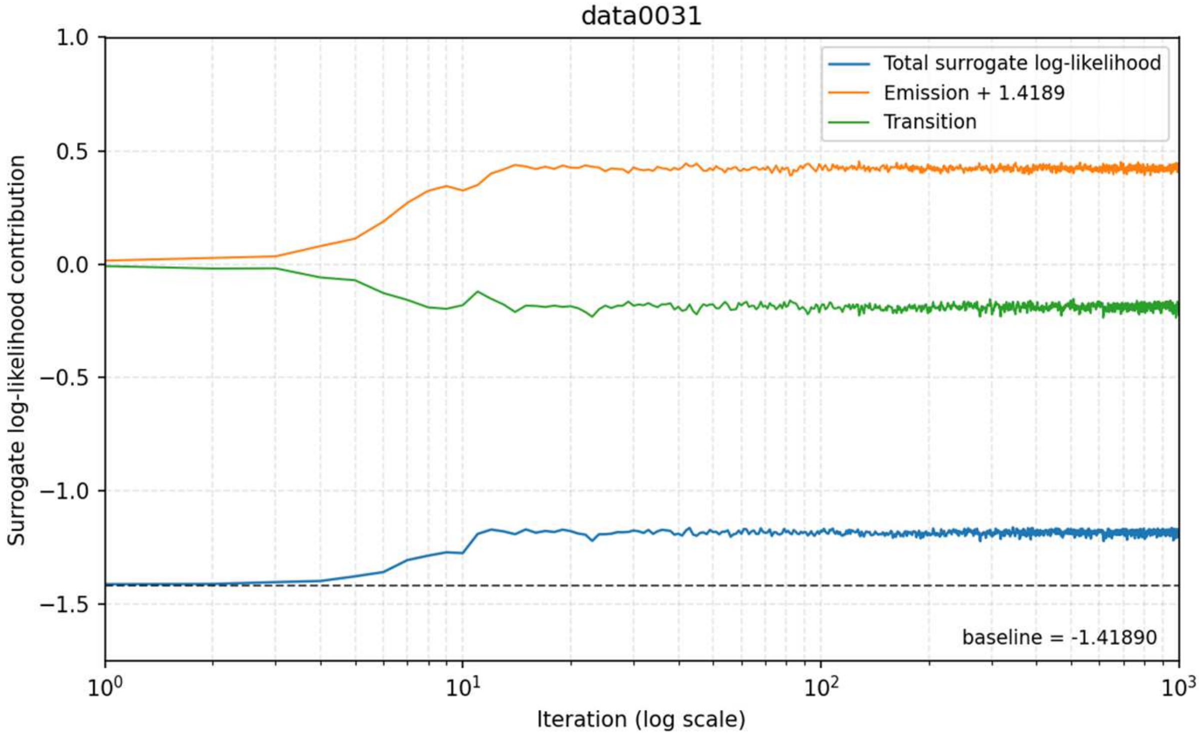
data0031
Total surrogate log-likelihood
Emission + 1.4189
Transition
Surrogate log-likelihood contribution
Iteration (log scale)
baseline = -1.41890
1.0
0.5
0.0
−0.5
−1.0
−1.5
10^0
10^1
10^2
10^3

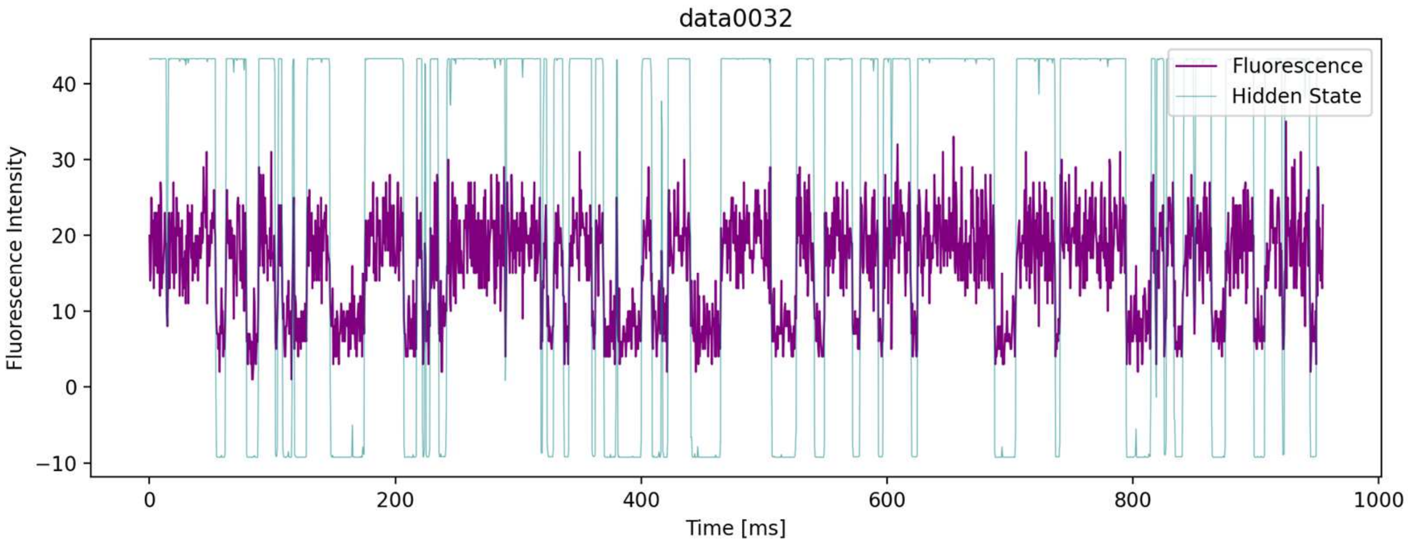
data0032
Fluorescence
Hidden State
Fluorescence Intensity
Time [ms]
0
200
400
600
800
1000
−10
0
10
20
30
40

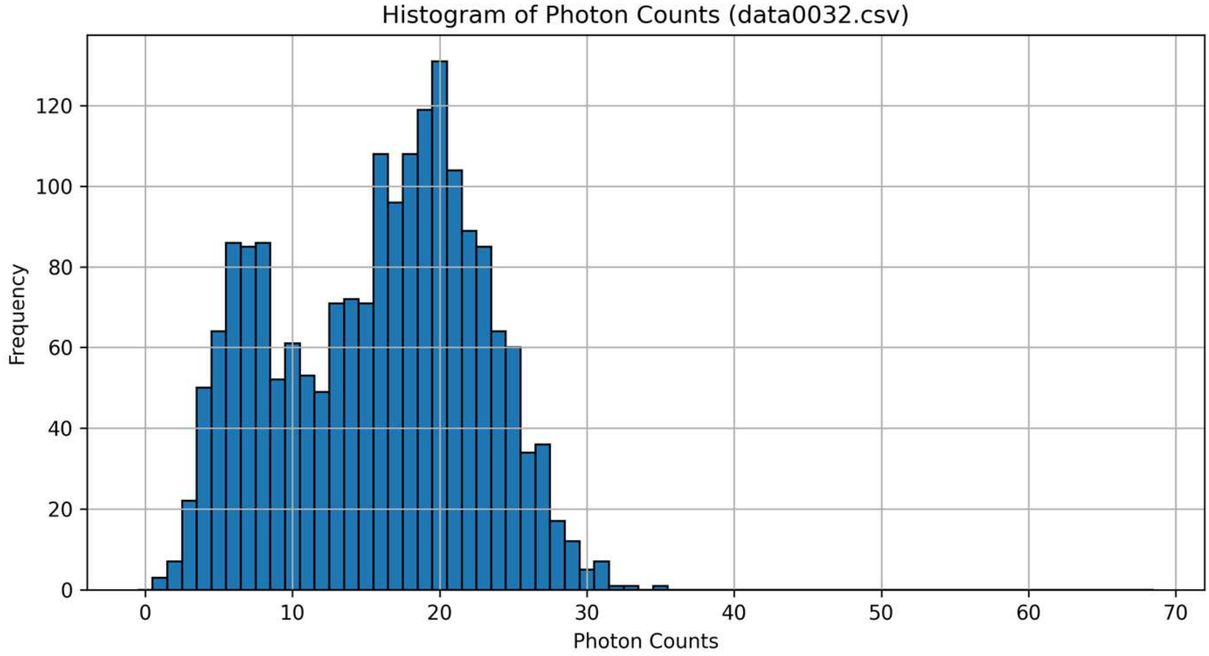
Histogram of Photon Counts (data0032.csv)
Frequency
Photon Counts
0
10
20
30
40
50
60
70
0
20
40
60
80
100
120

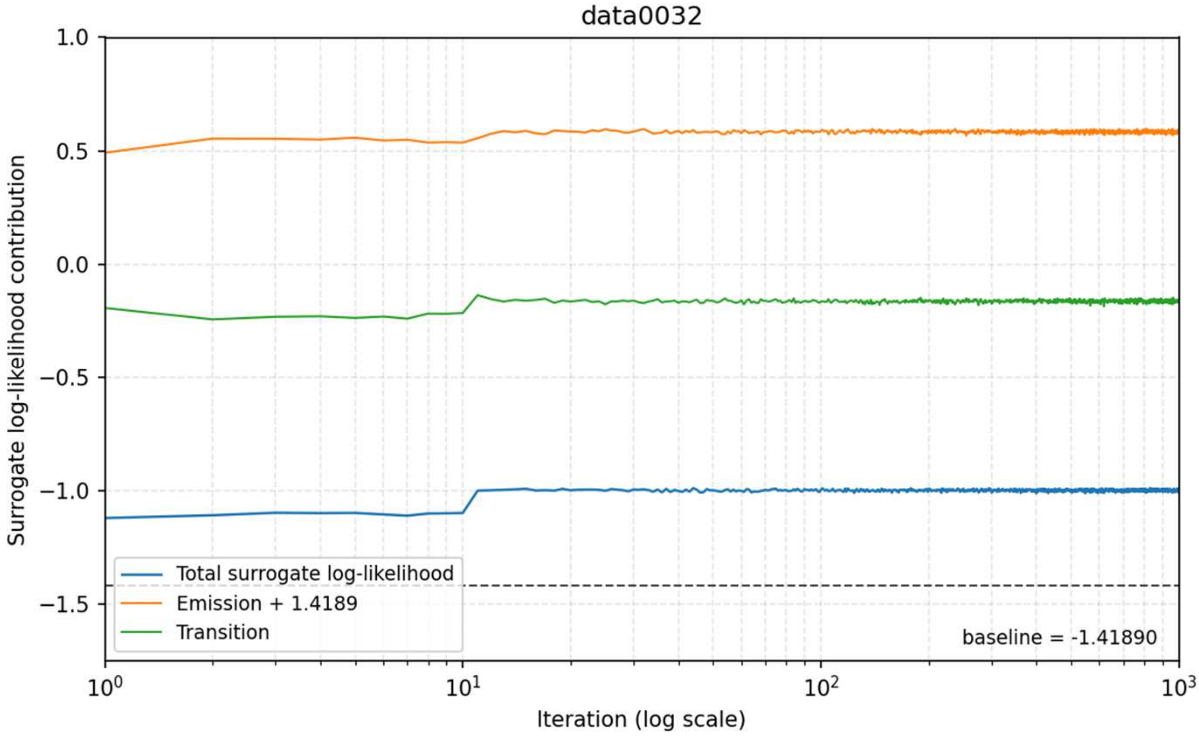
data0032
Surrogate log-likelihood contribution
Iteration (log scale)
Total surrogate log-likelihood
Emission + 1.4189
Transition
baseline = -1.41890
1.0
0.5
0.0
−0.5
−1.0
−1.5
10^0
10^1
10^2
10^3

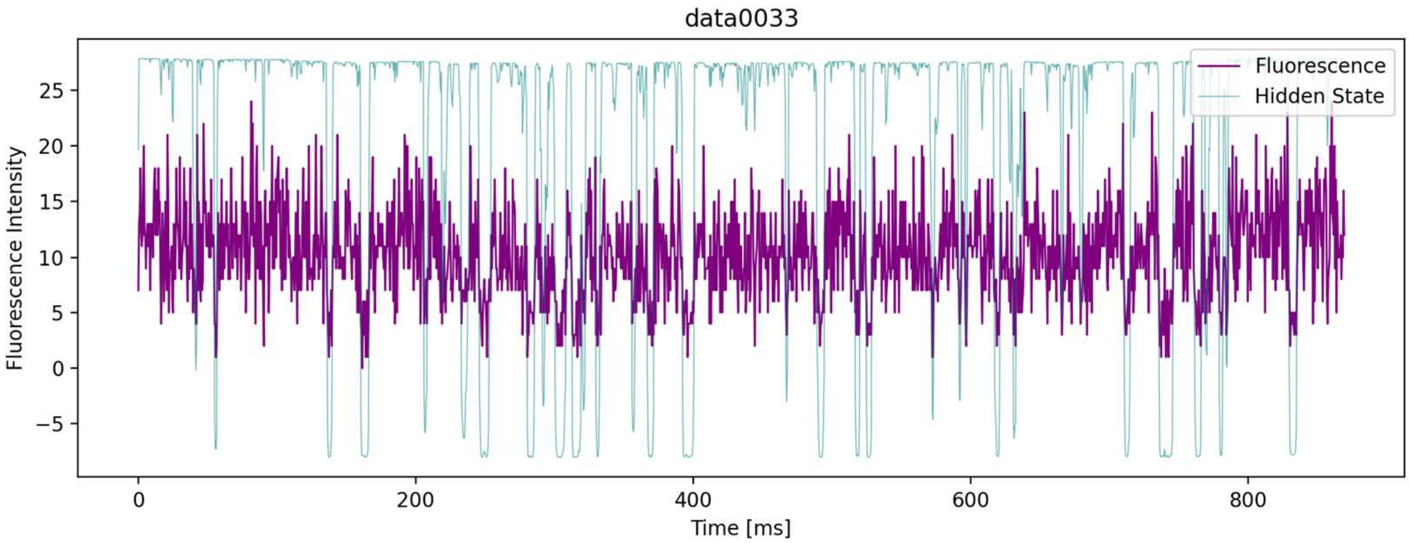
data0033
Fluorescence
Hidden State
Fluorescence Intensity
Time [ms]

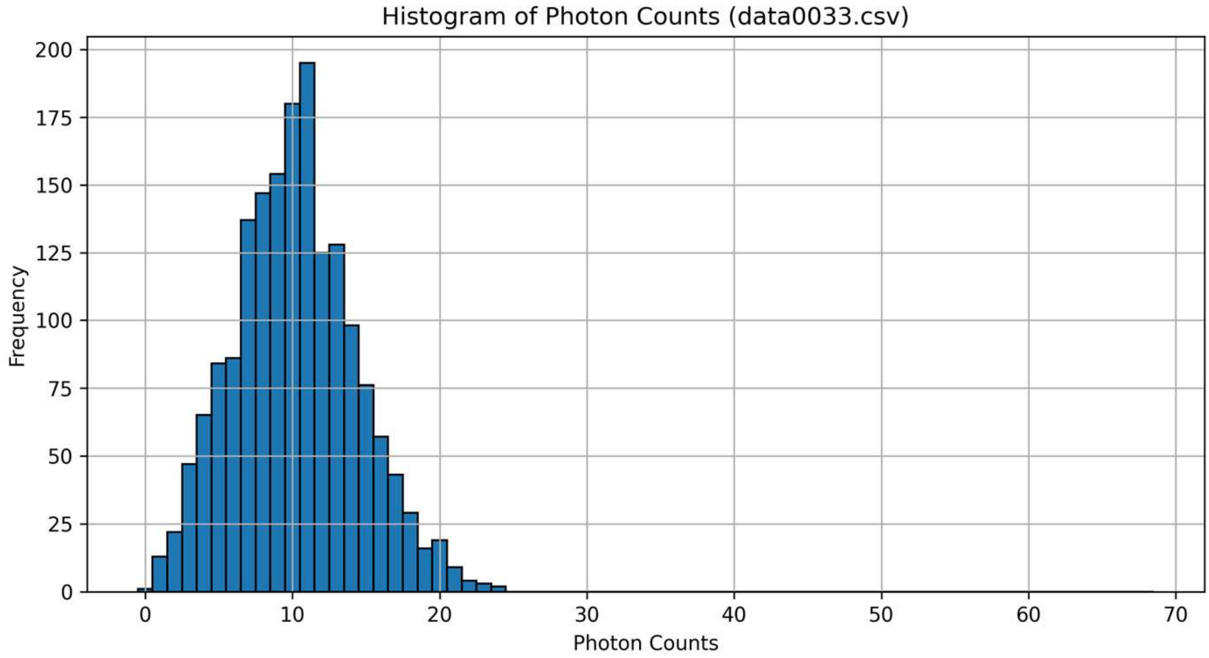
Histogram of Photon Counts (data0033.csv)
Frequency
Photon Counts

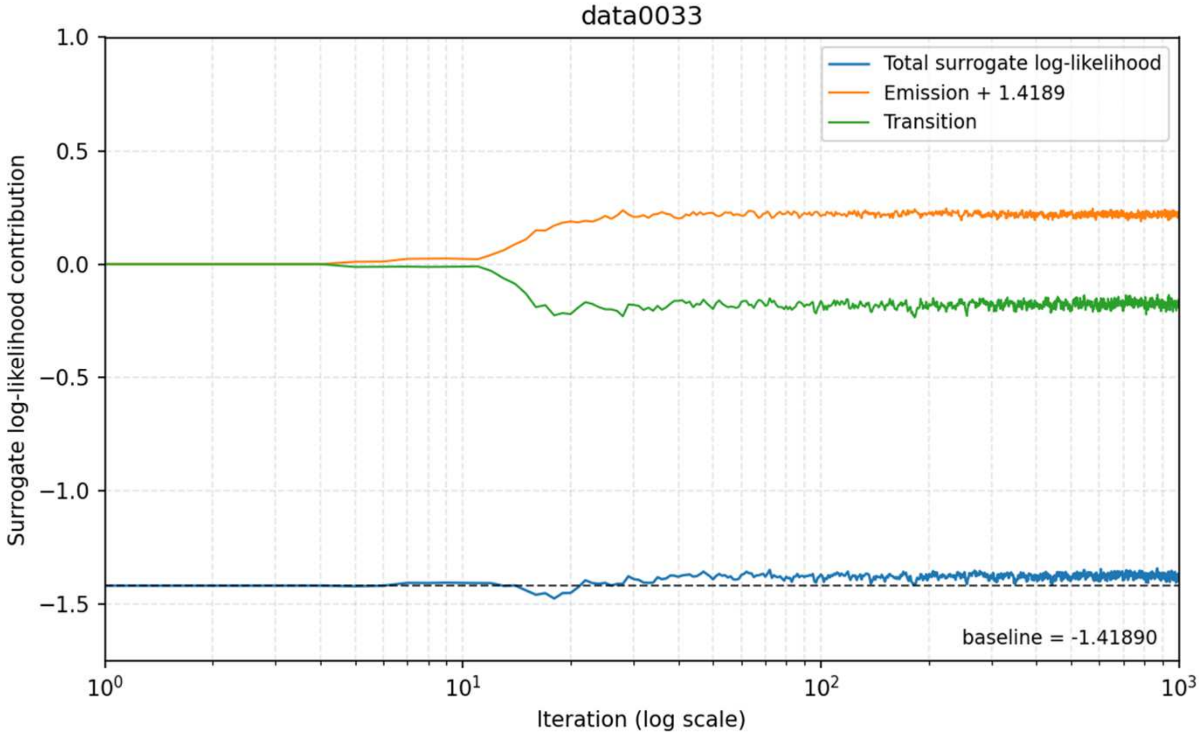
data0033
Total surrogate log-likelihood
Emission + 1.4189
Transition
Surrogate log-likelihood contribution
baseline = -1.41890
Iteration (log scale)

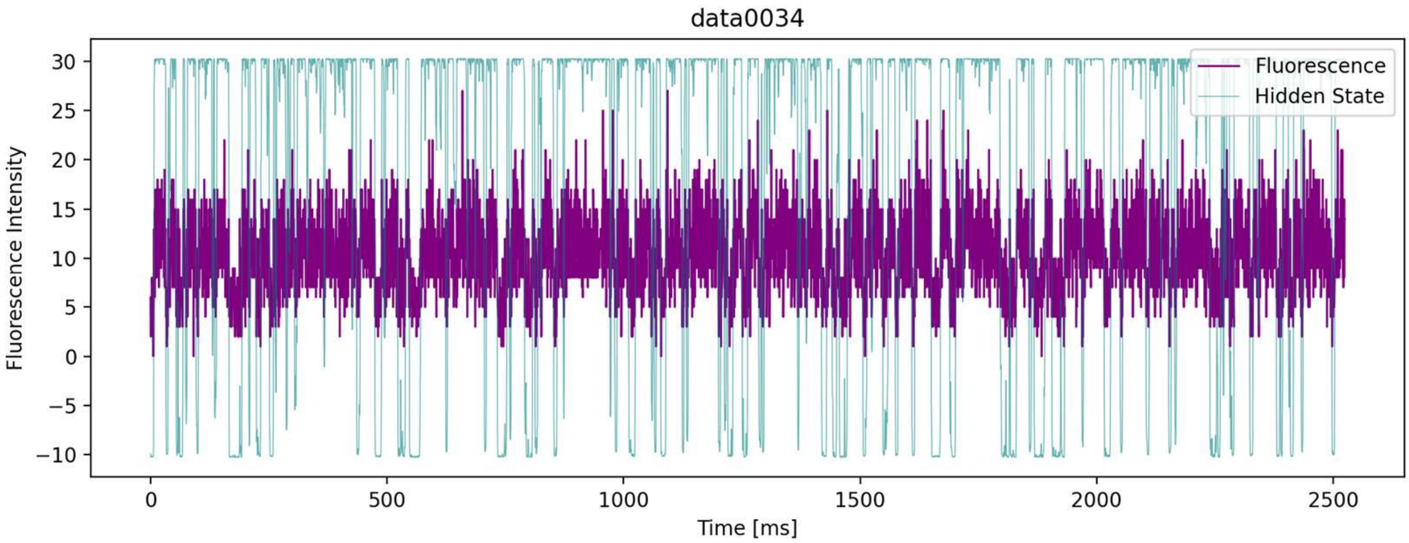
data0034
Fluorescence
Hidden State
Fluorescence Intensity
Time [ms]

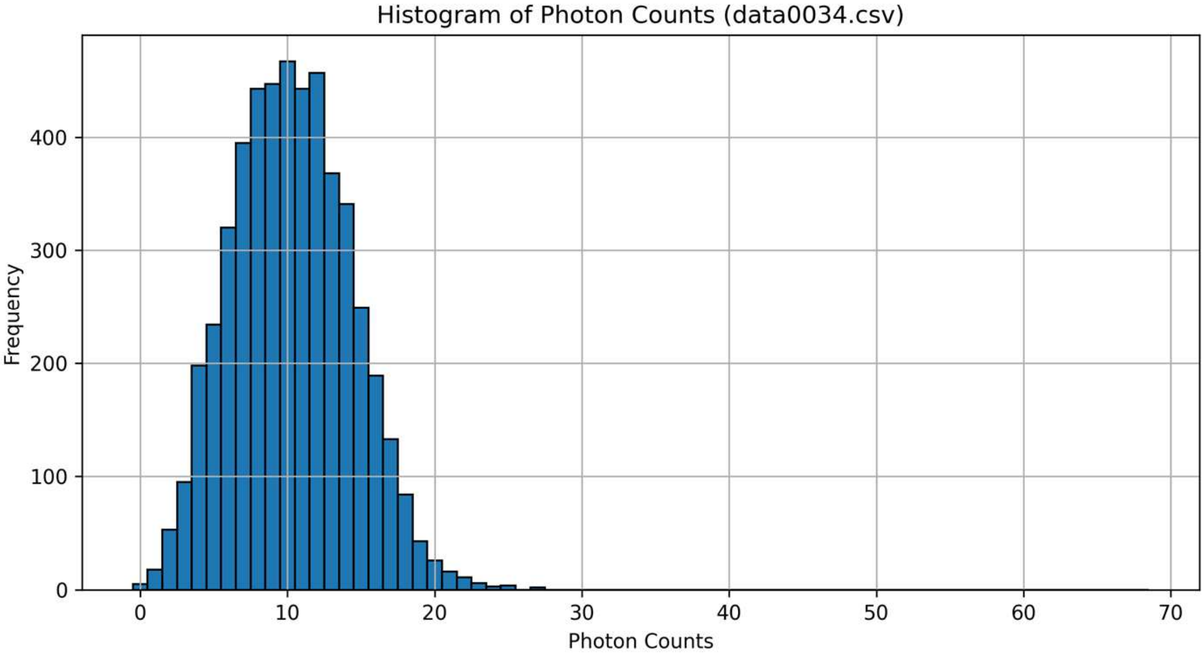
Histogram of Photon Counts (data0034.csv)
Frequency
Photon Counts

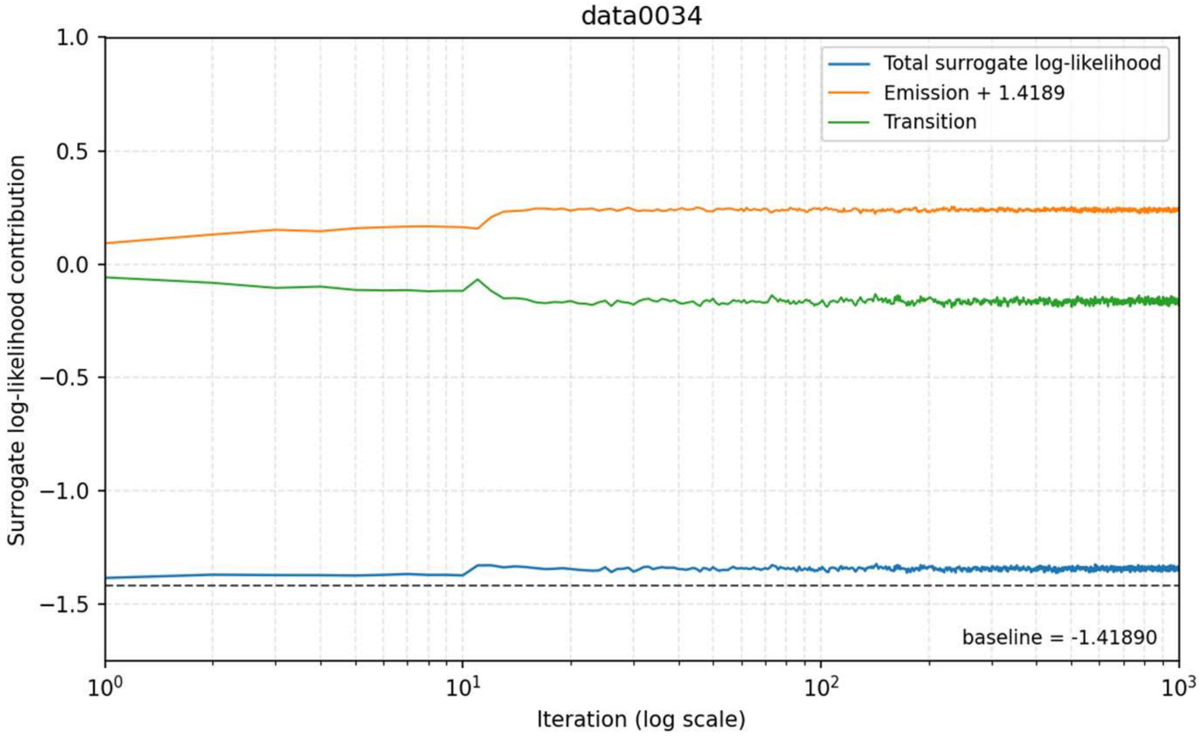
data0034
Total surrogate log-likelihood
Emission + 1.4189
Transition
Surrogate log-likelihood contribution
baseline = -1.41890
Iteration (log scale)

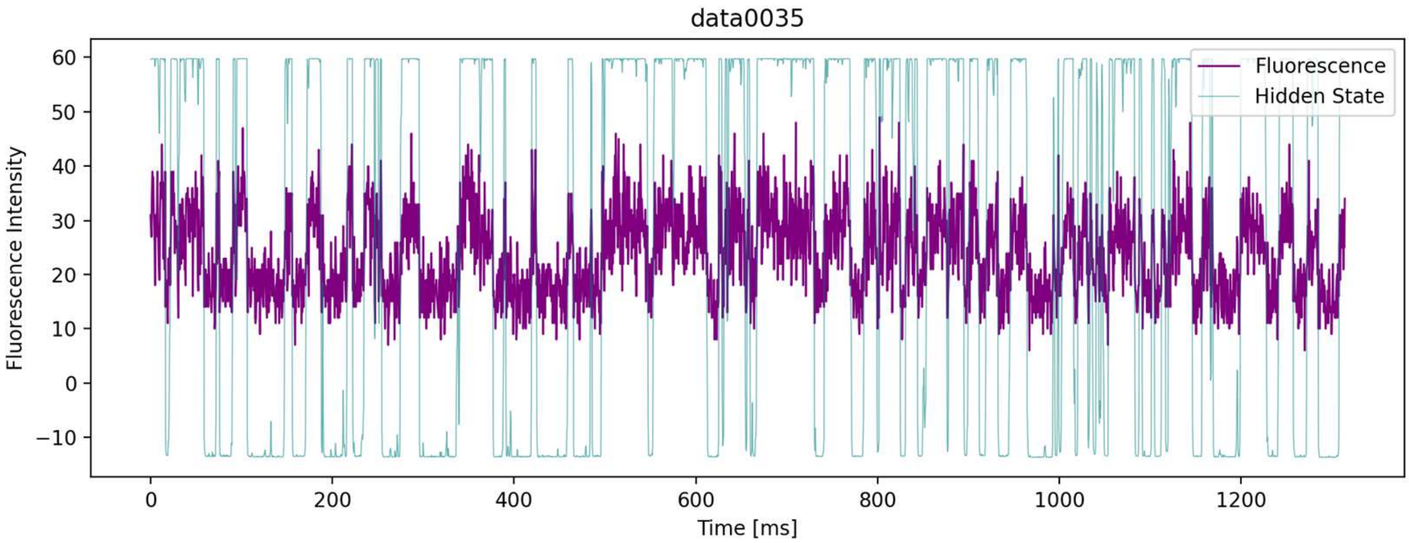
data0035
Fluorescence
Hidden State
Fluorescence Intensity
Time [ms]

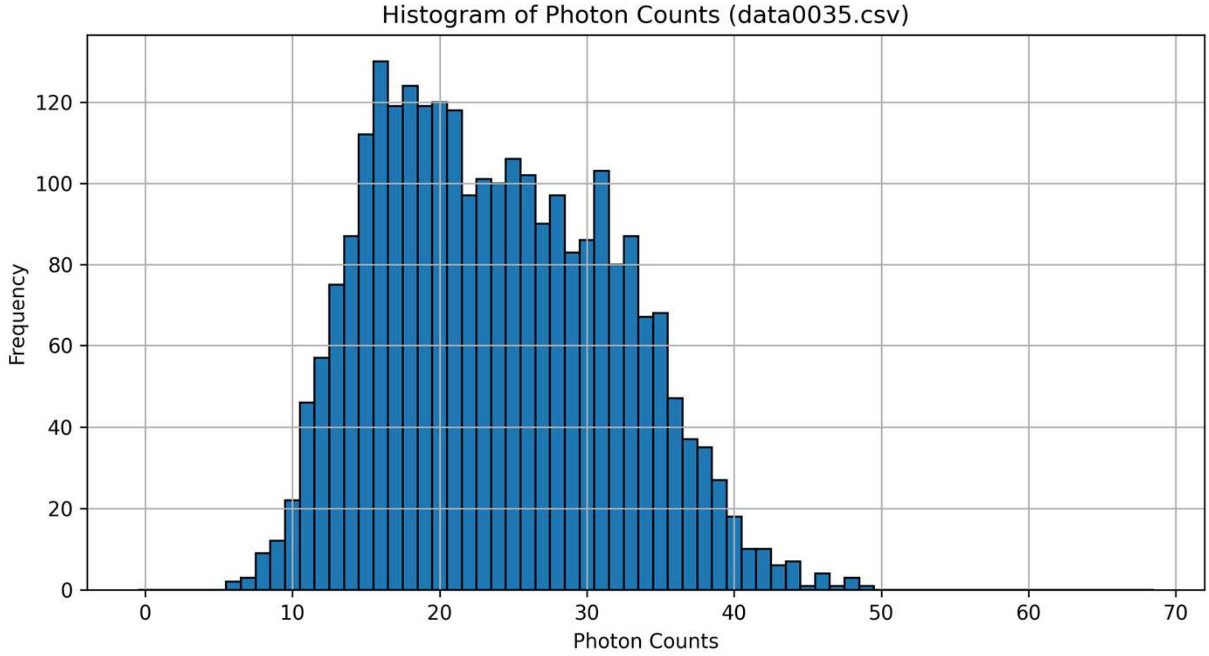
Histogram of Photon Counts (data0035.csv)
Frequency
Photon Counts

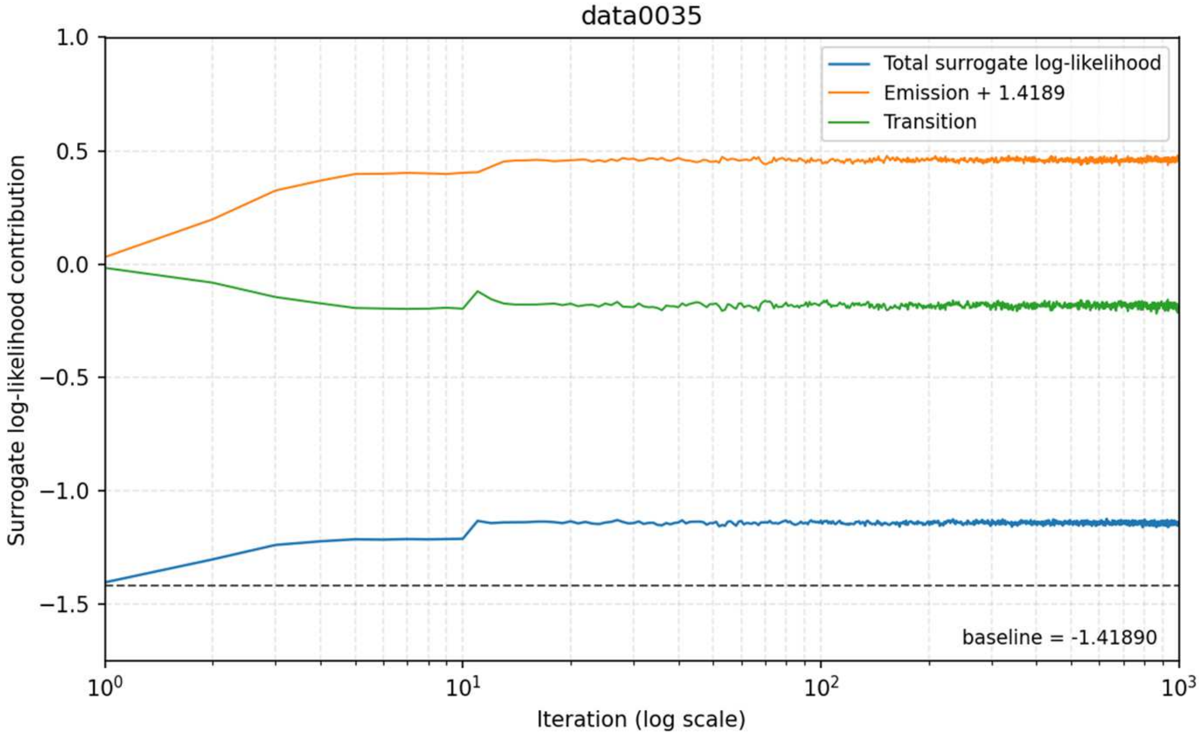
data0035
Total surrogate log-likelihood
Emission + 1.4189
Transition
Surrogate log-likelihood contribution
baseline = -1.41890
Iteration (log scale)

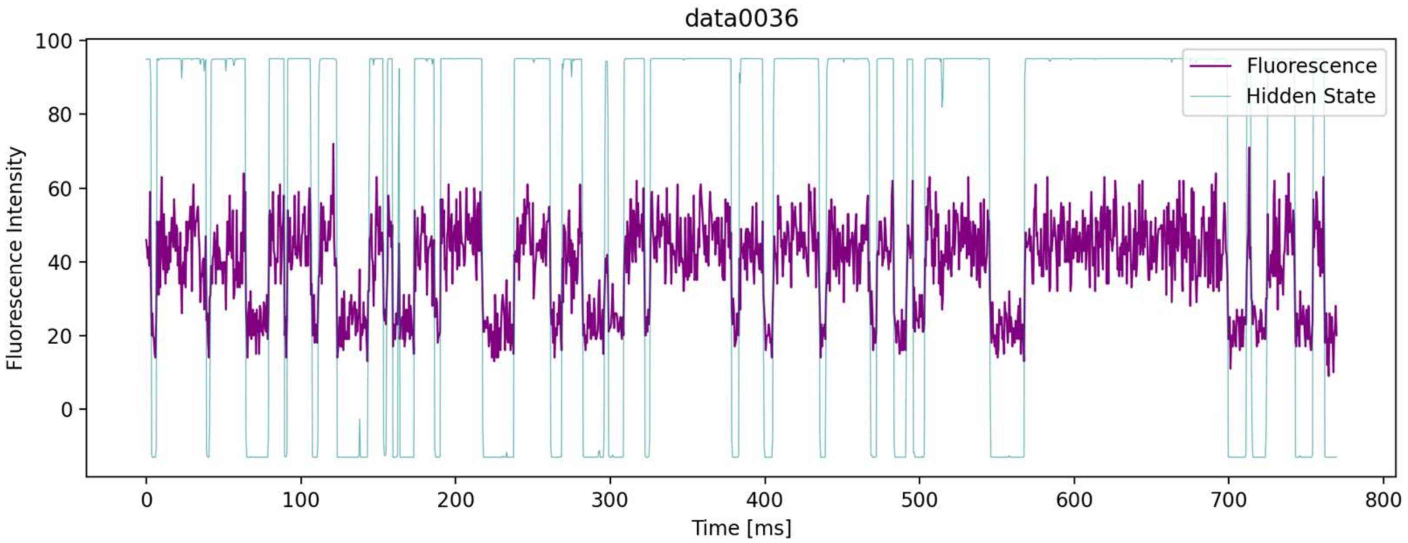
data0036
Fluorescence
Hidden State
Fluorescence Intensity
Time [ms]
0
100
200
300
400
500
600
700
800
20
40
60
80

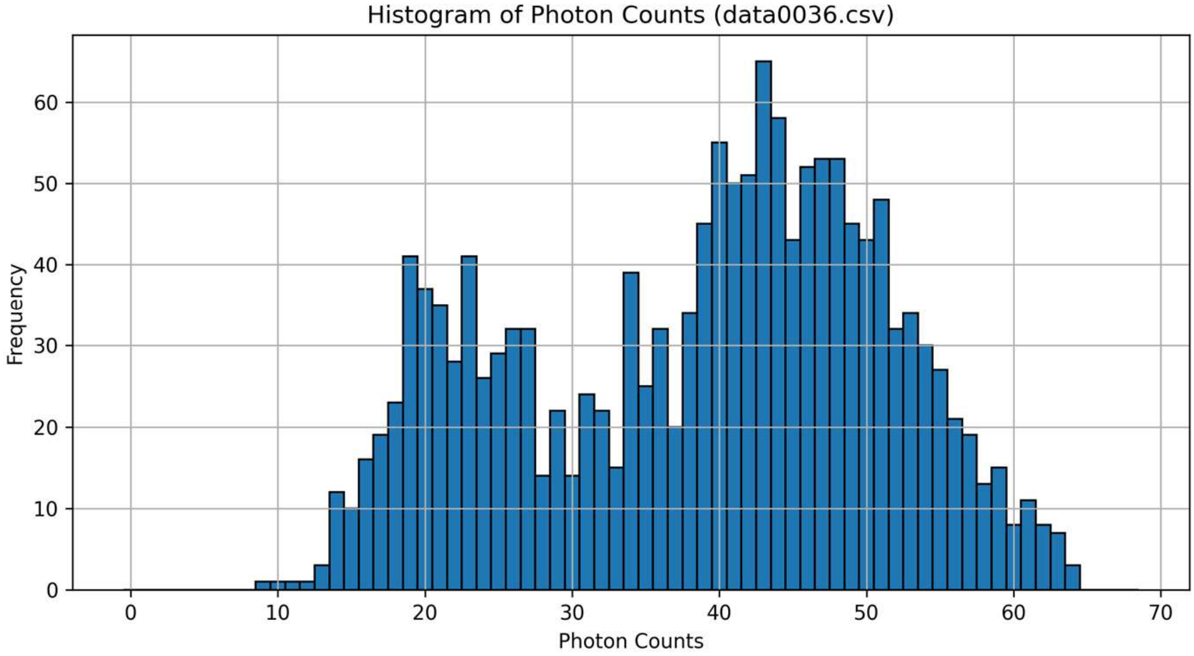
Histogram of Photon Counts (data0036.csv)
Frequency
Photon Counts
0
10
20
30
40
50
60
70

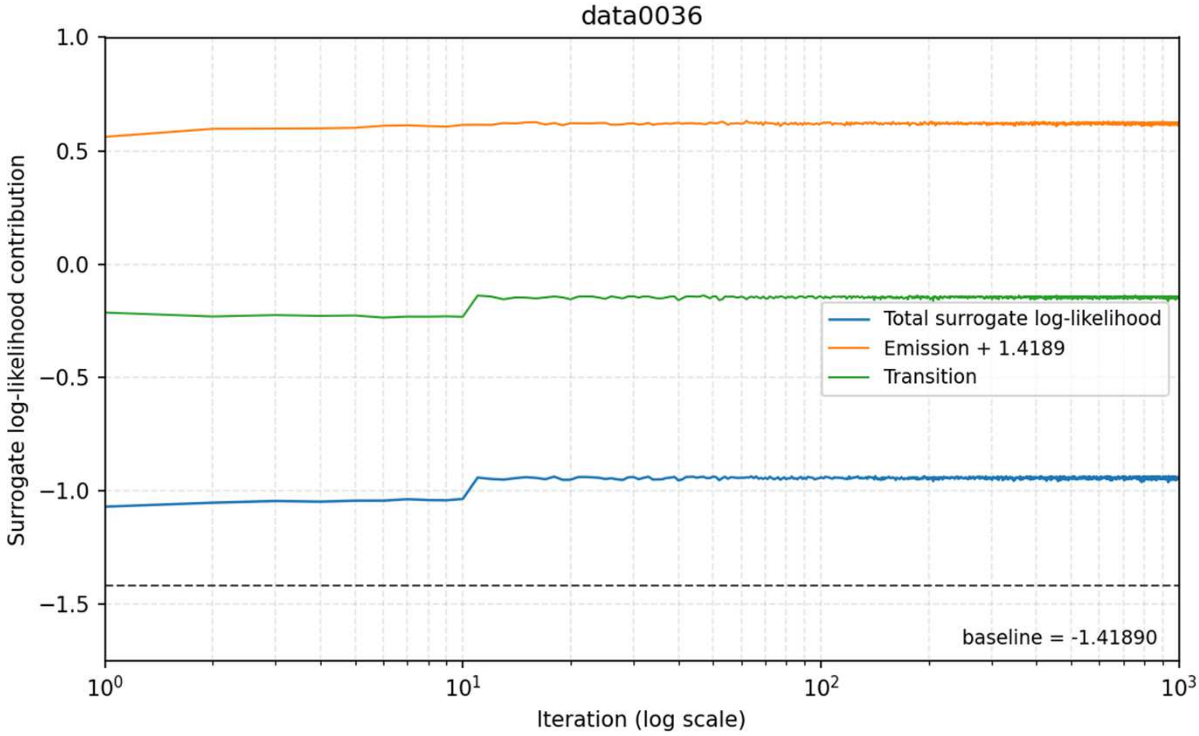
data0036
Total surrogate log-likelihood
Emission + 1.4189
Transition
Surrogate log-likelihood contribution
Iteration (log scale)
baseline = -1.41890
1.0
0.5
0.0
−0.5
−1.0
−1.5

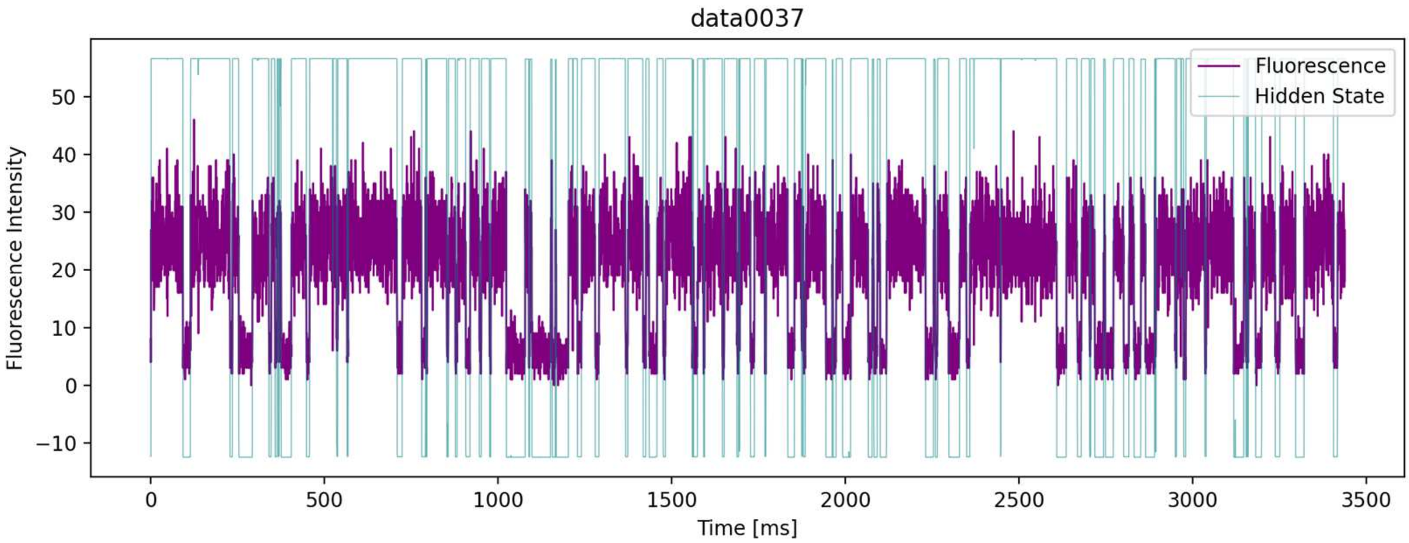
data0037
Fluorescence
Hidden State
Fluorescence Intensity
Time [ms]
50
40
30
20
10
0
−10
0
500
1000
1500
2000
2500
3000
3500

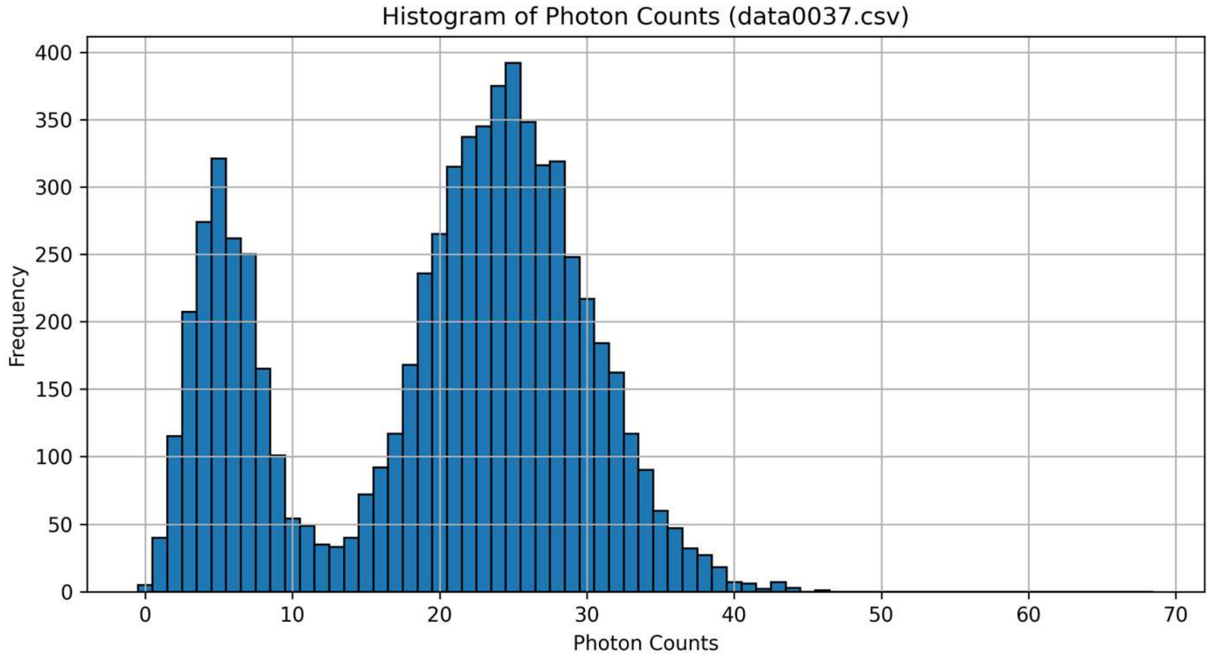
Histogram of Photon Counts (data0037.csv)
Frequency
Photon Counts
400
350
300
250
200
150
100
50
0
0
10
20
30
40
50
60
70

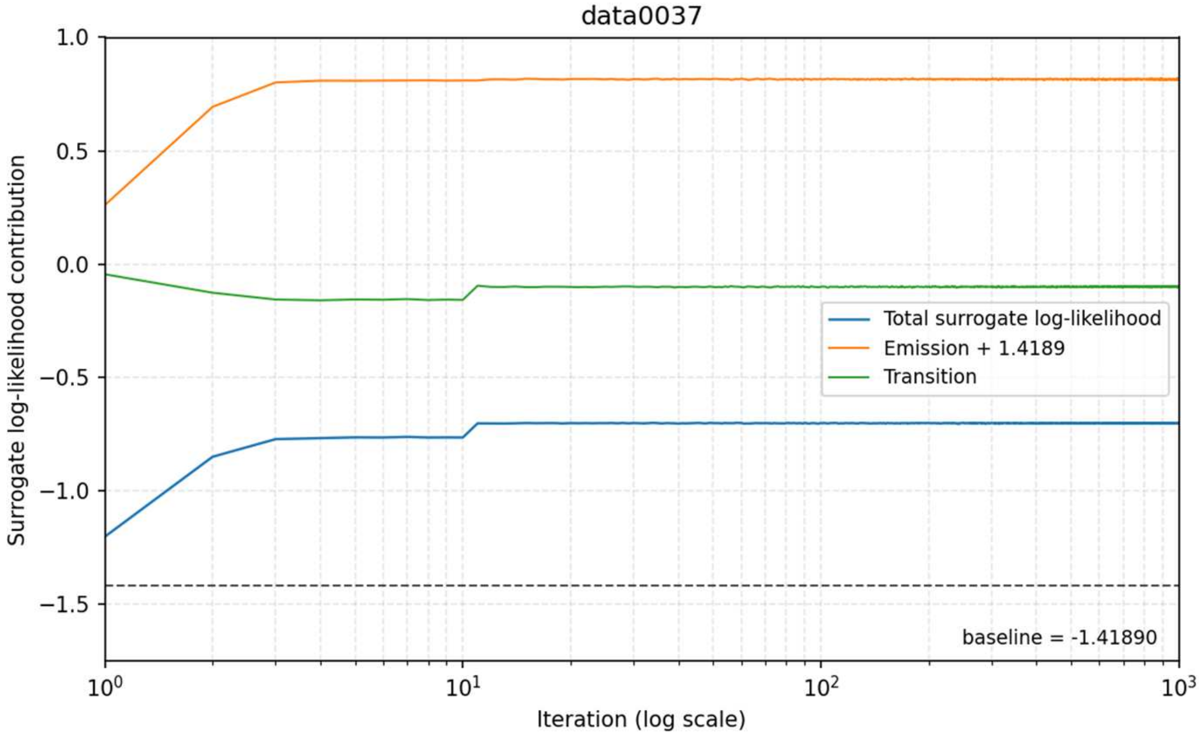
data0037
Total surrogate log-likelihood
Emission + 1.4189
Transition
Surrogate log-likelihood contribution
Iteration (log scale)
baseline = -1.41890
1.0
0.5
0.0
−0.5
−1.0
−1.5

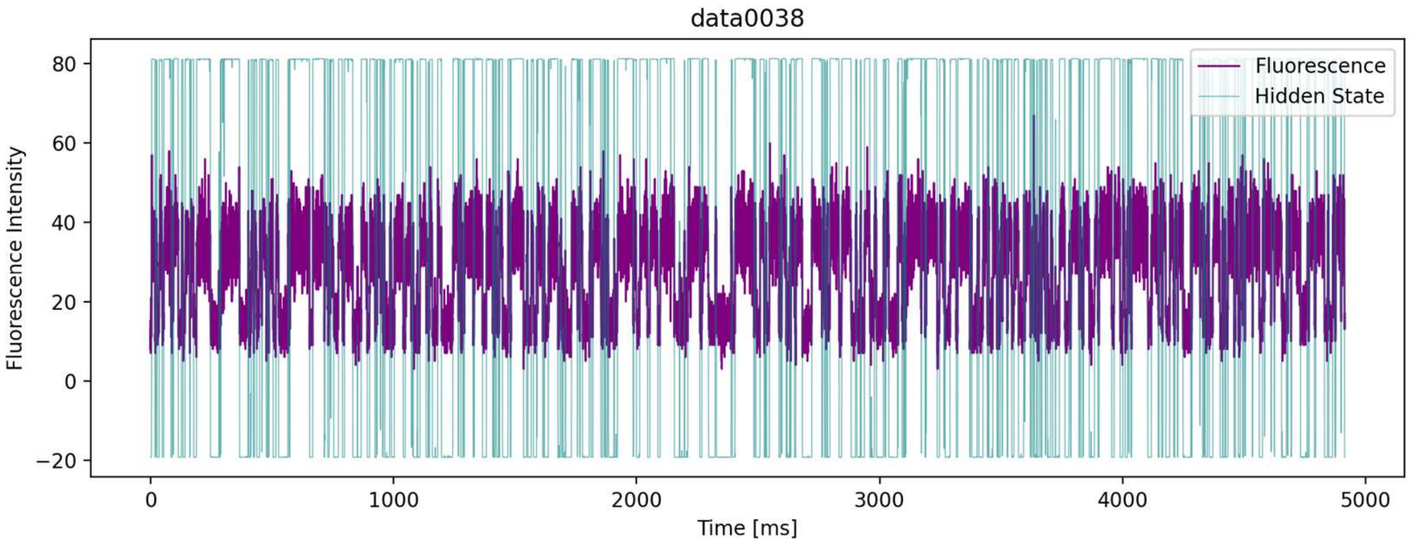
data0038
Fluorescence
Hidden State
Fluorescence Intensity
Time [ms]

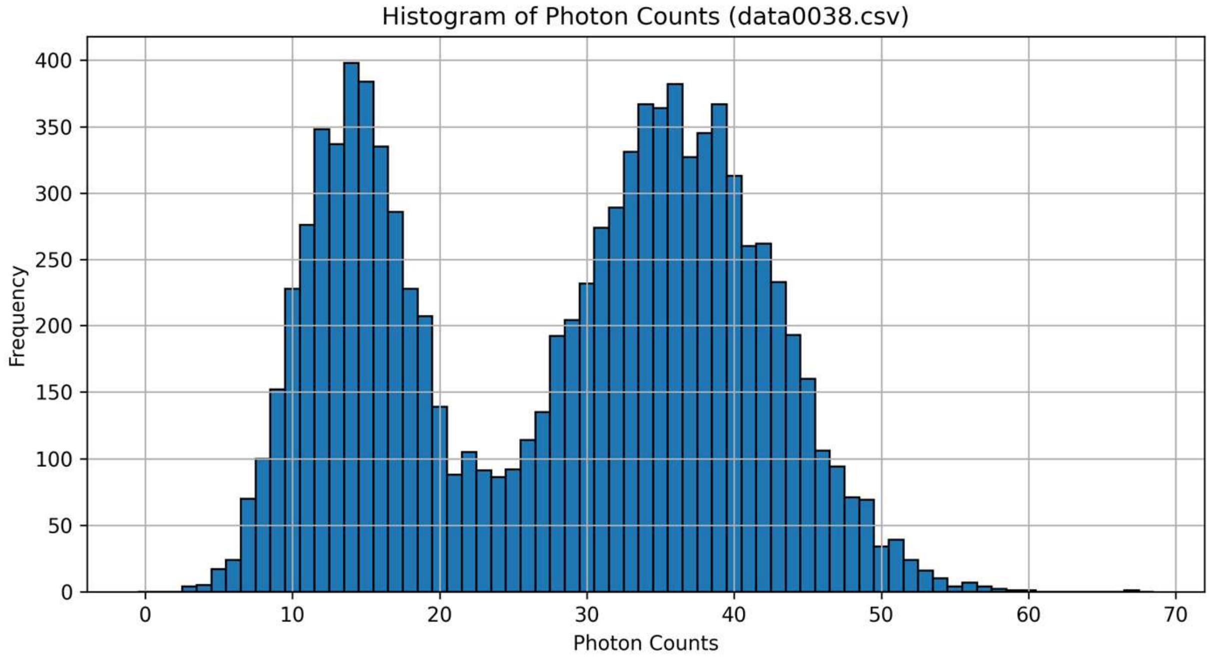
Histogram of Photon Counts (data0038.csv)
Frequency
Photon Counts

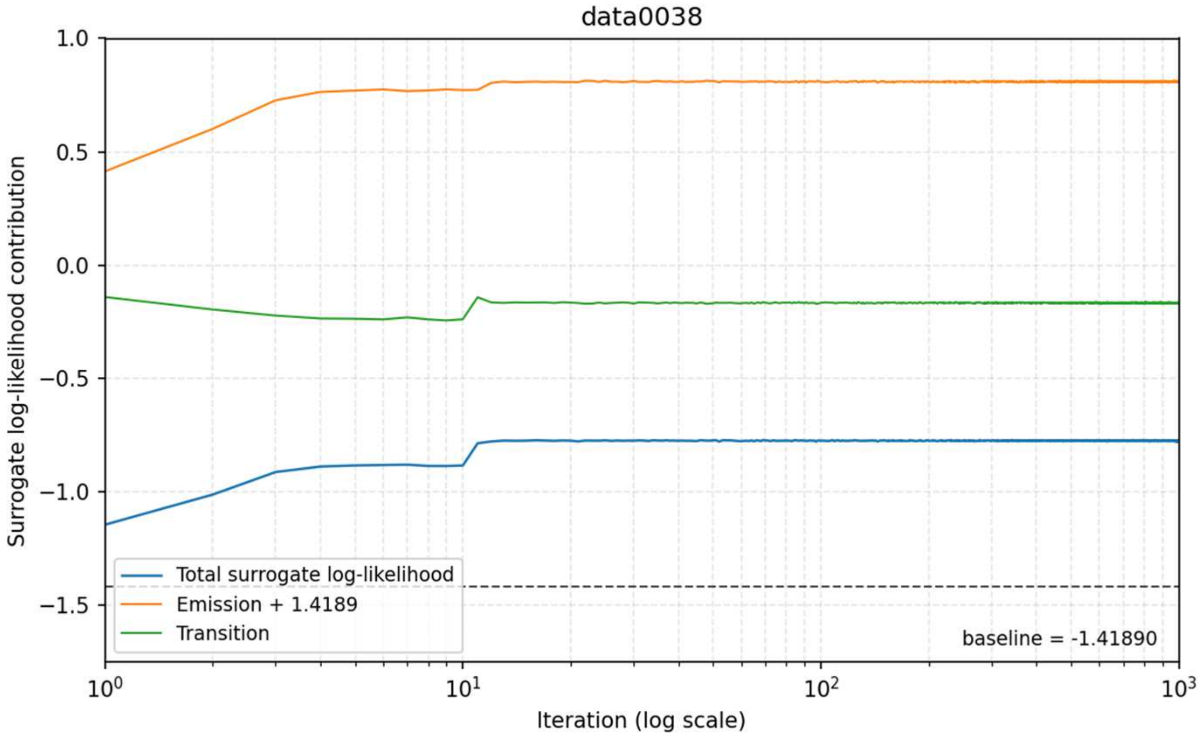
data0038
Surrogate log-likelihood contribution
Total surrogate log-likelihood
Emission + 1.4189
Transition
baseline = -1.41890
Iteration (log scale)

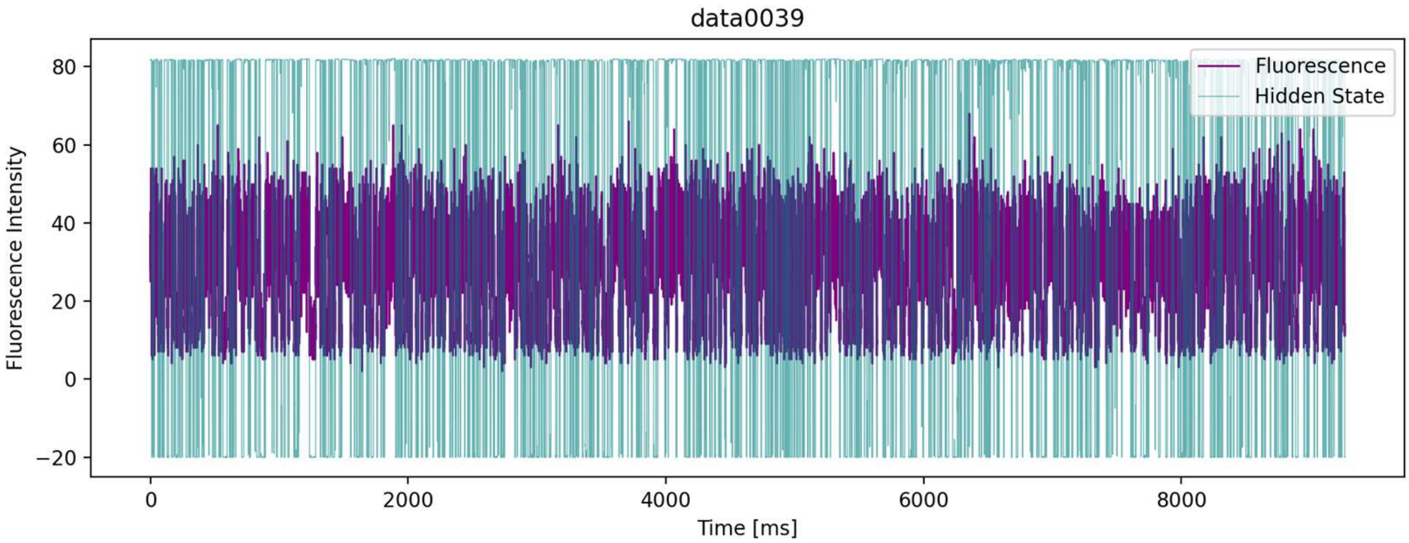
data0039
Fluorescence
Hidden State
Fluorescence Intensity
Time [ms]
80
60
40
20
0
−20
0
2000
4000
6000
8000

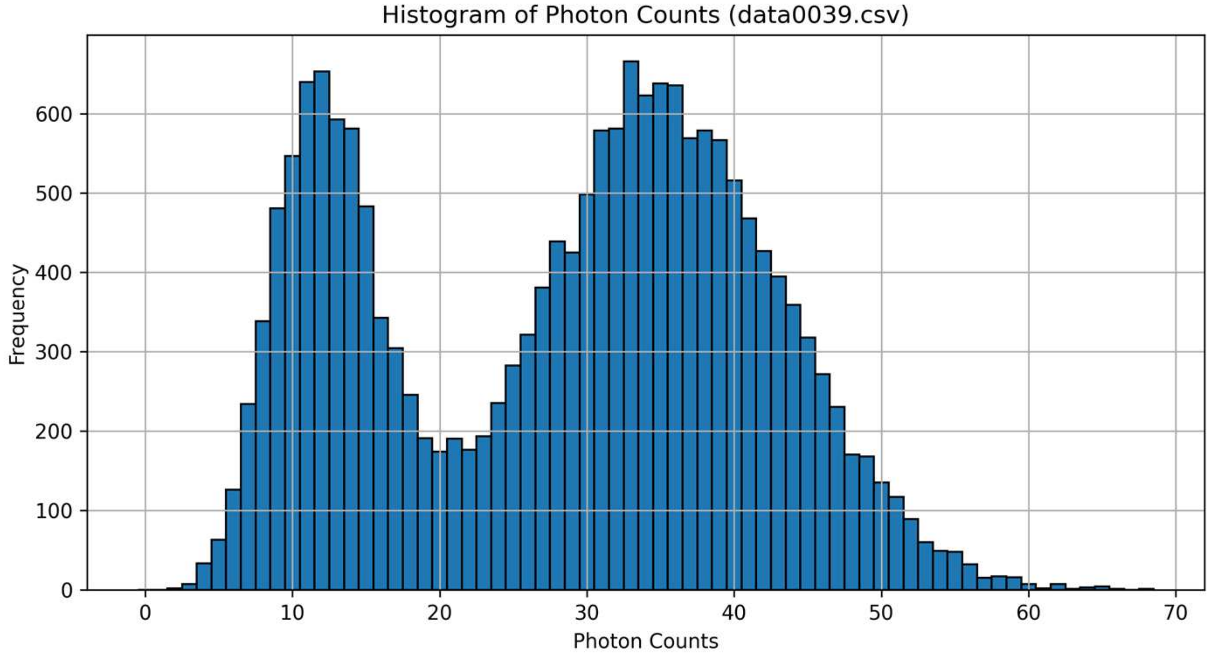
Histogram of Photon Counts (data0039.csv)
Frequency
Photon Counts
600
500
400
300
200
100
0
0
10
20
30
40
50
60
70

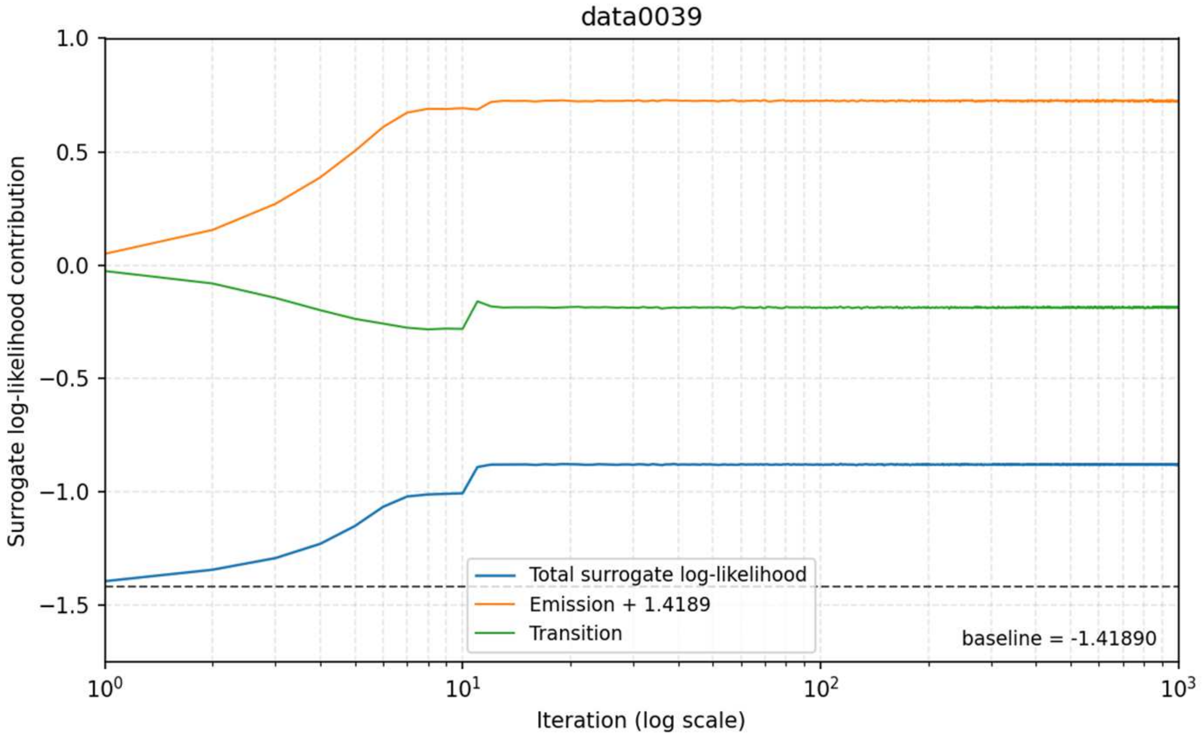
data0039
Surrogate log-likelihood contribution
Iteration (log scale)
Total surrogate log-likelihood
Emission + 1.4189
Transition
baseline = -1.41890
1.0
0.5
0.0
−0.5
−1.0
−1.5
10^0
10^1
10^2
10^3

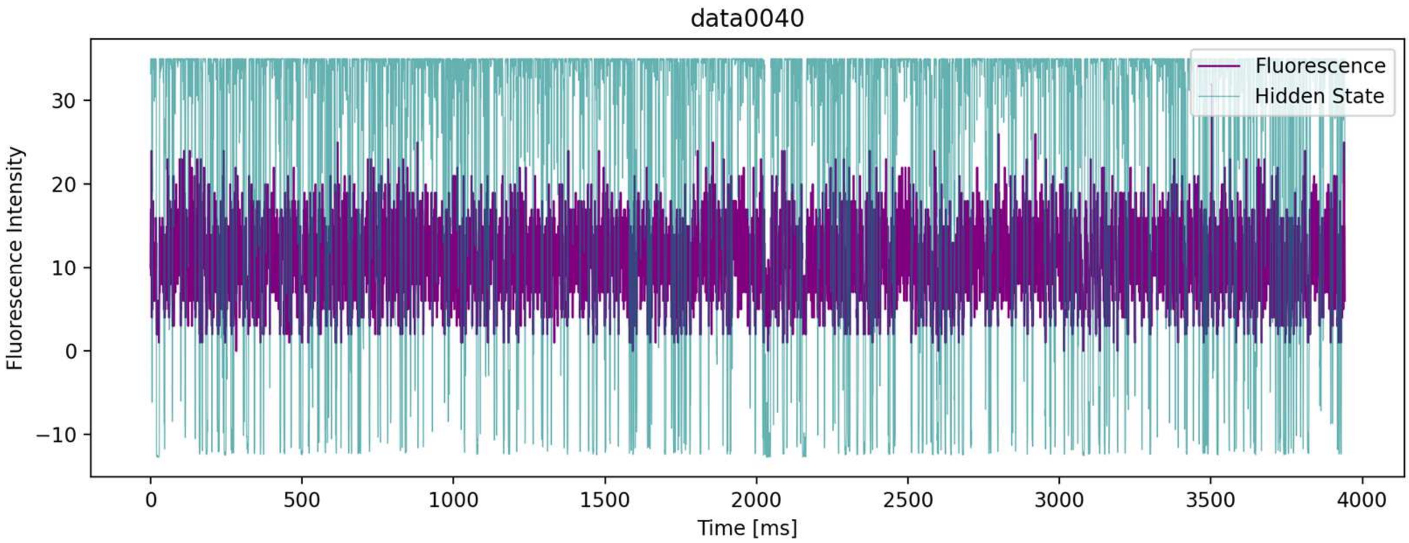

data0040
Fluorescence
Hidden State
Fluorescence Intensity
Time [ms]

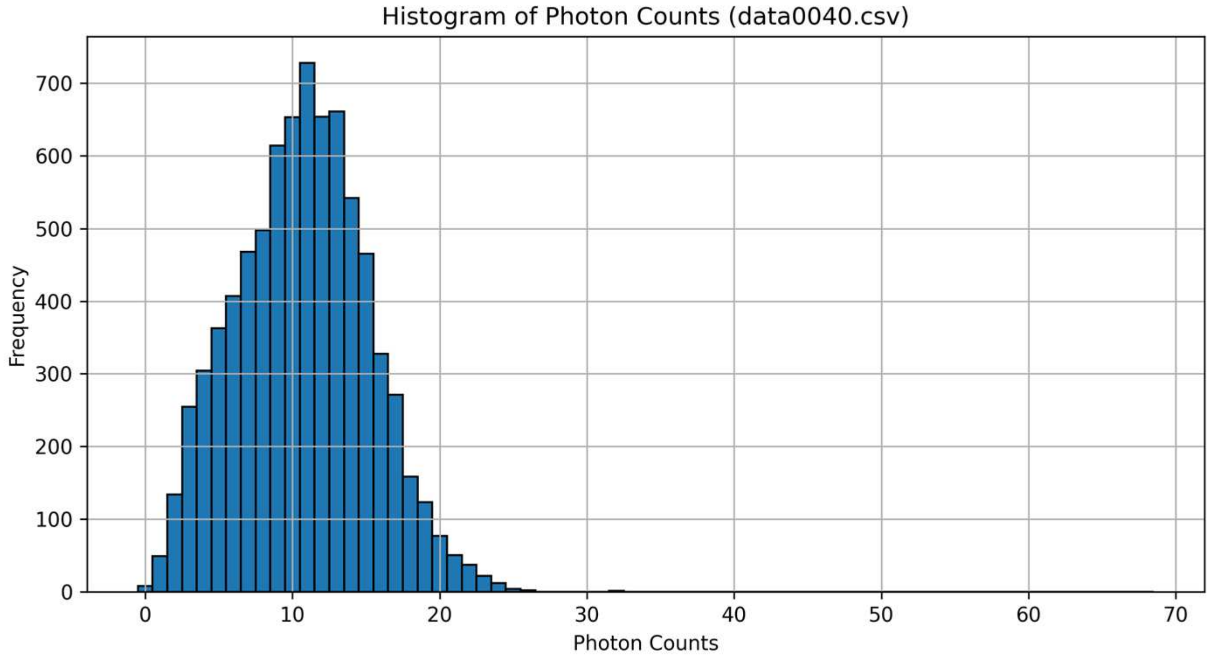

Histogram of Photon Counts (data0040.csv)
Frequency
Photon Counts

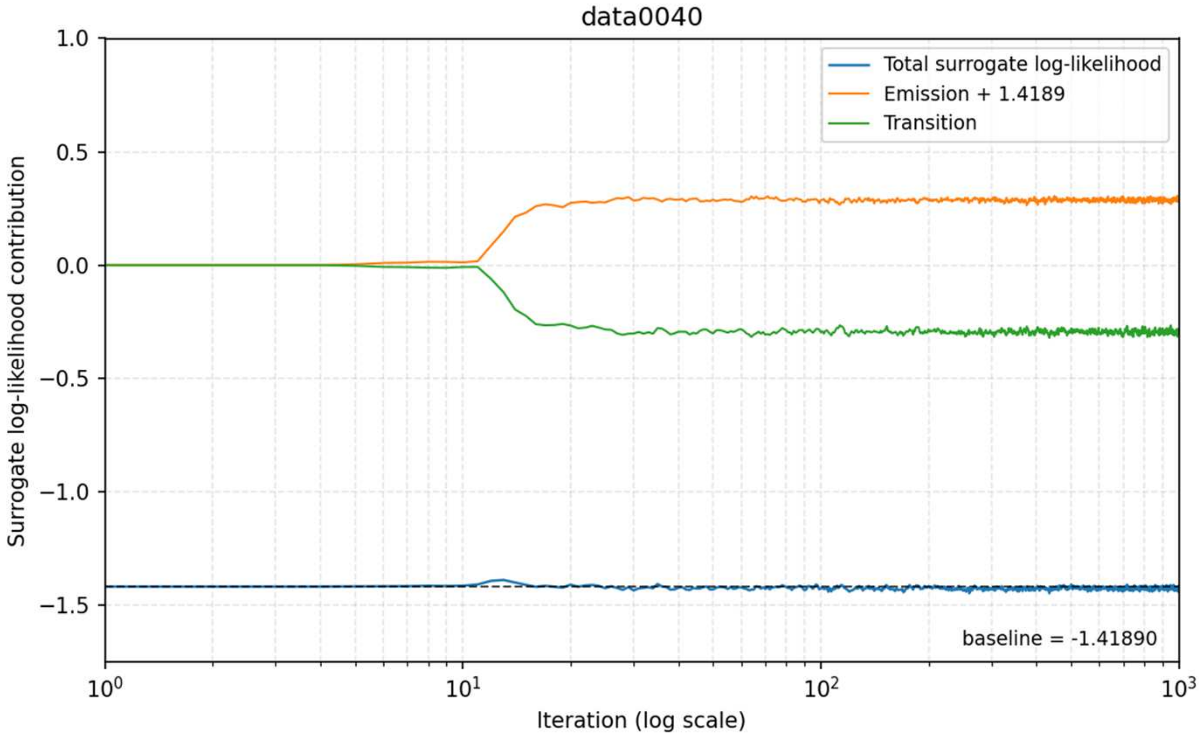

data0040
Total surrogate log-likelihood
Emission + 1.4189
Transition
Surrogate log-likelihood contribution
baseline = -1.41890
Iteration (log scale)
